# Efficacy and safety of CNM-Au8 in amyotrophic lateral sclerosis (RESCUE-ALS study): a phase 2, randomised, double-blind, placebo-controlled trial and open label extension

**DOI:** 10.1016/j.eclinm.2023.102036

**Published:** 2023-06-08

**Authors:** Steve Vucic, Parvathi Menon, William Huynh, Colin Mahoney, Karen S. Ho, Alan Hartford, Austin Rynders, Jacob Evan, Jeremy Evan, Shelia Ligozio, Robert Glanzman, Michael T. Hotchkin, Matthew C. Kiernan

**Affiliations:** a Brain and Nerve Research Centre, Concord Clinical School and Department of Neurology, Concord Repatriation General Hospital, The University of Sydney, Sydney, Australia; b Brain and Mind Centre, University of Sydney, and Department of Neurology, Royal Prince Alfred Hospital, Sydney, Australia; c Clene Nanomedicine, Inc., Salt Lake City, UT, USA; d Instat Clinical Research, Chatham, NJ, USA

**Keywords:** ALS, CNM-Au8, MUNIX, Survival

## Abstract

**Background:**

CNM-Au8® is a catalytically-active gold nanocrystal neuroprotective agent that enhances intracellular energy metabolism and reduces oxidative stress. The phase 2, randomised, double-blind, placebo-controlled trial and open label extension RESCUE-ALS trial evaluated the efficacy and safety of CNM-Au8 for treatment of amyotrophic lateral sclerosis (ALS).

**Methods:**

RESCUE-ALS and its long-term open label extension (OLE) were conducted at two multidisciplinary ALS clinics located in Sydney, Australia: (i) the Brain and Mind Centre and (ii) Westmead Hospital. The double-blind portion of RESCUE-ALS was conducted from January 16, 2020 (baseline visit, first-patient first-visit (FPFV)) through July 13, 2021 (double-blind period, last-patient last-visit (LPLV)). Participants (N = 45) were randomised 1:1 to receive 30 mg of CNM-Au8 or matching placebo daily over 36 weeks in addition to background standard of care, riluzole. The primary outcome was mean percent change in summed motor unit number index (MUNIX), a sensitive neurophysiological biomarker of lower motor neuron function. Change in total (or summated) MUNIX score and change in forced vital capacity (FVC) were secondary outcome measures. ALS disease progression events, ALS Functional Rating Scale (ALSFRS-R) change, change in quality of life (ALSSQOL-SF) were assessed as exploratory outcome measures. Long-term survival evaluated vital status of original active versus placebo randomisation for all participants through at least 12 months following last-patient last-visit (LPLV) of the double-blind period. RESCUE-ALS and the open label study are registered in clinicaltrials.gov with registration numbers NCT04098406 and NCT05299658, respectively.

**Findings:**

In the intention-to-treat (ITT) population, there was no significant difference in the summated MUNIX score percent change (LS mean difference: 7.7%, 95% CI: −11.9 to 27.3%, p = 0.43), total MUNIX score change (18.8, 95% CI: −56.4 to 94.0), or FVC change (LS mean difference: 3.6, 95% CI: −12.4 to 19.7) between the active and placebo treated groups at week 36. In contrast, survival analyses through 12-month LPLV demonstrated a 60% reduction in all-cause mortality with CNM-Au8 treatment [hazard ratio = 0.408 (95% Wald CI: 0.166 to 1.001, log-rank p = 0.0429). 36 participants entered the open label extension (OLE), and those initially randomised to CNM-Au8 exhibited a slower rate of disease progression, as measured by time to the occurrence of death, tracheostomy, initiation of non-invasive ventilatory support, or gastrostomy tube placement. CNM-Au8 was well-tolerated, and no safety signals were observed.

**Interpretation:**

CNM-Au8, in combination with riluzole, was well-tolerated in ALS with no identified safety signals. While the primary and secondary outcomes of this trial were not significant, the clinically meaningful exploratory results support further investigation of CNM-Au8 in ALS.

**Funding:**

The RESCUE-ALS was substantially funded by a grant from 10.13039/100014012FightMND. Additional funding was provided by Clene Australia Pty Ltd.

**Research in context**

**Evidence before this study**

Amyotrophic lateral sclerosis (ALS) is an adult-onset neurodegenerative disorder involving the progressive loss of motor neurons. Despite numerous clinical trials investigating a wide range of therapeutic interventions, only three disease-modifying ALS treatments, riluzole, edaravone, and sodium phenylbutyrate/taurursodiol have been approved by the US FDA, whilst only riluzole is available in Europe. There is an exigent need for (i) additional effective ALS treatments, and (ii) validation of sensitive neurophysiological biomarkers predictive of clinical efficacy to support efficient proof-of-concept development programs. To assess previous research ALS research strategies, we completed a non-systematic PubMed search using a combination of terms including, ‘ALS’, ‘neurophysiology’, ‘clinical trial’, and ‘electromyography’. The search included clinical observations and non-interventional research with a search date cut-off of March 30, 2019. No prior clinical trials reported use of neurophysiology as a primary outcome.

**Added value of this study**

CNM-Au8, a novel cellular energetic nanocatalyst, was investigated in a Phase 2 proof-of-concept clinical trial over 36 weeks of randomised double-blind treatment utilizing a novel primary outcome based on neurophysiological estimation of lower motor neuron preservation. CNM-Au8, in combination with riluzole, was well-tolerated and no safety issues were identified. While the primary and secondary outcomes did not demonstrate a statistical difference, exploratory clinical outcome measures and improved long-term survival suggested slowed disease progression.

**Implications of all the available evidence**

Neurophysiology endpoints can be successfully employed in focused proof-of-concept ALS clinical trials to provide evidence for further clinical development in adequately powered clinical studies. Further investigation of CNM-Au8 to slow ALS disease progression, and cellular energetic support as therapeutic strategy for neurodegeneration is warranted.

## Introduction

Amyotrophic lateral sclerosis (ALS) is a progressive neurodegenerative disorder of motor neurons with a median survival of three to five years from symptom onset.^1^^,^^2^ Three disease-modifying treatments, riluzole, edaravone, and sodium phenylbutyrate/taurursidiol, have been approved by the US Food and Drug Administration, while only riluzole has been approved by the European Medicines Agency. Given the number of large ALS clinical trials that have failed to demonstrate clinical benefit,^3^^,^^4^ there is an exigent need for novel and more effective ALS treatments.

Impaired energy metabolism and cytoplasmic accumulation of TAR DNA-binding protein 43 (TDP-43) aggregates are key aspects of ALS pathophysiology.^5^^,^^6^ Both oxidative phosphorylation and glycolysis, the main pathways for ATP energy generation in all cells, appear compromised in motor neurons derived from ALS patients.^7^ Addressing these energy metabolism deficits may alleviate TDP-43 mislocalisation and aggregation, and could serve as a potential therapeutic target.^6^

CNM-Au8 is an orally administered suspension of catalytically-active, clean-surfaced gold nanocrystals that provide energetic support to central nervous system (CNS) cells via mitochondrial complex I-like catalytic activity, and exert antioxidant effects via superoxide dismutase- and catalase-like activities.^8^

The potential of CNM-Au8 to treat bioenergetic deficits associated with neurodegenerative disease progression was investigated in several disease models.^8^^,^^9^ In addition to demonstrating CNM-Au8 remyelinating activity, this work demonstrated that CNM-Au8 treatment led to dose-dependent increases in motor neuron survival, preservation of motor neuron neurite network, and concomitant decreases in mislocalised TDP-43 protein aggregates in primary rodent spinal neuron-glial co-cultures challenged with excitotoxic levels of glutamate. In addition, CNM-Au8 treatment dose-dependently protected motor neurons from toxic effects of beta-amyloid 1–42 oligomers, demonstrating that CNM-Au8 counteracts different apoptotic signals downstream of the initial insult from glutamate or amyloid beta stressors. Additional work using primary hippocampal and cortical neurons further demonstrated that neuroprotection by CNM-Au8 was independent of neuronal subtype. Statistically significant efficacy results from these preclinical pharmacology studies together with no adverse effect level (NOAEL) findings up to the highest feasible doses of CNM-Au8 in animal nonclinical toxicology studies conducted per ICH M3 (R2) guidelines supported the advancement of CNM-Au8 into clinical trials.

A Phase 1 First-In-Human study of CNM-Au8 was conducted in 40 and 46 individuals randomised to the single ascending dose (SAD) and multiple ascending (MAD) phases, respectively.^10^ Both the SAD and MAD phases investigated oral dosing of CNM-Au8 15 mg, 30 mg, 60 mg, 90 mg, and placebo. CNM-Au8 was well-tolerated over the course of the study. Routine clinical laboratory assessments (haematology, serum chemistry, and urinalysis), vital signs, ECGs, and physical examinations did not reveal clinically notable findings. There were no serious adverse events (SAEs) or TEAEs leading to discontinuation of treatment. No safety trends or safety signals were observed.^10^

CNM-Au8 is blood–brain barrier penetrant, albeit with relatively low bioavailability in brain tissue (1%–10% in nonclinical animal toxicology studies). Using inductively coupled mass spectroscopy to detect gold levels in blood, it was demonstrated that CNM-Au8 is absorbed slowly, with levels not consistently quantifiable in human whole blood samples until 3–4 weeks of continuous daily oral dosing at 30 mg.^10^ Tissue levels do not reach equilibrium until >6 months of daily oral dosing in nonclinical animal models.

Motor unit number index (MUNIX) is a sensitive neurophysiological biomarker of lower motor neuron loss, which progresses more rapidly than, and is predictive of, clinical decline.^11^^,^^12^ We investigated the effects of CNM-Au8 through 36 weeks of blinded treatment utilizing MUNIX as the primary outcome in this focused proof-of-concept clinical trial. Following the double-blind portion of the trial, participants were offered participation in an open label extension. We report results from both the double-blind and open-label treatment periods as well as survival analyses from all participants.

## Methods

### Trial design and oversight

RESCUE-ALS^13^ was a Phase 2, randomised, parallel group, double-blind, placebo-controlled trial to investigate the efficacy and safety of CNM-Au8 to slow ALS disease progression. RESCUE-ALS and its long-term open label extension (OLE) were conducted at two multidisciplinary ALS clinics located in Sydney, Australia: (i) the Brain and Mind Centre and (ii) Westmead Hospital. The double-blind portion of RESCUE-ALS was conducted from January 16, 2020 (baseline visit, first-patient first-visit (FPFV)) through July 13, 2021 (double-blind period, last-patient last-visit (LPLV)). Survival status for all study participants, irrespective of enrolment into the OLE, were collected through a minimum of the 12-month cut-off following LPLV from the double-blind period through July 14, 2022. The open-label extension remains ongoing and will continue indefinitely so long as participants remain alive. The trial was conducted in accordance with Good Clinical Practice guidelines of the International Conference on Harmonisation and the ethical principles of the Declaration of Helsinki. Protocol approval was granted by the Human Research Ethics Committee of the 10.13039/100014467Western Sydney Local Health District (Reference: 2019/ETH12107). All participants provided written informed consent before screening, and again at entry into the open-label extension. RESCUE-ALS and the open label study are registered in clinicaltrials.gov with registration numbers NCT04098406 and NCT05299658, respectively.

Clene Australia Pty Ltd, a subsidiary of Clene Nanomedicine, Inc., provided active drug and matching placebo. The contract research organization Mobius Medical provided on-site trial monitoring, data collection, study oversight, and auditing of all study data. An independent data and safety monitoring board reviewed unblinded safety data at regular intervals during the trial. Statistical analyses were performed by Instat Clinical Research and Clene. Trial reporting follows CONSORT guidelines for randomised trials.^14^

### Trial participants

Adults with a diagnosis of possible, probable, or definite ALS per the Awaji-Shima criteria^15^ who were newly symptomatic within 24 months of screening or within 12 months of diagnosis were eligible to enrol. Additional criteria were: age between 30 and 80 years at diagnosis; forced vital capacity of at least 60% of the predicted^16^; and, if commenced on riluzole, use of a stable dose for at least 30 days prior to screening. History of two or more relatives with ALS or motor neuron disease (i.e., familial ALS), carpal tunnel syndrome, other compressive neuropathies, and polyneuropathy were exclusionary. Following completion of the 36-week randomised double-blind period, all eligible participants were offered to enter the OLE and receive CNM-Au8 30 mg.

### Trial interventions and procedures

#### Randomisation and masking

CNM-Au8 is an aqueous suspension of catalytically-active clean-surfaced gold nanocrystals concentrated to 500 μg/mL in USP-grade deionized water, buffered with 6.5 mM sodium bicarbonate (NaHCO_3_). The nanocrystals consist of gold atoms self-organized into crystals of consistently faceted geometrical structures (hexagonal bi-pyramid, pentagonal bi-pyramid, tetrahedron, decahedron) produced using a proprietary electrocrystallization method (US Patent 9,603,870).^17^ Placebo consisted of buffered USP-grade water, colour-matched with food-grade colorants. There is no flavour difference between active and placebo.

Participants were instructed to consume a 60 mL dose of investigational medicinal product, provided in single-use HDPE bottles, orally or by feeding tube, daily, and at least 30 min prior to food intake.

During the double-blind period, participants were randomised 1:1 to receive CNM-Au8 30 mg or matched placebo. Computerized block randomisation in blocks of four was implemented for each site by an independent statistician (Instat Clinical Research) and provided securely to an unblinded pharmacist at each site who was responsible for investigational product dispensation.

#### Study procedures

Within 6 weeks of the initial screening visit, eligible study participants returned for a baseline visit during which participants were randomised between active and placebo assignments. Following randomisation, subsequent study visits for data collection and safety assessments occurred every six weeks. Intensive clinical visits occurred at weeks 12, 24, and 36 with phone-calls at weeks 3, 6, 18, and 30. A four-week safety follow-up visit occurred following completion of the 36-week double-blind period, early termination from the trial, or upon exit from the OLE.

#### Open-label extension

Following the completion of the 36-week, randomised, double-blind, placebo-controlled portion of the trial, eligible participants were offered the opportunity to enter the OLE and receive CNM-Au8 30 mg. Key entry criteria for the OLE included completion of the double-blind period, lack of treatment compliance issues, and absence of laboratory abnormalities deemed clinically significant. Participants who qualified and elected to participate in the OLE also provided written informed consent to participate in the OLE. Clinical site visits occurred during the OLE every 12 weeks thereafter. OLE visits could be conducted remotely if due to COVID pandemic concerns or travel difficulties due to disease progression.

OLE participants and clinicians were kept blinded to participants’ original randomisation group while in the OLE. All OLE participants were provided with 30 mg daily doses of CNM-Au8 in identical single-use high density polyethylene containers with the same dosing instructions as during the double-blind portion of the trial.

### Objectives and outcomes

The objective of the RESCUE-ALS trial was to assess the efficacy and safety of CNM-Au8 as a disease-modifying treatment for ALS. ALS disease progression was quantitatively assessed by decline in motor unit number index (MUNIX), a surrogate neurophysiological estimate of functional motor unit numbers.^11^^,^^12^^,^^18^, ^19^, ^20^ Prior work demonstrated that MUNIX is a sensitive electrophysiological marker of disease progression in ALS with potential to precede or predict decline in the widely used ALSFRS-R score.^12^ The mean percent change to week 36 of the summated MUNIX score of four spinal cord-innervated limb muscles was selected as the primary outcome. The interrogated muscles were abductor digit minimi (ADM), abductor pollicis brevis (APB), biceps brachii (BB), and tibialis anterior (TA). MUNIX data were included for all muscles, irrespective of the compound muscle action potential (CMAP) amplitude. For muscles without a recordable CMAP response, a zero-value was recorded for all analyses. Limb-onset and bulbar-onset subgroups were evaluated as pre-specified exploratory endpoints. Respiratory function was measured as erect forced vital capacity (FVC). FVC scores were standardized to the percentage of the predicted normal value on the basis of age, sex, and height.^16^ Secondary efficacy outcomes included: (1) Mean total change of the average difference between active treatment and placebo from baseline through week 36 for the summated MUNIX score, and (2) mean % predicted FVC change of the average difference between active treatment and placebo from baseline to week 36.

Safety endpoints included the incidence of treatment-emergent adverse events (TEAEs), drug-related TEAEs, deaths, and serious adverse events (SAEs) leading to discontinuation from the study, as well as the Falls Questionnaire, and the Columbia Suicide Severity Rating Scale (C-SSRS).

Several exploratory clinical outcome measures were prespecified in the protocol to evaluate disease progression including assessment by the revised ALS functional rating scale (ALSFRS-R) score. The ALSFRS-R is a functional assessment consisting of 12 domains incorporating four types of neurological function (bulbar, fine motor, gross motor, and respiratory) scored using an ordinal scale from 0 (total loss of function) to 4 (no loss of function).^21^ We assessed the (i) proportion of participants free from a six-point or greater decline in ALSFRS-R score by week 36 (a threshold defined in a previous clinical trial),^22^ (ii) mean change in ALSFRS-R total score at week 36, and (iii) combined assessment of function and survival (CAFS), which is a joint-rank test that allows for end-of-study assessment of survival and function (ALSFRS-R change), with higher ranks indicating better outcomes.^23^ ALS disease progression was also evaluated as a composite time-to-event analysis, consisting of the first occurrence of death, tracheostomy, need for non-invasive ventilatory respiratory support, or gastrostomy tube placement during the 36-week double-blind period. Patient-reported outcomes included quality of life measured by the ALSSQOL-short form,^24^ and patient's global impression of change (PGI). Time to death, clinician global impression of change (CGI), and change in the slope of delta-FS^25^ were included as additional exploratory clinical outcomes. Exploratory neurophysiology outcomes included MUNIX percent change by unique muscle (ADM, APB, BB, and TA), Split Hand Index,^26^ Neurophysiological Index,^27^ MUSIX^28^ percent change, MScanFit MUNE,^29^ and summated MUNIX responder analyses with decline thresholds of ≥ −15% and ≥−25%. As per protocol, the participant genetic information was not assayed.

### Statistical analysis

The primary outcome was the difference in least squares mean percent change from baseline to week 36 of the summated MUNIX score for the ADM, APB, BB, and TA, where each participant's baseline MUNIX value was indexed to 100%, and changes were calculated as the percent change from baseline in the intent-to-treat (ITT) population. The ITT population included all screened participants who were randomised.

Based on an observational longitudinal study of MUNIX score decline in ALS patients,^12^ a 38.4% reduction in the summated MUNIX score at 36 weeks with a standard deviation of 19.8% was assumed for the study population. At a 1:1 (CNM-Au8 30 mg: placebo) allocation, an estimated 36 participants would be required to detect a treatment effect of 19.2% at week 36 (a 50% relative slowing of decline compared to the estimated rate of placebo decline of 38.4%) with 80% power and a two-sided alpha level of 0.05. Assuming a 12.5% estimated non-evaluable rate, a target enrolment of 42 patients (21 active:21 placebo) was planned for assignment to randomised treatment.

A mixed model for repeated measures (MMRM) was used for the primary and secondary endpoints; model terms included treatment, visit, treatment by visit interaction as fixed effects, and baseline risk score of the ENCALS survival prediction model^30^^,^^31^ as covariates with an unstructured covariance matrix. The ENCALS risk score is a validated model of ALS disease progression and incorporates baseline risk characteristics from each participant that have been identified to be predictive of survival, including bulbar vs. non-bulbar onset, age at onset, definite versus probably or possible ALS diagnosis, time from symptom onset, FVC, and ALSFRS-R progression rate.^28^ Exploratory endpoints were analysed by a chi-square test for the difference in proportions, or by a mixed model for repeated measures with baseline value and ENCALS risk score as covariates. The ALSFRS-R change was analysed using a mixed model with treatment, baseline ALSFRS-R score, time (months from first symptom onset), and ENCALS score as factors, including interaction terms for treatment by time and treatment by baseline, with time treated as a random effect. Due to the small size of this proof-of-concept study, no statistical hierarchy was proposed beyond the primary and secondary efficacy endpoints. No hierarchy was proposed for exploratory endpoints, and no adjustments were made for multiple comparisons. Accordingly, p-values are not reported for secondary and exploratory endpoints for the double-blind period. Treatment effects for exploratory endpoints are reported as the least square mean difference and 95% confidence interval, or the absolute risk reduction and 95% confidence intervals for differences in proportions or differences in event-rates, as applicable. Site level stratification was not incorporated in the statistical analysis plan.

Safety endpoints included the incidence of treatment-emergent adverse events (TEAEs), drug-related adverse events (Related TEAEs), deaths, and TEAEs assessed as serious (SAEs), TEAEs leading to discontinuation from the study, as well as Falls Questionnaire, and the Columbia Suicide Severity Rating Scale (C-SSRS). Changes from baseline in clinical laboratory results and vital signs were summarized by treatment group and time point. TEAEs were coded using the Medical Dictionary for Regulatory Activities (MedDRA v.21) and were tabulated by System Organ Class (SOC) and Preferred Term (PT).

For the long-term survival analysis, all-cause mortality of participants who were originally randomised to active were compared against participants originally randomised to placebo, from baseline through the data-cut of 14-July-2022, representing at least 12 months following the LPLV. Survival status or lost-to-follow-up status was reported by investigators for all study participants irrespective of whether they continued into the OLE. The 12-month follow-up period was selected as a cut-off for these analyses, as the protocol was amended to continue indefinitely based on observed survival. A hierarchy for analyses of endpoints following the completion of the double-blind period was not prespecified. Future analyses for subsequent 12-month intervals will be reported as they mature. Data were right censored for participants lost to follow-up or alive at the day of last study contact. Survival status was confirmed by investigators. Treatment groups were compared using a log-rank test comparing time to death conducted at a 2-sided alpha level of 0.05. *Post hoc* sensitivity analyses included imputation of death for participants lost-to-follow-up. Hazard ratios (HR) are presented along with 95% Wald confidence intervals and Kaplan–Meier curves by treatment group.

OLE data are reported as observed values including means and standard deviations for ALSFRS-R, FVC, and ALSSQOL-SF change including all available data as of the 14-July-2022 data cut-off. Due to the variability in OLE visit attendance due to COVID risk, or missing visits due to disease progression, we used random slopes models *post hoc* to investigate whether original randomisation (e.g., early versus delayed treatment) affected the rate of disease progression following the double-blind period. These models used all available data to test the difference in slope (i) from randomisation until 12-weeks after the crossover into the OLE (week 48 post-baseline), and (ii) difference of slope for all OLE participants from 24-weeks into the OLE, week 60 to week 120 from randomisation. Since there is low bioavailability and slow uptake of CNM-Au8, we reasoned the first 12-weeks of active treatment represented a subtherapeutic period in which to extend the comparison from the initial baseline. Similarly, we evaluated original active versus placebo randomisation groups during the OLE starting at OLE week 24 (week 60 post-randomisation), so as not to bias the slope estimates for original placebo randomised participants with declines that may have occurred during the subtherapeutic period from entry into the OLE. Random slopes models evaluated the difference in the rate of change for the ALSFRS-R, FVC, and ALSSQOL-SF. The random slope models incorporated covariates including delta-FS, time from symptom onset, treatment assignment, and time (days). Backward selection was used to minimise AICc,^32^ the finite-sample corrected version of the Akaike information criterion to determine the final model. Time to event analyses, including ALS disease progression events and death or tracheostomy during the OLE, are reported as the proportion event free of disease progression events along with the 95% confidence intervals.

Statistical analyses were performed using SAS 9.4. Figures were generated in Prism 9.4.1.

### Role of the funding source

RESCUE-ALS was substantially funded by a grant from 10.13039/100014012FightMND (Australia). Additional funding was provided by Clene Australia Pty. Ltd. The sponsor was involved in study design, data analysis, and interpretation of data, as well as writing of the report. These activities were undertaken in consultation with the study clinicians who had final say in data interpretation and writing of manuscript as well as submission. FightMND had no role in collection of data, interpretation of results, analysis or writing of manuscript. Every author had access to the full dataset if they had wanted and there were no restrictions to access. SV and MCK had final responsibility for the decision to submit for publication.

## Results

Between December 19, 2019, and November 2, 2020, 49 participants with ALS were screened for eligibility, of whom 45 were randomly allocated to CNM-Au8 (n = 23) or matching placebo (n = 22). Baseline demographics are summarized in Table 1 and presented graphically in Supplemental Fig. S1. Thirty-three (73%) of enrolled participants had limb-onset ALS; 12 (27%) had bulbar-onset ALS. Forty-one (91%) participants were on stable riluzole treatment at baseline and one participant (2%) received edaravone as background standard-of-care. Since edaravone was not approved for marketing authorisation in Australia, its use was not anticipated and therefore not explicitly excluded per the study protocol. Overall, treatment groups were well-matched with respect to baseline characteristics (Table 1).

**Table 1:** Demographic and clinical characteristics of the intent-to-treat population at baseline.

Category Number (%) or Mean (SD)	CNM-Au8 30 mg	Placebo	Overall
Male Sex^a^ – n (%)	13 (57%)	13 (59%)	26 (58%)
Female Sex^a^ – n (%)	10 (43%)	9 (41%)	19 (42%)
White Race – n (%)	19 (83%)	16 (73%)	35 (78%)
Other Race – n (%)	4 (17%)	6 (27%)	10 (22%)
Limb Onset – n (%)	16 (70%)	17 (77%)	33 (73%)
Bulbar Onset – n (%)	7 (30%)	5 (23%)	12 (27%)
Riluzole Use – n (%)	22 (96%)	19 (86%)	41 (91%)
Edaravone Use – n (%)	1 (4%)	–	1 (2%)
Awaji-Shima Criteria			
Definite – n (%)	11 (48%)	12 (55%)	23 (51%)
Probable – n (%)	10 (44%)	7 (32%)	17 (38%)
Possible – n (%)	2 (9%)	3 (14%)	5 (11%)
Age	57.0 (13.3)	61.3 (10.9)	59.1 (12.3)
Delta-FS^b^	0.75 (0.55)	0.81 (0.73)	0.78 (0.63)
ENCALS risk score^c^	−4.6 (1.7)	−4.2 (1.8)	−4.4 (1.8)
BMI (mg/k^2^)	26.5 (4.9)	24.6 (3.9)	25.5 (4.5)
MUNIX Sum^d^	380 (198)	376 (153)	378 (175)
FVC (% of predicted of normal)^e^	84.5 (18.3)	78.2 (14.5)	81.5 (16.7)
ALSFRS-R^f^ Total Score	38.6 (6.6)	38.8 (5.4)	38.7 (6.0)
Bulbar score	10.1 (1.9)	10.1 (2.3)	10.1 (2.1)
Gross motor score	8.6 (3.0)	9.0 (2.7)	8.8 (2.8)
Fine motor score	8.8 (3.3)	8.3 (2.7)	8.6 (3.0)
Respiratory score	11.1 (1.4)	11.4 (1.0)	11.2 (1.2)
Month since onset	15.5 (7.6)	16.1 (10.9)	15.8 (9.3)
Months since diagnosis	3.0 (2.9)	3.3 (3.2)	3.1 (3.0)

a Participants were requested to self-report “sex at birth” during the screening visit.

b Delta-FS is the pre-baseline ALSFRS-R slope defined as the maximum ALSFRS-R score minus the baseline total score divided by the number of months from onset of symptomatic weakness.

c ENCALS risk score is the European Network for Curing ALS individual participant risk score.

d MUNIX Sum is the neurophysiologic derived Motor Unit Index summed across the abductor digit minimi (ADM), abductor pollicis brevis (ABP), biceps brachii (BB), and tibialis anterior (TA).

e FVC is forced vital capacity.

f ALSFRS-R, the ALS Functional Rating Scale Revised, measures 12 items in four domains of function, each scored on a scale from 0 to 4, with higher scores indicating better function.

**Fig. 1:**
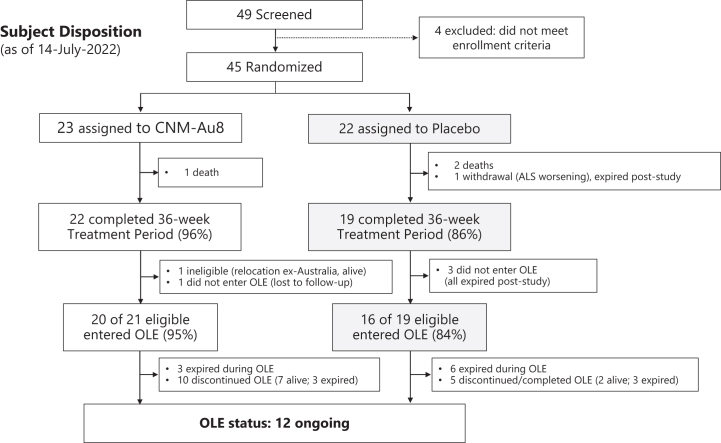
**Screening, randomisation, and follow-up of study participants**. Screening, randomisation, and participant vital status through at least 12 months of follow-up from the LPLV of the double-blind period). OLE: open-label extension; LPLV: last patient last visit.

Twenty-two (96%) participants allocated to CNM-Au8 and 19 (86%) to placebo group completed the 36-week double-blind treatment period (Fig. 1). Thirty-six (36) participants (90% of those eligible) entered the OLE at the end of the 36-week double-blind treatment trial, comprising twenty participants originally randomised to active and sixteen participants originally randomised to placebo.

### Primary, secondary, and exploratory outcomes

#### Primary outcome

**Fig. 2:**
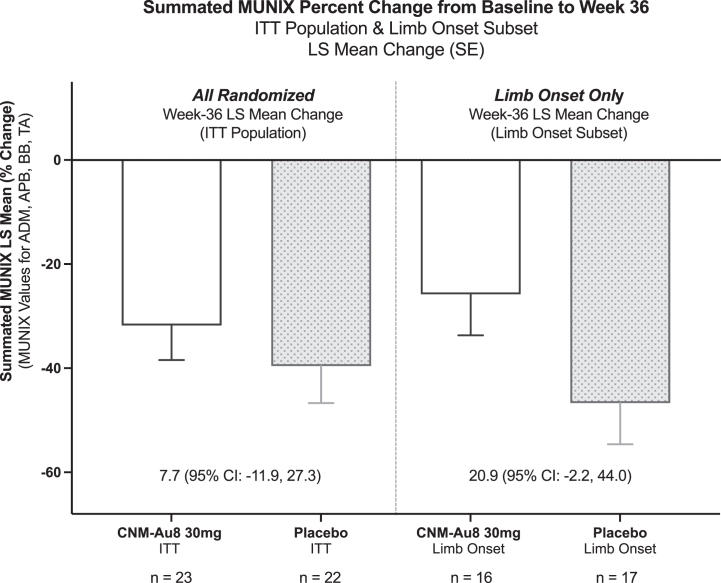
**Primary outcome in the ITT and limb onset subgroup**. Summated MUNIX Results for intention-to-treat (ITT) Population and Limb Onset subgroup. Summated MUNIX percent change for the abductor digit minimi (ADM), abductor pollicis brevis (ABP), biceps brachii (BB), and tibialis anterior (TA), where each participant's baseline summated MUNIX value was indexed to 100%. ITT: Intent to treat; LS Mean: least squares mean; SE: standard error. CI: 95% confidence interval.

The primary, secondary, and exploratory outcome measures related to MUNIX and clinical function are summarized in Table 2, Table 3. There was no significant effect of CNM-Au8 on the primary endpoint in the ITT population (least-squares [LS] mean change: −31.8% CNM-Au8, –39.6% placebo; LS mean difference: 7.7% (95% CI: −11.9% to 27.3%; p = 0.43, Fig. 2). Analysis of the pre-specified limb-onset and bulbar-onset subgroups revealed that the bulbar onset subgroup exhibited an unexpected lack of decline in the summated MUNIX scores (−7.9% bulbar placebo LS mean change at week 36 versus −38.4% *a priori* statistical assumption, Supplemental Fig. S2), while the decline in limb onset placebo participants exhibited a pronounced decline in summated MUNIX score at week 36 (−46.7% limb-onset placebo LS mean change, Supplemental Fig. S2). CNM-Au8 treated limb-onset participants exhibited a slower decline in summated MUNIX score compared to placebo treated participants at week 36 (LS mean difference 20.9%; 95% CI: 2.2%–44.0%, p = 0.074, Fig. 2). Nonetheless, a *post hoc* test of interaction (ALS onset site x Treatment) resulted in a p-value of 0.0783, indicating there was insufficient evidence to conclude there was a difference in MUNIX treatment response between limb and bulbar onset subgroups. The time course of MUNIX change by 12-week clinic visit is presented in Supplemental Fig. S3.

**Table 2:** Primary, secondary, and exploratory clinical outcomes in the ITT Population, evaluated by least squares mean change from BL to study week 36.

Primary, secondary, and exploratory Clinical Outcomes		Least-squares mean change (SE) at week 36		LS mean difference vs. placebo (95% CI) at week 36
		CNM-Au8 30 mg	Placebo	
1st	MUNIX^a^ Sum Percent	−31.8 (6.6)	−39.6 (7.1)	7.7 (−11.9, 27.3)
2nd	MUNIX Sum	−123 (25)	−141 (27)	19 (−56, 94)
	FVC (% predicted)	−16.7 (5.4)	−20.3 (5.8)	3.6 (−12.4, 19.7)
Exploratory	ALS Specific QoL^b^	−0.3 (0.2)	−1.2 (0.3)	0.9 (0.2, 1.6)
	CAFS Summated Score (Joint-Rank)^c^	4.4 (5.1)	−4.6 (5.2)	9.1 (−5.8, 23.9)
	Clinician Global Impression of Change	−1.4 (0.2)	−1.2 (0.2)	−0.2 (−0.7, 0.3)
	Patient Global Impression of Change	−1.2 (0.2)	−1.1 (0.3)	−0.1 (−0.8, 0.6)
	Delta-FS Change	0.07 (0.1)	0.06 (0.1)	0.01 (−0.18, 0.2)
	ALSFRS-R Total Score	−4.8 (0.8)	−5.8 (0.9)	1.0 (−1.6, 3.6)
	Bulbar Domain	−0.9 (0.4)	−1.6 (0.4)	0.7 (−0.4, 1.8)
	Respiratory Domain	−0.7 (0.3)	−0.8 (0.3)	0.0 (−0.8, 0.9)
	Gross Motor	−1.6 (0.4)	−1.7 (0.4)	0.1 (−1.0, 1.3)
	Fine Motor Domain	−1.8 (0.4)	−1.5 (0.4)	−0.3 (−1.5, 1.0)

The specified statistical model for the primary and secondary outcomes was an unstructured covariance model based on a mixed model for repeated measures with treatment, visit, treatment by visit interaction as fixed effects, and with baseline value and baseline risk score of the ENCALS survival prediction model as covariates.

ALSFRS-R change (and subdomains) was analysed using a mixed model with treatment, baseline ALSFRS-R score/subscale score, time (months from first symptom onset), and ENCALS score as factors, including interaction terms for treatment by time and treatment by baseline. Time was treated as a random effect and an unstructured covariance structure was assumed.

a Summated MUNIX change for the abductor digit minimi, abductor pollicis brevis, biceps brachii, and tibialis anterior, where each participant's baseline MUNIX value was indexed to 100%.

b ALS Specific Quality of Life-Short Form.

c Combined assessment of survival and ALSFRS-R total score; a joint-rank comparison of the mean summated score by treatment group.

**Table 3:** Exploratory clinical outcomes in the ITT population evaluated by time to event or proportion event free.

Exploratory time to event clinical outcomes	Percent event free (95% CI) at week 36		Absolute risk reduction vs. placebo
	CNM-Au8 30 mg	Placebo	
Mortality (% Event Free)	96% (87%–100%)	91% (79%–100%)	5%
ALS Clinical Worsening (% Event Free)^a^	78% (61%–95%)	23% (0%–55%)	55%
Proportion Free from ≥ 6-point ALSFRS-R Decline	48%	18%	30%

The time to ALS clinical worsening was assessed using the Kaplan–Meier method. Time to event between treatments was analysed using a log-rank test. The Chi–Square test was used to analyse treatment differences in proportions free from ALSFRS-R decline.

a Time to first occurrence (event) of death, tracheostomy, need for non-invasive ventilatory support, or gastrostomy tube placement using Kaplan–Meier analysis.

#### Secondary outcomes

The secondary outcomes, difference in the overall summated MUNIX score and FVC (% predicted) at week 36 did not differ between treatment groups. The LS mean difference for the MUNIX score was 18.8 (95% CI: −56.4 to 94.0). The LS mean difference for % predicted FVC change was 3.6 (95% CI: −12.4 to 19.7). *Post hoc* sensitivity analyses incorporating additional covariates such as pre-treatment baseline ALSFRS-R slope (delta-FS), age, sex, site of symptom onset, use of riluzole, and clinic recruiting site, in addition to the prespecified covariates did not change interpretation of the primary and secondary outcomes (Supplemental Table S1).

#### Exploratory outcomes

**Fig. 3:**
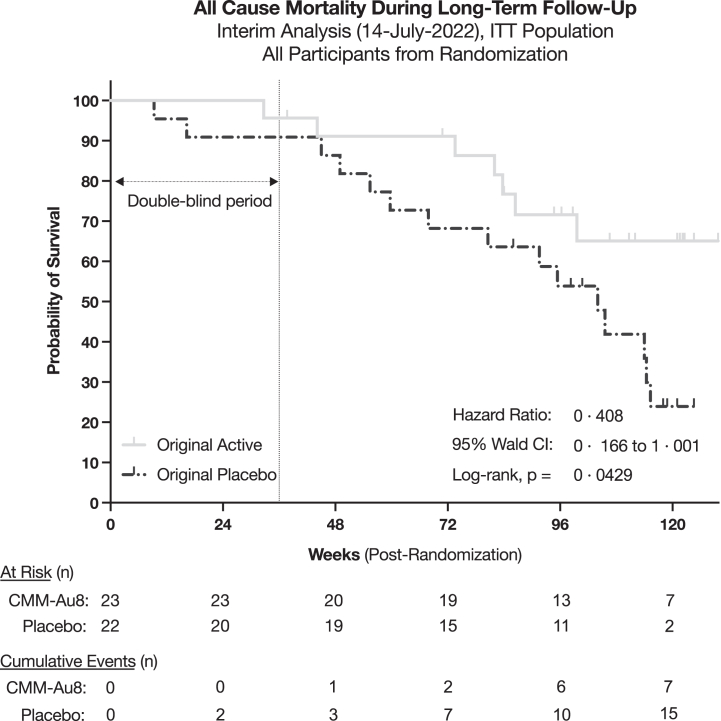
**Time to all-cause mortality through at least 12 months following LPLV**. Time to all-cause mortality amongst participants originally randomised to CNM-Au8 compared to participants originally randomised to placebo through at least 12 months following the last patient last visit (14-July-2022). Vital status and date of death (as applicable) were captured for all participants withdrawn from the study. Lost-to-follow-up were censored as of the date of last study contact. All OLE ex-placebo CNM-Au8 transitioned participants are assessed within the placebo group. All current OLE participants are right censored as of 14-July-2022.

Exploratory outcomes were assessed as hypothesis-generating for potential future clinical investigation (Table 2, Table 3). To measure the rate of ALS disease progression, a pre-specified time to event analysis was performed based on the first occurrence of (i) death, (ii) tracheostomy, (iii) need for non-invasive ventilatory support, or (iv) gastrostomy (feeding) tube placement. Kaplan–Meier analyses showed a 55% absolute risk reduction in CNM-Au8 treated group compared to placebo through week 36; percent event free: active 78% (95% CI: 61%–95%) vs. placebo 23% (95% CI: 0%–55%), Fig. 3. The distribution of ALS disease progression events by treatment group is shown in Supplemental Table S2. The proportion of ALS patients with a ≥ 6-point decline in the ALSFRS-R total score at week 36 was reduced in the CNM-Au8 treated group versus placebo (48% vs. 18%, 30% absolute risk reduction, Supplemental Fig. S4A). Slowing of quality-of-life decline, assessed by the ALSSQOL-SF,^24^ was also observed in the CNM-Au8 treated participants (LS mean difference: 0.9; 95% CI: 0.2 to 1.6; Supplemental Fig. S4B). Time-to-death analyses showed fewer mortality events with CNM-Au8 treatment through the double-blind period through week 36 (active: 4%, 95% CI: 0–13% vs. placebo: 9%, 95% CI: 0–21%). This difference in mortality favouring CNM-Au8 became more pronounced as additional mortality events occurred with continued follow-up through the reporting period of 12-months post LPLV (described below). Differences observed with respect to summated CAFS score, mean rate of ALSFRS-R slope decline, change in the pre-baseline delta-FS slope, clinician global impression of change, and patient global impression of change had wide and overlapping confidence intervals due to the limited sample size (Table 2).

Exploratory neurophysiology endpoints are described in Supplemental Table S3. Week 36 MUNIX measurements for each interrogated muscle directionally favoured CNM-Au8 (LS mean difference of the percent change from baseline (95% CI), for the TA: 23.7% (−24.7% to 72.1%), ADM: 17.5% (−38.1% to 73.1%), and APB: 7.7% (−26.4% to 41.9%), except for the BB, which directionally favoured placebo, BB: −2.2% (−28.1% to 23.7%). However, the confidence intervals are wide and do not support inference of a treatment effect.

### Long term survival from randomisation

A survival benefit was observed in participants initially randomised to CNM-Au8 treatment, with an approximate 60% reduction in all-cause mortality over at least 12 months of follow-up from the LPLV (HR = 0.408, 95% Wald CI: 0.166 to 1.001, log-rank p = 0.0429, Fig. 3), and was statistically significant. There were 7 deaths in participants originally randomised to CNM-Au8 and 15 deaths in participants originally randomised to placebo. Median survival for the original placebo group was 104 weeks (95% CI: 59.7 to 115.3 weeks), while median survival from randomisation for the active group was undefined due to insufficient events through the follow-up period (*indeterminate*, 95% CI: 83.7 to undefined). Survival status as of the data cut-off was available for 41 of 45 participants from the ITT population; the four participants lost to follow-up included three active and one placebo participant. A *post hoc* sensitivity analyses substituting death in place of censoring for the four participants lost to follow-up was not significant (HR = 0.545, 95% Wald CI: 0.247 to 1.201, log-rank p = 0.126) at the 0.05 alpha level.

### Open label extension

This initial report of the OLE treatment period encompassed the 12-month period from the double-blind LPLV through 14-January-2022. Twenty of 22 participants originally randomised to CNM-Au8, entered the OLE (one individual was ineligible based on emigrating from Australia; the other declined participation and was lost to follow-up). Sixteen of 19 participants originally randomised to placebo, who competed the double-blind treatment period, entered the OLE (the three non-OLE enrolling placebo participants all expired within 9–32 weeks following study exit). Since participation in the OLE was optional, caution should be used regarding interpretation of OLE results, as continuation into the OLE was not random. In addition, participant visits during the OLE were conducted remotely in many cases either due to concern regarding COVID risk or if related to disease progression, so there was potentially additional variability in reported data over time for neurophysiology assays, ALSFRS-R, FVC, and ALSSQOL during the OLE when contrasted with the double-blind treatment period. Neurophysiology was not collected consistently during the OLE (6–8 assessments per group per visit), so did not provide meaningful data for evaluation. Observed group mean values, range, and standard deviations for ALSFRS-R, FVC, and ALSSQOL assessments conducted during the OLE period assessments are reported in Supplemental Table S4. As reported in Supplemental Table S4, the observed values for ALSFRS-R, FVC, and ALSSQOL by visit seemingly demonstrated overall stability in these measures. However, these estimates are likely inflated due to intermittently missed clinical visits. To account for this, we evaluated change during the OLE period utilizing a random slopes models *post hoc*, which more robustly incorporates change in the rate of decline, especially with respect to missing information. Random slopes models for ALSFRS-R, FVC, and ALSSQOL compared originally randomised active versus originally randomised placebo participants showed significant differences through 48-weeks following randomisation in favour of CNM-Au8 for slowed decline for ALSFRS-R change (ALSFRS-R difference (95% CI): 0.364 (0.070 to 0.659) points/month; 2.6 point difference); and ALSSQOL change (ALSSQOL difference (95% CI): 0.127 (0.057 to 0.196) points/month (Table 4, Supplemental Fig. S6). Similarly, when offsetting the start of the random slopes model by 24 weeks into the OLE to account for the slow absorption and uptake of CNM-Au8, significant differences for ALSFRS-R decline were observed from study week 60 to week 120 (ALSFRS-R difference (95% CI): 0.397 (0.119 to 0.674) points/month; 6.0 point difference) (Table 4). In this 24 week random slopes offset model, the substantial difference in ALSFRS-R decline of 6 points, may suggest participants originally randomised to placebo had much worse decline overall and could not overcome the initial decline of ALSFR-R loss experienced during the double-blind period compared to participants originally randomised to active treatment.

**Table 4:** Random slopes model results for ALSFRS-R, ALSSQOL-SF, FVC in the OLE period.

Slope change Points/Month	Randomisation to Week 48 (12-weeks post OLE Baseline)	24 Weeks post OLE baseline (Week 60 to Week 120)
**ALSFRS-R Random Slopes Model**		
Original Active	−0.796	−0.447
Original Placebo	−1.166	−0.843
Slope Difference (SE)	0.364 (0.149)	0.397 (0.139)
95% CI of Slope Difference	0.070 to 0.659	0.119 to 0.674
Period Ending Difference (Active Less Placebo)	2.6	6.0
p-value	0.0159	0.0057
**FVC (% predicted) Random Slopes Model**		
Original Active	−2.05	−2.2
Original Placebo	−2.05	−2.2
Slope Difference (SE)	0	0
95% CI of Slope Difference	NA	NA
Period Ending Difference (Active Less Placebo)	6.9	5.3
p-value	NS	NS
**ALSSQOL-SF Random Slopes Model**		
Original Active	−0.010	0.124
Original Placebo	−0.136	−0.006
Slope Difference (SE)	0.127 (0.035)	0.119 (0.037)
95% CI of Slope Difference	0.057 to 0.196	0.045 to 0.192
Period Ending Difference (Active Less Placebo)	1.0	2.9
p-value	0.0004	0.0018

The random slope models incorporated covariates including delta-FS, time from symptom onset, treatment assignment, and time (days). Backward selection was used to minimise the Akaike information criterion (AIC) to determine final model terms.

**Fig. 4:**
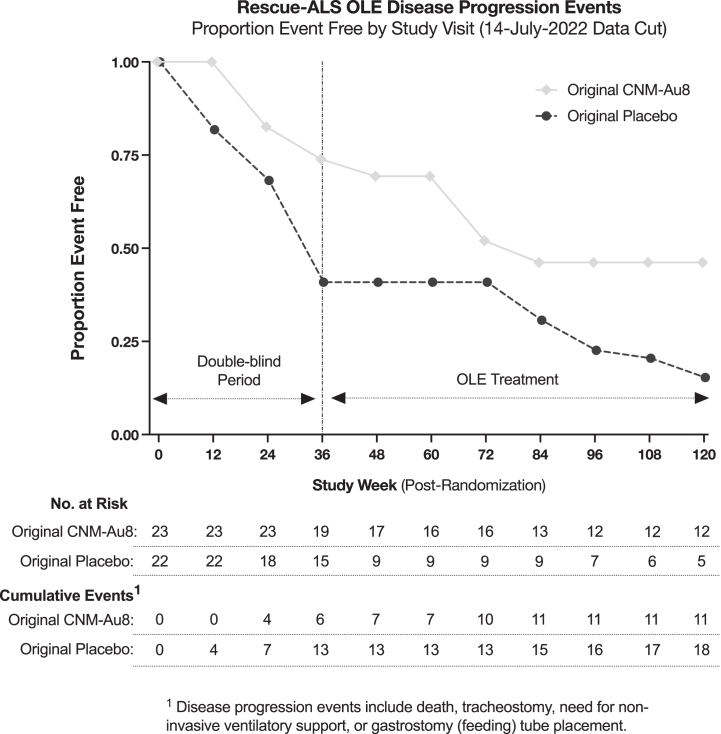
**Time to ALS disease progression during the double-blind and OLE treatment periods**. Time to ALS disease progression defined as the first occurrence of death, tracheostomy, need for non-invasive ventilatory support, or gastrostomy tube placement amongst originally randomised to CNM-Au8 compared to participants originally randomised to placebo through at least 12 months following the last patient last visit (14-July-2022). All OLE ex-placebo CNM-Au8 transitioned participants are assessed within the placebo group. All current OLE participants are right censored as of 14-July-2022.

As observed in the double-blind period, during the OLE period time to disease progression events (death, tracheostomy, need for non-invasive ventilatory support, or gastrostomy tube placement) continued to favour originally randomised CNM-Au8 treated participants through up to 120 weeks (Fig. 4). The proportion of originally randomised CNM-Au8 participants who did not experience a disease progression event at week 120 was 46.2% (95% CI: 23.8%–68.6%), which is contrasted with originally randomised placebo treated participants of 15.3% (95% CI: 0%–31.1%), log-rank p = 0.049. Double-blind participants who did not enrol into the OLE were not included past their respective safety follow-up visits, so the reported event rates during the OLE period may be underestimated. For instance, the three ex-placebo participants who did not enrol in the OLE all expired within 9–32-weeks of their week 36 visit. Time to death or tracheostomy amongst participants in the OLE favoured originally randomised CNM-Au8 participants through week 120 (Supplemental Fig. S7), where the proportion free of an event through week 120 was 79% (95% CI: 60.2%–97.7%) and 45% (95% CI: 17.2%–72.4%) for participants originally randomised to placebo. Time to King's clinical stage progression and MiToS progression, assessed by ALSFRS-R change by visit, did not differ significantly between originally randomised CNM-Au8 and originally randomised participants (data not shown).

### Safety

CNM-Au8 was well tolerated. TEAEs were predominantly assessed as transient an of mild-to-moderate in severity. Overall, TEAEs were balanced between active- and placebo-treated participants (Table 5). Related TEAEs occurred in 3 (13%) active and 2 (9%) placebo participants (Table 5). There were no differences observed in TEAE occurrence by system organ classification. The most common TEAEs associated with CNM-Au8 were aspiration pneumonia, nausea, contusion, and abdominal discomfort. The occurrence of SAEs was similarly balanced between active- and placebo-treated participants (Table 5). The most common SAE associated with the active treatment group was aspiration pneumonia. No SAEs were assessed as related to active treatment by the study investigators. In the active treatment group, there was one death and no participant withdrawals. In the placebo treated group, two deaths and one withdrawal due to disease progression were reported. Safety laboratory parameters (haematology, serum chemistry, or urinalysis) did not reveal any consistent clinically significant findings. Two active participants had clinically significant laboratory abnormalities at the week 24 visit (one with abnormally high haematology findings; one with abnormally high serum chemistry findings). These findings returned to normal or were assessed as not clinically significant by the next study visit at week 36. One placebo-treated participant had consistently elevated creatinine kinase at all study visits. No TEAEs were reported associated with clinical laboratory parameters, vital signs, or electrocardiograms. No clinically significant changes in vital signs were noted. Falls were reported in 14 (61%) participants in the active group and in 14 (64%) participants in the placebo group. No participants reported any form of suicidal behaviour or ideation during the study.

**Table 5:** Treatment emergent adverse event and serious adverse event summary during the double-blind period.

	CNM-Au8 30 mg (N = 23)	Placebo (N = 22)
**Participants with at more than one TEAE; n (%)**	17 (73.9)	17 (77.3)
**TEAEs reported in more than one participant in either group; Preferred term; n (%)**		
Fall	2 (8.7)	3 (13.6)
Pneumonia aspiration	3 (13.0)	0
Laceration	0	3 (13.6)
Contusion	2 (8.7)	0
Nausea	2 (8.7)	0
**TEAE Severity Distribution; n (%)**		
Mild	14 (60.9)	13 (59.1)
Moderate	7 (30.4)	5 (2.7)
Severe	3 (13.0)	6 (27.3)
**TEAEs considered related to study drug; Preferred term; n (%)**		
Nausea	2 (8.7)	0
Abnormal faeces	1 (4.3)	0
Diarrhea	0	1 (4.5)
Rash erythematous	0	1 (4.5)
**Serious Adverse Events (SAEs); Preferred term; n (%)**		
Participants with more than one event	5 (21.7)	5 (22.7)
Pneumonia aspiration	3 (13.0)	0

TEAE = treatment-emergent adverse event. SAE = Serious adverse event.

During the OLE phase, safety laboratory assessments including haematology, serum chemistry, and urinalysis did not reveal changes that were either clinically significant or consistently out of normal ranges. Two TEAEs, lethargy and rash on right heel, were identified as possibly related to treatment. Five participants who were originally randomised to CNM-Au8 experienced SAEs, during the OLE. Ten participants who were originally randomised to placebo reported SAEs during OLE follow-up. None of the participants experienced the same SAE (n = 1 for all events). No SAEs were assessed related to treatment by the study investigators. No new safety findings were observed during long-term follow-up.

## Discussion

The RESCUE-ALS clinical trial was a novel, phase 2, proof-of-concept study that investigated the safety and efficacy of CNM-Au8, a nanotherapeutic drug targeting CNS energetic dysmetabolism that supports cellular energy production and utilization. Historically, survival and functional decline have been regarded as optimal efficacy parameters by the FDA and EMA.^33^ In order to attain adequate statistical power, clinical trials utilizing survival and functional decline as primary outcome measures require large sample sizes and longer-term follow-up.^3^ An objective of RESCUE-ALS was to design an efficient and cost-effective trial by selecting a sensitive endpoint that would require fewer participants and less resource utilization, while minimizing inter-site variability. To our knowledge, this is the first ALS clinical trial to utilise a neurophysiological biomarker as the primary outcome, advancing the clinical framework for assessment of ALS treatments. Results from such focused studies can inform subsequent decisions with respect to conducting large phase 3 studies powered for clinical endpoints.

In multicentre observational studies of individuals with ALS, MUNIX has demonstrated an acceptable test–retest coefficient of variation,^11^^,^^34^ and showed greater relative decline than ALSFRS-R scores longitudinally.^12^^,^^18^ Previous work demonstrated that MUNIX provides a sensitive estimate of the number of functioning motor units, representing a biomarker for lower motor neuron decline. MUNIX does not reflect reinnervation status, although the latter is reflected by MUSIX.^20^ Based on these prior MUNIX studies, RESCUE-ALS trial was aggressively powered to demonstrate a 50% relative reduction in summated MUNIX decline over the 36-week double-blind trial period along with a rate of placebo decline (−38.6%) that was greater than the observed mean change in this trial (−32.5%). Overall, based on the observed decline from baseline and the standard deviation of the decline we observed amongst placebo-treated participants for the primary outcome at week 36, the 50% reduction in MUNIX decline would have required a sample size of approximately 36 participants per group (n = 72) at an alpha of 0.05 and power of 80%, which suggests the study was underpowered based on the original *a priori* assumptions.

In this trial of 45 participants, there were no significant changes between treatment groups in the primary and secondary outcomes analysed on the 36-week double-blind data. The absence of significant differences in MUNIX measures may be explained, at least in part, by inclusion of bulbar-onset ALS participants, where the placebo group did not exhibit an appreciable decline in limb-innervated muscles over 36 weeks. This was contrasted by a 20.9% LS mean difference of summated MUNIX scores in limb-onset patients at week 36 following CNM-Au8 treatment. A possible reason for the discrepancy between decline in MUNIX demonstrated in previous study^12^ compared to this study was that in this study participants were earlier in their disease course.

CNM-Au8, in combination with riluzole, was well-tolerated and provided reassuring preliminary safety findings. TEAEs and SAEs were balanced between active and placebo treated participants. No SAEs were assessed as related to active treatment by the study investigators, and no discontinuations were reported in the active treatment group. The enrolled population was representative of the typical ALS population and inclusion criteria were in keeping with recent clinical trials. The enrolled ALSFRS-R score was consistent with a mild-to-moderate level of disability, representative of an ALS cohort attending an outpatient clinical service.

This study had several limitations including its small sample size, which can make a trial vulnerable to discrepancies in participant disposition between active and placebo groups despite a rigorous randomisation scheme. In this trial there were slight differences between active and placebo treated participants at baseline that were not statistically significant. Placebo participants were treated less frequently with riluzole (86% vs. 96%), had a lower FVC (78% vs. 85%), a higher pre-treatment delta-FS progression rate (0.81 vs. 0.75), and were older (61 vs. 57 years) (Table 1, Supplemental Fig. S1). To address these potential imbalances, the primary statistical model incorporated the ENCALS risk score as a covariate which includes age, site of onset, FVC, ALSFRS-R, and time from symptom onset.^30^ In addition, we analysed baseline delta-FS, sex, and age as covariates in the random slopes models for analyses of disease progression during the OLE period to adjust for potential imbalances. Another limitation was the selection of MUNIX as the primary endpoint, as well as the selection of the four muscles innervated by the spinal cord motor neurons. Neurophysiological techniques require extensive training and expertise, without which excessive variability may occur.^34^ We attempted to mitigate this risk by utilizing trained assessors at two clinical trial sites with extensive electromyography expertise. The selection of the ABP, APM, BB, and TA muscles was driven by prior work showing declines with this index that we used to power this study.^12^ Based on our finding that bulbar-onset individuals early in their disease course do not demonstrate MUNIX decline across these muscles as limb-onset individuals do, other neurophysiology measures may have better assessed differences in limb and bulbar phenotypes, and served as better electrophysiological biomarkers of disease progression in different body regions. Further, we did not incorporate site level stratification effects in our sample size estimates, which may have contributed to the underpowering of this study. Finally, additional covariates to adjust for potential confounding effects may have been appropriate for longer-term and OLE analyses, however, the limited sample size precludes reliable conclusions regarding other covariates.

Because the primary and secondary endpoints of the trial were not reached, it is important to cautiously interpret the exploratory endpoints and OLE outcomes suggesting potential benefit favouring CNM-Au8 treatment, and for the purposes of hypothesis-generation only. The exploratory endpoint results were not adjusted for multiple comparisons and cannot be used to directly infer treatment effects. Amongst exploratory clinical endpoints, CNM-Au8 treatment was associated with several potential efficacy signals, including a significant reduction of time to ALS clinical worsening, including the first occurrence of death, tracheostomy, need for non-invasive ventilatory support, or gastrostomy tube placement (21.7% vs. 59.1%; 55% absolute risk reduction, log-rank, p = 0.0125), reduction in the proportion free of > 6-point ALSFRS-R decline (48% vs. 18%; p = 0.035, chi-square test), decreased decline of quality of life assessed by the ALS Specific QoL-SF (LS mean difference: 0.9; 95% CI: 0.2 to 1.6; p = 0.018). Additionally, there was a trend for decreased loss of the summated MUNIX percent change to week 36 in the prespecified assessment of limb-onset ALS (LS mean difference: 20.9%, 95% CI: −2.2%–44.0%, p = 0.074), and a trend for improvement in the rank sum score for the combined assessment of function and survival (CAFS) (LS mean difference: 9.1; 95% CI: −5.8 to 23.9; p = 0.224). All of these endpoints were prespecified exploratory outcomes.

The long-term OLE data through a minimum of 52-weeks of CNM-Au8 treatment (for participants originally randomised to placebo) provided additional signals of potential CNM-Au8 treatment benefit. Notably, analyses of ALS disease progression events continued to favour original CNM-Au8 randomised participants through 120-weeks from the initial randomisation: original CNM-Au8 randomisation, percent event free: 46.2% (95% CI: 23.8%–68.6%) compared to original placebo randomisation: 15.3% (95% CI: 0%–31.1%). Similarly, there were fewer death and tracheostomy events within the original CNM-Au8 randomisation group. Random slopes models incorporating the OLE treatment period showed benefits in favour of participants originally randomised to CNM-Au8 including for ALSFRS-R change and ALSSQOL-change, including baseline through week 48 (12 weeks into the OLE) and from week 60 through 120 weeks for ALFRS-R change.

The reduction in all-cause mortality observed with CNM-Au8 following long term treatment is notable. These preliminary results from the 12-month data cut, following the LPLV of the double-blind period, included survival status from nearly all study participants. Three mortality events were recorded during the double-blind period, while an additional 19 mortality events were recorded after the double-blind period during long-term follow-up of survival status. These results may be expected to underestimate the true effect of active treatment on overall survival compared with a true placebo, due to the crossover design of these survival analyses, as ex-placebo participants were treated with CNM-Au8 in the OLE.^35^^,^^36^ These preliminary survival results should similarly be evaluated prospectively in an appropriately powered Phase 3 study.

Historical studies report that 20–25% of ALS participants discontinue a clinical trial, with discontinuation rates being higher in participants with more severe disease.^37^ In RESCUE-ALS, in the active treatment group, there were no participant withdrawals and one death, and only 3 withdrawals in the placebo group (2 deaths and one discontinuation, all due to disease progression).

While larger and potentially longer duration clinical trials are required to confirm the clinical benefits of CNM-Au8 treatment, the RESCUE-ALS results suggest that continued studies of this novel therapeutic approach for treatment of neurodegenerative diseases by targeting CNS energy metabolism pathways are warranted given the multiple signals of clinical efficacy observed in the exploratory outcomes and improved survival following long term treatment.

Preliminary results announced from the recently completed Phase 2 Healey ALS Platform trial^38^ indicate that CNM-Au8 treatment did not slow ALSFRS-R change following 24 weeks of treatment,^39^ which is consistent with *post hoc* evaluation of these RESCUE-ALS results at 24 weeks. We hypothesize that slow uptake and absorption of CNM-Au8 may result in a lengthy subtherapeutic period before drug tissue concentrations reach a therapeutic index necessary to influence long-term survival and functional change. Indeed, more pronounced change associated with the exploratory clinical outcomes following 36 weeks of double-blind treatment were observed here with respect to treatment differences versus placebo when contrasted with *post hoc* evaluation of 24 week treatment effects, where limited treatment effects were observed in this trial after only 6 months of treatment.

A larger international multicentre clinical trial (RESTORE-ALS) powered to investigate clinical outcomes is planned to commence in 2023 based on these RESCUE-ALS findings and the forthcoming results from the HEALEY ALS Platform trial.

## Contributors

The study was designed by collaboration among SV, MCK, and MTH. MTH wrote the protocol with contributions from AR, RG, KSH, SV, and MCK. PM and WH are the study site lead investigators. AR, JaE, JeE, and RG were responsible for supervision and study monitoring. CM, PM, SV, WH, and MCK conducted investigations. SL and AH conducted the statistical analyses. SV, PM, WM, and MCK had access to blinded participant level data, but remain blinded due to the ongoing open-label extension. SV and MCK (academic authors) verified the data. AR, MTH, and SL had access to the unblinded treatment allocation and verified data and analyses. KSH and MTH wrote the first draft of this manuscript. All authors contributed to the writing, editing, and preparation of the final manuscript. Every author had access to the full dataset if they had wanted and there were no restrictions to access. SV and MCK had final responsibility for the decision to submit for publication.

*Concept and design:* SV, MCK, MTH.

*Acquisition, analysis, or interpretation of data:* SV, MCK, MTH, SL, AH, PM, WH, CM, RG, JaE, JeE.

*Drafting of the manuscript*: KSH, SV, MCK, MTH, SL, AH, RG.

*Critical revision of the manuscript for important intellectual content:* KSH*,* SV, MCK, MTH, RG, AH, SL.

*Statistical analysis*: SL, AH.

*Obtained* funding*:* KSH, MTH, SV, MCK.

*Supervision:* SV, MCK.

## Data sharing statement

Individual control participant data that underlie the results reported in this article, after de-identification will be made available to investigators whose proposed use of the data has been approved by an independent review committee (“learned intermediary”) selected by the sponsor. Data will be made available beginning 9 months and ending 36 months following article publication. Proposals should be directed to info@clene.com; to gain access, data requestors will need to sign a data access agreement.

## Declaration of interests

AR, KSH, RG, AH, JaE, JeE, and MTH are full time employees and hold stock or options in Clene Nanomedicine. SL is an employee of Instat Clinical Research, a clinical research organization contracted by Clene Nanomedicine. SV and MCK are directors of companies that hold equity in Clene Nanomedicine. SV, MCK, and their respective institutions receive research funding support for the Rescue-ALS study from Clene Nanomedicine. MCK was Editor-in-Chief of the Journal of Neurology, Neurosurgery and Psychiatry (BMJ Publishers, UK).

## Appendix A Supplementary data

Supplementary Figures S1–S6 and Tables S1–S4

Supplementary material_SAP

Supplementary material_Study Protocol
