# Supplementary material for: Efficacy and safety of CNM-Au8 in amyotrophic lateral sclerosis (RESCUE-ALS study): a phase 2, randomised, double-blind, placebo-controlled trial and open label extension
Source: eClinicalMedicine. 2023 Jun 8;60:102036. doi: 10.1016/j.eclinm.2023.102036 (PMC10314176; doi:10.1016/j.eclinm.2023.102036)
Supplement: Supplementary Figures S1–S6 and Tables S1–S4 [file mmc1.pdf]

Table of Contents

Supplemental Table Index

Supplemental Table 1. *Sensitivity Analyses for the Primary Endpoint Including the Effects of Incorporating Alternate Covariates in the Primary Outcome Model*.........2
Supplemental Table 2. *ALS Disease Progression Events During the Double-Blind Period*.........3
Supplemental Table 3. *Exploratory Neurophysiology Outcomes (ITT Population)*.........4
Supplemental Table 4. *Observed Values Reported During the OLE for ALSFRS-R, FVC (% predicted, and ALSSQOL-SF (ITT Population)*.........5

Supplemental Figure Index

Supplemental Figure 1. Comparison of Key Baseline Characteristics by Randomisation Group 6
Supplemental Figure 1. Summated MUINIX Change for Placebo Bulbar and Limb Onset Participants.....6
Supplemental Figure 2. Time Course of MUNIX Change for ITT and Limb Onset Populations.........8
Supplemental Figure 3. ALS Disease Progression Event Free by Study Visit.........9
Supplemental Figure 4. ALSFRS-R 6-Point Decline Responder and ALSSQOL-SF Change 10
Supplemental Figure 5. ALSFRS-R Random Slopes Models during the OLE.........11
Supplemental Figure 6. Death or Tracheostomy Events in OLE Participants.........12

1. Supplementary Tables

Supplemental Table 1. *Sensitivity Analyses for the Primary Endpoint Including the Effects of Incorporating Alternate Covariates in the Primary Outcome Model*

Covariate	F-Value	Test for Fixed Effect (p>F)
Baseline Value	0·17	0·998
Enrolling Site	0·27	0·609
Delta-FS (Pre-Baseline Slope)	4·37	0·044
Age	0·20	0·654
ENCALS Risk Score	3·29	0·078
Use of Riluzole	0·38	0·543
Visit Number	27·0	<0.001

Supplemental Table 2. *ALS Disease Progression Events During the Double-Blind Period*

Disease Progression Events	CNM-Au8 30mg	Placebo
**Death**	1	2
**Tracheostomy**	0	0
**Gastrostomy tube**	2	7
**Non-invasive ventilation**	2	4
**Total Events**	5	13

Supplemental Table 3. *Exploratory Neurophysiology Outcomes (ITT Population)*

**Neurophysiology Outcomes**	**Least-Squares Mean Change** (SE) at week 36		**LS Mean Difference vs. Placebo** (95% CI) at week 36	**Percent Event Free** (95% CI) at week 36		**ARR[5] vs. Placebo**
	**CNM-Au8 30mg**	**Placebo**		**CNM-Au8 30mg**	**Placebo**	
Joint-Rank (Survival or Summated MUNIX Score)	3·4 (5·4)	-3·4 (5·4)	6·7 (-8·8, 22·3)	NA		
Split Hand Index [1]	-3·1 (0·6)	-2·9 (0·7)	-0·2 (-2·0, 1·7)			
Neurophysiology Index [2]	-1·0 (0·2)	-0·65 (0·3)	-0·35 (-1·1, 0·37)			
MUSIX [3]	17·2 (15·0)	32·4 (16·3)	-15 (-60, 30)			
MScanFit MUNE [4]	-59·3 (8·2)	-49·0 (8·5)	-10·3 (-34·4,13·7)			
MUNIX ADM [5]	-12·2 (18·2)	-29·7 (20·5)	17·5 (-38·1, 73·1)			
MUNIX APB [5]	-49·2 (11·4)	-56·9 (12·2)	7·7 (-26·4, 41·9)			
MUNIX TA [5]	-18·1 (16·3)	-41·8 (17·1)	23·7 (-27·7, 72·1)			
MUNIX BB [5]	-25·8 (8·7)	-23·6 (9·3)	-2·2 (-28·1, 23·7)			
Summated MUNIX >= -15% Decline Responder	NA			22%	18%	4%
Summated MUNIX >= -25% Decline Responder				22%	23%	-1%

[1] Split Hand Index is calculated a the first dorsal interosseous (FDI)$^{\text{Peak CMAP}}$ Amplitude * APB$^{\text{Peak CMAP}}$ Amplitude/ADM$^{\text{Peak CMAP}}$ Amplitude.

[2] Neurophysiology Index is calculated as the (ADM$^{\text{CMAP Peak Amplitude}}$ / ADM$^{\text{Distal Motor Latency}}$) * ADM$^{\text{F-Wave (\%)}}$.

[3] MUSIX reported as the average of the values for the ADM, APB, BB, and TA, calculated as the percent change from the baseline visit indexed to 100%.

[4] MScanFit MUNE is reported as the percent change from the baseline visit indexed to 100%.

[5] Percent MUNIX change for the abductor digit minimi (ADM), abductor pollicis brevis (APB), biceps brachii (BB), and tibialis anterior (TA), where each participant's baseline MUNIX value was indexed to 100%.

Supplemental Table 4. *Observed Values Reported During the OLE for ALSFRS-R, FVC (% predicted, and ALSSQOL-SF (ITT Population)*

OLE Observed Values as of 14-July-2022<br>ITT Population		**Original Active**	**Original Placebo**	**All OLE Participants**
**ALSFRS-R**				
OLE Week 12	Mean (SD)	30.4 (10.19)	32.4 (6.14)	31.3 (8.61)
	Range	11.0, 46.0	23.0, 42.0	11, 46
	n	16	12	28
OLE Week 24	Mean (SD)	26.3 (11.37)	27.3 (9.58)	26.8 (10.4)
	Range	13.0, 46.0	7.0, 42.0	7, 46
	n	15	13	28
OLE Week 36	Mean (SD)	24.6 (12.21)	26.0 (10.79)	25.3 (11.36)
	Range	9.0, 46.0	8.0, 42.0	8, 46
	n	15	14	29
OLE Week 48	Mean (SD)	25.9 (11.41)	23.9 (12.27)	24.9 (11.63)
	Range	11.0, 46.0	3.0, 38.0	3, 46
	n	12	12	24
OLE Week 60	Mean (SD)	31.3 (9.58)	25.8 (12.0)	28.1 (11.00)
	Range	21.0, 46.0	1.0, 39.0	1, 46
	n	6	8	14
OLE Week 72	Mean (SD)	32.0 (9.27)	23.6 (11.48)	27.8 (10.79)
	Range	21, 46	6, 37	6, 46
	n	5	5	10
**FVC (% predicted)**				
OLE Week 12	Mean (SD)	66.3 (29.21)	64.0 (21.38)	65.17 (25.21)
	Range	20.0, 112.0	31.0, 97.0	20, 112
	n	12	11	23
OLE Week 24	Mean (SD)	83.2 (21.63)	62.1 (19.87)	71.1 (22.57)
	Range	57, 110	36, 95	36, 110
	n	6	8	14
OLE Week 36	Mean (SD)	64.8 (39.49)	44.5 (26.13)	56.7 (34.7)
	Range	1, 107	22, 81	1, 107
	n	6	4	10
OLE Week 48	Mean (SD)	62.7 (33.33)	59.6 (28.66)	61.2 (30.29)
	Range	12, 109	25, 99	12, 109
	n	9	8	17
OLE Week 60	Mean (SD)	78.8 (32.34)	64.7 (26.56)	70.6 (28.59)
	Range	37, 111	32, 99	32, 111
	n	5	7	12
OLE Week 72	Mean (SD)	77.8 (28.08)	62.3 (29.53)	70.9 (28.08)
	Range	48, 109	36, 96	36, 109
	n	5	4	9
**ALS Specific QoL**				
OLE Week 12	Mean (SD)	6.7 (1.44)	6.5 (1.49)	6.6 (1.43)
	Range	4.1, 9.3	2.9, 8.5	2.9, 9.3
	n	16	12	28
OLE Week 24	Mean (SD)	6.1 (1.4)	6.4 (1.23)	6.2 (1.3)
	Range	4.1, 9.6	3.9, 8.7	3.9, 9.6
	n	14	13	27
OLE Week 36	Mean (SD)	6.0 (1.35)	6.6 (1.21)	6.3 (1.3)
	Range	4.1, 9.1	4.4, 9.3	4.1, 9.3
	n	15	14	29
OLE Week 48	Mean (SD)	6.2 (1.83)	6.1 (1.43)	6.1 (1.6)
	Range	2.8, 9.3	3.6, 9.3	2.8 (9.3
	n	11	12	23
OLE Week 60	Mean (SD)	7.0 (1.62)	6.8 (1.55)	6.9 (1.52)
	Range	5.0, 8.7	4.6, 9.1	4.6, 9.1
	n	6	8	14
OLE Week 72	Mean (SD)	6.9 (1.47)	6.4 (1.84)	6.7 (1.59)
	Range	5.4, 8.6	4.3, 8.6	4.3, 8.6
	n	5	5	10

2. Supplementary Figures

Supplemental Figure 1. *Comparison of Key Baseline Characteristics by Randomisation Group*

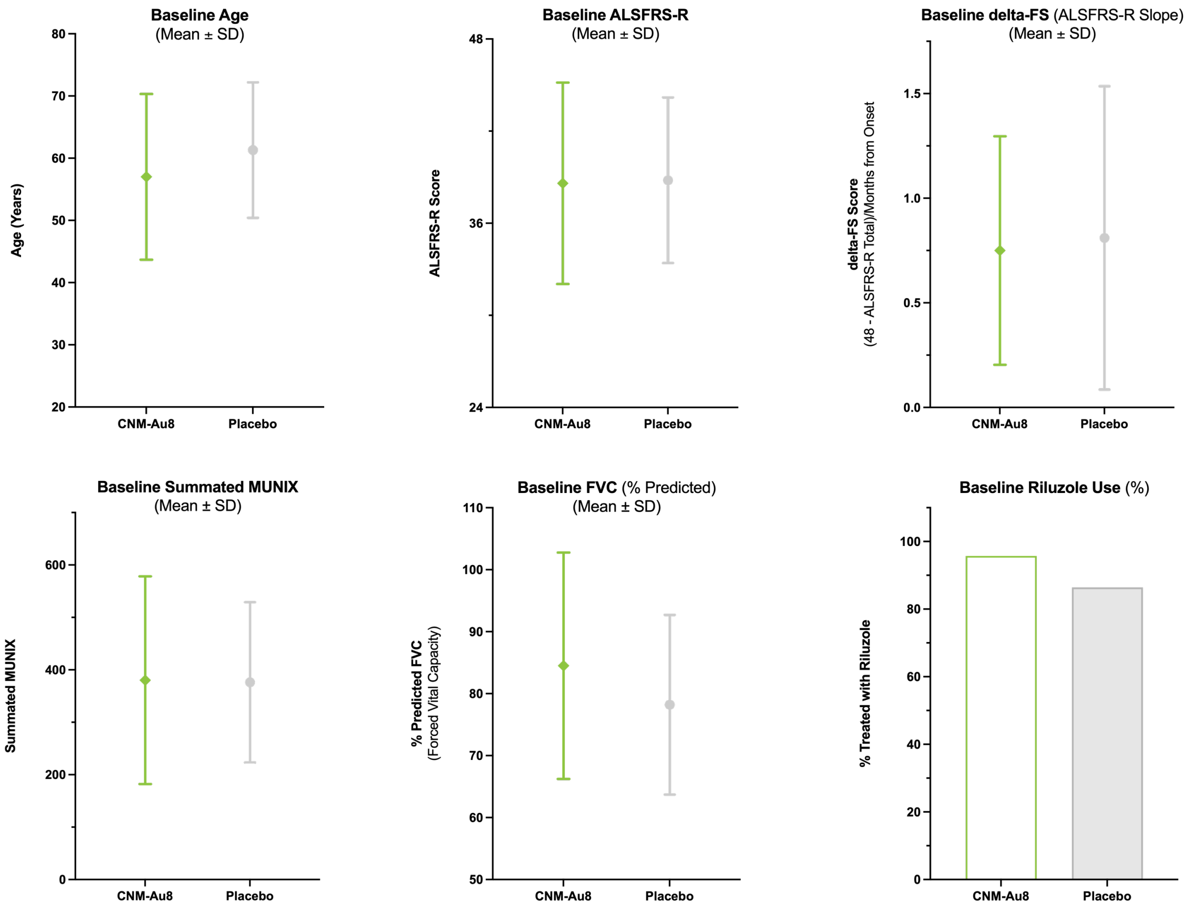

Supplemental Figure 1 Legend. Comparison of baseline characteristics by randomisation group for age, ALSFRS-R total score, delta-FS (pre-treatment ALSFRS-R slope), summated MUNIX of the ADM, APB, BB, and TA; Forced Vital Capacity (% predicted), and percent treated by riluzole at baseline.

Supplemental Figure 2. *Summated MUINIX Change for Placebo Bulbar and Limb Onset Participants*

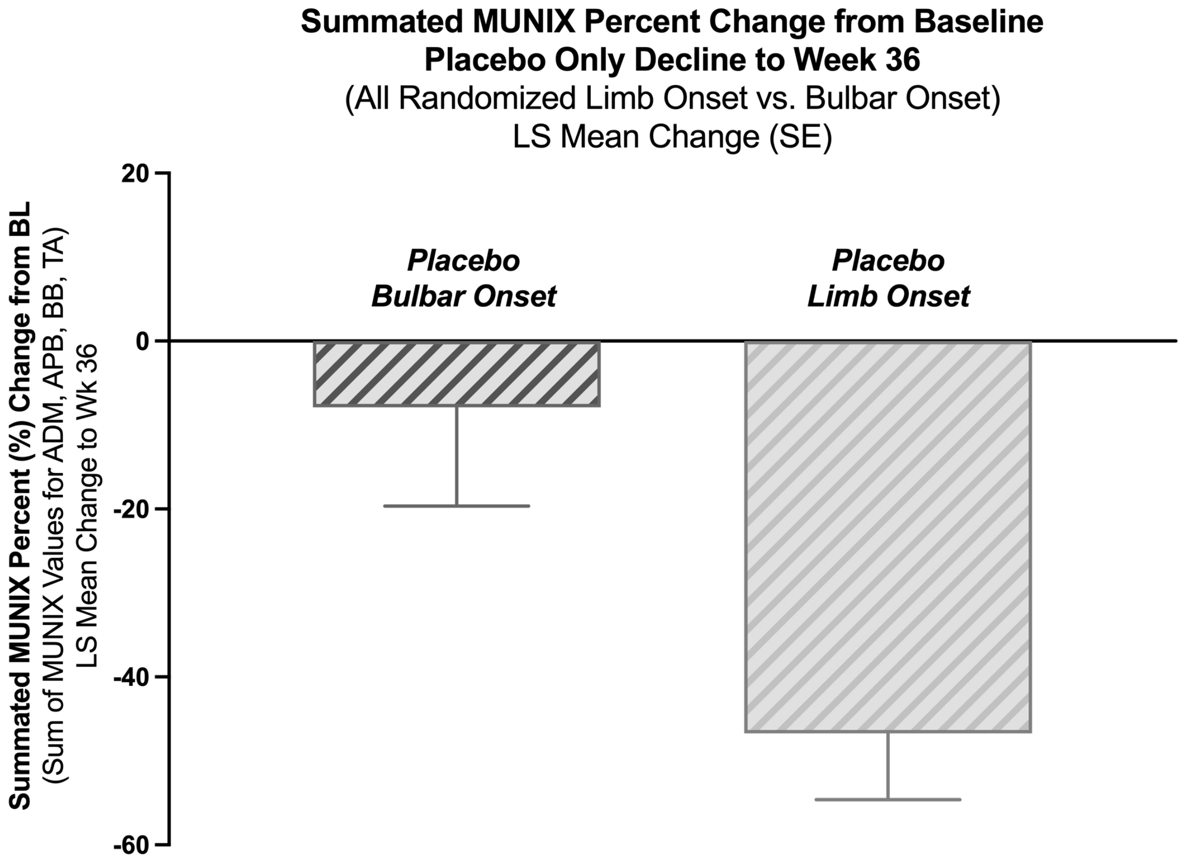

Supplemental Figure 2 Legend. Comparison of MUNIX decline in limb-onset and bulbar onset placebo groups revealed an unexpected lack of decline in bulbar-onset MUNIX scores over 36 weeks. Summated MUNIX percent change for the abductor digit minimi (ADM), abductor pollicis brevis (ABP), biceps brachii (BB), and tibialis anterior (TA), where each participant's baseline summated MUNIX value was indexed to 100%. ITT: Intent to treat; LS Mean: least squares mean; SE: standard error. CI: 95% confidence interval.

Supplemental Figure 3. *Time Course of MUNIX Change for ITT and Limb Onset Populations*

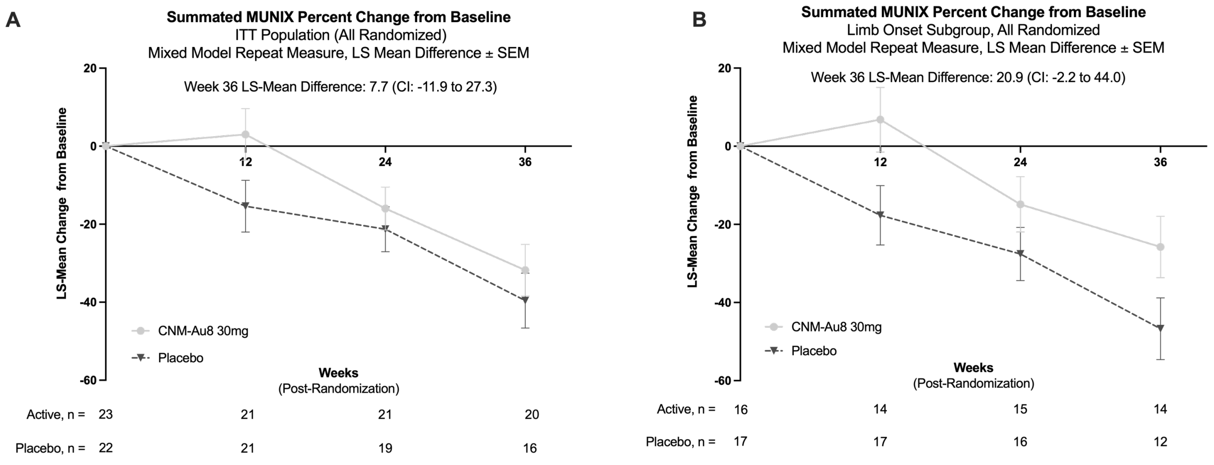

Supplemental Figure 3 Legend. Summated MUNIX Results by 12-week clinical visit for the ITT and Limb-Onset Populations. *Left (A)* ITT Population. *Right (B)* Limb Onset population. Summated MUNIX percent change for the abductor digit minimi (ADM), abductor pollicis brevis (ABP), biceps brachii (BB), and tibialis anterior (TA), where each participant's baseline summated MUNIX value was indexed to 100%. ITT: Intent to treat; LS Mean: least squares mean; SE: standard error. CI: 95% confidence interval.

Supplemental Figure 4. *ALS Clinical Worsening Event Free by Study Visit*

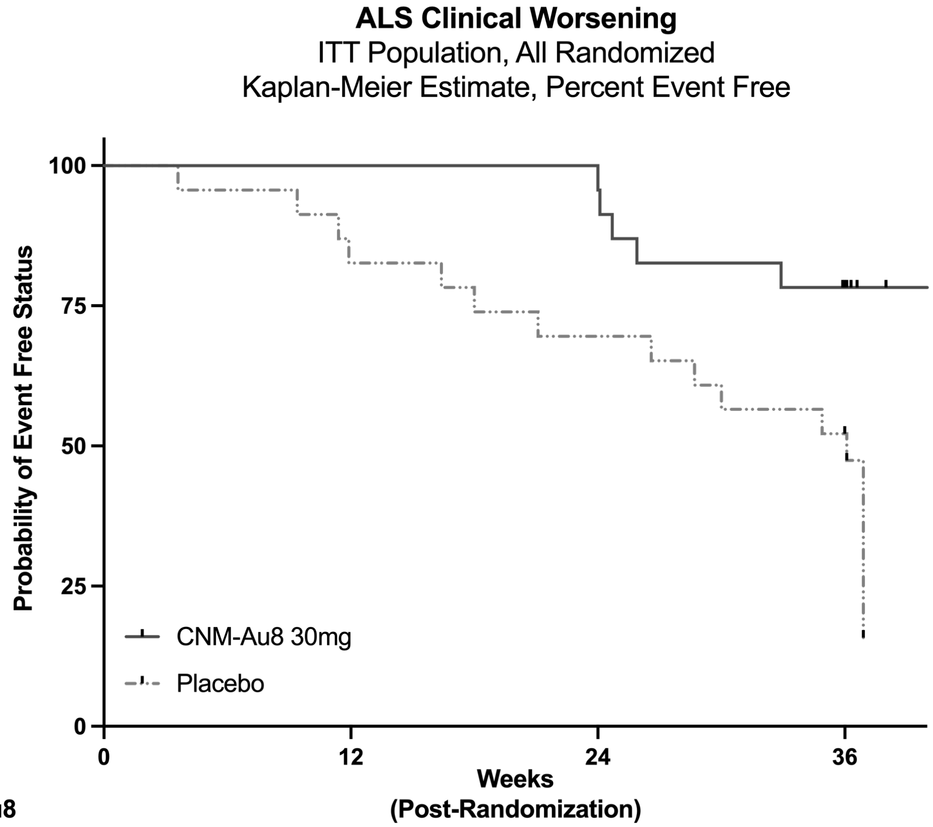

**CNM-Au8**				
At Risk (n)	23	23	23	20
With Event	0	0	3	5
**Placebo**				
At Risk (n)	22	22	18	15
With Event	0	4	7	13

Supplemental Figure 4 Legend. Time to ALS Disease Progression. ALS Disease Progression was defined as the first occurrence of death, tracheostomy, or need for non-invasive ventilatory support or gastrostomy tube placement.

Supplemental Figure 5. *ALSFRS-R 6-Point Decline Responder and ALSSQOL-SF Change*

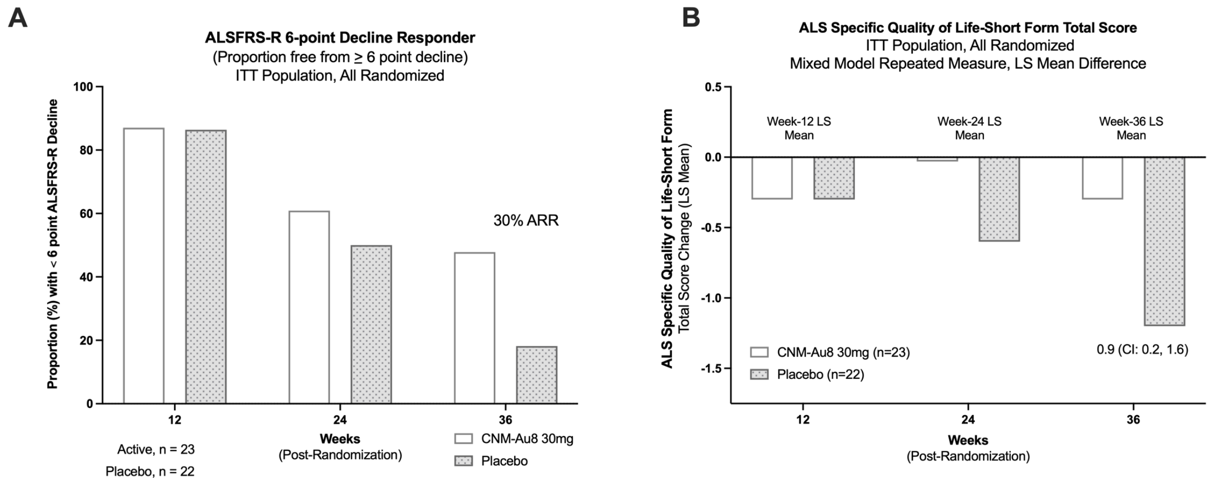

Supplemental Figure 4. Exploratory Clinical Outcomes. *Left (A).* Proportion Free From ≥6-point ALSFRS-R Decline. *Right (B)*. ALSSQOL-SF Total Score LS Mean Change**.** Left, Panel A. Proportion free from ≥6-point ALSFRS-R decline. Deaths and study withdrawals are considered as non-responders. Right, Panel B. ALS Specific QoL Short Form Total Score change to week 36. Mixed model for repeat measures with treatment, visit, treatment by visit interaction as fixed effects, and baseline value, and ENCALS score as covariates. An unstructured covariance model was used. LS: Least-squares mean change. ITT: intent to treat; LS Mean: least squares mean.

Supplemental Figure 6. *ALSFRS-R Random Slopes Models during the OLE*

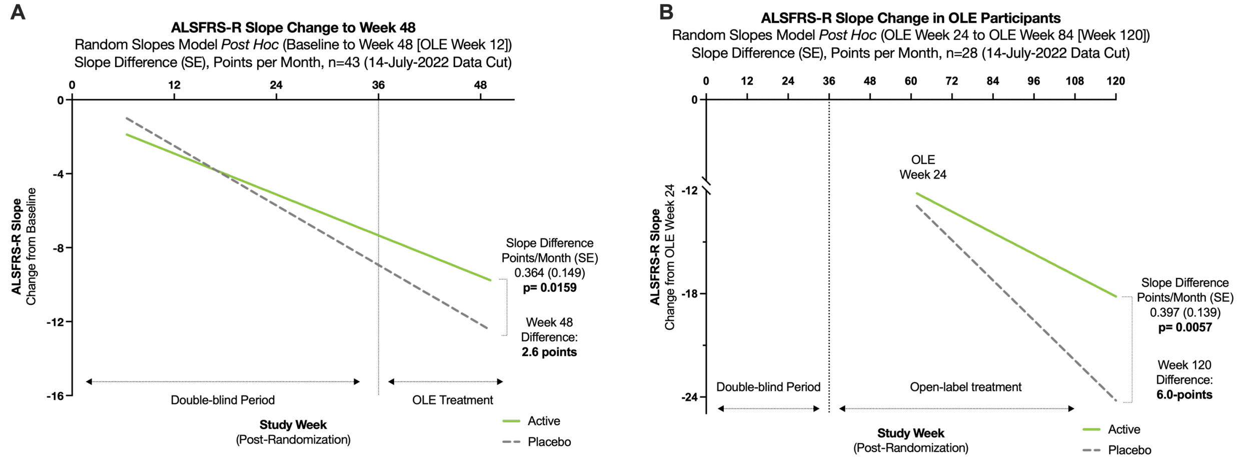

Supplemental Figure 6. OLE Random Slopes Models for ALSFRS-R Change. *Left (A).* ALSRSR-R Change from Randomisation to Week 48. *Right (B)*. ALSRSR-R Change from Week 60 to Week 120. Left, Panel A. Slope of change for original active and original placebo randomisation from Baseline to Week 48. Right, Panel B. Slope of change for original active and original placebo randomisation from Study Week 60 to Week 120. The random slope models incorporated covariates including delta-FS, time from symptom onset, treatment assignment, and time (days).

Supplemental Figure 7. *Death or Tracheostomy Events in OLE Participants*

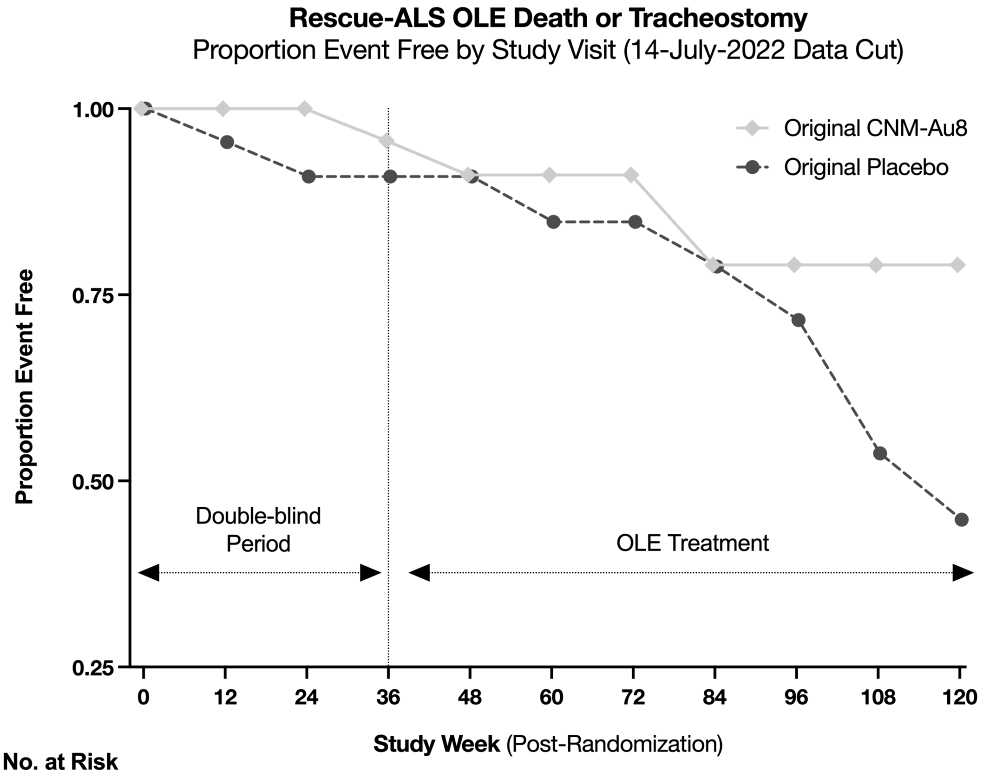

No. at Risk

	0	12	24	36	48	60	72	84	96	108	120
Original CNM-Au8:	23	23	23	23	22	21	21	21	19	19	19
Original Placebo:	22	21	20	20	20	20	19	19	18	17	15

Cumulative Events

	0	12	24	36	48	60	72	84	96	108	120
Original CNM-Au8:	0	0	0	1	2	2	2	4	4	4	4
Original Placebo:	0	1	2	2	2	3	3	4	5	7	8

Supplemental Figure 6 Legend. Death or Tracheostomy Events in OLE Participants. Proportion without death or tracheostomy by 12-week clinical visit amongst OLE participants.
