# Supplementary material for: Efficacy and safety of CNM-Au8 in amyotrophic lateral sclerosis (RESCUE-ALS study): a phase 2, randomised, double-blind, placebo-controlled trial and open label extension
Source: eClinicalMedicine. 2023 Jun 8;60:102036. doi: 10.1016/j.eclinm.2023.102036 (PMC10314176; doi:10.1016/j.eclinm.2023.102036)
Supplement: Supplementary material_SAP [file mmc2.pdf]

STATISTICAL ANALYSIS PLAN

STUDY NUMBER: CNMAu8.205

RESCUE-ALS

A PHASE 2, RANDOMIZED, DOUBLE-BLIND, PLACEBO-CONTROLLED STUDY IN EARLY SYMPTOMATIC AMYOTROPHIC LATERAL SCLEROSIS PATIENTS ON STABLE BACKGROUND THERAPY TO ASSESS BIOENERGETIC CATALYSIS WITH CNM-AU8 TO SLOW DISEASE PROGRESSION IN ALS

**Document version:**	**Final v2.0**
**Release date:**	**14Oct2021**
**Protocol:**	**CNMAu8.205**
**Prepared by:**	**Instat Clinical Research**<br>**Shelia Ligozio**<br>**Director, Biostatistics**

This study will be conducted in compliance with the Protocol, Good Clinical Practice and all other applicable regulatory requirements, including the archiving of essential documents.

DOCUMENT HISTORY

Version Number	Author	Date	Change
V1.0	S. Ligozio	7Sep2021	Final
V2.0	S. Ligozio	14Oct2021	Correct Munix Responder Analysis definition, Add possible exploratory analysis using historical controls, fix typos

SIGNATURE PAGE

STUDY NUMBER CNMAu8.205

RESCUE-ALS

A PHASE 2, RANDOMIZED, DOUBLE-BLIND, PLACEBO-CONTROLLED STUDY IN EARLY SYMPTOMATIC AMYOTROPHIC LATERAL SCLEROSIS PATIENTS ON STABLE BACKGROUND THERAPY TO ASSESS BIOENERGETIC CATALYSIS WITH CNM-Au8 TO SLOW DISEASE PROGRESSION IN ALS

**Author:**	
Shelia Ligozio<br>Director, Biostatistics<br>Instat Clinical Research	Signature: Shelia Ligozio<br>Shelia Ligozio (Oct 14, 2021 16:56 EDT)<br>Date: Oct 14, 2021
**Sponsor Representatives:**	
Austin Rynders<br>Head of Clinical Operations<br>Clene Nanomedicine	Signature: [signature]<br>Date: Oct 14, 2021
Robert Glanzman<br>Chief Medical Officer<br>Clene Nanomedicine	Signature: [signature]<br>Robert Glanzman (Oct 14, 2021 14:55 MDT)<br>Date: Oct 14, 2021
Michael Hotchkin<br>Chief Development Officer<br>Clene Nanomedicine	Signature: Michael Hotchkin<br>Date: Oct 14, 2021

TABLE OF CONTENTS

1. Introduction 7
1.1 Study Design 7
1.2 Study Objectives 11
2. Study Populations 11
3. Statistical Methodology 12
3.1 Determination of Sample Size 13
3.2 Handling of Missing Data 14
3.3 Subject Characteristics 15
3.3.1 Disposition 15
3.3.2 Protocol Deviations 15
3.3.3 Demographics and Baseline Characteristics 15
3.3.4 ALS History/Status and Medical History 15
3.3.5 Prior and Concomitant Medications 16
3.4 Efficacy Analyses 16
3.4.1 Primary Efficacy Endpoint 18
3.4.2 Secondary Efficacy Endpoints 20
3.4.3 Exploratory Efficacy Endpoints 21
3.4.4 Subgroup Analyses 31
3.5 Pharmacokinetic and Pharmacodynamic Endpoints 31
3.6 Safety Analyses 32
3.6.1 Extent of Exposure 32
3.6.2 Adverse Events 33
3.6.3 Clinical Laboratory Tests 34
3.6.4 Physical Examinations and Brief Neurological Examinations 34
3.6.5 Vital Signs 35
3.6.6 Electrocardiogram 35
3.6.7 Columbia Suicide Severity Rating Scale 35
3.6.8 Falls Questionnaire 35
4. Interim Analysis 36
5. Tables, Listings, Figures 36
6. REFERENCES 37

GLOSSARY AND ABBREVIATIONS

Abbreviation	Definition
ADM	Abductor Digit Minimi
AE	Adverse Event
AICC	Akaike's Information Criteria Corrected
ALS	Amytrophic Lateral Sclerosis
ALSFRS-R	ALS Functional Rating Score-Revised
ALSSQOL-SF	ALS Specific Quality of Life - Short Form
APB	Abductor Pollicis Brevis
Au	Gold
BB	Biceps Brachii
BMI	Body Mass Index
CAFS	Combined Assessment of Function and Survival
CI	Confidence Interval
CGI	Clinical Global Impression
CMAP	Compound Muscle Action Potential
CRF	Case Report Form
CSR	Clinical Study Report
C-SSRS	Columbia Suicide Severity Rating Scale
DSMB	Data Safety and Monitoring Board
ECG	Electrocardiogram
EMG	Electromyography
ENCALS	European Network for the Cure of ALS
FDI	First Dorsal Interosseous
FS	Functional Status
FVC	Forced Vital Capacity
ITT	Intent-to-treat
LOCF	Last Observation Carried Forward
MCMC	Markov Chain Monte Carlo
MedDRA	Medical Dictionary for Regulatory Activities
MiToS	Milano-Torino Staging
MMRM	Mixed Effect Model for Repeated Measurement
MUNE	Motor Unit Number Estimation
MUNIX	Motor Unit Number Index
MUSIX	Motor Unit Size Index
NP_{index}	Neurophysiology Index
NRI	Non-Response Imputation
OLE	Open Label Extension

Abbreviation	Definition
PD	Pharmacodynamics
PDF	portable document file
PGI	Patient Global Impression
PK	Pharmacokinetics
Q1	First Quartile
Q3	Third Quartile
SAE	Serious Adverse Event
SAP	Statistical Analysis Plan
SD	Standard Deviation
SEM	Standard Error of the Mean
SH_{index}	Split Hand Index
TA	Tibialis Anterior
TEAE	Treatment-Emergent Adverse Event
WHO	World Health Organization

1. Introduction

The purpose of this statistical analysis plan (SAP) is to ensure the statistical methodologies that will be used are complete and appropriate to allow valid conclusions regarding the study objectives. Results obtained from the analyses outlined in this document will be the basis of the final clinical study report (CSR) for this protocol. Any deviations from this SAP will be documented in the final CSR.

The pharmacokinetics (PK) and pharmacodynamics data (PD) will be analyzed outside of this SAP. Therefore, analyses planned for these data will be described in separate analysis plans.

1.1 Study Design

This is a multi-center randomized, double-blind, parallel group, placebo-controlled study of the efficacy, safety, pharmacokinetics, and pharmacodynamics of CNM-Au8 in subjects who are newly symptomatic within 24 months of screening and with a clinically probable, possible, or definite amytrophic lateral sclerosis (ALS) diagnosis per the Awaji-Shima criteria (de Carvalho et al., 2008).

Subjects will be randomized in a 1:1 ratio into one of two treatment groups: CNM-Au8 30 mg or Placebo. All subjects will receive their randomized oral treatment daily over thirty-six consecutive weeks during the Treatment Period. There will be up to four study periods:

(1) A 6-week screening period (Screening Period)

(2) A 36-week blinded treatment period (Treatment Period)

(3) A 48-week optional open-label extension (OLE) period (Open-Label Period)

(4) A 4-week safety follow-up period following completion or early termination of either the Treatment Period or Open-Label Period (Safety Follow-Up Period).

Approximately 42 subjects will be randomized into the study. Subjects who complete the Treatment Period but choose not to continue in the Open-Label Period or discontinue early from the Treatment Period will directly enter the 4-week Safety Follow-Up Period.

An independent data safety and monitoring board (DSMB) will be responsible for monitoring the safety of the study during the Treatment Period on a periodic basis or ad hoc at the request of the DSMB or Sponsor.

The visit schedule (without the optional Open-Label Period) is shown in Table 1 below. Table 2 shows the Open-Label Period visit schedule.

Table 1. Time and Events Schedule

**Time and Events Schedule**	**Visit**	**-1**	**0**	**1**	**2**	**3**	**4**	**5**	**6**	**7**	**8**
	**Phase**	**Screening**	**Baseline**	**Treatment Period**							**Safety Follow-Up**[b]
	**Week**	**-6**	**0**	**3**	**6**	**12**	**18**	**24**	**30**	**36**	**40**
	**Day**	-42 to -1	1	21[a]	42[a]	84[a]	126[a]	168[a]	210[a]	252[a]	280[c]
ICF Signed		X									
Eligibility Review		X	X								
Medical History & Prior Med Assess.		X									
Physical Examination		X	X			X[d]		X[d]		X[d]	X
Brief Neurological Exam		X	X			X		X		X	X
Anthropometrics (Height/Weight)		X	X[e]			X[e]		X[e]		X[e]	
Urine Drug Test		X									
HIV/Viral Hepatitis Screen		X									
Serum Pregnancy Test		X[f]									
Urine Pregnancy Test			X[f]			X[f]		X[f]		X[f]	
Vital Signs		X	X			X		X		X	X
12-lead ECG		X[g]	X[g]			X[g]		X[g]		X[g]	
Clinical Laboratory (Blood)		X	X			X		X		X	X
Urinalysis		X	X			X		X		X	X
Concomitant Medication		X	X	X	X	X	X	X	X	X	X
Randomization			X								
Dispense/Return Drug			X			X		X		X	
PD Sampling (Blood, Urine)			X[h]			X[h]		X[h]		X[h]	
PK Sampling (Blood)						X[h]		X[h]		X[i]	
Adverse Events		X	X	X	X	X	X	X	X	X	X
EMG: MUNIX, MUSIX, CMAP of the APB, ADM, BB, TA, FDI			X[j]			X[j]		X[j]		X[j]	
EMG: ADM, APB (F-Wave); Distal Motor Latency (APB, ADM, BB, TA, FDI)			X[j]			X[j]		X[j]		X[j]	
EMG: MScan for the APB			X[j]			X[j]		X[j]		X[j]	
ALSFRS-R		X	X			X		X		X	
FVC		X	X			X		X		X	

Time and Events Schedule	Visit	-1	0	1	2	3	4	5	6	7	8
	Phase	Screening	Baseline	Treatment Period							Safety Follow-Up[b]
	Week	-6	0	3	6	12	18	24	30	36	40
	Day	-42 to -1	1	21[a]	42[a]	84[a]	126[a]	168[a]	210[a]	252[a]	280[c]
ALSSQOL-SF			X			X		X		X	
Health Utilization Form (e,g., PEG, NIV, Wheel Chair)			X			X		X		X	
Falls Questionnaire						X		X		X	
PGI			X			X		X		X	
CGI			X			X		X		X	
C-SSRS		X	X			X		X		X	X
Phone call				X	X		X		X		

a. Scheduled Visit ± 5 days.
b. For subjects not transitioning to the optional Open-Label extension (OLE) phase.
c. Timing for the Safety Follow-Up visit should occur at 4 weeks (± 5 days) following study early termination or following the subject's final Week 36 visit if the participant chooses not to enter the optional OLE period.
d. Brief physical exam only.
e. Weight only.
f. For females of childbearing potential only.
g. Electrocardiogram (ECG) intervals will be summarized and presented descriptively. ECG rhythm will be interpreted by the Investigator as normal, abnormal not-clinically significant (aNCS), or abnormal clinically significant (aCS). Triplicate values will be collected at Baseline and averaged for comparison to single assessments at subsequent visits.
h. Whole blood, plasma, and/or serum for PK and PD will be taken pre-dose only (~1 hour prior to the dose of study drug)
i. Whole blood for PK will be taken at pre-dose (T0) and at 1, 2, 4, and 6 hours after dosing for the visit. The exact time at which the subject took his/her previous day's study drug dose must be recorded in order to impute a 24-hour trough value (T24-imputed).
j. EMG to be conducted on the least clinically affected hand, leg, and arm identified at the Baseline visit.

Table 2. Time and Events Schedule for Open-Label Treatment Period

Time and Events Schedule	Visit	0	1	2	3	4	5	6
	Phase	Baseline[a]	Open-Label Extension Phase					Safety Follow-Up[c]
	Week	0	6	12	24	36	48	52
	Day	0	42[b]	84[b]	168[b]	252[b]	336[b]	364[b]
ICF Signed		X						
Eligibility Review and Confirmation		X						
Brief Physical Examination		X		X	X	X	X	X
Brief Neurological Exam		X		X	X	X	X	X
Weight Assessment		X			X		X	

Time and Events Schedule — Visit	0	1	2	3	4	5	6
Phase	Baseline[a]	Open-Label Extension Phase					Safety Follow-Up[c]
Week	0	6	12	24	36	48	52
Day	0	42[b]	84[b]	168[b]	252[b]	336[b]	364[b]
Urine Pregnancy Test[d]	X		X	X	X	X	
Vital Signs	X		X	X	X	X	X
12-lead ECG[e]	X			X		X	
Clinical Laboratory (Blood)	X		X	X	X	X	X
Urinalysis	X		X	X	X	X	X
Dispense/Return Drug	X		X	X	X	X	
PK/PD Sampling (Blood, Urine)	X			X[f]		X[f]	
Adverse Events and Concomitant Medications Review	X	X	X	X	X	X	X
EMG: MUNIX, MUSIX, CMAP of the APB, ADM, BB, TA, FDI	X[g]			X[g]		X[g]	
EMG: ADM, APB (F-Wave); Distal Motor Latency (APB, ADM, BB, TA, FDI)	X[g]			X[g]		X[g]	
EMG: MScan for the APB	X[g]			X[g]		X[g]	
ALSFRS-R	X		X	X	X	X	
FVC	X		X	X	X	X	
ALSSQOL-SF	X		X	X	X	X	
Health Utilization Form (e,g., PEG, NIV, Wheel Chair)	X		X	X	X	X	
Falls Questionnaire			X	X	X	X	
PGI	X		X	X	X	X	
CGI	X		X	X	X	X	
C-SSRS	X		X	X	X	X	X
Phone call		X					

a. The Baseline Visit for the optional OLE study will correlate with the Week 36 visit of the randomized placebo-controlled phase. For those participants who may enter this OLE phase 12 or more weeks after completing their Week 36 visit, Baseline assessment indicated in Table 2 will be completed and serve as the participants baseline for the OLE phase.
b. Scheduled Visit ± 5 days.
c. Timing for the Safety Follow-Up visit should occur at four weeks (±5 days) following early termination or the patient's Week 48 visit.
d. For females of childbearing potential only.
e. Electrocardiogram intervals will be summarized and presented descriptively. ECG rhythm will be interpreted by the Investigator as normal, abnormal not clinically significant, or abnormal clinically significant.
f. Whole blood, plasma, serum, and/or urine for PK and PD will be taken pre-dose only (e.g., within1 hour prior to the dose of study drug).

g. EMG to be conducted on the least clinically affected hand, leg, and arm identified at the randomized placebo-controlled phase Baseline visit.

1.2 Study Objectives

To assess the efficacy, safety, and PK/PD effects of CNM-Au8 as a disease-modifying agent for the treatment of ALS by utilizing electrophysiological measures to detect preservation of motor neuron function.

- Efficacy will be assessed as the difference between placebo and active treated patients for disease progression from baseline through the treatment period, as reflected by the change in motor neuron loss measured by electromyography (EMG) endpoints (e.g., Motor Unit Number Index (MUNIX), the primary endpoint; Motor Unit Size Index (MUSIX), Split Hand Index (SH_{Index}), Neurophysiology Index (NP_{Index}), MScanFit Motor Unit Number Estimation (MUNE)); and clinical endpoints, including respiratory function (FVC), the key secondary endpoint; ALS Functional Rating Score-Revised (ALSFRS-R) score and subscores, change in the rate of ALSFRS-R progression (delta-FS score), survival status, Combined Assessement of Function and Survival (CAFS), and composite disease progression (as defined in Section 3.4.3.8); and patient reported outcomes.

- Safety will be assessed via the frequency of adverse events, serious adverse events, discontinuations due to adverse events, Falls Questionnaire, and the Columbia Suicide Severity Rating Scale (C-SSRS).

- Pharmacodynamics and pharmacokinetics will be assessed through blood and urine collection at baseline and then every 12 weeks thereafter during the treatment period and every 24 weeks during the optional OLE.

2. Study Populations

The following analysis populations are to be used for this study:

- Intent to Treat Population (ITT) – The Intent to Treat population will consist of all screened subjects who were randomized to a treatment.

- Safety Population – The Safety population will consist of subjects in the ITT population who received at least one dose of study drug.

- Partial Analysis Population – The Partial Analysis population will consist of subjects in the ITT population with at least one post-baseline EMG measurement.

- Per Protocol Population – The Per Protocol population will consist of subjects in the Safety population who have completed 36 weeks of treatment and all primary and secondary endpoint assessments and who have an overall treatment compliance between 80-120%. An EMG and %predicted FVC assessment must be present at Week 36, but each individual EMG measurement does not have to be non-missing.

The table below outlines the populations to be used for the summaries in both the double-blind and open-label treatment periods.

Table 3. Population Summary

	**Analysis Populations**				
	**All Subjects**	**Intent-to-Treat**	**Partial Analysis**	**Per Protocol**	**Safety**
**Double-Blind Treatment Period**					
Disposition Summary	X				
Baseline Summary		X	X	X	X
Efficacy Summary		X	X	X	
Safety Summary					X
**Open-Label Treatment Period**					
Disposition Summary	X				
Baseline Summary		X			X
Efficacy Summary		X			
Safety Summary					X

3. Statistical Methodology

All study data will be presented in listings, tables, and/or figures. Continuous variables will be summarized using descriptive statistics (i.e., mean, standard deviation (SD), minimum, median, and maximum). Categorical variables will be summarized using frequency counts and percentages.

All summaries, statistical analyses, and individual subject data listings described below will be performed using Version 9.3 or later of the SAS Statistical Analysis System (SAS Institute, Inc. Cary, NC) or Version 3.6.3 or later of R, a statistical computing software. A study-wise Type I error (alpha) of 0.05 will be used for the study. P-values will be based on a 2-sided test.

Reporting precision in the tables will be to one decimal place for percentages and typically one decimal place more than the precision of the variable when collected/reported for the mean and median. The SD will be presented to one decimal place more than the mean, and the minimum and maximum will be presented to the same precision as the reported value or follow the mean/median precision if derived. Missing values will not be considered in percentage calculations, unless stated otherwise. In these cases, footnotes will specify the percentage denominator definition, or the denominator used will be presented with the summary. P-values will be presented to 4 decimal points. If a p-value is less than or equal to 0.0001, it will be reported as <0.0001. The table shells will specify if there is a deviation from the precision noted here. Data listings will be sorted by subject, dose level, and visit (if applicable). Tables, listings, and figures will be in portable document format (PDF) files.

Baseline will be defined as the last non-missing measurement prior to dosing. For the open label period summaries, this will likely be the Week 36 measurements for the placebo subjects. Repeated or unscheduled assessments will not be summarized in the tables unless they are part of a baseline derivation or a worst value summary. Visit windowing will not be used in this study.

3.1 Determination of Sample Size

Based on previously reported longitudinal studies of MUNIX(4) sum score in ALS patients (Neuwirth et al., 2015), which is the sum of the respective MUNIX values for the Abductor Digiti Minimi (ADM), Abductor Pollicis Brevis (APB), Biceps Brachii (BB), and Tibialis Anterior (TA), with baseline summed values indexed to 100%, and changes from baseline calculated as the percent change from the baseline index of 100% for the summed values; it is assumed the common standard deviation for the MUNIX(4) sum score is 19.8% with a mean placebo deterioration of 38.4% (e.g., 61.6% of the baseline index value) at Week 36. The active treatment mean group deterioration is estimated at 19.2% (e.g., 80.8% of the baseline index value) at Week 36.

Accordingly, sample size calculations were based on the following criteria:

Mean difference between the MUNIX(4) sum score indexed value in the change from baseline between active and placebo of 19.2% at Week 36;

a) common standard deviation of 19.8%;

b) alpha of 0.05;

c) power of 80% and

d) 12.5% dropout rate.

At a 1:1 (CNM-Au8 30 mg: Placebo) allocation, 80% power and 5% statistical significance rate, an estimated 36 evaluable patients will be required for this study. With a 12.5% estimated non-evaluable rate, it is planned to assign at least 42 patients (21 active:21 placebo) to randomized treatment.

3.2 Handling of Missing Data

Incomplete dates needed for derivations will be imputed as follows:

If the incomplete date is a start/onset date:

(1) if the month and year are present, then the first day of the month will be used for day.

(2) if only the year is present, then the first day of January will be used for month and day.

If the incomplete date is an end date:

(1) if the month and year are present, then the last day of the month will be used for day.
(2) if only the year is present, then the last day of December will be used for month and day.

If the reported year is the same as the informed consent year and the imputed date is invalid using the rules above, then the informed consent date will be used.

Missing dates will not be imputed.

Missing data for the primary and secondary endpoints (MUNIX(4) sum score and %predicted FVC) will be handled using a Mixed-Effect Model Repeated Measure (MMRM) model (See Section 3.4.1), which imputes that missing data under the assumption that it would have behaved similar as a subject with a similar trajectory who did not dropout which targets the hypothetical estimand (the effect of treatment if the subject is able to complete

the study). As a sensitivity analyses, a tipping point analysis on the primary and secondary endpoints (MUNIX(4) sum score and %predicted FVC) will be conducted using multiple imputation methods.

Missing data for the Patient Global Impression (PGI) and Clinician's Global Impression (CGI) scores will be handled using a MMRM model as described above for the primary endpoint. As a sensitivity analysis, a Chi-square test will be performed on the categorical data.

Responder endpoints will be imputed using non-response imputation (NRI). If a subject is missing data to determine response, then they will be imputed as a non-responder. The exception to this will be the Health Outcome response. The Health Outcome response will use last observation carried forward (LOCF).

Data will be summarized at the visits reported in the database. Visit windows will not be applied.

3.3 Subject Characteristics

3.3.1 Disposition

Subject disposition will be summarized by treatment on all subjects. The number of screen failures and subjects randomized to treatment, included in each population, completing the study and discontinuing from the study as well as the reasons for discontinuation will be presented. Reasons for exclusions from the analysis populations will also be summarized. Disposition will be presented in data listings.

3.3.2 Protocol Deviations

All protocol deviations will be presented in a data listing.

3.3.3 Demographics and Baseline Characteristics

Demographic data such as age, gender, race, ethnicity, height, weight, body mass index (BMI), and childbearing potential, will be summarized by treatment and overall, as well as presented in data listings. Demographic summaries will be presented for ITT, Partial Analysis, Per Protocol, and Safety populations.

3.3.4 ALS History/Status and Medical History

ALS and medical history are collected during pre-exposure.

The age at diagnosis of ALS, the number of years from symptom onset to consent (defined defined using the symptom onset date and informed consent dates), the number of years since diagnosis of ALS (defined using the diagnosis date and informed consent dates), ALS site of onset (bulbar, limb (combined), other), ALS Ajawi diagnostic category (possible, probable, definite), delta Functional Status (delta-FS) score (48 minus the ALSFRS-R score at screening/(months since symptom onset date at baseline), European Network for the Cure of ALS (ENCALS) risk score at baseline, %predicted FVC at baseline, and whether currently treating with stable riluzole dose, will be summarized on the ITT, Partial Analysis, Per Protocol, and Safety populations.

ALS Ajawi diagnostic category and ENCALS risk score will be provided as external data from the sponsor and are not collected on the case report forms (CRF).

Medical history will be presented in a data listing.

3.3.5 <u>Prior and Concomitant Medications</u>

Medications taken before and during the study will be coded using the World Health Organization (WHO) Drug Dictionary B3 September 1, 2020. Incomplete dates will be imputed (See Section 3.2). If a medication started and ended before the first dose of study drug, then it is considered a prior medication. Medications that are ongoing at or started after the first dose of study drug are considered concomitant medications. If the start or end date of a medication is unknown, the medication will be summarized as concomitant. Prior and concomitant medications will be tabulated by treatment. All medications will be presented in a data listing.

3.4 Efficacy Analyses

The primary efficacy endpoint is the percent change from baseline in the MUNIX(4) sum score, which is the sum of the respective MUNIX values for the ADM, APB, BB, and TA at Week 36. The baseline summed values will be indexed to 100%. Changes from baseline will be calculated as the percent change from the baseline index of 100% for the summed values at subsequent visits.

The key secondary efficacy endpoints include absolute change from baseline in %predicted FVC, a measure of respiratory function and the absolute change from baseline in the MUNIX(4) sum score at Week 36.

Exploratory EMG endpoints of interest include:

- MUNIX(4) sum score responder analysis (all subjects with <= -15% and <= -25% decline from baseline by visit)
- MUNIX change from baseline by visit by unique muscle (e.g., ADM, APB, BB, TA)
- Split Hand Index (SH_{Index}) change from baseline by visit
- Neurophysiological Index (NP_{Index}) of the ADM change from baseline by visit
- MUSIX(4) sum score change from baseline by visit
- MScanFit MUNE of the APB change from baseline by visit.

Exploratory clinical endpoints include:

- ALSFRS-R and subscales change from baseline by visit
- Proportion of participants with a >=6-point decline in the ALSFRS-R total score by visit
- Delta-FS (time-adjusted rate of ALSFRS-R deterioration from symptom onset) change from baseline by visit
- Proportion of subjects with Composite disease progression by visit
- Overall Survival (time to death and time to death or tracheostomy)
- ALS Specific Quality of Life-Short Form (ALSSQOL-SF) and subscales change from baseline by visit
- Clinical and patient global impression changes from baseline by visit
- Proportion of subjects using Health economic outcome measures by visit
- Combined assessment of function and survival over treatment period
- Joint modeling of MUNE(4) sum score and death over treatment and open label periods

- Long term overall survival (time to death and time to death or tracheostomy over treatment and open label periods)
- Time to stage-up for King's and MiToS clinical staging criteria

All of these data will be collected at baseline, Week 12, 24, and 36 during the treatment period. During the open label period, the EMG data will be collected at Weeks 24 and 48, while all other data will be collected at Weeks 12, 24, 36, and 48.

An exploratory analysis on long term survival using matched historic controls or prediction models may be performed.

The primary analysis population for the treatment period will be the ITT population. All efficacy endpoints will be summarized on the Per Protocol and Partial Analysis populations as a supplementary analysis.

Statistical analyses will be conducted hierarchically for the primary and secondary endpoints in the order shown below:

- Percent Change in MUNIX(4) Sum Score
- Absolute Change in MUNIX(4) Sum Score
- Absolute Change in % predicted FVC

To adjust for multiplicity, a fixed-sequence method will be used to control the study-wise type 1 error of 0.05. The primary endpoint hypothesis will be tested first. If the hypothesis test is significant (p-value < 0.05), then the next hypothesis endpoint will be tested. If the hypothesis test is not significant (p-value > 0.05), then any further testing will be considered exploratory. Statistical analyses will be performed on summaries of the treatment period and will include the open label extension when specified in the following sections. Details of these analyses are provided in the subsequent sections. Otherwise, summaries of the open-label period will be descriptive with no statistical comparisons.

All efficacy parameters will be presented in data listings.

3.4.1 Primary Efficacy Endpoint

The primary efficacy endpoint is the percent mean change between active treatment and placebo from baseline at Week 36 for the MUNIX(4) sum score, which is the sum of the

respective MUNIX values for the ADM, APB, BB, and TA during the treatment period (i.e., through Week 36). The baseline summed values will be indexed to 100%. Changes from Baseline will be calculated as the percent change from the baseline index of 100% for the summed values at subsequent visits.

The primary hypothesis being tested is that the percent change from baseline in the MUNIX(4) sum score for CNM-Au8 is not equal to placebo (a 2-sided test).

H_0: $\mu_T - \mu_P = 0$

H_1: $\mu_T - \mu_P \neq 0$

where T is active treatment (CNM-Au8) and P is placebo.

The direction of interest for the alternate hypothesis is that the percent change from baseline in the primary endpoint is greater than that of placebo.

A restricted maximum likelihood-based repeated measures approach, using a mixed effect model for repeated measurement will be used to analyze the MUNIX(4) sum score at Week 36. The MMRM model will include treatment, visit, and treatment by visit interaction as fixed effects, as well as baseline MUNIX(4) sum score, and baseline ENCALS score as covariates. An unstructured covariance structure shared across treatment groups will be used to model the within subject errors. If convergence is not met using an unstructured covariance, then the corrected Akaike's Information Criteria (AICC) will be used to select the best covariance structure among first order autoregressive, compound symmetry, and Toeplitz. The Kenword-Roger approximation will be used to estimate denominator degrees of freedom and adjust standard errors. The least square mean difference along with the associated standard error and 95% confidence interval will be presented at Week 36. The MMRM analysis will be conducted at each of the preceding visits (Weeks 12 and 24) as well.

The observed and percent change from baseline values will be summarized by treatment at each post baseline visit during the treatment period. A figure of observed MUNIX(4) sum score values over time by treatment will also be generated. The open label period will be summarized in a separate table. Baseline values for subjects who were randomized to placebo will be the last non-missing measurements prior to the first dose of CNM-Au8. For most subjects this will be the Week 36 measurement of the treatment period.

3.4.1.1 Sensitivity Analyses of the Primary Endpoint

The MUNIX(4) sum score will be analyzed using multiple imputation with a tipping analysis to support the primary analysis. The Markov Chain Monte Carlo (MCMC) method will be used to impute the missing data within each treatment under the missing at random paradigm. One hundred imputed datasets will be generated. Each dataset will be analyzed using the analysis model specified above. The final estimate of treatment difference and inferences will be handled using PROC MIANALYZE. A shift parameter on CNM-Au8 will be applied in the imputation of missing data. This shift parameter will be increased/decreased in increments of 0.5 (increments can be increased or decreased as needed during modeling) which imputes unfavorable outcomes for the missing data in the active treatment group. The value of the shift parameter that results in a change in statistical outcome (i.e., p-value > 0.05) will be displayed in the table. The tipping point analysis will address the impact of the missing values and the robustness of the primary analysis model; therefore, it will only be performed if the primary analysis is statistically significant.

The observed case analysis will also be analyzed for the primary endpoint. The observed case analysis uses the actual data collected with no imputations. The observed treatment differences will be analyzed at each visit used an ANCOVA model with baseline MUNIX(4) sum score and baseline ENCALS score as covariates.

3.4.2 <u>Secondary Efficacy Endpoints</u>

3.4.2.1 Absolute change of the MUNIX(4) sum score

The absolute change from baseline between active treatment and placebo at Week 36 for the MUNIX(4) sum score will be summarized as a secondary endpoint. For each week, observed and change from baseline values will be summarized by treatment. Differences between treatments will be analyzed at each visit using the same MMRM model described above for the primary endpoint for the treatment period only.

The OLE data will be summarized separately using descriptive statistics.

3.4.2.2 %Predicted FVC

The key secondary endpoint is the mean change from baseline at Week 36 for the %predicted forced vital capacity. For each week, observed and change from baseline values will be summarized by treatment. Differences between treatments will be analyzed using the same MMRM model described above for the primary endpoint for the treatment period only but using baseline %predicted FVC and ENCALS score at baseline as covariates. Values obtained

are reported as the percent (%) predicted values. Treatment differences at each of the preceding weeks will be analyzed as well. A figure of the observed %predicted FVC value will be generated.

The OLE data will be summarized separately using descriptive statistics.

3.4.2.3 Sensitivity Analyses on Secondary Endpoints

The MUNIX (4) sum score and %predicted FVC will be analyzed using multiple imputation with a tipping analysis. The process described in Section 3.4.1.1 will be applied to each secondary parameter. The observed case analysis will also be analyzed for each secondary endpoint.

3.4.3 Exploratory Efficacy Endpoints

The exploratory analyses described below are descriptive. The type 1 error is not controlled under the study-wise alpha level of 0.05.

3.4.3.1 MUNIX(4) Sum Score Responder Analysis

The MUNIX(4) sum score responder will be defined as subjects whose percent change is greater than or equal to -15%. Non-response imputation will be used for missing data at each visit. Treatment differences will be analyzed using a Chi-square test at each week. The responder analysis will be conducted on subjects with a greater than or equal to -25% decrease in percent change from baseline also.

3.4.3.2 MUNIX by Unique Muscle (APB, ADM, BB, TA)

The values of APB, ADM, BB, and TA scores will be summarized using descriptive statistics for each treatment group. Each muscle group will be analyzed using the primary analysis model described in Section 3.4.1 with the baseline muscle value and ENCALS score as covariates.

3.4.3.3 Weighted MUNIX(4) Sum Score

The MUNIX(4) sum score will be weighted by the baseline muscle values for each post-baseline visit and then summarized along with the change from baseline. The weights will be calculated as: baseline APB/baseline MUNIX(4) sum score, baseline ADM/baseline MUNIX(4) sum score, baseline BB/baseline MUNIX(4) sum score, and baseline TA/baseline

MUNIX(4) sum score. These weights will then be multiplied to the respective muscle and the values summed to get the weighted sum as follows:

Weighted Sum: (APB*baseline APB/baseline MUNIX(4) sum score) +(ADM*baseline ADM/baseline MUNIX(4) sum score) + (BB*baseline BB/baseline MUNIX(4) sum score) + (TA*baseline TA/baseline MUNIX(4) sum score)

This computation will be done at baseline and each post-baseline visit to get the weighted sums for each week. The baseline, Weeks 12, 24, and 36 weighted sums will be summarized along with the change from baseline at each of the post-baseline weeks. Weeks 24 and 48 of the open label period will also be summarized. Treatment differences will be analyzed using the primary analysis model.

3.4.3.4 Split Hand Index

The Split Hand Index is derived from the reported Compound Muscle Action Potential (CMAP) Amplitude values (derived from the MUNIX research protocol) for each muscle described below as:

[First Dorsal Interosseous (FDI)*APB]/ADM.

Observed and change from baseline values will be summarized by treatment for each post baseline week, and treatment differences will be analyzed using the primary efficacy model using baseline split hand index and baseline ENCALS score as covariates for the treatment period.

The open label period will be summarized using descriptive statistics.

3.4.3.5 Neurophysiological Index

NP_{index} is derived from the reported electromyography ADM values (derived from the MUNIX research protocol) as:

[CMAP Amplitude (mv)/Distal Motor Latency (ms)]*FWave frequency (%).

The observed and change from baseline values will be summarized by treatment for each week. Treatment differences will be analyzed using the primary endpoint model using baseline NP_{index} and baseline ENCALS score as covariates for the treatment period.

Open label period data will be summarized using descriptive statistics.

3.4.3.6 MUSIX(4) sum score

The MUSIX(4) sum score will be derived by summing the MUSIX values of ADM, APB, BB and TA. Observed and percent change from baseline and change from baseline values will be presented by treatment for each week. Treatment differences during the treatment period will be analyzed using the primary endpoint model using baseline MUSIX(4) sum score and baseline ENCALS score as covariates; the open label period will be summarized separately using descriptive statistics.

3.4.3.7 MScanFit MUNE

The MScanFit MUNE score is reported on the CRF. The observed and percent change/change from baseline values will be tabulated by treatment for each week. The MScanFit MUNE score will also be analyzed using the primary endpoint model using baseline MUNE score and baseline ENCALS score as covariates for the treatment period. The open label period will be summarized using descriptive statistics.

3.4.3.8 ALS Functional Rating Scale-Revised

The ALSFRS-R will be conducted at screening, baseline, Weeks 12, 24, and 36 during the treatment period and baseline, Weeks 12, 24, 36, and 48 during the open label period. The questionnaire consists of 12 questions with responses scored from 0 to 4 with 0 indicating a worst progression of disability and 4 indicating normal function. The ALSFRS-R score is derived by summing the responses of the 12 questions and ranges from 0 to 48. A lower score indicates progression of disability. The observed and change from baseline values will be summarized for each visit. The 4 subscales of the ALSFRS-R, bulbar (question 1-3, fine motor (questions 4-6), gross motor (questions 7-9), respiratory (questions 10-12), will also be summarized for each visit.

The ALSFRS-R slope of decline (for the total score and for each subscale) will be analyzed using a mixed model with treatment, baseline ALSFRS-R score/subscale score, time (months from first symptom onset), and ENCALS score as factors, including interaction terms for treatment by time and treatment by baseline. Time will be treated as a random effect and an unstructured covariance structure will be assumed. The treatment difference in the overall slopes will be displayed. If the model does not converge, AICC will be used to assess the best fit (as described in Section 3.4.1).

The total score will be summarized in a figure over time for each treatment.

3.4.3.9 Delta-FS (Functional Status)

Delta-FS, the time-adjusted rate of ALSFRS-R deterioration is derived by subtracting the ALSFRS-R score from 48 (the non-affected normal score) and dividing by the duration of disease by months from symptom onset. The change in the rate of disease progression for active treatment and placebo will be summarized at each visit. Treatment differences will be compared using the mixed model specified for the ALSFRS-R slope of decline, with baseline delta-FS score instead of the ENCALS score as a covariate.

3.4.3.10 ALSFRS-R Decline Response Analysis

ALSFRS-R decline response will be defined as subjects who have a decline greater than or equal to 6 from baseline. Missing responses will be imputed using NRI. Frequencies and percentages will be summarized by treatment at each visit. The Chi-Square test will be used to analyze treatment differences.

3.4.3.11 ALS Disease Progression Composite Response Analysis

Frequencies and percentages of subjects who experience ALS disease progression will be summarized at each visit. ALS clinical composite disease progression will be defined as the occurrence of at least one of:

- death,
- tracheostomy,
- use of non-invasive ventilatory support,
- insertion of gastrostomy tube.

The use of non-invasive ventilatory support and/or placement of gastronomy tube will be derived from the Health Economics Questionnaire. Tracheostomy will be determined based on the Elective/Disease Progression Procedure log and confirmed by a value of 0 on Question 12 on the ALSFRS-R questionnaire.

The time to ALS clinical composite disease progression will be assessed using the Kaplan-Meier method. Time to event between treatments will be analyzed using a log rank test. The progression rate will be presented along with the Greenwood's 95% confidence interval (CI), medians, first quartile (Q1), and third quartile (Q3). Subjects who do not experience disease progression will be censored using the completion/discontinuation

date of the treatment period. If a subject is lost to follow-up or is missing this date, then the last known date of contact recorded during the treatment period in the database will be used. The Kaplan-Meier curve will be generated in support of the table.

3.4.3.12 King's Staging Time to Progression

The King's staging system is a 6 stage system to assess the anatomical spread of ALS based on the number of affected regions (bulbar, lower limb, upper limb) (Roche et al., 2012). The ALSFRS-R items that are used to derive the King's staging are:

- Item 1: Speech
- Item 2: Salivation
- Item 3: Swallowing
- Item 4: Handwriting
- Item 5A/B: Self-feeding
- Item 8: Walking
- Item 10: Dyspnoea
- Item 12: Respiratory insufficiency

The King's stages are:

- Stage 1: Functional involvement of 1 clinical region
- Stage 2: Functional involvement of 2 clinical regions
- Stage 3: Functional involvement of 3 clinical regions
- Stage 4A: Need for gastrostomy
- Stage 4B: Need for non-invasive ventilation
- Stage 5: Death

Functional involvement of a clinical region is derived as follows:

1. If the ALSFRS-R score for items 1, 2, or 3 decrease from the previous visit then the bulbar region is considered involved; hence 1 clinical region.

2. If the ALSFRS-R score for items 4 or 5A decrease from the previous visit then the upper limb region is considered involved.

3. If the ALSFRS-R score for item 8 decrease from the previous visit then the lower limb region is considered involved.

4. If the ALSFRS-R score for item 5B is non-missing then the subject has gastrostomy which would be considered Stage 4A.

5. If the ALSFRS-R score for item 10 is a 0 OR has an ALSRFRS-R item 12 score less than 4 then the subject has a need for ventilation which would be considered Stage 4B.

The stage at each week in the treatment and open label period will be derived and summarized using frequencies and percentages according to their randomized treatment. The time to first progression of King's Stage after dosing will be derived for each subject using the date of the ALSFRS-R questionnaire. Time to progression analysis and censoring for subjects who do not progress from baseline (from the treatment period) will be the same as described for the ALS clinical composite disease progression endpoint except that the open label period will be included. The survival curve will be presented also.

The time to first progression to each subsequent stage will also be analyzed.

3.4.3.13 ALS Milano-Torino Staging (MiToS) Time to Progression

The ALS Milano-Torino staging system is a 6 stage system to measure the loss of independent function in four key domains in the ALSFRS-R that involve loss of autonomy (Chiò et al., 2015). The four ALSFRS-R domains are:

- Movement: Item 8: Walking OR Item 6: Dressing/Hygiene,
- Swallowing: Item 3: Swallowing,
- Comunicating: Item 1: Speech AND Item 4: Handwriting,
- Breathing: Item 10: Dyspnea OR Item 12: Respiratory insufficiency.

The ALS-MiToS functional score assigned is a 0 if the ALSFRS-R Item score is Normal/None and 1 for any other ALSFRS-R Item score. The ALS-MiToS functional scores are then summed. The ALS-MiToS functional stages are:

- Stage 0: Functional score of 0 (no loss of independence on any domain)
- Stage 1: Functional score of 1 (loss of independence in one domain)
- Stage 2: Functional score of 2 (loss of independence in two domains)
- Stage 3: Functional score of 3 (loss of independence in three domains)
- Stage 4: Functional score of 4 (loss of independence in four domains)
- Stage 5: Death

The ALS-MiToS will be derived and summarized at each visit in both the treatment and open label period. The time to first progression of ALS-MiToS after dosing will be analyzed using the same model described for the ALS clinical composite disease progression. Censoring will include the open label period. The survival curve will be presented also.

The time to first progression to each subsequent stage will also be analyzed.

3.4.3.14 Combined Assessment of Function and Survival (CAFS) using ALSFRS-R

A combined assessment of function and survival (CAFS) analysis (Berry et al., 2013) will also be performed on the ALSFRS-R data for the treatment period only. This joint rank analysis will evaluate function while accounting for missing data due to deaths. The CAFS score is derived as follows:

Each subject is compared individually to all other subjects and is assigned a score of +1, 0 or -1. If both subjects are alive, then compare the ALSFRS-R slope of decline at the end of the treatment period (Week 36). If the subject has a smaller slope of decline, then assign a score of +1. If the subject has a larger slope of decline, then assign a score of -1. If the subject has the same slope of decline, then assign a 0. If the subject is alive and the other subject has died, then assign a score of +1. If the subject has died and the other subject lives, then assign a score of -1. If both subjects have died, then compare the death dates and assign a +1 if the subject's death date is after the other subject, assign a -1 if the subject's death date is before the other subject, or a 0 if the death dates are the same. If a subject discontinues the study, then the last observed ALSFRS-R slope of decline for both subjects is used (i.e., if the subject's last ALSFRS-R measurement is Week 12, then only compare to Week 12 for the other subject.). Once you have compared the subject to all other subjects, sum the scores to get the subject's CAFS score.

The CAFS scores will be summarized using descriptive statistics. Treatment differences will be analyzed using an analysis of covariance model with baseline ENCALS scores and treatment as covariates. ALS duration may also be added a covariate if an imbalance is notable at baseline.

3.4.3.15 Modified CAFS using MUNIX(4) Sum Score

The CAFS analysis described in Section 3.4.3.14 will also be performed using the percent change from baseline in MUNIX(4) sum score. The CAFS score is computed in the same manner but using the MUNIX(4) sum percent change instead of the ALSFRS-R slope of decline.

3.4.3.16 Overall Survival During Treatment Period

The proportion of subject deaths will be summarized by treatment. The time to death will be assessed using the Kaplan-Meier method. Time to event between treatments will be analyzed using a log rank test. The progression rate will be presented along with the Greenwood's 95% CI, medians, Q1, and Q3. Subjects without an event will be censored using the date of completion/discontinuation as recorded on the CRFs for the treatment period. If a subject is lost to follow-up or is missing this date, then the last known date of contact recorded during the treatment period in the database will be used. The survival curve will be presented also.

The proportion of subject deaths or tracheostomy will also be summarized in the same manner above.

3.4.3.17 Patient Global Impression Scale

The PGI severity will be performed at baseline, Weeks 12, 24, and 36 and, the PGI improvement will be performed at Weeks 12, 24, and 36 during the treatment period. During the open label period, the PGI Severity will be performed at baseline, Weeks 12, 24, 36, and 48, and the PGI change responses will be performed at all the post-baseline visits. Subjects will mark the response that best describes their symptoms at each visit and their improvement from baseline.

Frequencies and percentages will be used to summarize the responses for each visit by treatment. Additionally, the change from baseline will be summarized. The severity responses will be numbered sequentially: 0=Normal, 1=Mild, 2=Moderate, 3=Severe. The improvement responses will be numbered such that any positive or negative change will be evident: 3=Very much better, 2=Much better, 1=A little better, 0=No change, -1=A little worse, -2=Much worse, -3=Very much worse. Treatment differences at each visit will be analyzed using the MMRM model specified for the primary endpoint. As a sensitivity analysis, the categorical responses will also be analyzed using a Chi-square test.

3.4.3.18 Clinical Global Impression Scale

The CGI severity will be performed at baseline, Weeks 12, 24, and 36 and, the CGI improvement will be performed at Weeks 12, 24, and 36 during the treatment period. During the open label period, the CGI Severity will be performed at baseline, Weeks 12, 24, 36, and 48, and the CGI change responses will be performed at all the post-baseline visits. The severity scale is a measure of the subject against the clinician's patient population.

The results will be presented using frequencies and percentages by visit. Additionally, the change from baseline will be summarized. The severity responses will be numbered sequentially: 0=Normal, not at all, 1=Borderline ill, 2=Mildly ill, 3=Moderately ill, 4=Markedly ill, 5=Severely ill, 6=Among the most extremely ill patients. The improvement responses will be numbered such that any positive or negative change will be evident: 3=Very much improved, 2=Much improved, 1=Minimally improved, 0=No change, -1=Minimally worse, -2=Much worse, -3=Very much worse. Treatment differences at each visit will be analyzed using the MMRM model specified for the primary endpoint. As a sensitivity analysis, the categorical responses will also be analyzed using a Chi-square test.

3.4.3.19 Health Economic Outcomes Measures

Health Economic Outcomes Measures will be performed at baseline, Weeks 12, 24, and 36 during the treatment period and at baseline, Weeks 12, 24, 36, and 48 during the open label period. The proportion of subjects utilizing health economic outcome measures will be derived at each visit as a Yes (1) or No (0) response to the question proposed on the CRF. The number of subjects utilizing a resource will be summarized using frequencies and percentages. The treatment difference at each visit will be compared using a Chi-square test.

3.4.3.20 ALS Specific Quality of Life Short Form

ALS Specific Quality of Life Short Form (ALSSQOL-SF) will be performed at baseline, Weeks 12, 24, and 36 during the treatment period and baseline, Weeks 12, 24, 36, and 48 during the open label period. The form consists of one question regarding the subject's overall quality of life with responses ranging from 0 (Very Bad) to 10 (Excellent), and twenty specific questions regarding quality of life with responses ranging from 0 (Strongly Disagree) to 10 (Strongly Agree). A higher score indicates a better quality of life; therefore, responses to questions 1-7, 10, 14, and 15 should be transposed (10 – response). The questions can be grouped into 6 subscales: Negative Emotion (questions 10, 14, 15), Physical Functioning (questions 1, 2, 5, 6, 7), Bulbar Function (questions 3, 4), Interaction with People and the Environment (questions 8, 9, 11, 16), Religiosity (questions 12, 13), and Intimacy (questions 17, 18, 19, 20).

The total and subscale scores are derived by averaging the respective responses. The observed and change from baseline in the overall quality of life question, ALSSQOL-SF total score, and the 6 subscales will be summarized by treatment. Treatment differences for the total and subscale scores will be analyzed from baseline to Week 36 using the MMRM model

described for the primary analysis with the baseline ALSSQOL-SF total score and the baseline ENCALS score as covariates.

3.4.3.21 Overall Survival During Treatment and Open Label Period

The proportion of subject deaths and subject deaths or tracheostomy during the treatment and open label period will be summarized by the randomized treatment. Time to death will be analyzed using the log rank test as described for the overall survival during the treatment period. Censoring dates will include the date of completion/discontinuation during the open label period if applicable. The survival curve will be generated.

Time to death from randomization through the end of the open label period may also be compared to age-matched and disease severity-matched historical controls and/or prediction models (i.e., ENCALS prediction, site specific historic controls) to explore changes in the rate of death during the study versus anticipated rates.

3.4.3.22 Joint Model of MUNE(4) Sum Score and Time to Death

The MUNE(4) sum score and time to death will be analyzed using a joint model over the treatment and open label periods. The joint model will have 2 sub-models: the longitudinal sub-model for the MUNE(4) sum score and the time to event sub-model for the time to death. The joint model will be used to find the treatment effect of the MUNE(4) sum score adjusted for death.

The longitudinal sub-model will a linear mixed model including treatment, month, and treatment by month interaction as fixed effects, as well as baseline MUNIX(4) sum score, and baseline ENCALS score as covariates. An unstructured covariance structure shared across treatment groups will be used to model the within subject errors. If convergence is not met using an unstructured covariance, then the AICC will be used to select the best covariance structure among first order autoregressive, compound symmetry, and Toeplitz. The Kenword-Roger approximation will be used to estimate denominator degrees of freedom and adjust standard errors.

The time to event sub-model will be a Cox proportional hazards with a Weibull baseline hazard model with treatment as a factor. Subjects who do not have an event will be censored at their completion/discontinuation date or the last available date reported in the study.

The joint model will estimate both model parameters using the Weibull baseline hazard function. The link between functions is a single parameter that relates the increase in hazard of event for each unit increase in the longitudinal sub-model.

The difference in treatment effects on the MUNE(4) sum score from the longitudinal sub-model will be summarized along with the 95% CI and p-value.

3.4.4 Subgroup Analyses

Subgroup analyses on the primary and secondary endpoints as well as the ALSFRS-R slope of decline will be conducted on the ITT population only. Statistical tests will be performed using the MMRM models described in each section; however, the p-values are descriptive in nature and multiplicity issues are not addressed. Subgroups will include:

1) Awaji-Shima classification: Possible, Probable, Definite
2) ALS Onset Site: Bulbar, Limb, Other
3) delta-FS at baseline
   a) (< median, ≥ median)
   b) (<1.11, ≥1.11)
4) ENCALS Risk Score at baseline (< median, ≥ median)
5) ALSFRS-R score at baseline
   a) (< median, ≥ median)
   b) (≥ 40 vs < 40)
6) Time (years) from symptom onset to informed consent (< median, ≥ median)
7) Baseline Age (< median, ≥ median)
8) Gender (Male/Female)
9) Baseline MUNIX(4) sum score >=median , <median

Forest plots with estimates and 95% confidence intervals of the treatments will be presented with the exception of age and gender.

3.5 Pharmacokinetic and Pharmacodynamic Endpoints

3.5.1 Phamacokinetic (PK) Endpoints

Samples for the measurement of whole blood concentrations of gold (Au) will be collected before (pre-dose) administration of the investigational drug product during the Week 12, 24, and 36 visits of the treatment period. At the Week 36 visit, whole blood for PK will be taken at 1, 2, 4, and 6 hours after dosing as well. During the OLE period, blood concentrations will be collected before study drug administration at baseline (Week 36 of the treatment period), Week 24 and Week 48.

Au concentrations will be summarized using descriptive statistics for each treatment. PK parameters such as maximum concentration, time of maximum concentration, systemic clearance, and area under the curve over the 24-hour dosing interval will be calculated and summarized.

Summaries and analyses on these data will be described in a separate PK analysis plan.

3.5.2 Pharmacodynamic (PD) Endpoints

Plasma, serum, and urine samples will be collected before (pre-dose) administration of the investigational drug product during the Week 12, 24, and 36 visits of the treatment period for analyses of PD endpoints.

PD analyses will include targeted investigation of disease relevant biomarkers including plasma neurofilament light chain, urinary $p75^{ECD}$, and serum and/or plasma creatinine levels by study visit.

Untargeted global proteomic and metabolomic analyses will be conducted on plasma samples by study visit to investigate baseline predictors of clinical response and treatment differences between active and placebo.

Summaries and analyses on these data will be described in a separate PD analysis plan.

3.6 Safety Analyses

Safety summaries will be descriptive and performed on the Safety population. No statistical comparisons will be performed.

Safety assessments include extent of exposure, incidence of adverse events (AE), clinical laboratory results, physical examinations, vital signs, electrocardiogram results (ECG), Falls questionnaire, and Columbia Suicide Severity Rating Scale. All safety parameters will be presented in data listings.

3.6.1 Extent of Exposure

The duration of treatment will be summarized by treatment for the treatment period, open label extension period, and the combination of treatment and open label periods. Treatment duration for each will be derived as follows: Date of Last Dose – Date of First Dose +1.

For the treatment period, the date of last dose will be the recorded date of completion/discontinuation on the End of Treatment CRF.

For the open label extension period, the date of last dose will be the recorded date of completion/discontinuation on the End of Study CRF. The date of first dose is recorded on the CRF during the open label baseline visit or is assumed to be the day after the Week 36 visit.

For the overall duration of treatment, the date of last dose will be the maximum of the dates from the treatment and OLE periods. The date of first dose will be the date from the treatment period or for the placebo roll-over subjects, the OLE baseline visit .

Study duration for each period and overall will also be summarized and is defined as the total number of days in the study (date of last visit/participation – date of informed consent +1). The date of last visit for the treatment period will be the date of completion/discontinuation on the end of study CRF. For the OLE period, the date of last visit/participation date will be used. Informed consent will be recorded for each period separately. Similar to the treatment duration, the overall summary will compare the date of informed consent from the treatment period to the maximum date of last visit/participation from the treatment and OLE periods.

Compliance will also be summarized at each visit and overall by treatment for both periods. Drug accountability will be collected at Weeks 12, 24, and 36 of the treatment period and Weeks 12, 24, 36, and 48 of the open label period. Subjects are expected to consume 2 bottles per day. Therefore, the expected number of used bottles will be calculated for each subject based on the number of days between each visit (current visit date - previous visit date + 1). Compliance at each visit will be derived as follows: (Number of Used Bottles/ number of days between each visit) x 100. The overall compliance will be the total number of used bottles divided by the total number of expected used bottles multiplied by 100.

3.6.2 Adverse Events

Adverse events will be collected throughout the study and coded using MedDRA version 21.0. A treatment emergent adverse event (TEAE) is defined as any event that starts on or after the first dose of study drug.

An overall summary of adverse events will be tabulated for:

- the number of subjects with an adverse event
- the total number of adverse events
- the number of subjects with TEAEs

- the number of subjects with TEAEs related to study drug
- the number of subjects with deaths due to a TEAE
- the number of subjects with serious adverse events (SAE) that are treatment emergent
- the number of subjects with a TEAE leading to discontinuation of study drug

Treatment emergent adverse events will be summarized by system organ class and preferred term for each dose. Related TEAEs, SAEs, TEAEs leading to discontinuation, and TEAEs by severity (mild, moderate, severe) will also be summarized by system organ class and preferred term.

Subjects with more than one event in a system organ class will be counted once within the class and once for each preferred term. Subjects with more than one event with the same preferred term will be counted once, and if the summary is by severity, the subject's most severe event will be counted.

3.6.3 Clinical Laboratory Tests

Laboratory tests will be assessed at screening, baseline, and Weeks 12, 24, 36 for the treatment period and baseline, Weeks 12, 24, 36, 48 and Follow-up during the open label extension period. Tests with numeric results for hematology, chemistry, and urinalysis will be summarized using descriptive statistics (number of subjects, mean, SD, standard error of the mean (SEM), median, minimum, and maximum) for each visit by treatment. Tests with subjective results will be displayed in data listings only.

Table summaries will be presented for observed and change from baseline values. Values flagged as Low, Normal, Abnormal, or High using the laboratory reference ranges will be summarized using shift tables comparing the post-baseline values to baseline.

3.6.4 Physical Examinations and Brief Neurological Examinations

A physical and neurological examination will be performed at screening, baseline, and Weeks 12, 24, 36, and Follow-up of the treatment period. During the OLE period, examinations will be performed at baseline and Weeks 12, 24, 36, 48, and Follow-up. Clinically significant findings during a physical and neurological examinations will be reported as adverse events. Results from the physical and neurological examinations will be presented in a data listing.

3.6.5 Vital Signs

Vital signs will be measured at screening, baseline, and Weeks 12, 24, 36, and Follow-up during the treatment period and at baseline, Weeks 12, 24, 36, 48, and Follow-up during the OLE period. Observed and change from baseline values for systolic and diastolic blood pressure, pulse rate, respiration rate, temperature, and weight will be summarized using descriptive statistics (n, mean, SD, median, minimum, and maximum) for each visit by treatment.

3.6.6 Electrocardiogram

12-Lead ECG will be measured at screening, baseline, and Weeks 12, 24, 36 during the treatment period and at baseline, Week 24 and 48 during the OLE period. The baseline measurements will be performed in triplicate and the values averaged for summary. The worst response for the Investigator's interpretation of the ECG will be used in the baseline summary, and a shift table will be used to present the changes from baseline to each post baseline visit. Observed and change from baseline values for the numeric parameters will be summarized using descriptive statistics (n, mean, SD, median, minimum, and maximum) by treatment for each visit.

3.6.7 Columbia Suicide Severity Rating Scale

The C-SSRS will be given at screening, baseline, Weeks 12, 24, 36 and Follow-up during the treatment period and at baseline, Weeks 12, 24, 36, 48, and Follow-up during the OLE period. The post baseline assessments will be in relation to how they feel in comparison to their last visit. The results will be listed.

3.6.8 Falls Questionnaire

The Falls Questionnaire will be given at Weeks 12, 24, and 36 during the treatment period and Weeks 12, 24, 36, and 48 during the OLE period. The number of subjects who had at least one fall, the number of falls, and the activity the subject was engaged in at the time of the fall will be summarized. If a subject had multiple falls and the subject was engaged in the same activity, then the subject will be counted once for the activity. If a subject had multiple falls and the subject was engaged in different activities for each or some of the falls, then the subject will be counted for each unique activity.

4. Interim Analysis

No formal interim analysis will be performed. However, once all subjects have completed or discontinued from the treatment period, the database will be unblinded and the full efficacy and safety analyses will be performed on the data from the treatment period.

5. Tables, Listings, Figures

A table of contents for the tables, listings, and figures will be presented in a separate document as the list of summaries or numbering may change after finalization of this document. If additional summaries are added that are not described in this document, then this SAP will be amended, or an addendum will be created.

6. REFERENCES

Berry, J.D., Miller, R., Moore, D.H., Cudkowicz, M.E., van den Berg, L.H., Kerr, D.A., Dong, Y., Ingersoll, E.W., and Archibald, D. (2013). The Combined Assessment of Function and Survival (CAFS): a new endpoint for ALS clinical trials. Amyotroph. Lateral Scler. Front. Degener. 14, 162–168.

Chiò, A., Hammond, E.R., Mora, G., Bonito, V., and Filippini, G. (2015). Development and evaluation of a clinical staging system for amyotrophic lateral sclerosis. J Neurol Neurosurg Psychiatry 86, 38–44.

de Carvalho, M., Dengler, R., Eisen, A., England, J.D., Kaji, R., Kimura, J., Mills, K., Mitsumoto, H., Nodera, H., Shefner, J., et al. (2008). Electrodiagnostic criteria for diagnosis of ALS. Clin. Neurophysiol. Off. J. Int. Fed. Clin. Neurophysiol. 119, 497–503.

Neuwirth, C., Barkhaus, P.E., Burkhardt, C., Castro, J., Czell, D., de Carvalho, M., Nandedkar, S., St.lberg, E., and Weber, M. (2015). Tracking motor neuron loss in a set of six muscles in amyotrophic lateral sclerosis using the Motor Unit Number Index (MUNIX): a 15-month longitudinal multicentre trial. J. Neurol. Neurosurg. Psychiatry 86, 1172–1179.

Roche, J.C., Rojas-Garcia, R., Scott, K.M., Scotton, W., Ellis, C.E., Burman, R., Wijesekera, L., Turner, M.R., Leigh, P.N., Shaw, C.E., et al. (2012). A proposed staging system for amyotrophic lateral sclerosis. Brain 135, 847–852.
