# Supplementary material for: Efficacy and safety of CNM-Au8 in amyotrophic lateral sclerosis (RESCUE-ALS study): a phase 2, randomised, double-blind, placebo-controlled trial and open label extension
Source: eClinicalMedicine. 2023 Jun 8;60:102036. doi: 10.1016/j.eclinm.2023.102036 (PMC10314176; doi:10.1016/j.eclinm.2023.102036)
Supplement: Supplementary material_Study Protocol [file mmc3.pdf]

`

CLINICAL STUDY PROTOCOL

RESCUE-ALS:

A Phase 2, **R**andomised, Double-Blind, Placebo-Controlled Study in **E**arly **S**ymptomatic Amyotrophic Lateral Sclerosis Patients on Stable Background Therapy to Assess Bioenergetic **C**atalysis with CNM-A**u**8 to Slow Diseas**e** Progression in **ALS**

U.S. IND Number:	142,774
Protocol Number:	CNMAu8.205
Clinical Phase:	Phase 2
Investigational Drug:	CNM-Au8
Protocol Version No.:	7.0 (Amendment 6.0)
Current Protocol Version Date:	04-October-2021

CONFIDENTIALITY STATEMENT

The information contained in this document and all information provided to you related to CNM-Au8 (“Study Drug”) or (“Investigational Product”) are the confidential and proprietary information of Clene Australia Pty Ltd. (Sponsor) and Clene Nanomedicine, Inc. and except as may be required by federal, state or local laws or regulations, may not be disclosed to others without prior written permission of the Sponsor. The Principal Investigator may, however, disclose such information to supervised individuals working on the Study Drug, provided such individuals agree to maintain the confidentiality of such information.

PROTOCOL APPROVAL PAGE

**Study Title:**	**RESCUE-ALS**: A Phase 2, Randomised, Double-Blind, Placebo-Controlled Study in Early Symptomatic Amyotrophic Lateral Sclerosis Patients on Stable Background Therapy to Assess Bioenergetic Catalysis with CNM-Au8 to Slow Disease Progression in ALS
**Protocol Number:**	CNMAu8.205
**Current Protocol Date:**	04-October-2021
**Sponsor Name and Address:**	Clene Australia Pty Ltd.<br>C/- Prime Accounting & Business Advisory Pty Ltd.<br>Floor 19, HWT Tower, 40 City Road<br>SOUTHBANK, VIC 3006<br>Australia<br><br>A subsidiary of:<br>Clene Nanomedicine, Inc.<br>6550 South Millrock Drive, Suite G50<br>Salt Lake City, UT 84121<br>United States

I, the undersigned, have read and approve this protocol and agree on its content. It is confirmed that the information and guidance given in this protocol complies with scientific principles, the guidelines of Good Clinical Practices, the Declaration of Helsinki in the latest relevant version, and the applicable legal and regulatory requirements.

Sponsor Signature:

DocuSigned by:
Robert Glanzman
Signer Name: Robert Glanzman
Signing Reason: I approve this document
Signing Time: 10/4/2021 | 3:39:52 PM PDT
D169CA245CAF4E74982A7D386FF9400C

10/4/2021

Sponsor's Medical Representative
Robert Glanzman, MD FAAN

Date

INVESTIGATOR PROTOCOL AGREEMENT

**Protocol Title**:	RESCUE-ALS: A Phase 2, Randomised, Double-Blind, Placebo-Controlled Study in Early Symptomatic Amyotrophic Lateral Sclerosis Patients on Stable Background Therapy to Assess Bioenergetic Catalysis with CNM-Au8 to Slow Disease Progression in ALS
**Protocol Number**:	CNMAu8.205
**Protocol Date:**	04-October-2021
**Version**:	7.0 (Amendment 6.0)

By my signature, I:

- Confirm that my staff and I have carefully read and understand this protocol and the Investigator's Brochure (IB) and are thoroughly familiar with the appropriate use of the investigational drug described herein.
- Agree to comply with the conduct and terms of the study specified herein and with any other study procedures provided by the Sponsor, Clene Nanomedicine, Inc. (herein referred to as "Clene").
- Agree to comply with US Food and Drug Administration (FDA), Health Canada, and Australian Therapeutic Goods Administration (TGA) regulations, as applicable; the International Conference on Harmonization (ICH) GCP guidelines, the Declaration of Helsinki, and all applicable rules, regulations, and federal, state, and local laws relating to the conduct of clinical studies and the protection of human subjects.
- Agree not to implement changes to the protocol without agreement from the Sponsor and prior written approval (where required) from the Institutional Review Board (IRB) or Human Research Ethics Committee (HREC), except when necessary to eliminate an immediate hazard to the subjects.
- Agree to onsite monitoring of the case report forms (CRFs) and source documents by Clene or designee and to audit by Clene or designee and appropriate regulatory authorities, including, but not limited to, the FDA and IRB/HREC inspectors.
- Agree to supervise the conduct of the study and maintain responsibility for training and supervising all personnel who have delegated responsibilities under my leadership. The protocol and other important study materials will be available to study staff throughout the conduct of the study.

**Investigator's Signature**	**Date**

Print Name

PROTOCOL SYNOPSIS

Category	Description
**Sponsor**	Clene Australia Pty Ltd., a subsidiary of Clene Nanomedicine, Inc.
**Investigational Drug Product**	CNM-Au8
**Name of Active Ingredient**	Faceted clean-surfaced Au nanocrystals
**Phase of Development**	Phase 2
**Study Title**	A Phase 2, **R**andomised, Double-Blind, Placebo-Controlled Study in **E**arly **S**ymptomatic Amyotrophic Lateral Sclerosis Patients on Stable Background Therapy to Assess Bioenergetic **C**atalysis with CNM-A**u**8 to Slow Diseas**e** Progression in **ALS**<br>**(RESCUE-ALS)**
**Study Center(s)**	Two study sites in Australia.<br>Additional centres in Australia and/or the United States may be added at the discretion of the Sponsor based on patient recruitment rates.
**Chief Investigator(s)**	A/Professor Parvathi Menon PhD<br>Consultant Neurologist, Associate Professor in the Faculty of Medicine<br>University of Sydney<br><br>Brain and Nerve Research Centre<br>Concord Repatriation General Hospital<br>Building 20, Hospital Road<br>Concord NSW 2139<br>Australia<br>Phone: +61 (2) 9767 8423<br>Email: parmenon2010@gmail.com<br><br>Dr. William Huynh<br>Consultant Neurologist<br>Brain and Mind Centre<br>94 Mallet Street<br>Camperdown NSW 2050<br>Phone: +61 (2) 9114 4250<br>Email: william.huynh@sydney.edu.au
**Study Objectives**	To assess the efficacy, safety, and PK/PD effects of CNM-Au8 as a disease-modifying agent for the treatment of ALS by utilizing electrophysiological measures to detect preservation of motor neuron function.<br>• Efficacy will be assessed as the difference in disease progression from baseline reflected by changes in motor neuron loss measured by electromyography (e.g., MUNIX, MUSIX, Split Hand Index, Neurophysiology Index, MScanFit), change in ALS Functional Rating

Category	Description
	Score-Revised (ALSFRS-R) score, the mean rate of change (ΔFS) of the ALSFRS-R from symptom onset, survival status, respiratory functions, and composite disease progression.<br>• Safety will be assessed up through the frequency of treatment emergent adverse events, serious adverse events, discontinuations due to adverse events, Falls Questionnaire, and the Columbia Suicide Severity Rating Scale (C-SSRS).
**Overall Study Design and Plan**	This is a multi-centre randomised, double-blind, parallel group, placebo-controlled study of the efficacy, safety, pharmacokinetics, and pharmacodynamics of CNM-Au8 in patients who are newly symptomatic within 24-months of Screening and with a clinically *probable* or *possible* or *definite* ALS diagnosis per the Awaji-Shima criteria (de Carvalho et al., 2008).<br>Patients may be screened over up to a 6-week period. Patients who meet the inclusion criteria and none of the exclusionary criteria will be enrolled into the clinical study.<br>Patients will be randomised 1:1 into one of two groups: either active treatment with CNM-Au8 30 mg or Placebo.<br>All patients will receive their randomised oral treatment daily over thirty-six (36) consecutive weeks during the Treatment Period.<br>There will be up to four study periods:<br>**1.** Up to a six (6) week screening period (Screening Period);<br>**2.** A thirty-six (36) week blinded randomised treatment period (Treatment Period);<br>**3.** A forty-eight (48) week optional open-label extension period (Open-Label Period), which may be extended by 12-week increments until discontinued by the Sponsor;<br>**4.** A four (4) week safety follow-up period following completion of either the Treatment or Open-Label period or in the case of Early Termination (Safety Follow-Up Period).<br>Per protocol all patients will receive their blinded and randomised oral treatment daily over at least 36 consecutive weeks during the Treatment Period.<br>For those patients not transitioning into the optional OLE period, patients will complete a safety follow-up visit 4-weeks following study drug discontinuation.<br>An independent DSMB will be responsible for monitoring the safety of the study on a trimester basis and *ad hoc* at the request of the DSMB or the Sponsor (e.g., in the event of unexpected SAEs) to review data throughout the Treatment Period and OLE period. The DSMB may make recommendations on the conduct of the study, including study termination.<br>Appropriate procedures will be detailed in a DSMB Charter.

Category	Description
**Estimated Study Duration**	The estimated study duration for the randomized placebo controlled period of the trial will be approximately 72-weeks from the first patient-first visit to the last patient-last visit. This duration assumes a 27-week recruitment period (e.g., 21-weeks, average of 1 enrolled patient per site per week (n=42) based on the site-specific projections from the recently enrolled TEALS study, plus a 6-week projected Christmas and New Year holiday recruitment pause), up to a 6-week screening period, the 36-week Treatment Period, and a 4-week safety follow-up period for those patients not transitioning to the optional OLE period of the trial. It is currently planned that the OLE period will run for up to an additional 48 weeks beyond the 36-week placebo controlled portion of the trial, which may be extended for additional 12-week increments until discontinued by the Sponsor.
**Number of Patients**	Approximately 42 randomised patients (1:1); 36 evaluable (assuming a ~14% drop-out rate):<br>• 30 mg CNM-Au8 (n=21)<br>• Placebo (n=21)
**Inclusion Criteria**	The patients to be enrolled in this study must meet the following inclusion criteria:<br>1. Able to understand and give written informed consent.<br>2. Male or female patients aged 30 years or greater (inclusive) and less than 80 years of age at the time of Screening.<br>3. Patients whose conditions are defined as *possible* or *probable* or *definite* ALS per the diagnostic criteria by Awaji-Shima criteria as determined by a neurologist sub-specialising in ALS (e.g., the Principal Investigator by study site).<br>4. For patients taking riluzole, stable dosing of riluzole over the prior 30-days from Screening.<br>5. At the time of Screening either disease duration less than or equal to 24-months from symptom onset, or disease duration less than or equal to 12-months from diagnosis.<br>6. Forced vital capacity (FVC) ≥ 60% of predicted value as adjusted for gender, height, and age at the Screening Visit.<br>7. Patient who has established care with a neurologist at one of the specialised ALS clinics involved in the study and will maintain this clinical care throughout the study. If a patient is referred from a third party (neurologist or a State based ALS organisation) they must be willing to transfer care to the neurologist participating in the study.<br><br>Following completion of the 36-week randomized placebo controlled treatment period, interested participants must meet the following inclusion criteria to enroll in the open-label extension:<br>1. Participants must have completed the randomized placebo controlled Treatment Period without compliance issues

Category	Description
	2. Able to understand and give written informed consent to participant in the open-label extension.<br>3. If referred from a third party (neurologist or a State based ALS organisation), participant agrees to maintain transfer of care to a neurologist participating in the study.
**Exclusion Criteria**	1. Patients will be excluded from the study if they meet any of the following criteria:<br>2. At Screening patients who utilize, or in the Investigator's judgment will be imminently dependent upon:<br>a. Non-invasive ventilation $\geq$22 hours per day, or<br>b. Tracheostomy<br>*Note*: If the patient requires non-invasive ventilation post-randomisation, they will be allowed to continue in the study.<br>3. Patients with Familial ALS (e.g., 2 or more family members with ALS or motor neuron disease)<br>4. Patients with a history of carpal tunnel syndrome, polyneuropathy, or in the investigators judgement diseases that could induce polyneuropathy and interfere with electromyography (EMG) recordings.<br>5. Patients with too severe atrophy of the Abductor Digiti Minimi (ADM), Abductor Pollicis Brevis (APB), Biceps Brachii (BB), or Tibialis Anterior (TA) muscles in the least clinically affected hand and leg, respectively, to allow for reliable EMG recordings.<br>6. Patient with a history of significant other major medical conditions based on the Investigator's judgment.<br>7. Based on the investigator's judgment, patients who may have difficulty complying with the protocol and/or any study procedures.<br>8. Patient with clinically significant abnormalities in haematology, blood chemistry, ECG, or physical examination not resolved by the Baseline visit which according to Investigator can interfere with study participation.<br>9. Patients with clinically significant hepatic or renal dysfunction or clinical laboratory findings that would limit the interpretability of change in liver or kidney function, or those with low platelet counts ($< 150 \times 10^9$ per liter) or eosinophilia (absolute eosinophil count of $\geq$ 500 eosinophils per microliter) at Screening.<br>10. Patient participating in any other investigational drug trial or using investigational drug (within 12 weeks prior to screening and thereafter).<br>11. Females who are pregnant or nursing or who plan to get pregnant during the course of this clinical trial or within 6 months of the end of this trial.<br>12. Females of child-bearing potential, or men, who are unwilling or unable to use accepted methods of birth control.<br>13. Active inflammatory condition or autoimmune disorder.<br>14. Positive screen for drugs of abuse.

Category	Description
	15. History of gold allergy.<br>16. Patient is considered a suicide risk in the opinion of the Investigator, has previously made a suicide attempt, or is currently demonstrating active suicidal ideation. Subjects with intermittent passive suicidal ideation are not necessarily excluded based on the assessment of the Investigator.<br><br>Following completion of the 36-week randomized placebo controlled treatment period, interested participants will be excluded from participating in the open-label extension phase if they meet any of the following criteria:<br><br>1. Lack of treatment compliance during the randomized placebo controlled Treatment Period.<br>2. Positive pregnancy test at the Week 36 visit, or, females who plan to get pregnant during the course of this extension or within 6 months of the end of this extension.<br>3. Based on the investigator's judgment, patients who may have difficulty complying with the protocol and/or any study procedures.<br>4. Patient with clinically significant abnormalities in haematology, blood chemistry, ECG, or physical examination identified during the W36 visit which according to Investigator may interfere with continued participation.<br>5. Patients with clinically significant hepatic or renal dysfunction or clinical laboratory findings that would limit the interpretability of change in liver or kidney function, or those with low platelet counts ($< 150 \times 10^9$ per liter) or eosinophilia (absolute eosinophil count of $\geq$ 500 eosinophils per microliter) at the Week 36 visit.<br>6. Patient is considered a suicide risk in the opinion of the Investigator, has previously made a suicide attempt, or is currently demonstrating active suicidal ideation. Subjects with intermittent passive suicidal ideation are not necessarily excluded based on the assessment of the Investigator.
**Concomitant Medications**	Disease-specific medications (e.g., riluzole) are allowed per the inclusion criteria.<br><br>Except for disease specific medications (e.g., riluzole), acetaminophen (paracetamol), ibuprofen, naproxen, and 2nd generation antihistamines (including fexofenadine, loratadine, and cetirizine); patients may not take any new prescription medications, OTC, or dietary supplements from 14-days prior to Baseline through the safety follow-up (including the optional OLE period) unless used to manage a treatment emergent adverse event, in which case the patient should report to his/her Study Physician as soon as possible, the occurrence of the adverse event and the medication(s) they are taking to treat the adverse event.<br><br>The Investigator will make every effort to contact the Medical Monitor or Sponsor representative prior to administration of a new concomitant therapy

Category	Description
	(prescription or OTC) after randomisation, unless the concomitant therapy is needed immediately for patient safety.
**Duration of Treatment**	Patients will receive 36 weeks of consecutive daily dosing per protocol. Following completion of the 36-week randomized, placebo controlled treatment period, participants may choose to participate in an open-label extension (OLE) period for up to an additional 48 weeks, which may be extended for additional 12-week increments until discontinued by the Sponsor.
**Test Product, Dose, and Mode of Administration**	CNM-Au8 is an aqueous suspension of clean surfaced faceted nanocrystals consisting of gold atoms self-organized into crystals of various geometrical shapes (hexagonal bi-pyramid, pentagonal bi-pyramid, tetrahedron, decahedron, planar spheroids).<br>Highly pure elemental Au nanocrystals are suspended in USP purified deionized water buffered with 0.546 mg/mL (6.5 mM) sodium bicarbonate ($NaHCO_3$) nominally concentrated up to 0.5 mg/L (500 ppm).<br>Patients will orally receive 30 mg of CNM-Au8, or placebo, once daily. Those choosing to participate in the OLE period will orally receive 30 mg of CNM-Au8, once daily.<br>CNM-Au8 or matching placebo will be administered orally in volumes of 60 mL from single-dose HDPE containers.
**Primary Efficacy Endpoint**	**Electromyography Measures of Disease Progression**<br>Mean change in the average difference between active treatment and placebo from Baseline through Week 36 for the MUNIXscore(4), which is the sum of the respective MUNIX values for the Abductor Digiti Minimi (ADM), Abductor Pollicis Brevis (APB), Biceps Brachii (BB), and Tibialis Anterior (TA). The average baseline summed values will be indexed to 100%. Changes will be calculated as the percent change from the Baseline index of 100%.
**Secondary Efficacy Endpoints**	• Mean change of the average difference between active treatment and placebo from Baseline to Week 36 for respiratory function as measured by forced vital capacity (FVC).<br>• Mean absolute change of the average difference between active treatment and placebo from Baseline through Week 36 for the MUNIXscore(4), which is the sum of the respective MUNIX values for the Abductor Digiti Minimi (ADM), Abductor Pollicis Brevis (APB), Biceps Brachii (BB), and Tibialis Anterior (TA).
**Exploratory Efficacy Endpoints**	The hierarchical assessment of exploratory endpoints will be detailed in the statistical analysis plan. Exploratory endpoints include both electromyography and functional clinical endpoints, including:

Category	Description
	• Mean change in the average difference between active treatment and placebo from Baseline through Week 36 (overall difference at all time points) as measured by MScanFit MUNE (Jacobsen et al., 2017) of the APB. The baseline average will be indexed to 100%, and changes at Week 12 and Week 36 will be calculated as the percent change from the Baseline index of 100%.<br>• Mean change in the average difference between active treatment and placebo from Baseline through Week 36 for the MUSIXscore(4) (Neuwirth et al., 2015), which is the mean of the respective MUSIX values for the ADM, APB, BB, and TA. The average baseline mean values will be indexed to 100%. Changes will be calculated as the percent change from the Baseline index of 100%.<br>• Mean change in the average difference between active treatment and placebo from Baseline through Week 36 for the Neurophysiological Index (NPI) of the ADM (Cheah et al., 2011), defined as the ulnar nerve $(ADM^{CMAP\ Peak\ Amplitude} / ADM^{Distal\ Motor\ Latency}) * ADM^{F\text{-}Wave\ (\%)}$.<br>• Mean change in the average difference between active treatment and placebo from Baseline through Week 36 for the Split Hand Index (SI) (Menon et al., 2013), defined as the first dorsal interosseous $(FDI)^{Peak\ CMAP\ Amplitude} * APB^{Peak\ CMAP\ Amplitude} / ADM^{Peak\ CMAP\ Amplitude}$.<br>• Mean change between active treatment and placebo in the proportion of patients experiencing a $\geq$ 6-point decline in the ALSFRS-R between active treatment and placebo from Baseline to Week 36.<br>• Mean change in average ALSFRS-R score from Baseline to Week 36.<br>• Mean change in slope of the decline of the ALSFRS-R from Baseline to Week 36.<br>• Mean change between active treatment and placebo for the Combined Assessment of Function and Survival (CAFS), a joint-rank analysis of function (ALSFRS-R) and overall survival from Baseline to Week 36.<br>• Mean change between active treatment and placebo from Baseline to Week 36 in the proportion of patients experiencing ALS clinical composite disease progression defined as the occurrence of death, tracheostomy, use of non-invasive ventilatory respiratory support, insertion of a gastrostomy tube, or a 6-point drop in the ALSFRS-R score.<br>• Mean change in rate of disease progression defined as the average change in the ΔFS score ([Max ALSFRS-R minus current ALSFRS-R score]/symptom duration in months) from Baseline to Week 36.<br>• Mean change in average difference between active treatment and placebo from Baseline to Week 36 for:<br>○ ALSSQOL-Short Form questionnaire (ALSSQOL-SF)

<table>
<tr><th>Category</th><th>Description</th></tr>
<tr><td></td><td>
Clinician’s Global Impression (CGI)
Patient’s Global Impression (PGI)
Difference in the proportion of patients utilizing health economic outcome measures from Baseline to Week 36.
</td></tr>
<tr><td>Open-Label Extension Endpoints</td><td>The primary endpoint for the OLE is safety. Safety endpoints include incidence of treatment-emergent AEs, drug-related AEs, deaths, SAEs, and AEs leading to discontinuation from the study. Changes from baseline (Week 36 of the placebo controlled phase) in clinical laboratory results, physical examination findings, vital signs, ECGs, and C-SSRS will be summarized descriptively by group and timepoint.

The primary efficacy endpoint for the OLE is the Electromyography Measures of Disease Progression defined as:
Mean change in the average difference between active treatment and placebo from Baseline through End of Study for the MUNIXscore(4), which is the sum of the respective MUNIX values for the Abductor Digiti Minimi (ADM), Abductor Pollicis Brevis (APB), Biceps Brachii (BB), and Tibialis Anterior (TA).
Other secondary and exploratory endpoints will be described below in Section 6.</td></tr>
<tr><td>Pharmacodynamic (PD) Endpoints</td><td>Measures of Blood and Urine Biomarkers

Blood, plasma, and urine will be collected at Baseline and at the Week 12, Week 24, and Week 36 visits and stored for subsequent pharmacodynamic analyses. During the OLE period, blood and urine for PD analyses will be collected at the Week 24 and every 24 weeks thereafter as long as the period is ongoing. The PD analyses may include blood metabolomic markers (e.g., redox coenzymes: NAD+, NADH, NADP+, NADPH; energetic coenzymes: ATP, ADP, AMP and antioxidants: GSSG and GSH), urinary neurotrophin receptor $P75^{ECD}$ levels, and serum neurofilament light chain (NfL).

PD analyses will be specified in a separate PD analysis plan to be completed prior to database lock.</td></tr>
<tr><td>Pharmacokinetic (PK) Endpoints</td><td>A sample for the measurement of whole blood concentrations of Au will be collected before (pre-dose) administration of CNM-Au8 at Week 12 and Week 24. At the Week 36 Visit, samples for the measurement of whole blood concentrations of Au will be collected before (pre-dose) and at 1, 2, 4, and 6 hours, after administration of the last dose of the investigational drug product at the site. The study site will contact the patient and document the time at which the patient administered his/her prior day’s dose of study drug in order to impute a 24-hour trough value.

The Week 36 data will be used to estimate an apparent C_{max} and T_{max} and area under the curve (AUC) over the 24-hour dosing interval [$AUC_{(0-24)}$].</td></tr>
</table>

Category	Description
	During the open-label extension phase, blood and urine for PK analyses will be collected at the Week 24 and every 24 weeks thereafter as long as the period is ongoing.<br><br>PK data will be used to construct a composite whole blood concentration-time profile for the Q12 week visits during the randomised treatment period.
**Statistical Methods**	The primary endpoint, the mean change in the MUNIXscore(4) summed value will be analysed using Mixed Model for Repeated Measures (MMRM) with treatment (Week 12, Week 24, and Week 36) as fixed effects and Baseline ΔFS, Baseline ALSFRS-R scores, and bulbar vs. limb onset as covariates. Estimates of least-square means, standard errors and 95% confidence intervals will be presented by treatment group. For the active treatment versus placebo comparisons, the least square mean difference, the standard error of the difference, and the 95% confidence interval of the difference will be presented.<br><br>Sample size calculations were based on the following criteria:<br>(i) Mean difference between the MUNIX(4)score indexed value in the change from baseline between active and placebo of 19.2% at Week 36;<br>(ii) common standard deviation (SD) of 19.8%;<br>(iii) α value of 0.05;<br>(iv) β power of 0.8; and<br>(v) 12.5% dropout rate.<br><br>Based on previously reported longitudinal studies of MUNIXscore(4) in ALS patients (Neuwirth et al., 2015); it is assumed the common standard deviation for the MUNIXscore(4) is 19.8% with a placebo deterioration of 38.4% (e.g., 61.6% of the baseline index value) at Week 36. The active treatment group deterioration is estimated at 19.2% (e.g., 80.8% of the baseline index value) at Week 36.<br><br>At a 1:1 (CNM-Au8 30 mg: Placebo) allocation, 80% power and 5% statistical significance rate, an estimated 36 evaluable patients will be required for this study. With a 12.5% estimated non-evaluable rate, it is planned to assign 42 patients (21 active:21 placebo) to randomised treatment.<br><br>Safety endpoints include incidence of treatment-emergent AEs, drug-related AEs, deaths, SAEs, and AEs leading to discontinuation from the study. Changes from baseline in clinical laboratory results and vital signs will be summarized by treatment group and time point. Treatment-emergent adverse events will be coded using the Medical Dictionary for Regulatory Activities and will be tabulated in incidence tables by System Organ Class (SOC) and Preferred Term (PT). Treatment-emergent adverse events will be summarised by SOC, PT, and treatment group. Treatment emergent adverse events will be further summarised by maximum severity and relationship to the investigational medicinal product.

Category	Description
	All safety summaries will be descriptive; no statistical significance tests will be performed on safety data and will be based on the safety population.<br><br>Gold (Au) concentration data in whole blood will be summarized with descriptive statistics by treatment group at each time point of collection.

Table 1: Time and Events Schedule

Time and Events Schedule	Visit	-1	0	1	2	3	4	5	6	7	8
	Phase	Screening	Baseline	Treatment Period							Safety Follow-Up [b]
	Week	-6	0	3	6	12	18	24	30	36	40
	Day	-42 to -1	1	21 [a]	42 [a]	84 [a]	126 [a]	168 [a]	210 [a]	252 [a]	280 [c]
ICF Signed		X									
Eligibility Review		X	X								
Medical History & Prior Med Assess.		X									
Physical Examination		X	X			X [d]		X [d]		X [d]	X
Brief Neurological Exam		X	X			X		X		X	X
Anthropometrics (Height and Weight Assessment)		X	X [e]			X [e]		X [e]		X [e]	
Urine Drug Test		X									
HIV/Viral Hepatitis Screen		X									
Serum Pregnancy Test		X [f]									
Urine Pregnancy Test			X [f]			X [f]		X [f]		X [f]	
Vital Signs		X	X			X		X		X	X
12-lead ECG		X [g]	X [g]			X [g]		X [g]		X [g]	
Clinical Laboratory (Blood)		X	X			X		X		X	X
Urinalysis		X	X			X		X		X	X
Concomitant Medication		X	X	X	X	X	X	X	X	X	X
Randomisation			X								
Dispense/Return Drug			X			X		X		X	
Schedule Next Visit		X	X	X	X	X	X	X	X	X	
PD Sampling (Blood, Urine)			X [h]			X [h]		X [h]		X [h]	
PK Sampling (Blood)						X [h]		X [h]		X [i]	
Adverse Events		X	X	X	X	X	X	X	X	X	X
EMG: MUNIX, MUSIX, CMAP of the APB, ADM, BB, TA, FDI			X [j]			X [j]		X [j]		X [j]	
EMG: ADM, APB (F-Wave); Distal Motor Latency (APB, ADM, BB, TA, FDI)			X [j]			X [j]		X [j]		X [j]	
EMG: MScan for the APB			X [j]			X [j]		X [j]		X [j]	
ALSFRS-R		X	X			X		X		X	
FVC		X	X			X		X		X	
ALSSQOL-SF			X			X		X		X	
Health Utilisation Form (e.g., PEG, NIV, Wheel Chair)			X			X		X		X	
Falls Questionnaire						X		X		X	
PGI (Patient Global Impression)			X			X		X		X	

Time and Events Schedule	Visit	-1	0	1	2	3	4	5	6	7	8
	Phase	Screening	Baseline	Treatment Period							Safety Follow-Up [b]
	Week	-6	0	3	6	12	18	24	30	36	40
	Day	-42 to -1	1	21 [a]	42 [a]	84 [a]	126 [a]	168 [a]	210 [a]	252 [a]	280 [c]
CGI (Clinical Global Impression)			X			X		X		X	
Columbia-Suicide Severity Rating Scale (C-SSRS)		X	X			X		X		X	X
Phone call				X	X		X		X		

Time and Events Schedule Notes:

a. Scheduled Visit ± 5 days.
b. For patients not transitioning to the optional Open-Label extension phase.
c. Timing for the Safety Follow-Up visit should occur at four weeks (±5 days) following study early termination, or. following the patient's final Week 36 visit if the participant chooses not to enter the optional OLE period.
d. Brief physical exam only.
e. Weight only
f. For females of child bearing potential only.
g. Electrocardiogram (ECG) intervals will be summarized and presented descriptively. ECG rhythm will be interpreted by the Investigator as normal (N), abnormal not-clinically significant (aNCS), or abnormal clinically significant (aCS). Triplicate values will be collected at Baseline and averaged for comparison to single assessments at subsequent visits.
h. Whole blood, plasma, and/or serum for PK and PD will be taken pre-dose only (e.g., within1 hour prior to the dose of study drug).
i. Whole blood for PK will be taken at pre-dose (T_0) and at 1, 2, 4, and 6 hours after dosing for the visit. The exact time at which the patient took his/her previous day's study drug dose must be recorded in order to impute a 24-hour trough value ($T_{24\text{-imputed}}$).
j. EMG to be conducted on the least clinically affected hand, leg, and arm identified at the Baseline visit.

Table 2: Time and Events Schedule (Optional Open-Label Extension)

Time and Events Schedule	Visit	0	1	2, 4, 6, (n+2)	3, 5, 7, (n+2)	End of OLE
	Phase	Baseline [a]	Open-Label Extension Phase			Safety Follow-Up [c]
	Week	0	6	12, 36, 60, + every 24 weeks as applicable	24, 48, 72, + every 24 weeks as applicable	n+4
	Day	0	42 [b]	84, 252, 420, n+168 [b]	168, 336, 504, n+168 [b]	Last visit +28 [b]
ICF Signed		X				
Eligibility Review and Confirmation		X				
Brief Physical Examination		X		X	X	X
Brief Neurological Exam		X		X	X	X
Weight Assessment		X			X	
Urine Pregnancy Test [d]		X		X	X	
Vital Signs		X		X	X	X
12-lead ECG [e]		X			X	
Clinical Laboratory (Blood)		X		X	X	X
Urinalysis		X		X	X	X
Dispense/Return Drug		X		X	X	
Schedule Next Visit		X	X	X	X	
PK/PD Sampling (Blood, Urine)		X			X [f]	
Adverse Events and Concomitant Medication Review		X	X	X	X	X
EMG: MUNIX, MUSIX, CMAP of the APB, ADM, BB, TA, FDI		X [g]			X [g]	
EMG: ADM, APB (F-Wave); Distal Motor Latency (APB, ADM, BB, TA, FDI)		X [g]			X [g]	
EMG: MScan for the APB		X [g]			X [g]	
ALSFRS-R		X		X	X	
FVC		X		X	X	
ALSSQOL-SF		X		X	X	
Health Utilisation Form (e.g., PEG, NIV, Wheel Chair)		X		X	X	
Falls Questionnaire				X	X	
PGI (Patient Global Impression)		X		X	X	
CGI (Clinical Global Impression)		X		X	X	
Columbia-Suicide Severity Rating Scale (C-SSRS)		X		X	X	X
Phone call			X			

Optional Open-Label Extension Time and Events Schedule Notes:

a. The Baseline Visit for the optional OLE study will correlate with the Week 36 visit of the randomized placebo controlled phase. For those participants who may enter this OLE phase 12 or more weeks after completing their Week 36 visit, Baseline assessment indicated in Table 2 will be completed and serve as the participants baseline for the OLE phase.
b. Scheduled Visit ± 5 days.
c. Timing for the Safety Follow-Up visit should occur at four weeks (±5 days) following early termination or the patient's last OLE period visit.
d. For females of child bearing potential only.
e. Electrocardiogram (ECG) intervals will be summarized and presented descriptively. ECG rhythm will be interpreted by the Investigator as normal (N), abnormal not-clinically significant (aNCS), or abnormal clinically significant (aCS).
f. Whole blood, plasma, serum, and/or urine for PK and PD will be taken pre-dose only (e.g., within1 hour prior to the dose of study drug).
g. EMG to be conducted on the least clinically affected hand, leg, and arm identified at the randomized placebo controlled phase Baseline visit.

TABLE OF CONTENTS

PROTOCOL APPROVAL PAGE **2**

INVESTIGATOR PROTOCOL AGREEMENT **3**

PROTOCOL SYNOPSIS **4**

1 BACKGROUND INFORMATION **29**

1.1 DISEASE BACKGROUND AND PATHOPHYSIOLOGY 29

1.2.1 Investigational Product Background *32*

1.2.2 Investigational Product Characteristics *33*

1.4.1 In vitro ALS Neuroprotection Models *38*

1.4.1.1 CNM-Au8 Protects Primary Rodent Motor Neurons From Excitotoxicity *38*

1.4.1.2 CNM-Au8 Protects Normal iPSC Derived Human Motor Neurons From ALS-Patient-Derived Toxic Astrocytes *40*

1.4.2 In vivo ALS Neuroprotection Models *40*

1.4.3 Summary ALS Treatment Rationale *43*

2 SUMMARY OF NONCLINICAL SAFETY & TOXICOLOGY STUDIES **44**

2.1 SUMMARY OF SAFETY MUTAGENICITY STUDIES 44

2.2 SUMMARY OF SAFETY PHARMACOLOGY STUDIES 45

2.3 SUMMARY OF TOXICOLOGY AND TOXICOKINETIC STUDIES IN RODENTS AND MINIPIGS .. 45

2.3.1 Initial Toxicokinetic Studies in Rodents and Minipigs *45*

2.3.1.1 Study AB17834 (28-Day Repeat Dose Toxicity in Göttingen Minipig) 47

2.3.2 Maximum Feasible Dose Study and High Dose Subchronic Toxicity Studies in Rodents and Minipigs *48*

2.3.3 Chronic Rodent Toxicology (6-Month Dosing) *49*

2.4 SUMMARY OF TOXICOLOGY AND TOXICOKINETIC STUDIES IN CANINES 50

2.4.1 Single Dose Canine Toxicokinetic Summary *52*

2.4.2 Repeat Dose Canine Toxicokinetic Summary *54*

2.4.3 Maximum Feasible Dose Canine Toxicokinetic Study *55*

2.4.4 Chronic Canine Toxicology (9-Month Dosing) *56*

3 PRIOR HUMAN DOSING EXPERIENCE **58**

3.1 AU8.1000-14-01 DESIGN 58

3.2 AU8.1000-14-01 PHARMACOKINETIC RESULTS 58

3.3 AU8.1000-14-01 SAFETY RESULTS 59

4 INVESTIGATIONAL PLAN **62**

4.1 RATIONALE FOR THE STUDY 62

4.2 STUDY OBJECTIVES 62

4.3 OVERALL STUDY DESIGN AND PLAN 62

5 INVESTIGATORS AND KEY STUDY PERSONNEL **64**

6 STUDY ENDPOINTS **66**

6.1 EFFICACY ENDPOINTS (TREATMENT PERIOD) 66

6.1.1 Primary Efficacy Endpoint *66*

6.1.2 Secondary Efficacy Endpoint *66*

6.1.3 Exploratory Efficacy Endpoints *66*

6.1.3.1 Exploratory Electromyography Endpoints 66

6.1.3.2 Exploratory Clinical Efficacy Endpoints 67

6.1.4 Pharmacokinetic Endpoints ... 68
6.1.5 Pharmacodynamic Endpoints ... 68
6.1.5.1 Whole Blood Metabolomic Markers ... 69
6.2 OPEN-LABEL EXTENSION ENDPOINTS ... 69
6.2.1 Primary Safety Endpoint ... 69
6.2.2 Primary Efficacy Endpoint ... 69
6.2.3 Secondary and Exploratory Efficacy Endpoints ... 69

7 STUDY POPULATION ... 70
7.1 STUDY INCLUSION CRITERIA ... 70
7.2 STUDY EXCLUSION CRITERIA ... 70
7.3 REPRODUCTIVE POTENTIAL ... 72
7.4 REMOVAL OF PATIENTS FROM THERAPY/PREMATURE DISCONTINUATION ... 73
7.4.1 Patient Replacement ... 74
7.4.2 Reasons for Discontinuation ... 74
7.4.2.1 Lost to Follow-Up ... 74
7.5 PATIENT IDENTIFICATION ... 74

8 INVESTIGATIONAL DRUG PRODUCT ... 76
8.1.1 Treatments Administered ... 76
8.1.2 Identity of Investigational Products ... 76
8.1.2.1 CNM-Au8 ... 76
8.1.2.2 Placebo ... 77
8.1.3 Labeling ... 78
8.1.4 Storage and Handling ... 79
8.2 METHOD OF ASSIGNING PATIENTS TO TREATMENT GROUPS AND DOSE SELECTION ... 79
8.2.1 Treatment Assignment/Randomisation ... 79
8.2.2 First In Human Dosing Experience ... 79
8.2.3 Human Dosing Safety Margins vs. Nonclinical Toxicokinetic Data ... 80
8.3 PROCEDURES FOR BLINDING ... 82
8.3.1 Unblinding ... 83
8.3.1.1 Unblinding by Sponsor's Pharmacovigilance Representative ... 83
8.3.1.2 Emergency Unblinding By The Investigator ... 83
8.3.1.3 Unblinding For Ongoing Safety Monitoring ... 83
8.4 PRIOR AND CONCOMITANT THERAPY ... 84
8.5 TREATMENT COMPLIANCE ... 84
8.5.1 Study Drug Accountability ... 85
8.6 DATA SAFETY MONITORING BOARD ... 85

9 STUDY PROCEDURES ... 86
9.1 STUDY SCHEDULE (TREATMENT PERIOD) ... 86
9.1.1 Screening Phase (Day -42 through Day -1) – VISIT (-1) ... 86
9.1.2 Baseline (Day 1) – VISIT 0 ... 87
9.1.3 Treatment Phase (VISIT 1 through VISIT 7) ... 88
9.1.3.1 Treatment Period – VISIT 1, 2, 4, and 6 (WEEK 3, 6, 18, and 30) ... 88
9.1.3.2 Treatment Period – VISITS 3, 5, and 7 ... 88
9.1.3.3 Safety Follow-up Visit – Week 40 ... 89
9.2 STUDY SCHEDULE (OPEN-LABEL EXTENSION PERIOD) ... 90
9.2.1 OLE Baseline (Day 1) – Visit 0 ... 90
9.2.2 OLE Period – Visit 1 (Week 6) ... 91
9.2.2.1 OLE Period – Visits 2, 4, 6, and n+2 (Weeks 12, 36, 60, and n+24) ... 92
9.2.2.2 OLE Period – Visits 3, 5, 7 and n+2 (Weeks 24, 48, 72 and n+24) ... 92

9.2.2.3 Safety Follow-Up Visit – 4 weeks following last visit ... 94
9.3 STUDY MEASUREMENTS AND ASSESSMENTS ... 94
9.3.1 Demographic and Other Baseline Characteristics ... 94
9.3.2 Assessment of Efficacy ... 94
9.3.2.1 Electromyography Data Collection ... 95
9.3.2.2 MUNIX ... 95
9.3.2.3 MScan and MScan Fit ... 96
9.3.2.4 MUSIX ... 96
9.3.2.5 Neurophysiological Index (NPI) ... 96
9.3.2.6 Split Hand Index (SI) ... 97
9.3.2.7 ALSFRS-R ... 97
9.3.2.8 FVC ... 97
9.3.2.9 CGI ... 98
9.3.2.10 PGI ... 98
9.3.2.11 Healthcare Utilisation ... 98
9.3.3 Assessment of Safety ... 98
9.3.4 PK and PD Assessments ... 99
9.3.4.1 Collecting, Processing, and Shipping of PK and PD Samples ... 100
9.3.4.2 Bioanalytical Methods ... 100
9.3.5 Clinical Laboratory Tests ... 101
9.3.6 Physical Examinations ... 102
9.3.7 Vital Signs ... 102
9.3.8 12-Lead ECG ... 102
9.4 ASSESSMENT WINDOWS ... 102
10 ADVERSE EVENTS ... 103
10.1 SAFETY REPORTING GUIDELINES ... 103
10.1.1 Sponsor Guidance ... 103
10.1.2 Investigator Guidance ... 103
10.2 ADVERSE EVENTS (AE OR ADVERSE EXPERIENCE) ... 103
10.2.1 Intensity of Adverse Events ... 105
10.2.2 Relationship and Attribution of the AE to Study Drug ... 105
10.2.2.1 Relationship to Study Drug ... 105
10.2.2.2 Attribution to The Study Drug ... 106
10.3 SERIOUS ADVERSE EVENTS ... 106
10.3.1 Serious Adverse Events Related To Study-Mandated Procedures ... 107
10.4 REPORTING REQUIREMENTS FOR AES AND SAES ... 107
10.4.1 Reporting of Adverse Events ... 107
10.4.2 Reporting Abnormal Laboratory Test Values or Abnormal Clinical Findings ... 108
10.4.2.1 Reporting of AE Intensity and Causality ... 108
10.4.2.2 Follow-up of AEs ... 108
10.4.3 Reporting Requirements For SAEs ... 108
10.4.3.1 Reporting of SAEs ... 108
10.4.3.2 SAE Reporting Periods ... 109
10.4.3.3 SAE Reporting Procedures ... 110
10.4.3.4 SAE Regulatory Reporting ... 110
10.5 PREGNANCY ... 111
10.5.1 Prevention of Pregnancy During the Study ... 111
10.5.2 Reporting of Pregnancy ... 111
10.5.3 Pregnancy Follow-Up ... 112
10.6 STUDY HALTING RULES ... 112
10.7 CLINICAL MONITORING ... 112

10.7.1 Site Monitoring Plan ... *112*

11 STATISTICS AND DATA MANAGEMENT ... 113

11.1 DETERMINATION OF SAMPLE SIZE AND ANALYSES ... 113
11.2 ANALYSIS POPULATIONS ... 113
11.3 BASELINE CHARACTERISTICS AND PATIENT DISPOSITION ... 114
11.4 PRIOR AND CONCOMITANT MEDICATIONS ... 114
11.5 PHARMACOKINETIC ANALYSES ... 114
11.6 SAFETY ANALYSES ... 115
11.7 EFFICACY ANALYSES ... 115
11.8 INTERIM ANALYSES ... 116
11.9 DATA HANDLING AND QUALITY ASSURANCE ... 116
11.9.1 Data Recording ... *116*
11.9.1.1 Data Recorded From Screening Failures ... 116
11.9.2 Data Monitoring ... *117*
11.9.3 Data Collection and Management ... *117*
11.9.4 Data Audit and Inspection ... *118*
11.9.5 Data Archiving ... *118*

12 RESPONSIBILITIES, ETHICS, AND LEGAL ASPECTS ... 119

12.1 INVESTIGATOR RESPONSIBILITIES ... 119
12.1.1 Good Clinical Practice ... *119*
12.1.2 Institutional Review Board (IRB)/Independent Ethics Committee (IEC) Approval .. *119*
12.1.3 Informed Consent ... *119*
12.1.4 Adherence to Protocol ... *120*
12.1.5 Confidentiality ... *120*
12.1.6 Study Files and Retention of Records ... *120*
12.1.7 Case Report Forms ... *121*
12.1.8 Drug Accountability ... *121*
12.1.9 Inspections ... *122*
12.2 SPONSOR RESPONSIBILITIES ... 122
12.2.1 Study Materials and Instructions ... *122*
12.2.2 Protocol Modifications ... *122*
12.2.3 Insurance ... *123*
12.2.4 Premature Study Termination ... *123*
12.3 JOINT INVESTIGATOR/SPONSOR RESPONSIBILITIES ... 123
12.3.1 Access to Information for Monitoring ... *123*
12.3.2 Study Report and Publications ... *124*

13 PROTOCOL AMENDMENTS ... 125

14 APPENDICES ... 126

15 REFERENCES ... 127

LIST OF IN-TEXT TABLES

Table 1: Time and Events Schedule 14
Table 2: Time and Events Schedule (Optional Open-Label Extension) 16
Table 3. Estimated Volume, Mass, and Particle Characteristics of CNM-Au8 33
Table 4. Summary of Safety Mutagenicity Studies 44
Table 5. Summary of Safety Pharmacology Studies 45
Table 6. Initial Acute and Subchronic Dosing Studies in Rats and Minipigs 46
Table 7: Study AB17834 Platelet Change from Day -12 to Day 27 in Select Animals 47
Table 8. Maximum Feasible Dose Study and High Dose Subchronic Toxicity Studies 49
Table 9. Chronic Rodent Toxicity Study 50
Table 10. Acute and Subchronic Dosing Studies in Canines 52
Table 11. Summary of C_{max}, AUC, $T_{1/2}$, and Bioavailability Results Following Single Dose Administration of CNM-Au8 in Study CNAU-MPI-14-01 53
Table 12. Summary of CL, $T_{1/2}$, V_z, and V_{ss} after a Single 0.5 mg/kg IV Dose of CNM-Au8 (Study CNAU-MPI-14-01) 54
Table 13. Summary of CL/F, $T_{1/2}$, and Vz/F after a Single 10 mg/kg PO Dose of CNM-Au8 (Study CNAU-MPI-14-01) 54
Table 14. Chronic Canine Toxicity Study 57
Table 15. CNM-Au8 Investigational Product Dosing Administration 77
Table 16. CNM-Au8 Components and Quality Standards 77
Table 17. Matched Placebo Dosing Administration 77
Table 18. Matched Placebo Components and Quality Standards 78
Table 19. Summary of CNM-Au8 Conversion of Animal 21-Day Repeat Doses To Human Equivalent Dose (HED) Based On Body Surface Area (mg/m^2) for 60 kg Human 81
Table 20. Summary of CNM-Au8 Exposure Safety Margin Based on End of Study $AUC_{(0-24)}$ ng*hr/mL. Ratio of Animal Toxicokinetic to Human Pharmacokinetic AUC Results From 21-Day Repeat Dose Studies. 81
Table 21. Summary of CNM-Au8 Exposure Safety Margin Based on End of Study $AUC_{(0-24)}$ ng*hr/mL Ratio of Chronic Animal Toxicokinetic 6 and 9-Month Rodent and Canine Repeat Dose Studies to Human 21-Day Pharmacokinetic Results 82
Table 22. EMG Data Collection Plan 95
Table 23. AE Attribution to the Study Drug 106
Table 24. Summary of Study Safety Reporting Periods 111
Table 25. Summary of PK Parameters 114

LIST OF IN-TEXT FIGURES

Figure 1. CNM-Au8 NADH Catalysis Rates 35
Figure 2. Effects of CNM-Au8 on NAD Levels in Primary Rodent Mesencephalic Cultures 36
Figure 3. Effects of CNM-Au8 on NADPH Oxidation 36
Figure 4 . Effects of CNM-Au8 on SOD and ROS Generation 37
Figure 5. Effect of CNM-Au8 on OPC Differentiation and ATP Production 38
Figure 6 . Neuroprotection of Motor Neurons From Glutamate Excitotoxicity by CNM-Au8 39
Figure 7. CNM-Au8 Protects Normal iPSC Derived Human Motor Neurons From ALS-Patient-Derived Toxic Astrocytes 40
Figure 8. Locomotor Functional Efficacy of CNM-Au8 in an SOD1^{G93A} (C57Bl/6 congenic strain) Murine Study 41
Figure 9. Effect of CNM-Au8 on Survival in a Congenic Murine SOD1 G93A Model 42
Figure 10. Whole Blood Concentrations by Day in 28-Day Repeat Dosing Canine Study 55
Figure 11. Study Scheme 63
Figure 12. 21-Day AUC Exposure Ranges Between Rodent Toxicokinetic Studies, Preclinical Efficacy Models, and First-In-Human Dosing 82

ABBREVIATIONS

Abbreviation	Definition
aCS	Abnormal clinically significant
AE	Adverse event
AESI	Adverse events of special interest
ALS	Amytrophic Lateral Sclerosis
ALSFRS-R	Amytrophic Lateral Sclerosis Functional Rating Scale- Revised
ALT	Alanine aminotransferase
ANCOVA	Analysis of covariance
aNCS	Abnormal not clinically significant
AST	Aspartate aminotransaminase
ASTM	American Society for Testing and Materials
ATP	Adenosine Trinucleotide Phosphate
Au	Gold
AUC	Area under the curve
$AUC_{(0-24)}$	Area under the curve for 24 hours
βhCG	Beta-human chorionic gonadotropin
BID	Twice a day (*bis in die*)
BLQ	Below the limit of quantification
BMI	Body mass index
bpm	Beats per minute
BUN	Blood urea nitrogen
C_0	Initial or back-extrapolated plasma drug concentration at time zero following bolus intravenous injection
CA	Competent authority
CFR	Code of Federal Regulations
CGI	Clinician's Global Impression
CHDR	Center for Human Drug Research
CK	Creatine kinase
CL	Apparent total body clearance of the drug
CL/F	Apparent total clearance of the drug from whole blood after oral administration
CLr	Renal clearance
Cmax	Peak plasma concentration, observed
CNM-Au8	Aqueous suspension of clean surfaced nanocrystals consisting of gold atoms self-organized into crystals of various faceted, geometrical shapes
CNS	Central nervous system
CRF	Case report form
CRO	Contract research organization
CRU	Clinical research unit
CSF	Cerebrospinal fluid or cerebral spinal fluid
CTA	Clinical trial application

Abbreviation	Definition
CTAB	Cetyl trimethylammonium bromide
CTCAE	Common terminology criteria for adverse events
CV%	Coefficient of variation
λ_Z	Terminal phase rate constant (first-order)
DE	Differentially expressed
DMT	Disease modifying therapy
DO	Doctor of osteopathic medicine
DTPA	Diethylenetriaminepentacetate
EC	Ethics committee
ECG	Electrocardiogram
eCRF	Electronic case report form
EDC	Electronic data collection
EEG	Electroencephalogram
EOS	End of study
F	Bioavailability (systemic availability of the administered dose)
fALS	Familial Amytrophic Lateral Sclerosis
FCS-AuNC	Faceted clean surfaced Au nanocrystal
FDA	Food and Drug Administration
GCP	Good clinical practice
GFR	Glomerular filtration rate
GGT	Gamma-glutamyl transferase
GMP	Good manufacturing practices
H_20_2	Hydrogen peroxide
$HAuCl_4$	Chloroauric acid
HepB	Hepatitis B
HepC	Hepatitis C
HED	Human equivalent dose
HDPE	High density polyethylene
HIV	Human immunodeficiency virus
hr	Hour
HREC	Human Research Ethics Committee
IB	Investigator's Brochure
ICH	International Conference on Harmonization
ICH-GCP	International Conference on Harmonization – Good Clinical Practice
ICP-MS	Inductively Coupled Plasma Mass Spectrometry
IEC	Independent Ethics Committee
IND	Investigational New Drug
INR	International normalised ratio
IRB	Institutional Review Board
ISO	International Organization for Standardization

Abbreviation	Definition
ITT	Intent to treat
IV	Intravenous
K_2EDTA	Dipotassium ethylenediaminetetraacetic acid
kg	Kilogram
LDH	Lactate dehydrogenase
LGN	Lateral geniculate nucleus
LOQ/LLOQ	Limit of quantitation or Lower limit of quantitation
LPS	Lipopolysaccharide
m	meter
M:E	Ratio of myeloid to erythroid precursors in bone marrow
MAD	Multiple ascending dose
max	Maximum
MD	Doctor of medicine
MedDRA	Medical Dictionary for Regulatory Activities
MFD	Maximum feasible dose
mg	Milligram
min	Minute(s)
mL	Milliliter
mmHg	Millimeters of mercury
MRI	Magnetic Resonance Imaging
MRS	Magnetic Resonance Spectroscopy
mRNA	Messenger Ribonucleic acid (RNA)
ms	Milliseconds
MTD	Maximum tolerated dose
MWF	Myelin water fraction
N	Normal
NAD+	Oxidized form of nicotinamide adenine dinucleotide
NADH	Reduced form of nicotinamide adenine dinucleotide
NADPH	Reduced form of nicotinamide adenine dinucleotide phosphate
$NaHCO_3$	Baking soda
NCI	National Cancer Institute
NCS	Not clinically significant
ng	nanogram
nm	nanometer
NOAEL	No observed adverse effect level
OG	Oral gavage
OL	Oligodendrocyte
OLE	Open Label Extension
ON	Optic neuritis
OPC	Oligodendrocyte progenitor cells

Abbreviation	Definition
OR	Optic radiation
OTC	Over-the-counter
PA	Physician assistant
PAS	Partial analysis set
PCE	Polychromatic erythrocytes
PD	Pharmacodynamic
PEG	Polyethylene glycol
PGI	Participant Global Impression
pH	Potential hydrogen (relative acidity or alkalinity)
PK	Pharmacokinetic
PO	Oral administration (*per os)*
PPP	Pentose phosphate pathway
PPS	Per protocol set
PT	Preferred term
QD	Once daily (*quaque die*)
QTc	Corrected QT interval (measure of the time between the start of the Q wave and the end of the T wave)
R_{ac}	Accumulation ratio
SAD	Single ascending dose
SAE	Serious adverse event
sALS	Sporadic Amyptrophic Lateral Sclerosis
SAP	Statistical analysis plan
SAS	Safety analysis set
SC	Subcutaneous
SD	Standard deviation
SEM	Standard error of the mean
SGOT	Serum glutamic oxaloacetic transaminase
SGPT	Serum glutamate-pyruvic transaminase
SOC	System organ class
SOD1	Superoxide dismutase
SOP	Standard operating procedure
SRC	Safety Review Committee
SUSAR	Suspected unexpected serious adverse reaction
T½	Elimination half-life
T1	Time constant for the longitudinal relaxation time following an MRI radio-frequency pulse
T2	Time constant for the transverse relaxation time (perpendicular to the main field) following an MRI radio-frequency pulse
T3	Triiodothyronine (thyroid hormone)
TEAE	Treatment emergent adverse event
TEM	Transmission electron microscopy
TID	Three times daily (*ter in die*)

Abbreviation	Definition
TK	Toxicokinetic
T_{max}	Time to peak plasma concentration, observed
Ue	Urinary excretion
µM	Micrometer
USP	United States Pharmacopeia
V_{ss}	Volume of distribution at steady state
V_z	Apparent volume of distribution (Vz)
V_z/F	Apparent volume of distribution during terminal phase after non-intravenous administration

1 BACKGROUND INFORMATION

1.1 Disease Background and Pathophysiology

Amyotrophic lateral sclerosis (ALS) is an adult-onset, progressive, and fatal neurodegenerative disorder of the neuromuscular system resulting in muscle weakness and paralysis that leads to death as early as three to five years after initial diagnosis. In 2015, there were an estimated 222,801 cases of ALS worldwide, which has been projected to increase by 69% to 376,674 cases by 2040, a gain largely attributable to an ageing population (Arthur et al., 2016). ALS not only causes severe distress for patients and caregivers, but also results in a large economic burden on society (Gladman and Zinman, 2015). Despite these significant tolls and robust efforts to identify efficacious therapies for this disease, there have been limited clinical successes and no curative therapies approved to date (Mitsumoto et al., 2014). There are two FDA-approved therapeutic agents for the treatment of ALS: riluzole, an anti-glutamatergic agent, and edavarone, a free-radical scavenger. However, both of these treatments are acknowledged to have limited disease-modifying effects, as riluzole extends patient lifespans by an average of only two to three months, while edavarone slows the decline of the ALSFRS-R score only in a small subset of patients who are at an early stage of disease (Jaiswal, 2018). There is clearly an urgent unmet need for the development of safe and effective disease-modifying therapeutics for ALS.

While most ALS cases are sporadic (sALS) in which the cause remains unknown, 5-10% of cases are familial forms of the disease (fALS). fALS cases arise from variants in single genes, including SOD1, TDP43, FUS, and C9ORF72 and are clinically indistinguishable from sporadic cases (sALS). The functions of these specific genetic fALS variants on balance involve RNA processing and metabolism, regulation of protein aggregation, maintenance of mitochondrial function, and protection against oxidative stress. During adult life, motor neurons that harbor specific genetic or epigenetic changes, or that have been exposed to unidentified environmental factors, may become exquisitely sensitive to dysregulation of these pathways, resulting in an imbalance between signals regulating neuron survival versus programmed cell death. In addition, there appear to be extrinsic factors related to astrocytes, oligodendrocytes, and cells involved in neuroinflammatory responses that can exacerbate or ameliorate the survival of motor neurons in patients with ALS.

More recently, basic research has uncovered interconnections amongst the seemingly disparate functions of genes mutated in fALS. Importantly, these emerging insights suggest impairment of pathways related to oxidative stress and mitochondrial function. These are represented by mutations in superoxide dismutase (SOD1) on one hand, and regulation of RNA processing and metabolism, represented by mutations in FUS, TDP43 and several more genes, on the other. Both oxidative stress and RNA dysregulation are indicative of an underlying unifying pathogenetic mechanism of bioenergetic failure in ALS. Bioenergetic affects not only the target cell type, the motor neurons, but

also the astrocytes and oligodendrocytes that provide crucial energetic support to the survival of motor neurons. Postmortem analyses of brain tissues from both fALS and sALS patients exhibit evidence of widespread accumulation of oxidative damage to proteins, lipids, and DNA. Transgenic mice expressing mutant SOD1 forms have evidence of nerve and spinal cord tissue damage that recapitulates key aspects of ALS and demonstrates clear evidence of excessive protein and lipid oxidation (D'Amico et al. 2013) . Oxidative stress also leads to the accumulation of protein aggregates such as TDP43 (Bozzo, Mirra, and Carrì 2017)(Bozzo et al., 2017) , which are invariably found in ALS motor neurons, and which indicate failures of protein degradation systems such as autophagy (Blokhuis et al., 2013).

Importantly, elevated oxidative stress has been shown to impact the processing of gene transcripts important for motor neuron survival. This observation connecting oxidative stress to RNA processing was demonstrated when paraquat, a well-known inducer of oxidative stress, was shown to induce striking changes in RNA splicing in neuroblastoma cells (Maracchioni et al., 2007). Two of the pre-mRNAs whose splicing was shown to be altered in this study are transcripts of genes known to be important for motor neuron survival, Survival Motor Neuron (SMN) and Apoptotic Peptidase Activating Factor 1 (APAF1) (Maracchioni et al., 2007). The mislocalization of the RNA-binding protein TDP43 in ALS represents another link between oxidative stress and RNA dysmetabolism. In general, TDP43 is involved in pre-RNA processing in the nucleus, where it functions in the transcriptional control of many genes including those involved in neural functions. However, oxidative stress causes the TDP43 to translocate to the cytoplasm, where it accumulates in stress granules and forms protein aggregates. Sequestration of an RNA regulator such as TDP43 in the cytoplasm and away from the nucleus is thought to prevent it from carrying out its normal functions and thereby can lead to dysregulation of RNA processing and transcription. The converse relationship has similarly been shown, namely that misregulation of RNA metabolism impacts and increases oxidative stress within the CNS. Mutations in RNA regulators FUS and TDP43 cause disruptions in the expression of key nuclear-encoded mitochondrial genes (Zhang et al., 2014) as well as in the expression of energy metabolism regulators such as PGC-1α and oxidative stress reducers such as MnSOD and catalase (Sanchez-Ramos et al., 2011). Therefore, the pathophysiologic disease mechanism of ALS appears to involve a positive feedback cycle of oxidative stress and protein aggregation impacting RNA regulation which in turn leads to a sustained increased oxidative stress disease state, ending in motor neuron death (Bozzo, Mirra, and Carrì 2017)(Bozzo et al., 2017) .

Unifying both themes of RNA dysregulation and oxidative stress is the concept that ALS is a disease of energetic dysmetabolism (Dupuis et al., 2011; Tefera and Borges, 2016; Vandoorne et al., 2018). Motor neurons consume high amounts of energy in order to function, and are therefore exquisitely sensitive to apoptosis if their energetic demands cannot be met. The main energy-producing organelle of the cell, the mitochondria, exhibit dysfunction in ALS on multiple levels. For example, overall

there are fewer mitochondria, as measured by mitochondrial DNA, in the spinal cords of both fALS and sALS patients (Wiedemann et al., 2002). Presynaptic mitochondrial swelling in motor neurons has been observed in both ALS patients (Siklos et al., 1996), as well as in a SOD1 transgenic mouse model prior to disease onset (Manfredi and Xu, 2005). Swollen and vacuolarized mitochondria are indicative of impaired mitochondrial function (Siklos et al., 1996), which is corroborated by studies showing that there is decreased activity of the electron transport chain in spinal cord mitochondria of ALS patients (Wiedemann et al., 2002). Because reduced respiration and reduced ATP synthesis preceded clinical symptom onset in $SOD1^{G93A}$ mice (Jung et al., 2002; Mattiazzi et al., 2002; Szelechowski et al., 2018), energetic dysmetabolism appears to play an important role in the etiology of this disease. Overexpression of PGC1α, which stimulates mitochondrial biogenesis, improved survival, motor neuron function, and motor neuron survival in mutant $SOD1^{G93A}$ mice (Zhao et al., 2011), indicating that improving mitochondrial function and/or efficiency may be a successful therapeutic strategy for ALS (Vandoorne et al., 2018).

Energetic dysmetabolism in ALS is further underscored by studies uncovering carbohydrate metabolism abnormalities and ATP deficits in ALS motor neurons (Vandoorne et al., 2018). FDG-PET imaging has demonstrated a correlation between reduced ALS brain glucose uptake and cognitive impairment (Canosa et al., 2016) as well as overall disease severity (Dalakas et al., 1987). Some groups who conducted FDG-PET studies showing reduced glucose uptake in ALS patient brains suggested CNS hypometabolism can be used as an early diagnostic indication of ALS (Van Laere et al., 2014; Van Weehaeghe et al., 2016). While it is difficult to determine whether lowered glucose utilization detected in imaging studies is due to reduced neuronal catabolism of glucose or due to motor neuron loss, there are further lines of evidence to suggest energetic dysmetabolism in ALS cells. A proteomic study of ALS patient fibroblasts indicated a significant reduction in key enzymes involved in glycolysis (Szelechowski et al., 2018), and expression profiling of sALS patient motor cortexes showed downregulation of glycolytic genes (Lederer et al., 2007). A GWAS analysis involving 12577 cases and 23475 controls identified glycolysis/gluconeogenesis as among the top seven biological pathways represented by the genes identified as associated with ALS (Du et al., 2018). Because glia preferentially utilize glycolysis, these results may indicate a dysfunction of glia in providing energetic support to motor neurons in ALS patients. Energetic stress on motor neurons may increase their levels of oxidative stress, as observed in post-mortem brain samples of ALS patients (Ferrante et al., 1997), and lead to motor neuron death.

Given the clear involvement of cellular bioenergetic failure in the pathogenesis of ALS, the development of a disease modifying therapeutic that addresses the energetic dysregulation underlying the progressive accumulation of oxidative stress and dysregulated RNA processing is a rational therapeutic strategy.

1.2 Investigational Product Rationale and Characteristics

1.2.1 Investigational Product Background

CNM-Au8 is an aqueous suspension of clean-surfaced nanocrystals consisting solely of Au atoms organized into crystals of highly faceted substantially uniform geometrical shapes. CNM-Au8 is supplied for dosing orally in sodium bicarbonate buffered USP purified water.

Historically, monomolecular gold complexes (e.g., injectable sodium aurothiomalate, aurothioglucose; oral auranofin) have been widely used historically as immunomodulating therapies in the treatment of autoimmune disorders, predominantly rheumatoid arthritis (Dabrowiak, 2009). However, multiple forms of toxicity, including pruritus, dermatitis, stomatitis, diarrhea, proteinuria, and less frequently hematological effects compromised the clinical use of gold complexes (Dabrowiak, 2009; Menninger et al., 1998). While the role of gold in the pattern of toxicity remains unclear, it is hypothesized that adverse events (AEs) related to gold complexes may be specifically related to the monomolecular covalent formulations of the gold complexes rather than from the activity of gold *per se* (Dabrowiak, 2009; Yei Ho and Tiekink, 2005).

When functionalized as nanocrystals, gold adopts novel electrochemical characteristics unlike those of gold complexes. In addition, as clean-faceted nanocrystals, CNM-Au8 has unique properties including an improved safety and tolerability profile as well as higher level of biocatalytic activity in cells unlike that of gold nanoparticles manufactured using traditional methods that require stabilizing or residual chemical modifications to the surfaces of the particles.

The faceted gold nanocrystals of CNM-Au8 generally consist of shapes including hexagonal bi-pyramids, pentagonal bi-pyramids, tetrahedrons, and octahedrons. These crystalline shapes, absent organic residues on their surfaces, are central to the biologic activity of CNM-Au8. In contrast to molecular covalently bound gold compounds (e.g., historical gold salts), or colloidal gold nanoparticles containing surface residue or surfactants as a surface layer (e.g., polyethylene glycol (PEG), cetyl trimethylammonium bromide (CTAB), citrate, thiols, other surfactants), CNM-Au8 consists solely of nanocrystals grown through an electro-crystal chemistry process, which produces consistently faceted catalytically active geometrical shapes (without utilizing surfactants such as those derived from the reduction of chloroauric acid ($HAuCl_4$)).

The characteristics of a nanoparticle’s size, shape, and surface chemistry impact, and define the biological activity of the nanoparticles. Residual surface chemistry also affects the biological activity of nanoparticles and directly impacts cellular uptake. Nanoparticle surfactants and residual chemical monolayers are also well-known to cause nanoparticle toxicity (Balasubramanian et al., 2010; Freese et al., 2012; Qiu et al., 2010).

In summary, CNM-Au8 was designed to promote biocatalytic activity on clean-surfaced, faceted nanocrystalline surfaces.

1.2.2 Investigational Product Characteristics

The median diameter of CNM-Au8 nanocrystals is approximately 13 nm, as determined by transmission electron microscopy (TEM). Based upon the distributed range ($D_n5 – D_n95$: 6.5 – 15.8 nm) of measured nanocrystal diameters and approximate geometrical shapes (e.g., low volume estimate: disc-like approximation, aspect ratio of 0.2; maximum volume estimate: spheroid, aspect ratio of 1.0), each Au nanocrystal has an approximate composition ranging from 13,000 – 66,000 Au atoms per nanocrystal at the 13 nm median diameter with a corresponding molar mass ranging between 2.7 x 10^3 kDA to 1.3 x 10^4 kDA. Summary estimates for CNM-Au8 mass, volume, and particle characteristics are described below in Table 3. Each mL of CNM-Au8 suspension at 500 μg/mL is estimated to contain between 100 - 500 trillion highly faceted Au nanocrystals.

Table 3. Estimated Volume, Mass, and Particle Characteristics of CNM-Au8

**Metric** (CNM-Au8 500 μg/mL, 60 mL Dose)	**Disc-Like Approximation Minimum** *(Aspect 0.2)*	**Spherical Approximation Maximum** *(Aspect 1.0)*
Median Au Nanocrystal Diameter (nm)	13	
Au Nanocrystal Volume (nm^3)	2.3 x 10^2	1.2 x 10^3
Au Nanocrystal Surface Area (nm^2)	3.2 x 10^2	5.3 x 10^2
Au Atoms per Nanocrystal (count)	1.4 x 10^4	6.8 x 10^4
Au Nanocrystal Molecular Weight (kDa)	2.7 x 10^3	1.3 x 10^4
Total Au Nanocrystal Surface Area per mL (cm^2)	3.6 x10^2	1.2 x10^2
Au Nanocrystals per mL CNM-Au8 (count)	1.1 x 10^{14}	2.3 x 10^{13}
Au Nanocrystals per 60 mL Dose of CNM-Au8 (count)	3.4 x 10^{15}	6.8 x 10^{14}

1.3 Preclinical Evidence of Mechanistic Rationale for CNM-Au8 Treatment of ALS

CNM-Au8 passes into the systemic circulation via intestinal absorption. Cellular uptake of CNM-Au8 has been demonstrated in a variety of cells and tissue matrices including macrophages, oligodendrocytes, neurons, and brain tissue using Inductively Coupled Plasma Mass Spectrometry (ICP-MS) analyses and high resolution TEM visualization of nanocrystals.

Preclinical characterization of the *in vitro* and *in vivo* properties of CNM-Au8 have demonstrated that it acts to protect neuronal populations from chemical, inflammatory, and hypoxic insults through a

series of unique catalytic mechanisms involving 1) the nicotine adenine dinucleotide redox couple (NAD+/NADH) to boost glycolytic energy production, 2) the nicotine adenine dinucleotide phosphate redox couple (NADP+/NADPH) to influence anabolic processes associated with differentiation, and 3) the SOD-like inactivation of reactive oxygen species and nitric oxide to protect cells from oxidative damage and mitochondrial dysfunction.

The nicotinamide adenine dinucleotide redox couple (NAD+, NADH) plays a central role in the energy metabolism of all living cells. NAD+, NADH and their relative intracellular ratio (NAD+/NADH) are linked to regulation of energy homeostasis, neuroprotection, immune function, chromosome stability, DNA repair mechanisms, sleep and circadian rhythms, and longevity (Canto et al., 2015; Imai, 2010a, b; Nikiforov et al., 2015; Ying, 2008). Fundamentally, NAD+ and NADH are essential coenzymes in the adenosine triphosphate (ATP)-generating reactions driving both glycolysis and oxidative phosphorylation. More specifically, in aerobic glycolysis, NAD+ availability is integral to the enzymatic cascade from glyceraldehyde 3-phosphate via glyceraldehyde phosphate dehydrogenase to 1,3-bisphosphoglyceric acid. Similarly, two electrons are removed via NADH oxidation in Complex I of the electron transport chain during oxidative phosphorylation, resulting in NAD+, and ultimately these electrons are transferred to a lipid-soluble carrier, ubiquinone.

In addition, NAD+ and its metabolites act as binding substrates for a wide range of proteins including the metabolic and transcription-regulating sirtuins, the poly-ADP-ribose polymerases (PARPs) involved in DNA repair, and the cyclic ADP-ribose synthases (cADPRs) such as CD38 that serve to regulate Ca^{2+} signaling involved in cell cycle control and insulin signaling (Canto et al., 2015).

NAD+ and NADH are essential for life; dietary deficiency of vitamin B3, a NAD+ precursor, gives rise to a life-threatening condition known as pellagra, which is characterized by a dark, pigmented skin rash, diarrhea, and dementia (Sydenstricker, 1958). Transgenic mice lacking both copies of the NAD+ salvage enzyme *Nmnat1* resulting in NAD+ deficiency, do not live past embryonic stages (Conforti et al., 2011). Conversely, the heightened expression of NMNAT1, resulting in increased NAD+ in the Wallerian Degeneration Slow Dominant Mutant Mouse, has been attributed to neuronal protection against axon degeneration observed in these mice (Sasaki et al., 2009).

Quantitative measurement of regional brain area NAD+ concentrations and redox states in healthy and diseased humans has recently been demonstrated using a novel ^{31}P-Magnetic Resonance Spectroscopy (MRS) imaging technique (Chouinard et al., 2017; Lu et al., 2016). In humans, brain NAD+/NADH redox potential is inversely correlated with age. This age-related decrease in cerebral energy metabolism is believed to be intimately associated with the bioenergetic failure that may underpin the pathophysiology of all neurodegenerative diseases, because NAD+/NADH redox potential is essential for fundamental ATP-generating processes such as glycolysis and mitochondrial oxidative phosphorylation. These bioenergetic processes power all cellular processes including 'housekeeping'

functions that are responsible for maintaining overall cellular health including processes of autophagy, apoptosis, and the unfolded protein response (UPR) (Villanueva-Paz et al. 2016; Chua and Tang 2013; Cantó, Menzies, and Auwerx 2015) . Boosting brain NAD+ levels in a mouse model of AD reduced DNA damage, neuroinflammation, and apoptosis while improving cognitive function in multiple behavioral tests and restored hippocampal synaptic plasticity (Hou et al., 2018). In humans, ^{31}P-MRS of the visual cortex of young (21-26 year old), middle-aged (33-36 year old), and aged (59-68 year old) healthy individuals has been show to demonstrate a measurable linear decline of NAD+ redox potential that occurs with increasing age (Zhu et al., 2015).

The first demonstration of oxidation of NADH to NAD+ by gold nanoparticles was demonstrated by (Huang et al., 2005) using a simple cell-free assay. In order to compare the catalytic oxidation rate of clean-surfaced CNM-Au8 against similarly-sized gold nanoparticles made using citrate reduction, the same cell-free assay utilized by Huang et al. was repeated comparing gold nanoparticles sourced from the U.S. National Institutes of Standards and Technology (NIST). The resulting change in the 339 nm NADH peak was assessed while investigating each of the gold nanoparticles at the same NADH and Au starting concentrations. In all cases, CNM-Au8 nanocrystals consistently showed significantly superior catalytic activities regardless of size and/or method of preparation of the comparator gold nanoparticles (Figure 1).

Figure 1. CNM-Au8 NADH Catalysis Rates

Figure 1A. CNM-Au8 Catalytic Effects vs. NIST Standard Citrated AuNP

Figure 1B. Relative NADH Oxidation Rates vs. NIST Citrated AuNP

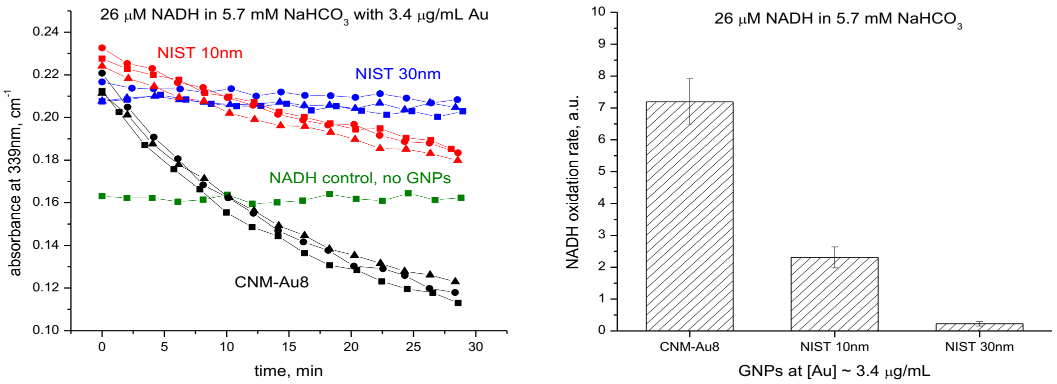

CNM-Au8 treatment of rodent central nervous system cells *in vitro* also significantly increases levels of both NAD+ and NADH (Figure 2).

Figure 2. Effects of CNM-Au8 on NAD Levels in Primary Rodent Mesencephalic Cultures

Figure 2A. Effect of CNM-Au8 on NAD+ Levels

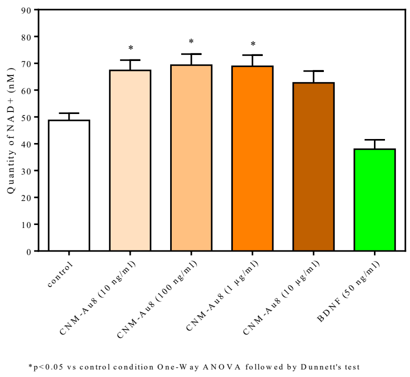

Figure 2B. Effect of CNM-Au8 on NADH Levels

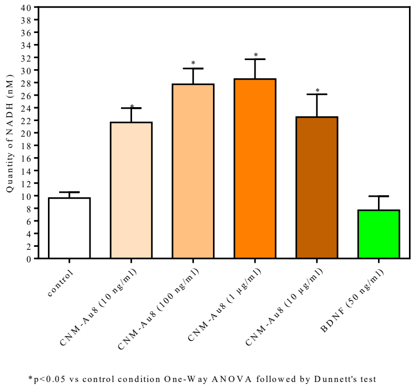

The redox couple NADP+/NADPH, similar to NAD+/NADH, plays a fundamental role in energy homeostasis, anabolic processes, and protection against oxidative stress. NADP+ and NADPH function in the pentose phosphate pathway (PPP), serves to provide precursors for nucleotide and amino acid biosynthesis (Stincone et al., 2015). In addition, NADP+ and NADPH serve as the redox equivalents for both the thioredoxin and glutathione systems (Grant, 2008), which in turn serve as the cell's primary defenses against oxidative stress.

CNM-Au8 catalyzes the oxidation of NADPH to NADP+ (Figure 3). While the time course of catalysis of NADPH to NADP+ is longer than that observed for conversion of NADH to NAD+, the magnitude of the effect is similar. Thus, CNM-Au8 plays an important role in providing NADP+ to the enzyme glucose 6-phosphate dehydrogenase for the conversion of 6-phosphogluconate to ribulose 5-phosphate and NADPH + CO_2, which represents the rate-limiting step of the oxidative branch of the PPP.

Figure 3. Effects of CNM-Au8 on NADPH Oxidation

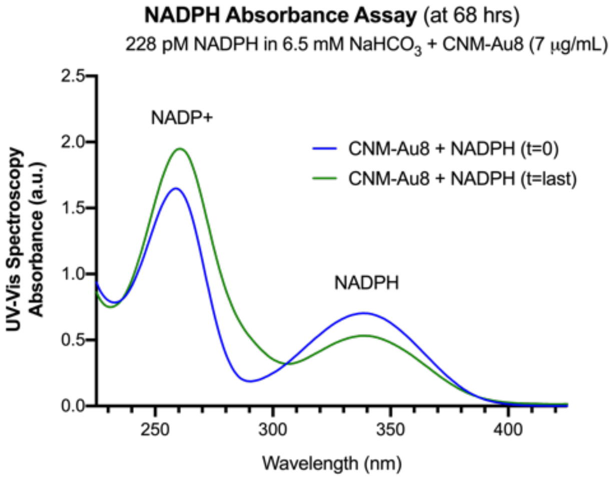

Excessive reactive oxygen exposure disrupts cellular redox homeostasis, damages intracellular organelles as well as DNA and proteins, and may also underpin pathophysiologic mechanisms of ALS. Super oxide dismutases (SODs) evolved as a component of the cell's antioxidant defense system to regulate the levels of reactive oxygen species (ROS) and prevent damage from oxidative stress. SOD enzymes are found in the cytoplasm and mitochondria where they convert oxygen radicals to O_2 or H_2O_2, which can in turn be converted to water and O_2 by catalases. A dose-dependent reduction of ROS levels was observed in differentiating oligodendrocyte precursor cells in primary culture with CNM-Au8 treatment (Figure 4).

Figure 4 . Effects of CNM-Au8 on SOD and ROS Generation

Figure 4A. Effects on SOD Activity Assay

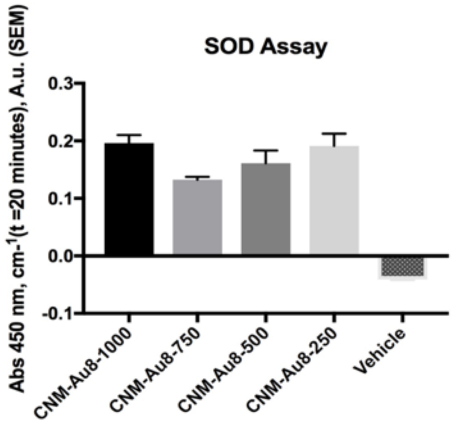

Figure 4B. Effects on ROS Generation in Purified Murine OPC cultures

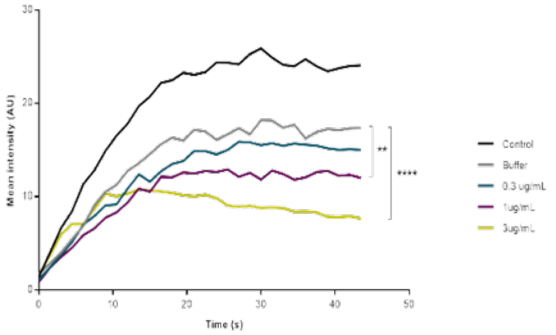

Figure 4C. Effects on ROS Generation in Murine OPC Cultures Plus Rotenone

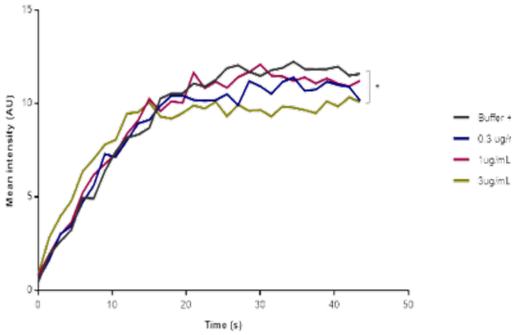

Demonstration of SOD-like catalytic activity with CNM-Au8 treatment has several important implications. First, neurons and glial cells are energetically demanding and generate large amounts of ROS as byproducts of the energetic processes necessary for their function. Theories of aging and neurodegeneration suggest that as CNS cells age, they are less able to efficiently remove ROS and thereby succumb to the consequences of long-term ROS exposure. As CNS cells are exquisitely sensitive to ROS, they are among the first cell types to degenerate, leading to the cognitive, psychomotor, and movement impairments observed in diseases such as ALS, Alzheimer's disease, and Parkinson's disease (Angelova and Abramov, 2018). In addition, SOD1 may act not only as a

regulator of ROS but also as a part of a glucose-oxygen sensing mechanism of a cell to determine whether the cell utilizes oxidative phosphorylation or aerobic glycolysis for ATP production (Reddi and Culotta, 2013). A study of oligodendrocyte (OL) energetics showed that human OLs preferentially use aerobic glycolysis during differentiation and myelination (Rone et al., 2016). Data with CNM-Au8 demonstrate that treatment switches OPCs from proliferation to differentiation, while simultaneously stimulating aerobic glycolysis production of ATP likely through NADH oxidation (Figure 5). Stimulation of SOD-like activity therefore is consistent with a role for CNM-Au8 in regulating cellular bioenergetics.

Figure 5. Effect of CNM-Au8 on OPC Differentiation and ATP Production

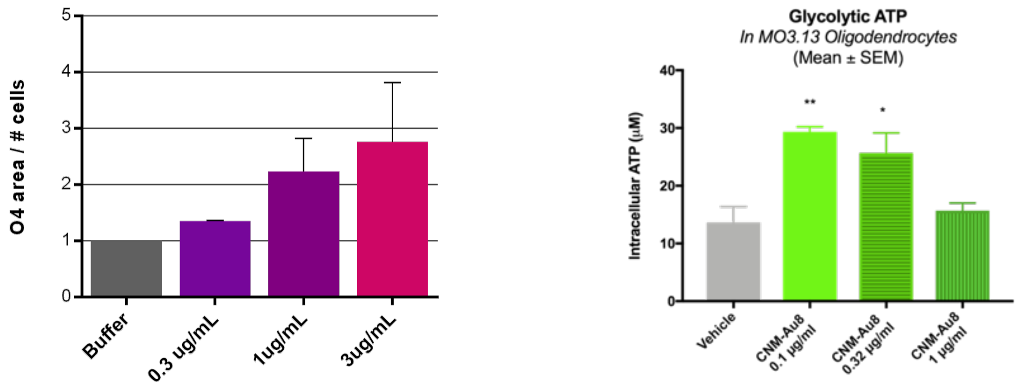

Effect of CNM-Au8 on a) OPC differentiation (O4+ cells) in isoldated murine OPCs and b) ATP production in MO3.13 oligodendrocytes (72hr). Statistical analysis performed using one-way ANOVA ($p < 0.05$).

1.4 Nonclinical Summary of In Vitro and In Vivo ALS Neuroprotection Studies

1.4.1 *In vitro* ALS Neuroprotection Models

CNM-Au8 exhibits neuroprotective effects in a broad range of neural cell types in *in vitro* and *in vivo* preclinical models of ALS.

1.4.1.1 CNM-Au8 Protects Primary Rodent Motor Neurons From Excitotoxicity

Excitotoxicity is the pathological process by which neuronal death occurs as a result of excessive stimulation of receptors at excitatory synapses such as the NMDA receptor. Excitotoxicity has been implicated in acute neurological damage from ischemia and traumatic brain injury and in the chronic neurodegeneration in Huntington's disease. Glutamate excitoxicity is also believed to be a significant contributor to motor neuron death in ALS. Excitotoxic neuronal death via over-activation of NMDA receptors contributes to excessive flux of calcium (Ca2+) into the cell. This triggers a range of responses resulting in cell death, including increased oxidative stress, inappropriate activation of proteases such as calpain, dysregulation of Ca2+-related pathways, mitochondrial damage, and the apoptotic cascade.

Determination of the neuroprotective effects of CNM-Au8 on motor neurons in an *in vitro* glutamate challenge model was carried out in ventral spinal cord cultures from E14 rat embryos (Martinou et al., 1992). On Day 11 after seeding, cultures were pre-treated with CNM-Au8 or vehicle. The positive control riluzole was added for 1 hour of pre-treatment before glutamate addition. On Day 13, glutamate was added to the cultures for 20 minutes, followed by treatment of riluzole or CNM-Au8 for another 48 hours before cells were fixed and stained. Cultures were stained with anti-MAP-2 to assess motor neuron survival and neurite lengths, and also with TDP-43 and Hoechst stain to assess extranuclear TDP43. CNM-Au8 dose-dependently protects motor neurons from glutamate-induced cell death (Figure 6A) while also preserving neurite length (Figure 6B). CNM-Au8 also outperformed riluzole on neurite network preservation. In addition, CNM-Au8 treatment prevented the accumulation of cytoplasmic TDP-43, a hallmark of ALS cytotoxicity (Figure 6D).

Figure 6 . Neuroprotection of Motor Neurons From Glutamate Excitotoxicity by CNM-Au8

Figure 6*A. Motor Neuron Survival*

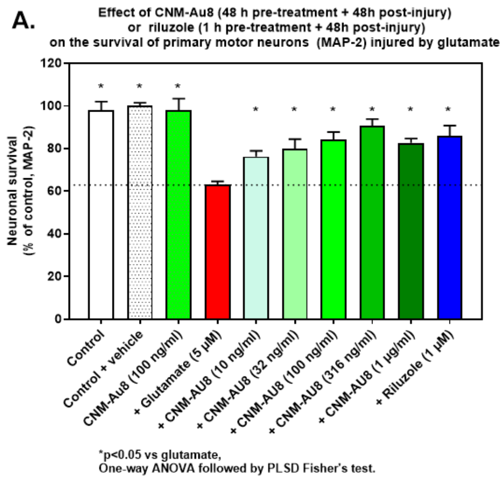

Figure 6*B. Motor Neuron Neurtie Network Area*

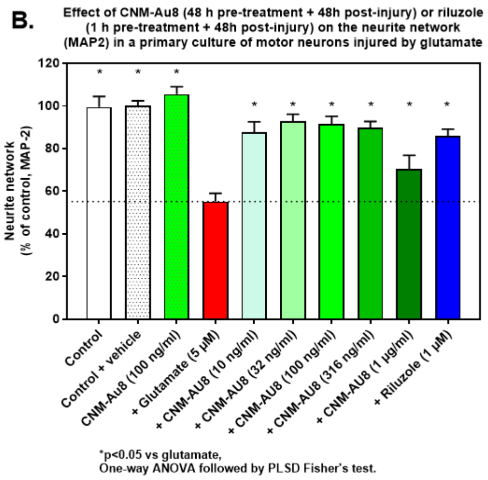

Figure 6*D. Reduction in Cytoplasmic TDP43*

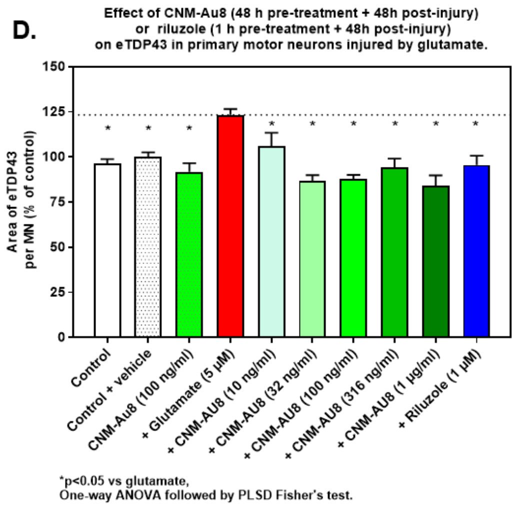

1.4.1.2 CNM-Au8 Protects Normal iPSC Derived Human Motor Neurons From ALS-Patient-Derived Toxic Astrocytes

Astrocytes from both sporadic and familial ALS patients display toxicity against normal human motor neurons (MN) in co-culture (Meyer et al., 2014a). Toxic ALS-patient-derived astrocytes appear to secrete an unidentified toxic factor; and when co-cultured with human MNs, the MNs die (Meyer et al., 2014a). Similar studies have shown that astrocytes from sporadic patients also exhibit toxicity against healthy MNs, likely by similar mechanisms (Haidet-Phillips et al., 2011; Meyer et al., 2014b; Qian et al., 2017). To determine whether CNM-Au8 protects MNs from toxic ALS-patient-derived astrocytes, healthy human iPSC-derived MNs were plated with $SOD1^{A4V}$ astrocytes for 14 days in the presence of various doses of CNM-Au8 or vehicle. Neuroprotection was assessed by quantification of the expression of neuronal markers (Tuj1, Isl1/2, and ChAT) compared to vehicle treated control. Measurement of primary neurite lengths and a neurite network complexity Sholl analysis was also conducted (Sholl, 1953). As shown in Figure 7, significant neuroprotective dose-dependent effects of CNM-Au8 treatment were consistently observed on survival in the presence of $SOD1^{A4V}$ patient-derived toxic astrocytes.

Figure 7. CNM-Au8 Protects Normal iPSC Derived Human Motor Neurons From ALS-Patient-Derived Toxic Astrocytes

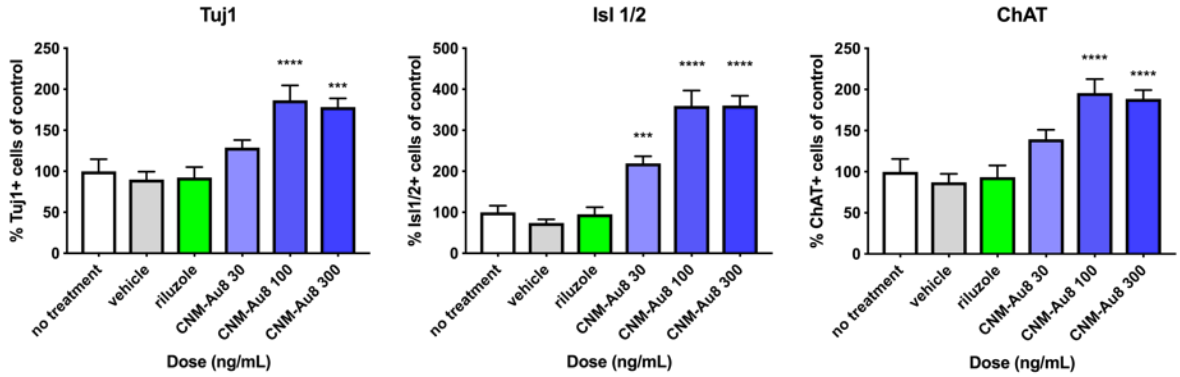

1.4.2 *In vivo* ALS Neuroprotection Models

To demonstrate the efficacy of CNM-Au8 treatment in an *in vivo* model of ALS, two studies were conducted in separate $SOD1^{G93A}$ mouse model strains. These included the rapidly progressive $SOD1^{G93A}$ transgenic line on a mixed SJL/C57BL6 background (Heiman-Patterson et al., 2005) and the more slowly progressive $SOD1^{G93A}$ transgenic line on a congenic C57Bl/6 background (Lutz, 2018) with lifespans of ~157 days compared to ~129 days, respectively. The study using rapidly progressing $SOD1^{G93A}$ animals showed only minor improvement in clinical onset ($p=0.13$, Mantel-Cox test), as well as significant lack of brainstem atrophy ($p<0.05$, unpaired t-test) in the CNM-Au8-treated group (N=15 animals per group;); all other functional measures were not significant (data not shown). In the study of the slower progressing $SOD1^{G93A}$ (N=20 female mice, 10 per group), four-

week-old female SOD1 mice were randomly assigned one of two groups with each group balanced for weight.

Beginning at four weeks of age, mice were provided either CNM-Au8 or vehicle *ad libitum* in their drinking water. Beginning at Week 8 of age each mouse was tested weekly for strength and motor coordination utilizing several measures including the triple horizontal bar hang time test, static rod orientation test, inverted screen hang time test, weight hold test, and home cage wheel activity (average speed) in addition to standard ALS clinical scoring (Deacon, 2013a, 2013b; Hatzipetros et al., 2015). The CNM-Au8-treated group significantly outperforms the vehicle treated group at virtually all timepoints across these tests (Figure 8). In addition, survival benefits have been observed through Week 22 of the study (Figure 9).

Taken together, these preliminary data from an ongoing study in a transgenic ALS mouse model demonstrate preservation of motor function following chronic treatment with CNM-Au8.

Figure 8. Locomotor Functional Efficacy of CNM-Au8 in an SOD1^{G93A} (C57Bl/6 congenic strain) Murine Study

A. ALS Clinical Score

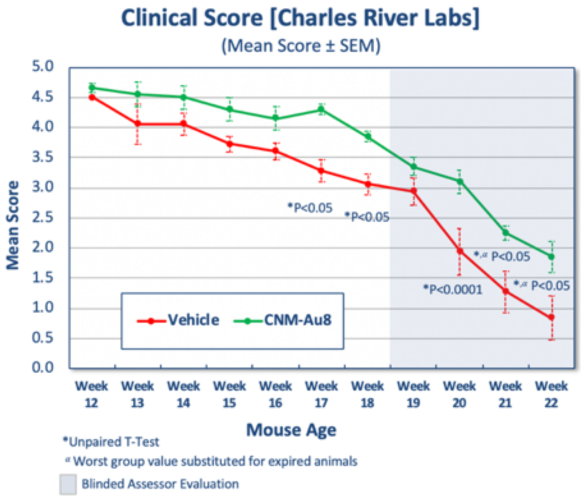

B. ALS TDI Neuro Score

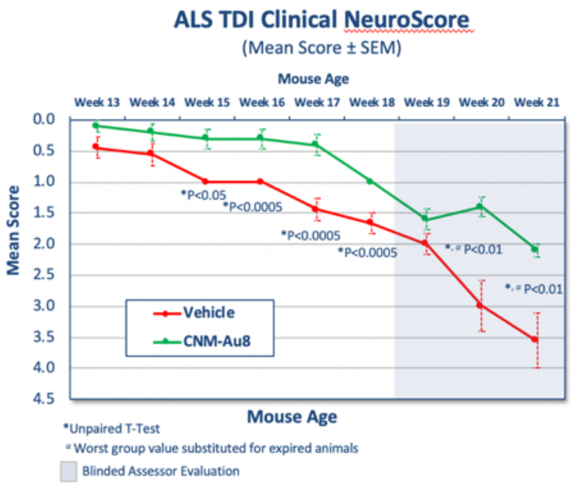

Figure 8. Locomotor Functional Efficacy of CNM-Au8 in an SOD1G93A (C57Bl/6 congenic strain) Murine Study

C. Weights Hold Test

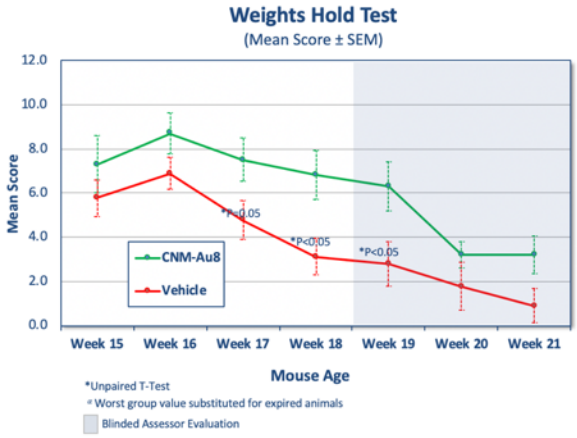

Figure 8. Locomotor Functional Efficacy of CNM-Au8 in an SOD1G93A (C57Bl/6 congenic strain) Murine Study

D. Static Rod Orientation Time

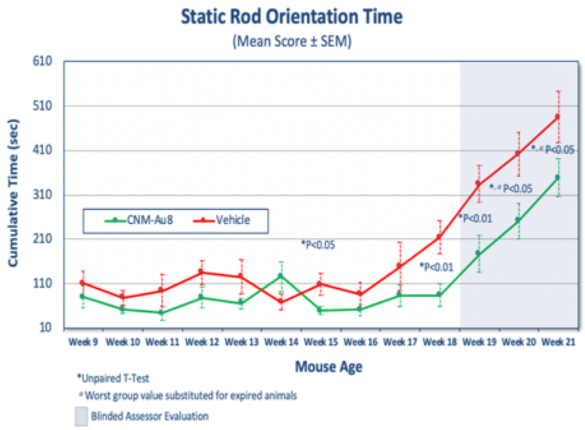

E. Triple Horizontal Bar Hang Time

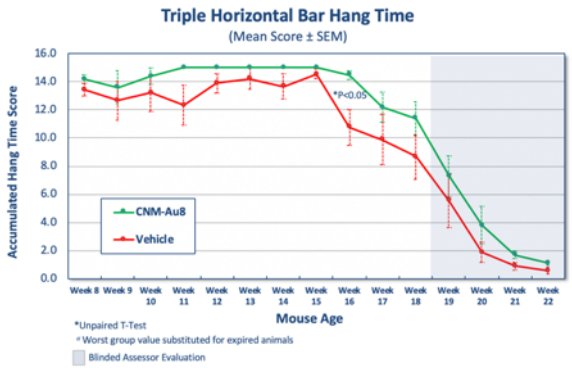

F. Inverted Screen Hang Time

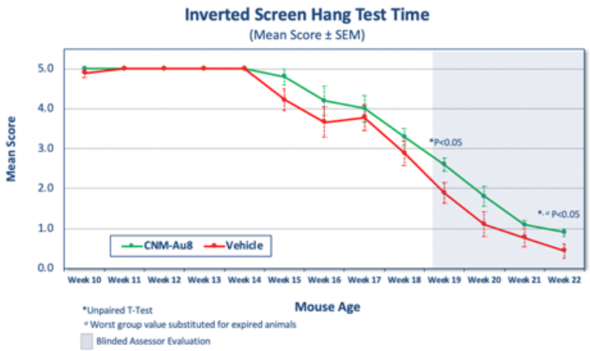

Figure 9. Effect of CNM-Au8 on Survival in a Congenic Murine SOD1 G93A Model

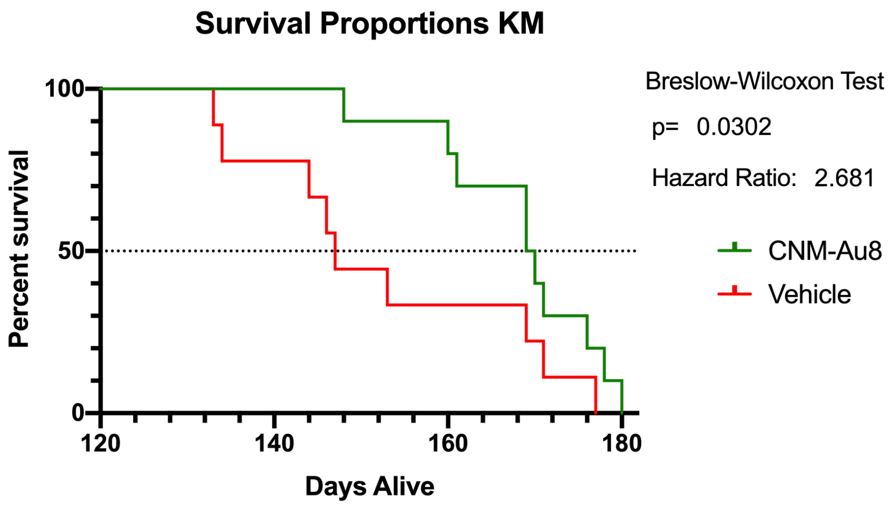

1.4.3 *Summary* ALS Treatment Rationale

CNM-Au8-treated motor neurons are protected against cell death by glutamate excitotoxicity in a dose-dependent manner in a primary rat neural-glial co-culture where cytoplasmic levels of TDP-43 are significantly reduced. Aberrant cytoplasmic localization and aggregation of TDP-43 has been observed in over 90% of cases of ALS in humans (Neumann et al., 2006). Further, results from these multiple iPSC human motor neuron studies derived from sporadic, familial, and healthy lines indicate that CNM-Au8 is neuroprotective to MNs challenged with multiple different ALS disease related stressors, namely, excitotoxic and toxic astrocytic stress. Results from the SOD1 G93A-overexpression mouse study demonstrate significant improvements in locomotor performance of CNM-Au8-treated mice with a significant survival benefit.

In summary, CNM-Au8 has demonstrated neuroprotection across a range of neuronal cell types, anti-oxidant activity to counteract the accumulation of ROS in ALS, and preservation of motor function in an ALS mouse model. CNM-Au8 therefore represents an important therapeutic candidate for ALS.

2 SUMMARY OF NONCLINICAL SAFETY & TOXICOLOGY STUDIES

The Sponsor has conducted multiple safety and toxicology studies with CNM-Au8 in accordance with ICH M3 (R2) guidelines across three species including rats, minipigs, and canines, in addition to standard *in vitro* mutagenicity studies. Initial *in vivo* dosing levels were modeled on delivering comparable amounts of gold as the historical gold complex, auranofin, to a 50-kg patient on a milligram per kilogram basis without adjustments for body surface area variance between species. This resulted in target nonclinical daily doses of Au at an estimated 0.0348 mg/kg/day, which were increased 10-fold (0.348 mg/kg) and 100-fold (3.48 mg/kg) to provide an anticipated safety margin for planned human dosing.

2.1 Summary of Safety Mutagenicity Studies

Three safety mutagenicity studies were conducted to address the mutagenic potential of CNM-Au8 including *in vivo* Micronucleus testing, and the in vitro Mammalian Cell Gene Mutation Test and Bacterial Reverse Mutation Test. These studies each demonstrated no effect of CNM-Au8 on cytotoxicity or mutation frequency as summarized below in Table 4.

Table 4. Summary of Safety Mutagenicity Studies

Study ID (Internal ID/ CRO ID)	GLP Status	Mutagenicity Model	Brief Design [Route; Frequency]	Effective Dose	CNM-Au8 Conc. (µg/mL)	Summary/Notes
AB06525	GLP	L5178Y Mouse Lymphoma Cells (Thymidine Kinase+/-)	Mammalian Cell Gene Mutation Test (In vitro)	1.7, 3.5, 5.2, 8.7 and 17 µg/mL	348	No signs of cytotoxicity; no biologically significant increases in the mutant frequency
AB05703	GLP	Salmonella typhimurium	Bacterial Reverse Mutation Test (In vitro)	17, 35, 70, 104, 174 µg/plate	348	No precipitate and no sign of cytotoxicity were noted in any strain and at any dose level, either with or without metabolic activation
AB17832 (sub-study)	GLP	Rat Micronucleus Test	Bone Marrow Micronucleus Test after 4-weeks of treatment (In vivo)	N/A	0.0348, 0.348, 3.48	No significant increase in the frequency of micronucleated polychromatic erythrocytes; no decrease in the PCE/NCE ratio
CNM-Au8-MPI-17-01	GLP	Rat Micronucleus Test	Bone Marrow Micronucleus Test after 2-days of treatment (In vivo)	N/A	10, 20, 40 mg/kg/d	No significant increase in the incidence of micronuclei in the test article dosed animals compared to the vehicle control. The test article CNM-Au8 was evaluated as negative (non-clastogenic)

2.2 Summary of Safety Pharmacology Studies

Safety pharmacology assessments included testing of three doses of CNM-Au8 (0.0348, 0.348 and 3.48 mg/kg) in rodents in the standard CNS Irwin test and the same dosing regimen for assessment of renal function following saline overload. Cardiovascular safety was assessed by constant telemetry in minipigs. The CNS Irwin testing protocol provided no evidence CNM-Au8 affected central and peripheral neurologic function. Similarly, renal function was not affected in Han Wistar rats following saline overload. Telemetry recording of cardiovascular responses in minipigs including heart rate, blood pressure, ECG, and body temperature demonstrated no negative effects of CNM-Au8 on cardiovascular function. These safety pharmacology results are summarized in Table 5.

Table 5. Summary of Safety Pharmacology Studies

**Study ID** (Internal ID/ CRO ID)	**GLP Status**	**Species** (N)	**Brief Design** [Route; Frequency]	**Nominal Dose** (mg/kg/day)	**CNM-Au8 Conc.** (µg/mL)	**Summary/Notes**
AB17835	GLP	Han Wistar Rat (N=24)	CNS Irwin Test Following Single-Dose [OG, QD]	0.0348	3.3	No relevant effect on a battery of behavioral and physiological parameters, covering the main central and peripheral nervous system functions, evaluated for 24 hours after dosing
				0.348	36.6	
				3.48	346.5	
AB17836	GLP	Han Wistar Rat (N=40)	Renal Function Following Saline Overload [OG, QD]	0.0348	3.3	No mortality or clinical signs; no change in the serum concentrations of sodium, potassium, chloride or creatinine or in the serum osmolality; no changes in urine volume, pH, osmolality, electrolytes, or creatinine; no change in GF, free water clearance, or excretion fractions of electrolytes
				0.348	36.6	
				3.48	346.5	
AB17837	GLP	Göttingen Minipig (N=6)	Cardiovascular Telemetry (11-Day + Washout + PK) [OG, QD]	0.0348	3.3	No effect on general health status, body weight gain, or body temperature; no relevant effects on the arterial blood pressure, heart rate, or ECG parameters
				0.348	36.6	
				3.48	346.5	

2.3 Summary of Toxicology and Toxicokinetic Studies in Rodents and Minipigs

2.3.1 Initial Toxicokinetic Studies in Rodents and Minipigs

Two initial toxicokinetic studies were completed in the Göttingen Minipig and two initial studies were completed in rats as summarized in Table 6.

These initial studies were conducted up to a maximum of 3.48 mg/kg or approximately 100-fold higher delivery of gold when compared to comparable doses of auranofin in humans. These studies did not identify any treatment related toxicities, so a maximum tolerated dose was not defined. The no-adverse-effect-level (NOAEL) was identified at 3.48 mg/kg in rats and minipigs.

Table 6. Initial Acute and Subchronic Dosing Studies in Rats and Minipigs

**Study ID** (Internal ID/ CRO ID)	**GLP Status**	**Species** (N)	**Brief Design** [Route; Frequency]	**Nominal Dose** (mg/kg/day)	**CNM-Au8 Conc.** (µg/mL)	**Safety Summary/ Toxicity Notes**
AB17839	Non-GLP	Göttingen Minipig (N=2)	1-Day & 5-Day Acute Toxicity; 2-phases [OG; QD]	0.0348	3.7	No mortality; no treatment related clinical signs; no related macroscopic abnormalities
				0.348	35.3	
				3.48	343.3	
AB17834	GLP	Göttingen Minipig (N=24; 6/group)	28-Day Repeat Dose Toxicity [OG; QD]	0.0348	3.3	No mortality; no treatment-related clinical signs or ophthalmological findings; no treatment-related effects on hematology, serum clinical chemistry, and urine parameters; no treatment-related macroscopic or histological findings
				0.348	36.6	
				3.48	346.5	
AB17831	Non-GLP	Han-Wistar Rat (N=40; 10/group)	7-Day Acute Toxicity [OG; QD]	0.0348	3.7	No mortality; no clinical signs; no treatment-related changes in body weight, body weight gain and food consumption; no effects on hematology and serum clinical chemistry; no treatment-related changes in organ weights; no macroscopic and histopathological changes
				0.348	35.3	
				3.48	343.3	
AB17832	GLP	Han-Wistar Rat (N=100; 25/group)	28-Day Repeat Dose Toxicity [OG; QD]	0.0348	3.3	No mortality; no clinical signs; no treatment-related changes in body weight, body weight gain and food consumption; no effects on hematology, coagulation, serum clinical chemistry and urine parameters; no treatment-related ophthalmological findings at any dose level; no organ weight, macroscopic changes or histological findings
				0.348	36.6	
				3.48	346.5	

2.3.1.1 Study AB17834 (28-Day Repeat Dose Toxicity in Göttingen Minipig)

At the end of the treatment period in Study AB17834, a lower platelet count from the pretest baseline was noted in 3 animals as described in Table 7, below. One female in Group 2 (low dose) and one in Group 3 (intermediate dose) had very low levels of platelets counts, and one male in Group 4 (high dose) group had a significant reduction.

Table 7: Study AB17834 Platelet Change from Day -12 to Day 27 in Select Animals

Group	Animal ID	Platelets (Giga/L)	
		Day -12	Day 27
Low Dose (0.0348 mg/kg)	Female 310	470	16
Intermediate Dose (0.348 mg/kg)	Female 317	401	6
High Dose (3.48 mg/kg)	Male 321	561	109

While the reduction in platelet counts were not attributed as related to the test article, given the history of hematological adverse events associated with gold complexes the Sponsor organized a series of measures to explore the potential relationship to test article, including:

- Review of historical data from the minipig supplier and contact research organization (CRO) laboratory values for pre-dose and control minipigs to evaluate whether platelet reductions or low platelet counts had been previously observed.
- Specialty hematological assessment of bone marrow sections from these animals and repeat examination of bone marrow smears taken at necropsy.

Review of historical data sets revealed that the platelet count in the peripheral blood of the minipig is highly variable and low platelet counts have sporadically been observed in untreated minipigs at the CRO laboratory (Eurofins). Further examination of bone marrow smears revealed no treatment-related differences in group means or individual Myeloid:Erythroid (M:E) ratios. Female no. 317 had a low M:E ratio due to higher proportion of erythroid precursors, which correlated with the histopathological observation of a high cellularity of the bone marrow. These changes were consistent with a hyperplastic change of the erythroid compartment, not accompanied by any increase in reticulocyte count in the peripheral blood as might be expected. There were no cytological abnormalities correlating with the severe decrease in platelet counts observed at necropsy in treated female nos. 310 and 317 and at lower severity in male no. 321. It could not be excluded that the low platelet counts may be part of the background variation in this species or may have resulted from hemorrhage occurring accidentally at blood sampling. There was no evidence of qualitative or quantitative treatment-related effects on lymphoid cells, monocytes, reticulum, mast cells, or megakaryocytes. There were also no treatment-related differences in M:E ratio (group mean and individual values) or in absolute and relative counts of myeloid, erythroid, and lymphoid cell series.

To date, no significant platelet reductions have been observed in any of the toxicology studies conducted in rodents or canines.

2.3.2 Maximum Feasible Dose Study and High Dose Subchronic Toxicity Studies in Rodents and Minipigs

Upon submission of the Investigational New Drug (IND) application to the U.S. FDA, the Agency requested additional higher dose studies to identify a maximum tolerated dose, or alternatively a maximum feasible dose. Subsequently, a 7-day maximum feasible dose study was conducted in rats with subcutaneous (SC) dosing compared to 90 mg/kg oral gavage (OG) dosing. The maximal feasible dose (MFD) represented the highest concentration of CNM-Au8 (3000 parts per million, 3 mg/mL) that could be manufactured with currently available techniques dosed at the maximum permissible dosing volume delivered via gavage or SC administration. Of note, the 3 mg/mL concentration used in the two MFD studies had deviations out of product specification (hydrodynamic radius of 54.38 nm, which exceeded the production specification range of 10 – 30 nm) likely indicating nanoparticle agglomeration.

In rodents, doses of 5, 15, 30 and 90 mg/kg/day over seven consecutive days, were administered in a divided dose, i.e., twice daily subcutaneously (2.5, 7.5 and 15 mg/kg, BID) or, orally, three times daily (30 mg/kg TID; 90 mg/kg/day). The three times daily oral gavage administration was well tolerated by all rodents and not associated with any adverse findings. Test-article related findings that were not adverse, included discolored feces (black), decreased body weight gain and food consumption when compared to controls, and anatomic pathology findings of extramedullary hematopoiesis noted in the spleen and/or liver. This study defined 90 mg/kg/day as the NOAEL with oral gavage administration.

The daily subcutaneous administration was associated with adverse events related to subcutaneous administration of the test article. The skin was thickened/leathery in all treatment groups with associated changes in clinical pathology, defined by increased neutrophils and fibrinogen levels and decreases in albumin; and anatomic pathology, defined by subacute/chronic inflammation, edema, and cavitation in the injection sites, which were considered adverse in their totality. Systemic test article-related changes not considered adverse were noted in body weights (decreased gain), food consumption (decreased), and anatomic pathology (increased spleen weights, and extramedullary hematopoiesis in the spleen and liver). Accordingly, a NOAEL could not be established in the rodents receiving daily SC administration up to 30 mg/kg.

With twice-daily SC administration of CNM-Au8, blood Au exposures increased proportionally or slightly greater than proportionally from 5 to 30 mg/kg/day on Day 1 and Day 7. Blood Au exposures after SC administration were considerably higher than after oral gavage administration with the dose normalized $AUC_{(0\text{-Last})}$ ratio (SC:OG) ranging from approximately 9x – 50x higher dose-normalized

AUC depending upon animal gender and the SC dose. Despite substantially greater Au accumulation, no systemic toxicities were observed with SC administration.

Table 8 below summarizes the design and results of the maximum feasible dose and subsequent high dose subchronic toxicity studies in rodents.

Table 8. Maximum Feasible Dose Study and High Dose Subchronic Toxicity Studies

**Study ID** (Internal ID/ CRO ID)	**GLP Status**	**Species** (N)	**Brief Design** [Route; Frequency]	**Nominal Dose** (mg/kg/day)	**CNM-Au8 Conc.** (µg/mL)	**Safety Summary/ Toxicity Notes**
CNAU-MPI-14-04 (2257-004)	Non-GLP	Sprague-Dawley Rat (N=60; 12/group)	7-Day Max Feasible Dose Study [SC; BID] [OG; TID]	5 [SC; 2.5 mg/kg BID]	500	Discolored feces (black), decreased body weight gain and food consumption; non-adverse anatomic pathology findings consisting of extramedullary hematopoiesis noted in the spleen and/or liver. Skin thickened/leathery in SC injection groups associated with changes in clinical pathology (increased neutrophils and fibrinogen levels; decreases in albumin) and anatomic pathology (subacute/ chronic inflammation, edema, and cavitation in the injection sites).
				15 [SC; 7.5 mg/kg BID]	1,500	
				30 [SC; 15 mg/kg BID]	3,000	
				90 [OG; 30mg/kg, TID]	3,000	
CNM-Au8-MPI-16-01 (2257-008)	GLP	CD Rat (N=146)	21-Day Repeat Dose Toxicity [OG; QD/BID]	10	1,000	No treatment-related signs of toxicity; no effects on body weights, food consumption or clinical pathology parameters; no treated related ophthalmologic effects; examinations; no organ weights changes; no treatment-related observations during necropsy and no histopathologic changes.
				20	1,000	
				40 [20mg/kg, BID]	1,000	

2.3.3 Chronic Rodent Toxicology (6-Month Dosing)

The objective of this study was to evaluate the toxicity of the test article in rats after administration for 13- and 26-weeks and to evaluate reversibility, progression, or delayed appearance of any observed changes following a 4-week postdose observation period after each dosing termination.

Assessment of toxicity was based on mortality, clinical observations, body weight, and food consumption; ophthalmoscopic examinations; and clinical and anatomic pathology. Toxicokinetic (TK) assessment [whole blood, urine, cerebral spinal fluid (CSF), and designated frozen tissues] was conducted for gold content.

There were no definitive test article-related mortalities and no test article-related effects on body weight, food consumption, ophthalmoscopic examinations; clinical pathology, macroscopic examinations and organ weights. Based on the data from the detailed clinical observations, body weight, food consumption, clinical and anatomical pathology, oral doses of CNM-Au8 at 10, 20, and 40 mg/kg/day resulted in no adverse findings of these rats. Therefore, the NOAEL for this study was considered to be 40 mg/kg/day, the highest dose tested.

Table 9. Chronic Rodent Toxicity Study

**Study ID** (Internal ID/ CRO ID)	**GLP Status**	**Species** (N)	**Brief Design** [Route; Frequency]	**Nominal Dose** (mg/kg/day)	**CNM-Au8 Conc.** (µg/mL)	**Safety Summary/ Toxicity Notes**
CNM-Au8-MPI-16-04 (2257-006)	GLP	CD Rat (N=306)	26-Week Repeat Dose Toxicity with 4-Week Recovery [OG; QD] [OG; BID]	10 [OG QD]	1,000	There were no definitive test article-related mortalities. No test article-related effects on body weight, food consumption, ophthalmoscopic examinations; clinical pathology, organ weights macroscopic and microscopic examinations.
				20 [OG QD]	1,000	
				40 [OG; 20 mg/kg BID]	1,000	

2.4 Summary of Toxicology and Toxicokinetic Studies in Canines

Five toxicokinetic studies have been conducted in Beagle dogs including:

- Single Dose Toxicokinetic Study
- Maximum Tolerated Dose study (7-Day)
- 28-Day Subchronic Repeat Dose Toxicokinetic Study
- 21-Day Maximum Feasible Dose Study
- 9-Month Chronic Toxicity Study

Doses of CNM-Au8 up to 90 mg/kg by oral gavage have not demonstrated any acute or subchronic toxicity, and accordingly, a MTD has not been identified to date. All dosing levels and all concentrations (ranging from 350 – 3,000 µg/mL) explored in canines have produced NOAELs.

Table 10 below summarizes the design and results of the toxicokinetic studies conducted to date in Beagle dogs.

Table 10. Acute and Subchronic Dosing Studies in Canines

**Study ID** (Internal ID/ CRO ID)	**GLP Status**	**Species** (N)	**Brief Design** [Route; Frequency]	**Nominal Dose** (mg/kg/day)	**CNM-Au8 Conc.** (µg/mL)	**Safety Summary/ Toxicity Notes**
CNAU-MPI-14-01 (2257-001)	GLP	Beagle Dog (N=18; 6/group)	Single Dose Toxicity and Toxicokinetics (With 9 Day Follow-Up)	0.5 mg/kg IV	100	No mortality and no clinical observations noted during study.
				3.5 mg/kg [OG, QD]	350	
				10 mg/kg [OG, QD]	1,000	
CNAU-MPI-14-02 (2257-003)	GLP	Beagle Dog (N=48; 12/group)	28-Day Repeat Dose Toxicity [OG, QD]	0.35	35	No mortality; no treatment-related toxicity; no effects on body weights, food consumption or clinical pathology parameters; no treatment-related effects on ophthalmologic or physical examinations; no effects on organ weights or histopathologic changes
				3.5	350	
				10	1,000	
CNAU-MPI-14-03 (2257-002)	Non-GLP	Beagle Dog (N=8; 2/group)	Ascending Dose and 7-Day Repeat Dose Toxicity [OG, QD]	10	1,000	No mortality and no clinical observations noted during study; discolored feces were observed in all treated animals; no related treatment effects on food consumption, body weight, or physical exam.
				20	2,000	
				30	3,000	
CNAU-MPI-14-05 (2257-005)	GLP	Beagle Dog (N=24; 6/group)	21-Day Repeat Dose Toxicity (Maximum Feasible Dose) [OG; QD, BID, TID]	30 [30mg/kg QD]	3,000	No mortality; treatment-related salivation and discolored feces at 60 and 90 mg/kg/day. No treatment-related effects on body weights, food consumption, ophthalmology, electrocardiograms, clinical pathology parameters, organ weights or macroscopic examinations. Non-adverse changes in the kidneys in three animals at 90 mg/kg/day with minimal bilateral tubular cell hypertrophy, characterized by a diffuse increase in the size of tubular epithelial cells.
				60 [30mg/kg BID]	3,000	
				90 [30mg/kg TID]	3,000	

2.4.1 Single Dose Canine Toxicokinetic Summary

After intravenous (IV) administration, the mean CNM-Au8 whole blood estimated concentration at time zero (C_0) was calculated as 9.39 ng/mL (range 4.97 to 18.4 ng/mL) extrapolated using a log-

linear regression from the first two measured concentrations. The first blood sample after IV dosing was collected at 30 minutes postdose, so it is possible that the concentration at 30 minutes underestimates C_0. After oral administration, maximum CNM-Au8 plasma concentrations were observed at a T_{max} range of 4 – 48 hours and 12 – 48 hours postdose for the 3.5 and 10 mg/kg cohorts, respectively.

Due to limited interpretable data points above the lower limit of quantitation (LLOQ) in the IV dosing group, AUC_{∞} was not calculated, and absolute bioavailability (F) was therefore calculated using the mean values for $AUC_{0\text{-}336}$ from two of six animals with quantifiable Au whole blood concentrations to 336 hours. As shown in Table 11, F was 30.5% for the 3.5 mg/kg group and 20.5% for the 10 mg/kg group. The difference may be due to the use of different animals in the 3 cohorts rather than a true difference in bioavailability. The mean C_{max} value at 3.5 mg/kg includes two animals with CNM-Au8 concentrations all below the lower limit of quantitation with subsequent C_{max} values set to zero, so the reported C_{max} value may be an underestimate in this dosing group.

Table 11. Summary of C_{max}, AUC, $T_{1/2}$, and Bioavailability Results Following Single Dose Administration of CNM-Au8 in Study CNAU-MPI-14-01

CNM-Au8 Dose (mg/kg)	Statistic	C_{max} or C_0 (ng/mL)	$AUC_{0\text{-}336hr}$ (hr*ng/mL)	$AUC_{0\text{-}336/Dose}$ (kg*hr*ng/mL/mg)	$T_{1/2}$ (hr)	Bioavailability (F%) (Dose Normalized AUC Ratio (PO/IV)
0.5 (IV)	N	6	2	2	2	N/A
	Mean	9.39	146	291	31.0	
	SD	5.25	N/A	N/A	N/A	
	CV%	55.9	N/A	N/A	N/A	
**3.5 (PO)**	N	6	4	4	N/A Insufficient Data)	30.5%
	Mean	1.80	311	88.8		
	SD	1.65	234	66.8		
	CV%	92%	75%	75%		
**10.0 (PO)**	N	6	5	5	4	20.5%
	Mean	4.04	596	59.6	150	
	SD	2.99	457	45.7	51.5	
	CV%	74%	77%	77%	34%	

The urine volume and feces mass in each collection interval was not determined, therefore, comparisons among dosing groups were limited to urine and fecal concentration data. In the IV dosing group, feces concentrations were all below the LLOQ (<150 ng/mL); however, identification of CNM-Au8 above the lower limit of detection (LLOD) in the feces over the 2-week period established the feces as a route of elimination. Quantifiable urine concentrations were observed between 36 to 48 hours postdose in the 0.5 mg/kg IV dosing group, confirming urine as an elimination pathway. Urinary excretion data were not collected at consistent intervals with whole blood concentration, so renal clearance could not be calculated. Peak urine concentrations in the 3.5 and 10

mg/kg groups occurred between 12 – 24 hours and 36 – 48 hours, respectively, and exceeded whole blood concentrations.

Mean values for half-life ($T_{1/2}$), clearance (CL), volume of distribution (V_z), and volume of distribution at steady state (V_{ss}), respectively, for the 0.5 mg/kg IV dosing group are shown in Table 12.

Table 12. Summary of CL, $T_{1/2}$, V_z, and V_{ss} after a Single 0.5 mg/kg IV Dose of CNM-Au8 (Study CNAU-MPI-14-01)

CNM-Au8 Dose	Statistic	$T_{1/2}$ (hr)	CL (L/hr/kg)	V_z (L/hr/kg)	V_{ss} (L/hr/kg)
0.5 mg/kg (IV)	N	2	2	2	2
	Mean	31.0	5.64	249	242
	$Animal_{102}$	33.6	4.72	229	224
	$Animal_{105}$	28.5	6.57	270	261

In the oral gavage dosing groups, dose normalized systemic exposure ($AUC_{0\text{-}336hr/Dose}$) and C_{max} values for CNM-Au8 showed less than proportional increases. An approximate 1:3-fold increase in dose (3.5 mg/kg to 10 mg/kg) resulted in an approximate 1:1.5-fold decrease in mean dose normalized $AUC_{0\text{-}336hr/Dose.}$ Mean values for terminal half-life ($T_{1/2}$), oral clearance (CL/F), and oral volume of distribution (V_z/F), for the 10 mg/kg dosing group are reported in Table 13.

Table 13. Summary of CL/F, $T_{1/2}$, and Vz/F after a Single 10 mg/kg PO Dose of CNM-Au8 (Study CNAU-MPI-14-01)

CNM-Au8 Dose	Statistic	$T_{1/2}$ (hr)	CL/F (L/hr/kg)	V_z/F (L/hr/kg)
10 mg/kg (PO)	N	4	4	4
	Mean	150	13.1	2,970
	SD	51.5	7.3	2,080
	CV%	34%	56%	70%

2.4.2 Repeat Dose Canine Toxicokinetic Summary

In brief, in the subchronic toxicity study with a 4-week recovery period, 48 beagles were dosed for 28-days to assess toxicity and toxicokinetics. CNM-Au8 was well tolerated when administered at gold concentrations of 0.35, 3.5, and 10 mg/kg/day as a single daily dose of 1mg/mL CNM-Au8 via oral gavage, and the10 mg/kg/day dose was considered the NOAEL. There were no significant changes in serum chemistry or hematology measures or in the macroscopic or histologic assessments post-necropsy. In terms of toxicokinetics, systemic exposure appeared independent of gender. There was an increase in C_{max} from 0.35 to 3.5 mg/kg, but no apparent change from 3.5 to 10 mg/kg, suggesting a plateau in exposure to 3.5 and 10 mg/kg. Mean systemic exposure $AUC_{(0\text{-}24\text{ hr})}$ accumulated in whole blood following repeated administration over the first two weeks, with no significant change in accumulation ratio (R_{ac}) after that time, indicating that steady-state was reached

within 2 weeks of dosing. Elimination half-life ($T_{1/2}$) was long; 333 hours (13.9 days) in the 10 mg/kg dose group. During the recovery elimination phase, gold levels were still quantifiable at 4-weeks post-dosing in both blood and tissue. Whole blood Au concentrations are shown in Figure 10.

Figure 10. Whole Blood Concentrations by Day in 28-Day Repeat Dosing Canine Study

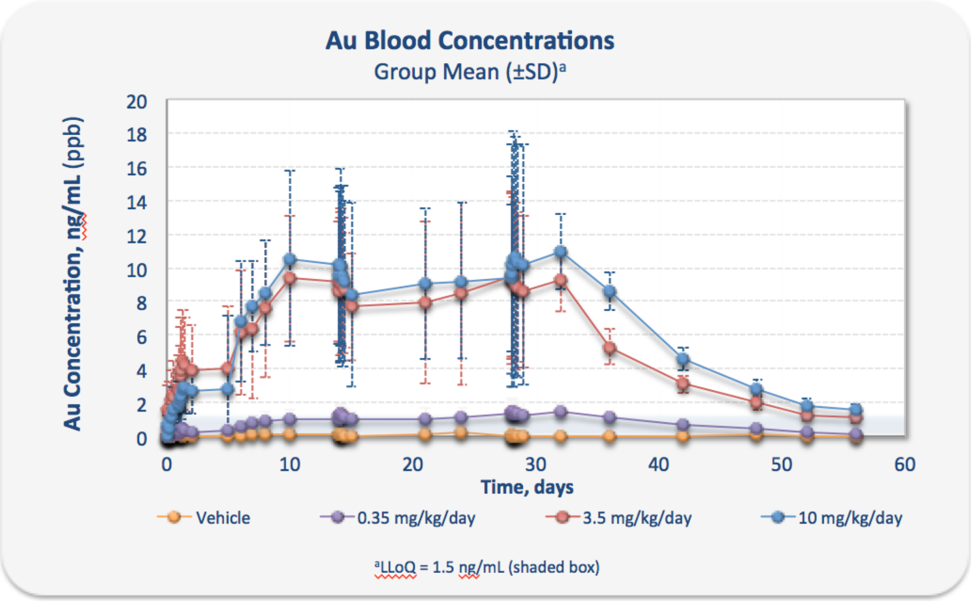

A noteworthy finding in the three-animal species studied was a disproportionate tissue concentration of gold measured in the kidney. This renal accumulation was not accompanied by changes in renal function, as measured by serum biochemistry, urinalysis, or structural changes identified in the histologic examination of the kidney. In the 4-week recovery period in the 28-day dog study, gold concentrations in the kidney decreased significantly over that recovery period, and continuing urinary excretion of gold was measured throughout the recovery period.

2.4.3 Maximum Feasible Dose Canine Toxicokinetic Study

In the canine maximum feasible dose study, the doses administered were 30 mg/kg/day (QD), 60mg/kg/day (30 mg/kg BID), or 90 mg/kg/day (30 mg/kg TID) over three consecutive weeks (21-days). Dosing was well tolerated in the dogs over the 21 consecutive days of dosing. At the highest dose, 90mg/kg/day, minimal bilateral tubular cell hypertrophy was observed in the kidneys of males and females but was considered non-adverse by the MPI study pathologist-toxicologist.

No deaths occurred. Treatment-related salivation and black discolored feces were observed at 60 and 90 mg/kg/day. No treatment-related effects were observed on body weights, food consumption, ophthalmology, ECGs, clinical pathology parameters, organ weights or macroscopic examinations. There was slightly less than a proportional increase in gold exposure when the dose was increased from 30 to 60 mg/kg/day. Increasing the dose from 60 to 90 mg/kg/day was not generally accompanied by an increase in gold exposure. Thus, maximal systemic exposure in the dogs may

occur with a dose of 60mg/kg/day divided into two daily oral doses of 30 mg/kg. Accumulation appeared to plateau by Day 7, with no substantive increase in exposure on Days 14 and 21. Blood gold exposures at steady-state were approximately 1.1 to 3.3 times higher than on Day 1, and the extent of the accumulation tended to be lower with higher doses. Systemic gold concentration appears to be approximately at steady state after 1 to 2 weeks of dosing. Systemic exposure appears to be higher in female dogs than in male dogs.

2.4.4 Chronic Canine Toxicology (9-Month Dosing)

The objective of this study was to evaluate the potential toxicity of the test article in beagle dogs when administered orally via gavage once daily for 13 and 39 weeks, and to evaluate reversibility, progression, or delayed appearance of any observed changes following a 4-week post dose observation period after each termination.

Assessment of toxicity was based on mortality, clinical observations, body weight, and food consumption; ophthalmoscopic, and electrocardiographic examinations; and clinical and anatomic pathology. Blood, urine, CSF, and designated frozen tissues were analyzed for gold content. Toxicokinetic assessment for gold content in blood and urine was conducted.

No early deaths occurred. There were no treatment-related clinical signs of toxicity observed, no effects on body weight or food consumption, or during ophthalmoscopic observations or electrocardiographic examinations. There were no test article-related effects among clinical pathology parameters noted during the interim dosing and recovery periods. No organ weight changes were observed and no test article related macroscopic or microscopic findings were observed at the interim or recovery necropsies.

CNM-Au8 was well tolerated in dogs when administered at doses of 0.35, 3.5 and 10 mg/kg/day for 39-weeks. No adverse signs of toxicity were observed at any dose level. Therefore, the dose of 10 mg/kg/day was considered the NOAEL in dogs following oral administration.

Table 14. Chronic Canine Toxicity Study

**Study ID** (Internal ID/ CRO ID)	**GLP Status**	**Species** (N)	**Brief Design** [Route; Frequency]	**Nominal Dose** (mg/kg/day)	**CNM-Au8 Conc.** (µg/mL)	**Safety Summary/ Toxicity Notes**
CNM-AU8-MPI-16-03 (2257-007)	GLP	Beagle Dog (N=80; 8 or 12 (TK)/ group)	39-Week Repeat Dose Toxicity with 4-Week Recovery [OG; QD]	0.35 [OG, QD]	1,000	No treatment-related clinical signs of toxicity observed, no effects on body weight or food consumption, or during ophthalmoscopic observations or electrocardiographic examinations. There were no test article-related effects among clinical pathology parameters noted during the interim dosing and recovery periods. No organ weight changes were observed and no test article related macroscopic or microscopic findings were observed at the interim or recovery necropsies.
				3.5 [OG, QD]	1,000	
				10 [OG, QD]	1,000	

3 PRIOR HUMAN DOSING EXPERIENCE

A Phase 1, First-Time-In-Human study was previously conducted under a clinical trial application (CTA) in the Netherlands (AU8.1000-14-01). The study was a 2-phase, randomised, placebo-controlled, double-blind, single- and multiple-dose escalating study to evaluate the safety, tolerability, and PK of CNM-Au8 in healthy male and female volunteers. There were 2 phases to this study: a single ascending dose (SAD) Phase and a multiple ascending dose (MAD) Phase. The SAD Phase was conducted first, followed by the MAD Phase of the study.

3.1 AU8.1000-14-01 Design

A total of 8 subjects per cohort were randomly assigned (3:1 ratio) to receive a single dose of either CNM-Au8 (n = 6) or placebo (n = 2) at an initial dose level of 15 mg CNM-Au8. Additional cohorts of 8 subjects were enrolled to investigate single-escalating doses of CNM-Au8 at 30 mg, 60 mg, and 90 mg. Cohorts were to be balanced by gender with no more than two-thirds of subjects being of one gender. Safety parameters included findings from electrocardiograms (ECGs), vital signs, clinical laboratory panels, physical examinations, urinalysis, AEs, and concomitant medications. A follow-up visit was conducted for final safety assessments on Day 17 ± 1.

After the SAD Phase was completed, a total of 12 subjects per cohort were randomly assigned (3:1 ratio) to receive a multiple dose of either CNM-Au8 (n = 9) or placebo (n = 3) once daily for 21 days at an initial dose level of 15 mg CNM-Au8. Additional cohorts of 12 subjects were enrolled to investigate multiple escalating doses of CNM-Au8 at 30 mg, 60 mg, and 90 mg. Cohorts were balanced by gender, with no more than two-thirds of subjects being of one gender. Following the final dose of study drug on Day 21, subjects entered a 28-Day follow-up phase and returned to the clinical research unit (CRU) for PK sampling on Days 23, 24, 25, 26, 27, 28, 32, 36, 40, and 49, which completed study participation. Safety parameters included findings from ECGs, vital signs, clinical laboratory panels, physical examinations, urinalysis, AEs, and concomitant medications.

3.2 AU8.1000-14-01 Pharmacokinetic Results

The PK Population included all randomised subjects who received CNM-Au8 study drug and had sufficient samples collected for estimation of PK parameters.

- The PK Population for the SAD Phase included 24 subjects who received a single dose of CNM-Au8 study drug at 15 mg, 30 mg, 60 mg, and 90 mg.

- The PK Population for the MAD Phase included a total of 35 subjects: 8 subjects in the 15 mg dosing group and 9 subjects each in the other 3 cohorts who received multiple doses of CNM-Au8 study drug at 30 mg, 60 mg, and 90 mg (Cohorts 5–8).

In the SAD Phase, PK analyses were not completed since the majority of whole blood Au concentrations were below the LLOQ. Since the lack of quantitation of Au concentrations may have been related to ICP-MS assay sensitivity, the Sponsor upgraded to an advanced, more sensitive, ICP-MS instrument for the PK analyses in the MAD Phase.

In the MAD Phase:

- The geometric mean whole blood concentrations from 1 week onward increased in a dose-related, but not a dose-proportional manner. Based on Days 14 and 21, the increases in both C_{max} and $AUC_{(0-24)}$ were less than dose proportional.
- Based on pre-specified fit criteria, the elimination $t_{½}$ ranged from 277 to 628 hr (11.5 to 26.2 days).
- Steady-state for all cohorts, based on the geometric mean whole blood concentrations, was reached by the end of the second week of dosing (Day 14), which was substantially less than predicted by the $t_{½}$ [range of 46 to 105 days (6.6 to 15 weeks)].

PK parameters related to urinary excretion (Ue, CL_r) could not be calculated. Only 1 urine Au concentration ≥ LLOQ (3 ng/mL) was within limits of the assay.

3.3 AU8.1000-14-01 Safety Results

The Safety Population included randomised subjects who received at least 1 dose of study drug and had at least 1 post-baseline safety assessment, and was used to perform all safety analyses. The Safety Population included 40 subjects in the SAD Phase, 46 subjects in the MAD Phase, and 86 subjects in the Pooled (SAD + MAD) group.

All planned dose cohorts were completed, and the Safety Review Committee (SRC) agreed to escalate to each subsequent dose cohort per the protocol. An MTD was not identified. All subjects in the SAD Phase completed a single oral dose per protocol. In the MAD Phase, the mean duration of treatment was 21.0 days of consecutive oral dosing for the 15 mg, 30 mg, and 60 mg Cohorts and placebo. Subjects in the 90 mg Cohort received on average 20.1 days of consecutive dosing. One subject (subject 0085-MAD 90 mg) in the MAD Phase did not complete the 21 days of consecutive dosing per protocol due to a reported pregnancy after 13 days of consecutive dosing, and had a medically induced abortion 7 days later.

For the SAD and MAD Phases of the study, the overall incidence of TEAEs was comparable between treatments, including placebo; overall incidence of TEAEs considered related to study drug by the Investigator was also comparable between treatments, including placebo. The most frequently reported TEAEs were in the classes of Nervous system disorders and Gastrointestinal disorders. The majority of TEAEs were Grade 1 severity (mild). There were no serious TEAEs, TEAEs leading to discontinuation of treatment, or TEAEs considered severe, life threatening, or resulting in death. Overall, no dose response relationship to TEAEs was observed in the SAD or MAD Phase of the study; however, the frequency of headache and gastrointestinal TEAEs was higher in the 90 mg MAD treated subjects.

In the SAD Phase, TEAEs considered related to study drug by the Investigator were reported by 2 (16.7%) out of 12 subjects in the 15 mg group, 2 (33.3%) out of 6 subjects in the 30 mg group, 4 (66.7%) out of 6 subjects in the 60 mg group, and 5 (83.3%) out of 6 subjects in the 90 mg group compared with 7 (70.0%) out of 10 subjects in the placebo group. All related TEAEs were Grade 1 severity (mild) in the SAD Phase, except for 2 related TEAEs (diarrhea and abdominal pain: both in Subject 0024-SAD 60 mg) that were considered Grade 2 severity (moderate).

In the MAD Phase, TEAEs considered related to study drug by the Investigator were reported by 5 (62.5%) out of 8 subjects in the 15 mg, 4 (44.4%) out of 9 subjects in the 30 mg, 3 (33.3%) out of 9 subjects in the 60 mg, 8 (88.9%) out 9 subjects in the 90 mg, and 6 (54.5%) 11 subjects in the placebo group. All related TEAEs in the MAD Phase were Grade 1 severity (mild).

Modified treatment emergent events were defined as a treatment emergent adverse event that started within 24 hours from first dose of study medication. Modified TEAEs for all CNM-Au8 doses were reported by 11 (36.7%) out of 30 subjects in the SAD Phase, 7 (20.0%) out of 35 subjects in the MAD Phase, and 18 (27.7%) out of 65 subjects in the Pooled (SAD + MAD) group. A total of 9 (42.9%) out of 21 placebo treated subjects reported modified TEAEs. The most frequently reported modified TEAEs included headache, blood creatinine increased, dizziness, diarrhea, and nausea.

The Sponsor performed a pre-specified search of TEAE preferred terms potentially associated with gold complex compounds to determine adverse events of special interest (AESIs). All AESIs were considered Grade 1 severity (mild), except for 2 TEAEs of Grade 2 severity (moderate): diarrhea and abdominal pain in the same subject (Subject 0024-SAD 60 mg). Incidence rates were comparable between all dosing groups including placebo.

Dosing with either a single dose or multiple doses of CNM-Au8 was generally well tolerated over the course of this study. The most frequently reported TEAEs in the pooled population were headache, somnolence, fatigue, abdominal pain upper, diarrhea, nausea, abdominal pain, and dizziness. Overall

for both the SAD and MAD Phases, routine clinical laboratory assessments (hematology, serum chemistry, and urinalysis), vital signs, ECGs, and physical examinations did not reveal clinically notable findings; there were no serious TEAEs or TEAEs leading to discontinuation of treatment. No safety trends or safety signals were observed in this study.

Oral administration of either a single (SAD) or repeated dose (MAD) of CNM-Au8 over 21 consecutive days in healthy volunteers was safe and well tolerated. The TEAEs observed across the 4 doses (15 mg, 30 mg, 60 mg, and 90 mg) of CNM-Au8 were mostly mild in severity. While no dose response relationship to TEAEs was observed overall in the SAD or MAD Phase of the study, increases in headache and gastrointestinal related TEAEs were observed in the 90 mg cohort of the MAD Phase.

Overall, for both the SAD and MAD Phases, routine clinical laboratory assessments (hematology, serum chemistry, and urinalysis), vital signs, ECGs, and physical examinations did not reveal clinically notable findings or trends; none resulted in serious TEAEs or TEAEs leading to discontinuation of treatment.

4 INVESTIGATIONAL PLAN

4.1 Rationale for the Study

CNM-Au8 has been studied in several preclinical ALS rodent disease models and has demonstrated significant neuroprotection in human iPSC derived neuronal populations. Through a mechanism of action targeting restoration of cellular redox homeostasis, CNM-Au8 addresses pathophysiologic bioenergetic failure in neurons, which may underlie the pathogenesis of ALS and other neurodegenerative diseases. CNM-Au8 acts catalytically to affect cellular bioenergetics protecting sensitive neuronal populations through a unique mechanism promoting cellular redox homeostasis that involves the catalytic oxidation of NADH resulting in enhanced aerobic glycolytic energy production.

4.2 Study Objectives

To assess the efficacy, safety, and PK/PD effects of CNM-Au8 as a disease-modifying agent for the treatment of ALS by utilizing electrophysiological measures to detect preservation of motor neuron function.

- Efficacy will be assessed as the difference in disease progression from baseline reflected by changes in motor neuron loss measured by electromyography (e.g., MUNIX, MUSIX, Split Hand Index, Neurophysiology Index, MScanFit), change in ALS Functional Rating Score-Revised (ALSFRS-R score), the mean rate of change (ΔFS) of the ALSFRS-R from symptom onset, survival status, respiratory functions, and composite disease progression.
- Safety will be assessed up through the frequency of treatment emergent adverse events, serious adverse events, discontinuations due to adverse events, Falls Questionnaire and the Columbia Suicidality Severity Rating Scale (C-SSRS).
- Pharmacodynamics and pharmacokinetics (PK/PD) will be assessed through blood and urine collection at Baseline and then every 12-weeks thereafter during the treatment period and every 24-weeks during the optional open-label extension period. PD analyses may include whole blood metabolomic markers (e.g., redox coenzymes: NAD+, NADH, NADP+, NADPH; energetic coenzymes: ATP, ADP, AMP and antioxidants: GSSG and GSH), urinary neurotrophin receptor $P75^{ECD}$ levels, and serum neurofilament light chain (NfL).

4.3 Overall Study Design and Plan

This is a multi-centre randomised, double-blind, parallel group, placebo-controlled study of the efficacy, safety, pharmacokinetics, and pharmacodynamics of CNM-Au8 in patients who are newly

symptomatic within 24-months of Screening and with a clinically probable or possible or definite ALS diagnosis per the Awaji-Shima criteria (de Carvalho et al., 2008).

Patients may be screened over up to a 6-week period. Patients who meet the inclusion criteria and none of the exclusionary criteria will be enrolled into the clinical study.

Patients will be randomised 1:1 into one of two groups: either active treatment with CNM-Au8 30 mg or Placebo. All patients will receive their randomised oral treatment daily over thirty-six (36) consecutive weeks during the Treatment Period. For those who complete the Treatment Period and meet the required inclusion and exclusion criteria, an optional open-label extension period will be available for interested participants for up to and additional 48 weeks, which may be extended for additional 12-week increments until discontinued by the Sponsor.

There will be four study periods:

1. Up to a six (6) week screening period (Screening Period);
2. A thirty-six (36) week blinded randomised treatment period (Treatment Period);
3. An optional open-label extension period (Open-Label Period) that will remain active until discontinued by the Sponsor;
4. A four (4) week safety follow-up period following completion of either the Treatment or Open-Label period or in the case of Early Termination (Safety Follow-Up Period).

Per protocol all patients will receive their blinded and randomised oral treatment daily over at least 36 consecutive weeks during the Treatment Period. Following the end of the Treatment Period patients may elect to participate in the optional open-label extension (OLE) period. All participants will complete a Safety Follow-Up Visit four (4) weeks following their last dose of study medication.

An independent DSMB will be responsible for monitoring the safety of the study on a periodic basis (e.g., Q16 weeks) and ad hoc at the request of the DSMB or the Sponsor (e.g., in the event of unexpected SAEs) to review data throughout the Treatment and OLE Periods. The DSMB may make recommendations on the conduct of the study, including study termination. Appropriate procedures will be detailed in a DSMB Charter.

The study scheme is shown below in Figure 11.

Figure 11. Study Scheme

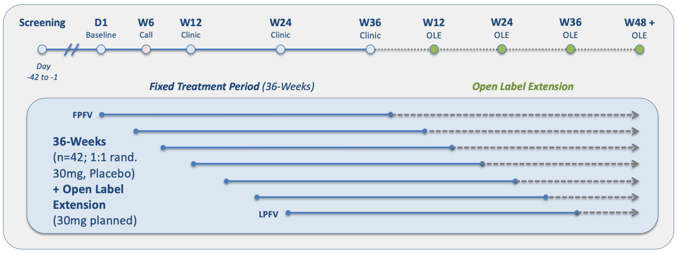

5 INVESTIGATORS AND KEY STUDY PERSONNEL

Key study personnel and investigators include the following:

- Sponsor's Medical Expert:

Name: Robert Glanzman, MD, FAAN
Title: Chief Medical Officer
Address: 6550 S. Millrock Drive
Suite G50
Salt Lake City, Utah 84121
United States
Email: robert@clene.com
Telephone: +1 (801) 676 –9695

- Chief Investigator

Name: A/Professor Parvathi Menon PhD
Title: Consultant Neurologist, Associate Professor in the Faculty of Medicine University of Sydney
Address: Brain and Nerve Research Centre
Concord Repatriation General Hospital
Building 20, Hospital Road
Concord NSW 2139
Australia
Email: parmenon2010@gmail.com
Telephone: +61 (2) 9767 8423

- Chief Investigator

Name: Dr. William Huynh
Title: Consultant Neurologist
Address: Brain and Mind Centre
94 Mallet Street,
Camperdown NSW 2050
Australia
Email: Email: william.huynh@sydney.edu.au
Telephone: +61 (2) 9114 4250

All other study personnel not included in this section are identified in a separate personnel list (not part of this clinical study protocol) as appropriate. This list will be updated as needed; an abbreviated version with personnel relevant for the centers will be available in each center's Investigator site file.

Whenever the term 'Investigator' is noted in the protocol text, it may refer to either the Principal Investigator at the site or an appropriately qualified, trained and delegated individual of the investigational site.

The Principal Investigator of each site/study center must sign the protocol signature sheet before

patient recruitment may occur at the respective center. Similarly, all protocol amendments and/or revised integrated protocols must be signed and dated by the site's Principal Investigator before coming into effect at the respective center. A complete list of all participating centers and their investigators, as well as all required signature documents, will be maintained in the Sponsor study file.

Additional investigators and study sites may be added at the discretion of the Sponsor.

6 STUDY ENDPOINTS

6.1 Efficacy Endpoints (Treatment Period)

6.1.1 Primary Efficacy Endpoint

The primary study endpoint is the change in the average difference between active treatment and placebo from Baseline through Week 36 for the summed MUNIXscore(4), which is the sum of the respective MUNIX values for the Abductor Digiti Minimi (ADM), Abductor Pollicis Brevis (APB), Biceps Brachii (BB), and Tibialis Anterior (TA). The average baseline summed values will be indexed to 100% for each participant. Changes will be calculated as the percent change from the Baseline index of 100%.

Electromyography measurements for the MUNIX assessment will be taken in the least-clinically affected hand or limb, as applicable, identified at the Baseline visit.

6.1.2 Secondary Efficacy Endpoint

Key secondary efficacy endpoints include both a functional clinical endpoint and an electromyography endpoint as described below. The endpoints will be assessed hierarchically as follows:

a. Mean change of the average difference between active treatment and placebo from Baseline to Week 36 for respiratory function as measured by Forced vital capacity (FVC).

b. Mean absolute change of the difference between active treatment and placebo from Baseline through Week 36 for the MUNIXscore(4), which is the sum of the respective MUNIX values for the Abductor Digiti Minimi (ADM), Abductor Pollicis Brevis (APB), Biceps Brachii (BB), and Tibialis Anterior (TA).

6.1.3 Exploratory Efficacy Endpoints

Exploratory endpoints include both electromyography and functional clinical endpoints as described below. The hierarchical assessment and final exploratory endpoints will be detailed in the statistical analysis plan.

6.1.3.1 Exploratory Electromyography Endpoints

Exploratory efficacy endpoints of electromyographic measurements of disease progression include assessment of Motor Unit Size Index (MUSIX), MScanMUNE, Split Hand Index (SI), and the Neurophysiological Index (NPI).

6.1.3.1.1 MScanFit MUNE

Mean change in the average difference between active treatment and placebo from Baseline through

Week 36 (overall difference at all time points) as measured by MScanFit MUNE (Jacobsen et al., 2017) of the APB in the least clinically affected hand. The baseline average will be indexed to 100%, and changes at Week 12, 24 and Week 36 will be calculated as the percent change from the Baseline index of 100%.

6.1.3.1.2 MUSIX

Mean change in the average difference between active treatment and placebo from Baseline through Week 36 for the MUSIXscore(4) (Neuwirth et al., 2015), which is the mean of the respective MUSIX values for the ADM, APB, BB, and TA. The average baseline mean values will be indexed to 100%. Changes will be calculated as the percent change from the Baseline index of 100%.

6.1.3.1.3 Split Hand Index

Mean change in the average difference between active treatment and placebo from Baseline through Week 36 for the Split Hand Index (SI) (Menon et al., 2013), defined as the first dorsal interosseous $(FDI)^{\text{Peak CMAP Amplitude}} * APB^{\text{Peak CMAP Amplitude}}/ADM^{\text{Peak CMAP Amplitude}}$.

6.1.3.1.4 Neurophysiological Index

Mean change in the average difference between active treatment and placebo from Baseline through Week 36 for the Neurophysiological Index (NPI) of the ADM (Cheah et al., 2011), defined as the ulnar nerve $(ADM^{\text{CMAP Peak Amplitude}} / ADM^{\text{Distal Motor Latency}}) * ADM^{\text{F-Wave (\%)}}$.

6.1.3.2 Exploratory Clinical Efficacy Endpoints

Exploratory clinical efficacy endpoints will include the following assessments:

- Mean change between active treatment and placebo in the proportion of patients experiencing a ≥ 6-point decline in the ALSFRS-R between active treatment and placebo from Baseline to Week 36.
- Mean change in average ALSFRS-R score from Baseline to Week 36.
- Mean change in slope of the decline of the ALSFRS-R from Baseline to Week 36.
- Mean change between active treatment and placebo for the Combined Assessment of Function and Survival (CAFS) (Berry et al., 2013), a joint-rank analysis of function (ALSFRS-R) and overall survival from Baseline to Week 36.
- Mean change between active treatment and placebo from Baseline to Week 36 in the proportion of patients experiencing ALS clinical composite disease progression defined as the occurrence of

death, tracheostomy, use of non-invasive ventilatory respiratory support, insertion of a gastrostomy tube, or a 6-point drop in the ALSFRS-R score.

- Mean change in rate of disease progression defined as the average change in the ΔFS score ([Max ALSFRS-R minus current ALSFRS-R score]/symptom duration in months) from Baseline to Week 36.
- Mean change in average difference between active treatment and placebo from Baseline to Week 36 for:
    - ALSSQOL-Short Form questionnaire (ALSSQOL-SF)
    - Clinician's Global Impression (CGI)
    - Patient's Global Impression (PGI)
- Difference in the proportion of patients utilizing health economic outcome measures from Baseline to Week 36.

Additional exploratory efficacy parameters may be specified in the statistical analysis plan.

6.1.4 Pharmacokinetic Endpoints

Samples for the measurement of whole blood concentrations of Au will be collected every 12 weeks before (pre-dose) administration of the investigational drug product. The data will be used to construct a composite whole blood concentration-time profile for the Q12 week visits over the randomised treatment period for the 30 mg QD dose.

PK collection procedures are described in Section 9.3.4.

Samples for the measurement of whole blood concentrations of Au will be collected before (pre-dose, and at 1, 2, 4, and 6 hours, after administration of the last dose of the investigational drug product at the Week 36 study visits. The study site shall contact the patient and document the time at which the patient administered his/her prior day's dose of study drug in order to impute a 24-hour trough value.

The data will be used to estimate an apparent Cmax and Tmax and area under the curve (AUC) over the 24-hour dosing interval [$AUC_{(0\text{-}24)}$].

6.1.5 Pharmacodynamic Endpoints

Blood, plasma, and urine will be collected at Baseline and at the Week 12, Week 24, and Week 36 visits per the schedule listed in Table 1. Samples for the measurement of pharmacodynamic (PD) assays will be collected at baseline (pre-dose) and at each study visit, and stored for subsequent pharmacodynamic analyses. PD collection procedures are detailed in Section 9.3.4. PD analyses will

be specified in a separate PD analysis plan and may include additional assays not directly specified in Section 6.1.5.

6.1.5.1 Whole Blood Metabolomic Markers

The PD analyses may include whole blood metabolomic markers for assessment of bioenergetic target engagement (e.g., redox coenzymes: NAD+, NADH, NADP+, NADPH; energetic coenzymes: ATP, ADP, AMP and antioxidants: GSSG and GSH). Disease Progression Pharmacodynamic Markers in urine may include urinary neurotrophin receptor $p75^{ECD}$ levels (Shepheard et al., 2017) and plasma neurofilament light chain (NfL) levels (Poesen and Van Damme, 2018).

6.2 Open-Label Extension Endpoints

6.2.1 Primary Safety Endpoint

The primary endpoint for the OLE is safety. Safety endpoints include incidence of treatment-emergent AEs, drug-related AEs, deaths, SAEs, and AEs leading to discontinuation from the study. Changes from baseline (Week 36 of the placebo controlled phase) in clinical laboratory results, physical examination findings, vital signs, ECGs, and C-SSRS will be summarized descriptively by group and timepoint.

6.2.2 Primary Efficacy Endpoint

The primary efficacy endpoint for the OLE is the mean change from Baseline (Week 36 of the Treatment Period) through End of Study for the MUNIXscore(4), which is the mean of the respective MUNIX values for the Abductor Digiti Minimi (ADM), Abductor Pollicis Brevis (APB), Biceps Brachii (BB), and Tibialis Anterior (TA). The average baseline mean values will be indexed to 100%. Changes will be calculated as the percent change from the Baseline index of 100%.

Electromyography measurements for the MUNIX assessment will be taken in the same hand or limb, as applicable, identified during the Treatment Period.

6.2.3 Secondary and Exploratory Efficacy Endpoints

All secondary and exploratory efficacy endpoints described above in sections 6.1.2 through 6.1.6 will be included in the optional OLE. The data collected at the Week 36 visit of the Treatment Period will service and the participants Baseline value for the OLE Period. Mean change will be compared between the Baseline and End of Study visits.

7 STUDY POPULATION

The study selection criteria were chosen to exclude patients who may potentially be exposed to specific risks after administering the study drug as well as patients with conditions that may have an impact on assessing the objectives of the study.

7.1 Study Inclusion Criteria

The patients to be enrolled in this study must meet the following inclusion criteria:

1. Able to understand and give written informed consent.
2. Male or female patients aged 30 years or greater (inclusive) and less than 80 years of age at the time of Screening.
3. Patients whose conditions are defined as *possible* or *probable* or *definite* ALS per the diagnostic criteria by Awaji-Shima criteria as determined by a neurologist sub-specialising in ALS (e.g., the Principal Investigator by study site).
4. For patients taking riluzole, stable dosing of riluzole over the prior 30-days from Screening.
5. At the time of Screening either disease duration less than or equal to 24-months from symptom onset, or disease duration less than or equal to 12-months from diagnosis.
6. Forced vital capacity (FVC) ≥ 60% of predicted value as adjusted for gender, height, and age at the Screening Visit.
7. Patient who has established care with a neurologist at one of the specialised ALS clinics involved in the study and will maintain this clinical care throughout the study. If a patient is referred from a third party (neurologist or a State based ALS organisation) they must be willing to transfer care to the neurologist participating in the study.

Following completion of the 36-week randomized placebo controlled phase of the trial, interested participants must meet the following inclusion criteria to enroll in the open-label extension:

1. Participants must have completed the randomized placebo controlled Treatment Period without compliance issues.
2. Able to understand and give written informed consent to participant in the open-label extension.
3. If referred from a third party (neurologist or a State based ALS organisation), participant agrees to maintain transfer of care to a neurologist participating in the study.

7.2 Study Exclusion Criteria

Patients will be excluded from the study if they meet any of the following criteria:

1. At Screening patients who utilize, or in the Investigator's judgment will be imminently dependent upon:

a. Non-invasive ventilation ≥22 hours per day, or
b. Tracheostomy

Note: If the patient requires non-invasive ventilation post-randomisation, they will be allowed to continue in the study.

2. Patients with Familial ALS (e.g., 2 or more family members with ALS or motor neuron disease)
3. Patients with a history of carpal tunnel syndrome, polyneuropathy, or in the investigators judgement diseases that could induce polyneuropathy and interfere with electromyography (EMG) recordings.
4. Patients with too severe atrophy of the Abductor Digiti Minimi (ADM), Abductor Pollicis Brevis (APB), Biceps Brachii (BB), or Tibialis Anterior (TA) muscles in the least clinically affected hand and leg, respectively, to allow for reliable EMG recordings.
5. Patient with a history of significant other major medical conditions based on the Investigator's judgment.
6. Based on the investigator's judgment, patients who may have difficulty complying with the protocol and/or any study procedures.
7. Patient with clinically significant abnormalities in hematology, blood chemistry, ECG, or physical examination not resolved by the Baseline visit which according to Investigator can interfere with study participation.
8. Patients with clinically significant hepatic or renal dysfunction or clinical laboratory findings that would limit the interpretability of change in liver or kidney function, or those with low platelet counts ($< 150 \times 10^9$ per liter) or eosinophilia (absolute eosinophil count of ≥ 500 eosinophils per microliter) at Screening.
9. Patient participating in any other investigational drug trial or using investigational drug (within 12 weeks prior to screening and thereafter).
10. Females who are pregnant or nursing or who plan to get pregnant during the course of this clinical trial or within 6 months of the end of this trial.
11. Females of child-bearing potential, or men, who are unwilling or unable to use accepted methods of birth control.
12. Active inflammatory condition or autoimmune disorder.
13. Positive screen for drugs of abuse.
14. History of gold allergy.
15. Patient is considered a suicide risk in the opinion of the Investigator, has previously made a suicide attempt, or is currently demonstrating active suicidal ideation. Subjects with intermittent passive suicidal ideation are not necessarily excluded based on the assessment of the Investigator.

Following completion of the 36-week randomized placebo controlled phase of the trial, interested participants will be excluded from participating in the OLE phase if they meet any of the following criteria:

1. Lack of treatment compliance during the randomized placebo controlled Treatment Period.
2. Positive pregnancy test at the Week 36 visit, or, females who plan to get pregnant during the course of this extension or within 6 months of the end of this extension.
3. Based on the investigator's judgment, patients who may have difficulty complying with the protocol and/or any study procedures.
4. Patient with clinically significant abnormalities in haematology, blood chemistry, ECG, or physical examination identified during the W36 visit which according to Investigator may interfere with continued participation.
5. Patients with clinically significant hepatic or renal dysfunction or clinical laboratory findings that would limit the interpretability of change in liver or kidney function, or those with low platelet counts ($< 150 \times 10^9$ per liter) or eosinophilia (absolute eosinophil count of ≥ 500 eosinophils per microliter) at the Week 36 visit.
6. Patient is considered a suicide risk in the opinion of the Investigator, has previously made a suicide attempt, or is currently demonstrating active suicidal ideation. Subjects with intermittent passive suicidal ideation are not necessarily excluded based on the assessment of the Investigator.

7.3 Reproductive Potential

Female patients of child bearing potential should have a negative serum β-hCG pregnancy test at the Screening Visit (Visit -1) and a negative urine pregnancy test at the Baseline Visit (Visit 0) prior to randomization. Male and female patients must abstain from sexual activity that could result in pregnancy or agree to use acceptable methods of contraception during the study (both Treatment and OLE Periods) and for 6-months following their last dose. Condoms should be used with the following acceptable contraceptives:

- Intrauterine devices
- Hormonal contraceptives (oral, depot, patch, injectable, or vaginal ring).

Other acceptable contraception methods are:

- Double barrier methods (e.g., condoms and diaphragms with spermicidal gel or foam).

All patients must be advised to use acceptable contraceptives throughout the study period(s) (both treatment and OLE) and for 180 days following the last dose of investigational product. If hormonal contraceptives are used, they should be administered according to the package insert. Male and female patients who are not currently sexually active must agree to use acceptable contraception, as defined

above, if they become sexually active during their participation and 180-days following the last dose of investigational product.

7.4 Removal of Patients from Therapy/Premature Discontinuation

A patient may withdraw from the study at any time for any reason without prejudice to their future medical care by the physician or the institution. The Investigator or Sponsor may withdraw the patient at any time (e.g., in the interest of patient safety). The withdrawal of a patient from investigational product by the Investigator should be discussed where possible with the Medical Monitor before the patient stops the investigational product. Any patient removed from the study will complete an end of treatment visit and remain under medical supervision until study discharge is medically acceptable.

If the investigational product is prematurely discontinued, regardless of the reason, the final study evaluations are to be performed as completely as possible. The information should be collected in the eCRF until the safety follow-up visit 28 ± 5 days after the last intake of study drug. Adverse events that occur within 28 ± 5 days after the last dose of study drug will be followed up until resolution, if possible.

All discontinued patients should also undergo the protocol-specified Safety Follow-Up Visit. Comments (spontaneous or elicited) or complaints made by the patient must be recorded in the appropriate source documents (e.g., an adverse event must be recorded and entered into the eCRF). The reason for termination, date of stopping investigational product, and the total amount of investigational product taken must be recorded on the electronic case report form (eCRF) and source documents for all discontinued patients.

Patients will be encouraged to complete the study and all assessments.

Patients *will be* discontinued from the study for the following medical and/or administrative reasons:

- Patient request or at the request of the patient’s legally acceptable representative
- Pregnancy, breast feeding, or repeat non-compliance with the scheduled pregnancy testing
- The Investigator judges that continuation of the study would be harmful to the patient's well-being
- TEAE or SAE that limits the patient’s ability to continue the study
- Treatment interruption for 35 days or more

Patients *may be* discontinued from the study for the following medical and/or administrative reasons:

- At the specific request of the Sponsor and in liaison with the Investigator (e.g., obvious non-compliance, safety concerns)
- If any exclusion criterion applies during the treatment periods

- Substantial non-compliance with planned study procedures
- Use of illicit drugs or other substances that may, in the opinion of the Investigator have a reasonable chance of contributing to toxicity or otherwise confound the study results

7.4.1 Patient Replacement

Patients who withdraw or are withdrawn following administration of the investigational product will be replaced under this protocol at the discretion of the Sponsor and Investigator.

7.4.2 Reasons for Discontinuation

The reason for withdrawal must be determined by the Investigator and recorded in the patient's medical record and on the eCRF. If a patient is withdrawn for more than one (1) reason, each reason should be documented in the source document and the most clinically relevant reason should be entered on the eCRF.

If a TEAE is the reason for discontinuation, then TEAE must be recorded on the eCRF.

Reasons for discontinuation include but are not limited to:

- Adverse event
- Protocol violation
- Withdrawal by patient
- Lost to follow-up
- Lack of efficacy
- Pregnancy
- Other (must be specified)

7.4.2.1 Lost to Follow-Up

At least 3 documented attempts must be made to contact any patient lost to follow-up at any time point prior to the last scheduled contact (office visit or telephone contact). One of these documented attempts must include a written communication sent to the patient's last known address via courier or mail (with an acknowledgement of receipt request) asking that the patient return any unused investigational product and return to the site for final safety evaluations, as applicable.

7.5 Patient Identification

After a patient has signed an informed consent form (ICF), the patient identification number for each patient will be assigned by site staff. Patients will be identified by a unique number (e.g., 036-001-001), which consists of:

- First three (3) digits: International Organization for Standardization (ISO) UN M49 Country code (e.g., Australia [036])
- Next three (3) digits: Site code within the country (e.g., [001] Sydney Brain Mind Centre)
- Last three (3) digits: Current patient number within the center

Patient identification numbers must be used in sequence and no number should be skipped, substituted, or re-used. Patients will retain their same identification number during the optional OLE Period.

8 INVESTIGATIONAL DRUG PRODUCT

8.1.1 Treatments Administered

During the Treatment Period, patients will either receive CNM-Au8 30 mg or color-matched placebo in the study. Study drug will be self-administered by patients who will be directed to take the study drug each day in the morning (7:00 – 10:00 am) at approximately the same time (e.g., 8:00 a.m. ± 1 hour), at least 30 minutes prior to planned food intake. Patients will be instructed to hold their daily dose on days where they will report to the clinic for a study visit. The daily dose should be administered at the site following collection of the pre-dose PK/PD samples.

The drug formulations will be identical in appearance (size, shape, volume, color) and smell. The packaging and labeling will be designed to maintain blinding to the Investigator's team and to patients. There are no visible differences between the 30 mg and placebo dosing units.

During the OLE Period, all patients who choose to participate will receive open-label CNM-Au8 30 mg for as long as the period is active. The dose for all patients may be adjusted once efficacy and safety data from the treatment period becomes available, which may occur after patients have already started this period.

8.1.2 Identity of Investigational Products

8.1.2.1 CNM-Au8

CNM-Au8 is a dark red/purple-colored liquid formulation consisting of a stable suspension of faceted clean surfaced elemental gold nanocrystals in buffered deionized water with a nominal concentration of 500 µg/mL of gold. The formulation is buffered by sodium bicarbonate ($NaHCO_3$), present at a concentration of 0.546 mg/mL. The suspension is initially created as an in-process bulk suspension with a gold nanocrystal concentration of 5.5 – 8.5 mg/L, which is then further processed to increase the gold concentration up to 1000 mg/L. The $NaHCO_3$ is present to assist in the manufacturing process and the concentration of $NaHCO_3$ remains nominally unchanged throughout processing. The pH of the suspension is between 8.0 and 10.0. There are no other excipients. The drug product is formulated to be taken orally and will be provided in single dose HDPE containers. The study doses vary by the concentration of gold nanocrystals per milliliter in a volume of 60 mL as described in Table 15.

The CNM-Au8 suspension is filtered using sterile, nonfiber-releasing 0.20 µm filters. The filtration occurs in a clean room with either ISO 7 or ISO 8 standards (depending upon the specific room utilized). The filtered solution is dispensed from the bulk container into clean, food-grade HDPE bottles.

The bottles will be labeled as described in Section 8.1.3.

Manufacturing, testing, product characterization, release of CNM-Au8 will be carried out at the Clene Nanomedicine, Inc. facility at 500 Principio Parkway West, Suite 400, North East, MD 21901, USA.

Table 15. CNM-Au8 Investigational Product Dosing Administration

**CNM-Au8**	
**Dosage**	30 mg
**Nominal Concentration**	500 µg/mL
**Volume**	60 mL
**Route of Administration**	Oral
**Time and Frequency**	Once Daily; Same Time Each Day (± 1 hour)

The investigational product components and quality standards are described in Table 16 below.

Table 16. CNM-Au8 Components and Quality Standards

**Description**	**Quality Standard**	**Concentration (mg/mL)**	**Amount Per Liter**
NaHCO3 (mg/mL)	USP	0.546	546 mg
Au (mg/mL)	Conforms with ASTM B562-95 and USP <232>	0.5	500 mg
USP water	USP for total organic carbon, and conductivity	N/A	1 L

8.1.2.2 Placebo

The color matched placebo to be used in this study will consist of water, $NaHCO_3$, and colorants. Manufacturing, testing, product characterization, and release of the investigational product will be carried out at the Clene Nanomedicine, Inc. facility at 500 Principio Parkway West, Suite 400, North East, Maryland 21901, USA

Table 17. Matched Placebo Dosing Administration

**Placebo**	
**Dosage**	NA
**Au Concentration**	NA
**Volume**	60 mL
**Route of Administration**	Oral
**Time and Frequency**	Once Daily; Same Time Each Day (± 1 hour)

The placebo components and quality standards are described below in Table 18**.**

Table 18. Matched Placebo Components and Quality Standards

Description	Quality Standard	Concentration (mg/mL)	Amount Per Liter
$NaHCO_3$	USP	0.546	546 mg
FD&C Red 40	Conforms with 21 CFR 74.340(a)(1) and (b)	0.28	280 mg
FD&C Blue 1	Conforms with 21 CFR 74.101(a)(1) and 74.101(b)	0.03	30 mg
USP water	USP for total organic carbon, and conductivity	N/A	1 L

The placebo is made with the same process water that is used to make CNM-Au8. The process water consists of deionized water supplemented with 0.546 mg/mL of $NaHCO_3$. The colors FD&C Red 40 and FD&C Blue 1 are added to match the color profile of CNM-Au8. The placebo solution will be filtered using sterile, nonfiber-releasing 0.20 µm filters. The filtration occurs in a clean room with either ISO 7 or ISO 8 standards (depending upon the specific room utilized). The filtered solution will be dispensed from the bulk container into clean, food-grade HDPE bottles. The bottles will be labeled as described below in Section 8.1.3.

8.1.3 Labeling

All investigational drug product will be labeled according to applicable local and legislative requirements. Label text will be approved according to the Sponsor's agreed procedures, and a copy of the labels will be made available to the study site upon request. For all study drugs, a system of numbering in accordance with all requirements of Good Manufacturing Practice (GMP) will be used, ensuring that each dose of study drug can be traced back to the respective bulk ware of the ingredients. The Sponsor's Quality Assurance group will maintain lists linking all numbering levels. A complete record of batch numbers and expiry dates of all study treatment as well as the labels will be maintained in the Sponsor study file.

Study drug label may include, but is not limited to, the following information:

- Batch number
- Storage information
- Site identification
- Expiry date
- Unique number/code
- Study period

8.1.4 Storage and Handling

Investigational product will be stored at the investigational site in accordance with Good Clinical Practice (GCP) and GMP requirements and will be inaccessible to unauthorized personnel. A complete record of batch numbers and expiry dates can be found in the Sponsor study file; the site-relevant elements of this information will be available in the Investigator site file. The unblinded responsible site personnel (e.g., site study pharmacist) will confirm receipt of study drug to Sponsor's designated production representative and will use the study drug only within the framework of this clinical study and in accordance with this protocol. Receipt, distribution, return and destruction (if any) of the study drug must be properly documented according to the Sponsor's agreed and specified procedures.

All study drug will be kept in a locked area with limited access and stored at 15°- 25°C (59°- 77°F). Mean kinetic temperature should not to exceed 25°C. Excursions between 15°C and 30°C (59° and 86° F) that may be experienced in pharmacies, hospitals, or warehouses, and during shipping are allowed.

8.2 Method of Assigning Patients to Treatment Groups and Dose Selection

8.2.1 Treatment Assignment/Randomisation

Patients who complete all screening procedures and meet the eligibility criteria to enter the study will be randomised using a site specific pre-specified block randomisation scheme at a 1:1 allocation (CNM-Au8 30 mg:Placebo). Randomisation will be performed by an unblinded site designee (e.g., site study pharmacist) and kit dispensation will be facilitated by unblinded site personnel in a blinded manner to others who must remain blinded throughout the study (e.g. investigators, clinical coordinators and patients). Refer to the Pharmacy operations manual for additional details regarding stratification and site-level randomization. All patient who choose to participate in the optional open-label extension period will received open-label CNM-Au8 30 mg regardless of their prior treatment assignment during the Treatment period.

8.2.2 First In Human Dosing Experience

The original planned CNM-Au8 dose levels in the first-time-in-human study were 15, 30, 60, and 90 mg CNM-Au8. The initial dose level selected was calculated based on the FDA guidance document "Estimating the Maximum Safe Starting Dose in Clinical Trials for Therapeutics in Adult Healthy Volunteers". The lowest NOAEL was in the rat and was 40 mg/kg. Based on this NOAEL, an equivalent human dose was determined to be approximately 6.4 mg/kg. In a 60-kg human patient, the selected starting dose of 15 mg CNM-Au8 represented a safety margin of approximately 26-fold.

The protocol for Study AU8.1000-14-01 was submitted to an Independent Ethics Committee and Competent Authority in the Netherlands and was subsequently approved for initiation under a Clinical Trial Application (CTA). The study was conducted in the Netherlands, at a Phase 1 facility, The Center for Human Drug Research (CHDR) and has been completed.

A total of 40 patients were randomised in the SAD phase and 46 patients in the MAD phase. Oral administration of either a single (SAD) or repeated dose (MAD) of CNM-Au8 over 21 consecutive days in healthy volunteers was safe and well tolerated. The TEAEs observed across the 4 doses (15 mg, 30 mg, 60 mg, and 90 mg) of CNM-Au8 were mostly mild in severity. While no dose response relationship to TEAEs was observed overall in the SAD or MAD Phase of the study, increases in headache and gastrointestinal related TEAEs were observed in the 90 mg cohort of the MAD Phase. No safety trends or safety signals were observed in the study.

In the SAD phase, PK analyses were not completed because the majority of whole blood AU concentrations were below the LOQ. However, a more sensitive ICP-MS instrument was employed for the MAD phase.

In the MAD Phase:

- The geometric mean whole blood concentrations from 1 week onward increased in a dose-related, but not a dose-proportional manner. Based on PK results from Days 14 and 21, the increases in both C_{max} and $AUC_{(0-24)}$ were less than dose proportional.
- Based on pre-specified fit criteria, the elimination $t_{½}$ ranged from 277 to 628 hr (11.5 to 26.2 days).
- Steady-state for all cohorts, based on the geometric mean whole blood concentrations, was reached by the end of the second week of dosing (Day 14), which was substantially less than predicted by the $t_{½}$ [range of 46 to 105 days (6.6 to 15 weeks)].
- PK parameters related to urinary excretion (Ue, CLr) could not be calculated. Only one urine Au concentration ≥ LOQ (3 ng/mL) was within limits of the assay.

8.2.3 Human Dosing Safety Margins vs. Nonclinical Toxicokinetic Data

Based upon the maximum doses (mg/kg/day) evaluated in the nonclinical GLP repeat-dose 21-day toxicokinetic studies, the completed first-time-in-human Phase 1 study (AU8.1000-14-01) doses of 15, 30, 60, 90 mg provided a safety margin to the NOAEL based on dose ratios (mg/m^2) ranging from 26x – 4x in rodents, and 195x – 32x in canines, as described in the tables below. Therefore, at the top dose of 90 mg tested in humans, there was a minimum 4x safety margin to the NOAEL in rats.

Table 19. Summary of CNM-Au8 Conversion of Animal 21-Day Repeat Doses To Human Equivalent Dose (HED) Based On Body Surface Area (mg/m^2) for 60 kg Human

Species	21-Day NOAEL Dose (mg/kg/day)	21-Day NOAEL Dose (mg/m^2)	Safety Margin Based on Dosing Ratios (mg/m^2) *(For 21-Day dosing Studies)*			
			**15 mg** (9.3 mg/m^2)	**30 mg** (18.5 mg/m^2)	**60 mg** (37.0 mg/m^2)	**90 mg** (55.5 mg/m^2)
Rat	40	**240**	25.9	13.0	6.5	4.3
Canine	90	**1800**	194.6	97.3	48.6	32.4

Further, when evaluating Au exposure based on the end of study Day 21 AUC$_{(0-24)}$ (ng*hr/mL) in the canine, and rodent studies in comparison with the human 21-day MAD study, the human doses provided an exposure safety margin ranging from 3.3x – 1.6x compared with rodents, and 18.5x – 9.0x compared with canines, as described in Table 20 below.

Table 20. Summary of CNM-Au8 Exposure Safety Margin Based on End of Study AUC$_{(0-24)}$ ng*hr/mL. Ratio of Animal Toxicokinetic to Human Pharmacokinetic AUC Results From 21-Day Repeat Dose Studies.

**Species**	**21-Day NOAEL Dose** (mg/kg/day)	**Animal End of Study AUC$_{(0-24)}$** (ng*hr/mL)[a]	**Safety Margin Based on Animal/Human AUC$_{(0-24)}$ Ratio** *(For 21-Day Dosing Studies)*			
			**Human 15 mg AUC** (32.3 ng*hr/mL)	**Human 30 mg AUC** (41.4 ng*hr/mL)	**Human 60 mg AUC** (50.3 ng*hr/mL)	**Human 90 mg AUC** (66.0 ng*hr/mL)
Rat	40	**106**	3.3	2.6	2.1	1.6
Canine	90	**596**	18.5	14.4	11.8	9.0

Notes: [a] Average of Male and Female AUC$_{(0-24)}$ ng*hr/mL values at End of Study

CNM-Au8 exposure at the NOAEL at the end of the chronic toxicokinetic studies in the 9-Month canine and 6-Month rodent chronic dosing studies, provided an exposure safety margin ranging from 6.5x – 3.2x, and 13.6x – 6.7x in rodents and canines, respectively in comparison with the human exposures in the 21-day MAD study, as described below in Table 21.

Table 21. Summary of CNM-Au8 Exposure Safety Margin Based on End of Study $AUC_{(0-24)}$ ng*hr/mL Ratio of Chronic Animal Toxicokinetic 6 and 9-Month Rodent and Canine Repeat Dose Studies to Human 21-Day Pharmacokinetic Results

Species (Study)	NOAEL Chronic Dosing (mg/kg/day)	Animal End of Study $AUC_{(0-24)}$ (ng*hr/mL)[a]	Safety Margin Based on Chronic Animal End-of-Study/ Human 21-Day $AUC_{(0-24)}$ Ratio			
			Human 15 mg AUC (32.3 ng*hr/mL)	Human 30 mg AUC (41.4 ng*hr/mL)	Human 60 mg AUC (50.3 ng*hr/mL)	Human 90 mg AUC (66.0 ng*hr/mL)
Rat (6-Month)	40	209	6.5	5.0	4.2	3.2
Canine (9-Month)	10	440	13.6	10.6	8.7	6.7

[a] Average of Male and Female $AUC_{(0-24)}$ ng*hr/mL values at End of Study

Based on CNM-Au8 exposures at the NOAELs in the chronic rodent and canine studies along with observed CNM-Au8 exposures in 21-day multiple dosing in humans while also considering safety and tolerability, doses chosen for this Phase 2 study in patients with ALS is 30 mg. This doses encompass the range of exposures from rodent toxicokinetic (TK) studies seen at the same dose used in the preclinical demyelination models at 10 mg/kg, where significant neuroprotection was observed per Figure 12.

Figure 12. 21-Day AUC Exposure Ranges Between Rodent Toxicokinetic Studies, Preclinical Efficacy Models, and First-In-Human Dosing

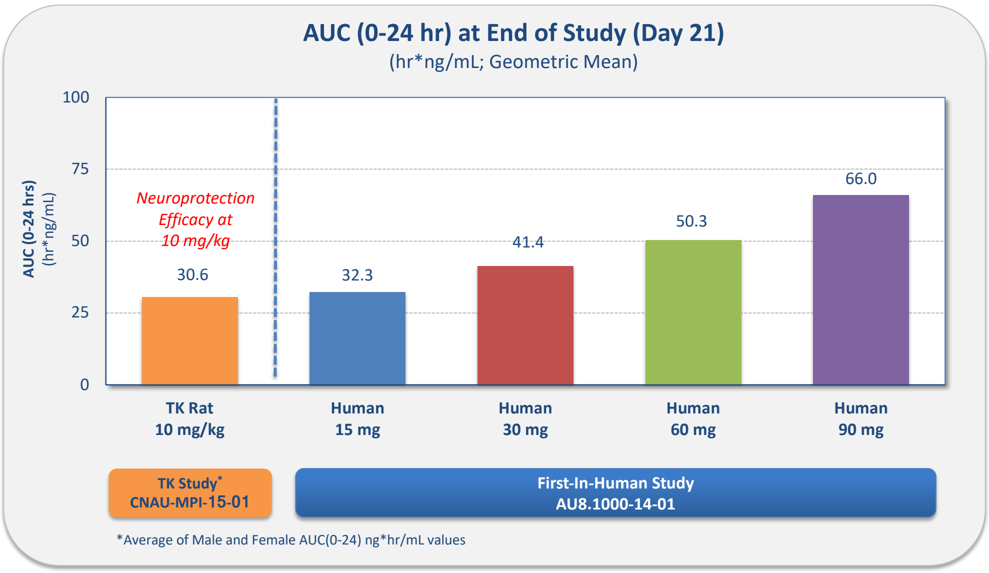

8.3 Procedures for Blinding

Patients will be randomised to receive CNM-Au8 or matching placebo in a double-blind fashion. The study data will remain blinded until database lock and authorisation of data release according to standard operating procedures (SOPs). More specifically, the blinding of study medication will be maintained until the last visit has been completed and all study databases have been locked.

The block randomisation code for each site will be generated by the study statistician and securely shared with the unblinded study site personnel (e.g., site study pharmacist). The randomisation code will be kept under secure conditions to not reveal the treatment assignments to the Sponsor, Investigators, clinical staff, or patients. The pharmacist at each site may maintain a secured copy of the randomisation code according to the site's SOP and dispense medication based on the randomisation scheme for each patient, and for emergency unblinding in the case it is required. An unblinded clinical research associate or study monitor will reconcile study drug assignment and allocation during specified monitoring visits.

8.3.1 Unblinding

The treatment allocation of a patient will be made available to the Investigator in case of emergency and when knowledge of the treatment allocation is important in the medical management of AEs. All unblinding events must be reported to the IRB/HREC in accordance with IRB/HREC timelines. Unblinding may occur in the circumstances as specified within the sub-sections below.

8.3.1.1 Unblinding by Sponsor's Pharmacovigilance Representative

In compliance with applicable regulations, in the event of a suspected unexpected serious adverse reaction (SUSAR), which is deemed related to blinded treatment, the patient's treatment code may be unblinded by the Sponsor before reporting to the health authorities, HRECs, IRBs, and investigators if the SUSAR is related to the blinded treatment.

8.3.1.2 Emergency Unblinding By The Investigator

In case of emergency and where knowledge of assigned treatment allocation is required for the acute management of a TEAE, the Investigator may unblind the case via the unblinded site pharmacist. Investigators should note that the occurrence of an AE or SAE should not routinely precipitate the immediate unblinding. If possible, the Investigator should always consult with the Medical Monitor prior to breaking the blind. The Investigator must contact the Medical Monitor in the event that the blind is broken and document the reason for unblinding.

8.3.1.3 Unblinding For Ongoing Safety Monitoring

In order to allow an ongoing safety monitoring during the conduct of the study by an external DSMB, members of the Committee will receive unblinded safety data on a quarterly or *ad hoc* basis. The involvement of an external Statistical Analysis Center in this process will ensure that unblinded information is not available for third parties. Details of the process are described in the DSMB charter.

8.4 Prior and Concomitant Therapy

Disease-specific medications (e.g., riluzole) are allowed per the inclusion criteria.

Except for disease specific medications (e.g., riluzole), acetaminophen (paracetamol), ibuprofen, naproxen, and 2nd generation antihistamines (including fexofenadine, loratadine, and cetirizine); patients may not take any new prescription medications, OTC, or dietary supplements from 14-days prior to Baseline through the safety follow-up (including the OLE) unless used to manage a treatment emergent adverse event, in which case the patient should report to his/her Study Physician as soon as possible, the occurrence of the adverse event and the medication(s) they are taking to treat the adverse event.

The Investigator will make every effort to contact the Medical Monitor or Sponsor representative prior to administration of a new concomitant therapy (prescription or OTC) after randomisation, unless the concomitant therapy is needed immediately for patient safety.

Any use of new prescription medicines, OTC medications, or dietary supplements during the study period must be reported on the eCRF.

8.5 Treatment Compliance

Patients will receive study drug dispensed per visit schedule in Table 1 during the Treatment Period and Table 2 during the Open-Label Extension Period.

To facilitate treatment compliance patients may be reminded daily to take their investigational medicine with automated email and/or mobile text message reminders. The administration of the automated reminder program will be per the Investigator, authorized clinical site personnel, or the CRO.

Patients will be requested to return any unused study drug including empty packaging and used bottles at each study visit. Treatment compliance will be assessed at study visits through bottle counts and will be documented and summarized by a drug-dispensing log for each patient. A web-based or mobile application compliance tool may also be utilized to collect supportive information regarding daily intake of the investigational medicine. Overall treatment compliance with study drug intake for the *per protocol* analysis set should be between 80% and 120% of the planned dose and will be assessed at each study visit. In the event patients are not compliant within this range, discontinuation may be considered by the Investigator in consultation with the Sponsor.

The date of dispensing the study drug to the patient will be documented in the eCRF.

If a dose of study drug is missed, the patient should take the dose that day and continue with the planned dosing interval for the following day. The dose should not be doubled to make up for a missed dose within the same day.

8.5.1 Study Drug Accountability

Study drug will be administered in accordance with the procedures of this protocol. Only authorized site personnel may supply study drug, and only patients enrolled in the study may receive study drug, in accordance with applicable regulatory requirements.

Blinded CRO representatives will perform ongoing drug accountability assessments during planned interim study monitoring visits. A different designated unblinded CRO representative who is not involved in routine study monitoring will reconcile study drug assignment and allocation by the site study pharmacist on a periodic basis. Following the specific *unblinded* monitoring visits by the designated unblinded CRO representative, unused study drug and empty containers may be destroyed per Section 12.1.8. Final reconciliation and full drug accountability will be completed by site personnel, pharmacy staff, and will be assessed by Sponsor or designee after the study blind is broken.

8.6 Data Safety Monitoring Board

An independent Data Safety Monitoring Board (DSMB) will be responsible for monitoring the safety of the study on a trimester basis and *ad hoc* at the option of the DSMB or the Sponsor (e.g. in the event of unexpected SAEs) to review data throughout study. The DSMB may make recommendations on the conduct of the study, including study termination. Appropriate procedures will be detailed in a DSMB Charter that defines disclosure of any findings along with patient and study-stopping criteria.

9 STUDY PROCEDURES

9.1 Study Schedule (Treatment Period)

A Time and Events Schedule is provided in Table 1 for the blinded, randomized controlled Treatment Period.

Screening may occur on Days -42 through -1. Of note, there is not a minimum required screening duration, so study subjects may be randomised into the study at any time within the 42-day screening window if they meet the study enrolment criteria.

The Baseline visit will occur on Day 1. The treatment period will include Day 1 through the last dose of study medication.

9.1.1 Screening Phase (Day -42 through Day -1) – VISIT (-1)

The initial Screening Visit may be conducted up to 42 days prior to Baseline on Day 1. The following procedures/assessments will be performed at Screening:

- Informed consent (must be done prior to any of the following procedures)
- Medical history and patient demographics (confirm ALS diagnosis)
- Prior and current medication assessment
- Physical examination
- Brief neurological exam
- Confirm patient meets all eligibility criteria
- Height and weight assessment
- Vital Signs
- Urinalysis
- Urine drug screen (cocaine, marijuana, opiates, benzodiazepines, amphetamines)
- Venous blood for clinical laboratory tests
- Venous blood for infectious disease screen (HIV, hepatitis B, and hepatitis C)
- Serum pregnancy test (for females of childbearing potential)
- 12-Lead ECG
- ALSFRS-R

- FVC
- Adverse Events
- C-SSRS (Baseline Version)
- Schedule next study visit (Baseline Visit)

9.1.2 Baseline (Day 1) – VISIT 0

Prior to performing any Baseline assessment, all screening assessments and reports must be reviewed and signed by an Investigator. Patients will present to the study site for the Baseline assessments prior to dosing initiation, all Baseline assessments must be performed prior to administration of the first dose of study drug. The following assessments are to be completed at or by the Baseline visit:

- Re-confirm patient meets all eligibility criteria
- Physical exam
- Brief neurological exam
- Concomitant medication assessment
- Vital signs
- Weight
- 12-Lead ECG in triplicate
- Urine pregnancy test (for females of childbearing potential)
- Randomisation
- Venous blood for clinical laboratory tests
- PD whole blood, plasma, and urine collection
- Urinalysis
- Electromyography (Standard EMG: MUNIX, MUSIX, F-Wave, CMAP, DML)
- Electromyography (MScan of APB)
- ALSFRS-R
- ALSSQOL-SF
- FVC

- Health Utilisation
- Patient's Global Impression Scale (Severity)
- Clinician's Global Impression Scale (Severity)
- C-SSRS (Since Last Visit Version)
- Adverse events
- Dispense Study Drug Package
- Take First Study Dose at the Site from the dispensed supply (following all Baseline assessments)
- Schedule next study visit

9.1.3 Treatment Phase (VISIT 1 through VISIT 7)

Following the Baseline Visit, patients will either be contacted via telephone or will return to the study site for evaluations of safety and efficacy as outlined in Table 1.

9.1.3.1 Treatment Period – VISIT 1, 2, 4, and 6 (WEEK 3, 6, 18, and 30)

At Weeks 3, 6, 18, and 30, clinical site staff will contact patients by telephone and assess for the following items:

- Adverse Events
- Concomitant medication assessment
- Treatment compliance assessment
- Confirm scheduling of next visit

9.1.3.2 Treatment Period – VISITS 3, 5, and 7

All patients will participate in the study for a minimum of 36-weeks per protocol.

Study activities for Visits 2, 3, and 4 at Weeks 12, 24, and 36 include:

- Brief physical exam
- Brief neurological exam
- Concomitant medication assessment
- Vital signs
- Weight

- 12-Lead ECG
- Urine pregnancy test (for females of childbearing potential)
- Venous blood for clinical laboratory tests
- PD blood, and urine collection
- PK whole blood sample(s)
- Urinalysis
- Electromyography (Standard EMG: MUNIX, MUSIX, F-Wave, CMAP, DML)
- Electromyography (MScan of APB)
- ALSFRS-R
- ALSSQOL-SF
- FVC
- Health Utilisation
- Falls Questionnaire
- Patient’s Global Impression Scale (Severity and Improvement) Clinician’s Global Impression Scale (Severity and Improvement)C-SSRS (Since Last Visit Version)
- Adverse events
- Dispense Study Drug Package
- Take Study Dose at the Site from the dispensed supply (following all Baseline assessments)
- Schedule next study visit

9.1.3.3 Safety Follow-up Visit – Week 40

Timing for the safety follow-up visit should occur at 28-days (±5 days) following study discontinuation for: 1) patients who discontinue therapy prior to Week 36, or 2) who do not transition to the optional OLE period.

Study activities during the follow-up visit include:

- Brief physical exam
- Brief neurological exam

- Vital signs
- Venous blood and urine for clinical laboratory tests
- Concomitant medication assessment
- Adverse events
- C-SSRS (Since Last Visit Version)
- Return Study Drug Package (if discontinued prior to the Week 36 visit)

Any AEs/SAEs occurring up to the time of the Follow-up visit will be recorded. Appropriate follow-up should continue until all safety concerns, in the Investigator's opinion, are resolved, or return to baseline.

9.2 Study Schedule (Open-Label Extension Period)

A Time and Events Schedule is provided in Table 1 for the optional Open-Label Extension (OLE) Period.

The assessments performed during the Week 36 visit of the Treatment Period will serve as the Baseline values for the optional OLE Period. The OLE Period is planned to run for up to 48 weeks beyond the 36-week treatment period, which may be extended for additional 12-week increments until until discontinued by the Sponsor. For patients who enter the OLE Period 12-weeks or more after completing their Treatment Period, the assessments indicated in Table 2 will be repeated to serve as their respective Baseline values.

9.2.1 OLE Baseline (Day 1) – Visit 0

Prior to dispensing open-label CNM-Au8, patients must provide consent to participate in the OLE period and eligibility should be confirmed. The following assessments are to be completed at or by the Baseline visit:

- Obtain informed consent for OLE period
- Confirm patient meets all eligibility criteria
- Brief physical exam (collected during W36 visit)
- Brief neurological exam (collected during W36 visit)
- Concomitant medication assessment (collected during W36 visit)
- Vital signs (collected during W36 visit)

- Weight (collected during W36 visit)
- 12-Lead ECG (collected during W36 visit)
- Urine pregnancy test (for females of childbearing potential) (collected during W36 visit)
- Venous blood for clinical laboratory tests (collected during W36 visit)
- PD blood, and urine collection (collected during W36 visit)
- PK whole blood sample(s) (collected during W36 visit)
- Urinalysis (collected during W36 visit)
- Electromyography (Standard EMG: MUNIX, MUSIX, F-Wave, CMAP, DML) (collected during W36 visit)
- Electromyography (MScan of APB) (collected during W36 visit)
- ALSFRS-R (collected during W36 visit)
- ALSSQOL-SF (collected during W36 visit)
- FVC (collected during W36 visit)
- Health Utilisation (collected during W36 visit)
- Falls Questionnaire (collected during W36 visit)
- Patient's Global Impression Scale (Severity and Improvement) Clinician's Global Impression Scale (Severity and Improvement)C-SSRS (Since Last Visit Version) (collected during W36 visit)
- Adverse events (collected during W36 visit)
- Dispense Study Drug Packages
- Take Study Dose at the Site from the dispensed supply (following collection of informed consent)
- Schedule next study visit

9.2.2 OLE Period – Visit 1 (Week 6)

At Week 6 of the OLE Period, clinical site staff will contact patients by telephone and assess for the following items:

- Adverse Events
- Concomitant medication assessment

- Treatment compliance assessment
- Confirm scheduling of next visit

9.2.2.1 OLE Period – Visits 2, 4, 6, and n+2 (Weeks 12, 36, 60, and n+24)

Study activities for Weeks 12, 36, 60 and every 24 weeks thereafter include:

- Brief physical exam
- Brief neurological exam
- Concomitant medication assessment
- Vital signs
- Urine pregnancy test (for females of childbearing potential)
- Venous blood for clinical laboratory tests
- Urinalysis
- ALSFRS-R
- ALSSQOL-SF
- FVC
- Health Utilisation
- Falls Questionnaire
- Patient's Global Impression Scale (Severity and Improvement) Clinician's Global Impression Scale (Severity and Improvement) C-SSRS (Since Last Visit Version)
- Adverse events
- Drug Accountability
- Dispense Study Drug Packages
- Take Study Dose at the Site from the dispensed supply (following all assessments)
- Schedule next study visit

9.2.2.2 OLE Period – Visits 3, 5, 7 and n+2 (Weeks 24, 48, 72 and n+24)

Study activities for Weeks 24, 48, 72 and every 24 weeks thereafter include:

- Brief physical exam

- Brief neurological exam
- Concomitant medication assessment
- Vital signs
- Weight
- 12-Lead ECG
- Urine pregnancy test (for females of childbearing potential)
- Venous blood for clinical laboratory tests
- PD blood, and urine collection
- PK whole blood sample(s)
- Urinalysis
- Electromyography (Standard EMG: MUNIX, MUSIX, F-Wave, CMAP, DML)
- Electromyography (MScan of APB)
- ALSFRS-R
- ALSSQOL-SF
- FVC
- Health Utilisation
- Falls Questionnaire
- Patient's Global Impression Scale (Severity and Improvement) Clinician's Global Impression Scale (Severity and Improvement) C-SSRS (Since Last Visit Version)
- Adverse events
- Drug Accountability
- Dispense Study Drug Packages
- Take Study Dose at the Site from the dispensed supply (following all assessments, excluding last visit)
- Schedule next study visit

9.2.2.3 Safety Follow-Up Visit – 4 weeks following last visit

Timing for the safety follow-up assessment should occur at 28-days (±5 days) following last dose of study drug administer for those who participate in the optional OLE period.

Study activities during the follow-up visit include:

- Brief physical exam
- Brief neurological exam
- Vitals
- Venous blood and urine for clinical laboratory tests
- Concomitant medication assessment
- Adverse events
- C-SSRS (Since Last Visit Version)
- Return Study Drug Package (if discontinuing prior to completion)

9.3 Study Measurements and Assessments

Measurements and assessments are to be performed according to the schedule shown in Table 1 for the Treatment Period and Table 2 for the OLE Period.

Measurements and assessments should only be performed by trained and qualified personnel and whenever possible, the same person at each site should perform each of these assessments for all Patient visits.

If a patient terminates treatment prematurely, all assessments listed in Table 1 or Table 2 for the safety follow-up should be completed 28 days (±5 days) from date of last dose.

9.3.1 Demographic and Other Baseline Characteristics

Demographic characteristics such as age, sex, weight, height, ethnicity, and body mass index (BMI) will be collected during the study according to the schedule in Table 1.

9.3.2 Assessment of Efficacy

Efficacy will be assessed at each visit noted in the Study Schedules (Table 1 and Table 2) using the measures described below. All efficacy measurements are to be performed by a trained and qualified person.

To standardize acquisition data across study sites, healthy volunteers may participate during site training, data acquisition, and test transfer to the central reading centres for the electromyography assessments described below.

9.3.2.1 Electromyography Data Collection

Electromyography data collection will occur in the least-clinically affected hand/limb identified at the Baseline visit in the Abductor Digiti Minimi (ADM), Abductor Pollicis Brevis (APB), Biceps Brachii (BB), Tibialis Anterior (TA), and the First Dorsal Interosseous per Table 22.

Table 22. EMG Data Collection Plan

**Least Clinically Affected Hand/Limb**			**EMG Sequence**				
			**Standard EMG**				**MScan**
**Muscle**		**Abbr**	MUNIX + MUSIX	CMAP Peak Amplitude	Distal Motor Latency	F-Wave/ Latency	CMAP Stimulus Response
**Hand**	Abductor Pollicis Brevis	APB	X	X	X	X	X
	First Dorsal Interosseous	FDI	X	X	X		
	Abductor Digiti Minimi	ADM	X	X	X	X	
**Arm**	Biceps Brachii	BB	X	X	X		
**Leg**	Tibialis Anterior	TA	X	X	X	X	
**Est. Time per Visit**			~25-30 min				~6-8 min

9.3.2.2 MUNIX

The Motor Unit Number Index (MUNIX) is quantitative neurophysiological method that reflects loss of motor neurons in ALS in longitudinal studies (Neuwirth et al., 2018). MUNIX assessment employs an electromyography stimulation of compound muscle action potential (CMAP) and the surface electromyography interference pattern (SIP) and then uses a mathematical model to derives an index related to the number of motor units (Nandedkar et al., 2010). MUNIX requires minimal electrical stimulation, is highly reproducible in healthy volunteers and ALS patients, and is suitable for serial EMG investigations (Nandedkar et al., 2004; Neuwirth et al., 2010).

EMG data collection for assessment of MUNIX will be conducted at the Baseline Visit and at the Week 12, Week 24, and Week 36 visits during the Treatment Period and every 24 weeks during the OLE Period. The MUNIXscore(4), the mean of the MUNIX values for the APB, ADM, BB, and TA is the study primary efficacy endpoint.

9.3.2.3 MScan and MScan Fit

In MScan, after recording a maximal CMAP by stimulating the median nerve at the wrist, an electrical stimulus is applied and gradually reduced from supramaximal level to subthreshold in 0.2% steps to generate a detailed and inverted stimulus–response curve or CMAP scan (Bostock, 2016; Jacobsen et al., 2018). The stimulus–response curve describes the amplitude of the motor response as a function of stimulus current, due to recruitment of more and more motor units with increasing stimulus intensity. The stimulus–response curve describes the amplitude of the motor response as a function of stimulus current, due to recruitment of more and more motor units with increasing stimulus intensity. In MScanFit, a model is then fitted to the recorded stimulus response curve scan data to obtain an estimate of the number of motor units (motor nerves) in the specific nerve. The stimulus-response curves will be analysed in customized software [Qtrac software program, Institute of Neurology, University College, London), and from this model the number of motor units, mean amplitude of motor units and the amplitude of the largest motor unit (a biomarker of re-innervation) will be derived according to established methodology (Bostock, 2016; Jacobsen et al., 2017, 2019)

MScan will be conducted at the Baseline Visit and at the Week 12, Week 24, and Week 36 visits during the Treatment Period and every 24 weeks during the OLE Period. The MScanFit is a secondary efficacy endpoint.

9.3.2.4 MUSIX

The Motor Unit Size Index (MUSIX) is obtained by dividing MUNIX into CMAP amplitude: $CMAP^{amplitude}$/MUNIX. MUSIX is measured in microvolts (μV) and reflects the average amplitude of the surface-recorded motor unit potential (SMUP) (Nandedkar et al., 2010). Accordingly, MUSIX is an electromyographic index for size of motor units and not their absolute values. Since the rate of symptom progression in neuromuscular diseases such as ALS is determined by both the amount of axon loss and effective reinnervation, the MUSIX measurement therefore represents the compensatory reinnervation process in neuromuscular disorders, which may reveal important information on the natural course of such conditions and the response to new treatments (Alix et al., 2019).

EMG data collection for assessment of MUSIX will be conducted at the Baseline Visit and at the Week 12, Week 24, and Week 36 visits during the Treatment Period and every 24 weeks during the OLE Period. The MUSIXscore(4), the mean of the MUSIX values for the APB, ADM, BB, and TA is a secondary efficacy endpoint.

9.3.2.5 Neurophysiological Index (NPI)

The neurophysiological index (NPI) was developed to quantify peripheral disease burden in ALS

patients (Cheah et al., 2011). NPI has been shown to be more responsive to disease progression over a short measurement period than the ALSFR-S, together with moderate correlation with functional deterioration. The NPI is defined as the ulnar nerve ($ADM^{CMAP\ Peak\ Amplitude}$ / $ADM^{Distal\ Motor\ Latency}$) * $ADM^{F\text{-}Wave\ (\%)}$.

EMG data collection for assessment of NPI will be conducted at the Baseline Visit, and at the Week 12, Week 24, and Week 36 visits during the Treatment Period and every 24 weeks during the OLE Period. The NPI a secondary efficacy endpoint.

9.3.2.6 Split Hand Index (SI)

Dissociated atrophy of intrinsic hand muscles, including the abductor pollicis brevis (APB) and first dorsal interosseous (FDI), with relative preservation of the hypothenar muscles, the split hand sign appears to be an early clinical feature of ALS (Menon et al., 2013). The clinical observation of dissociated hand muscle atrophy a simple neurophysiological biomarker for evaluating ALS. The split-hand index (SI) was derived by multiplying the compound muscle action potential (CMAP) amplitude recorded over the APB muscle by the CMAP amplitude recorded over the FDI muscle and dividing this product by the CMAP amplitude recorded over the abductor digiti minimi (ADM).

EMG data collection for assessment of the SI will be conducted at the Baseline Visit, and at the Week 12, Week 24, and Week 36 visits during the Treatment Period and every 24 weeks during the OLE Period. The SI a secondary efficacy endpoint.

9.3.2.7 ALSFRS-R

The ALS Functional Rating Scale-Revised (ALSFRS) is a validated rating instrument for monitoring the progression of disability in patients with amyotrophic lateral sclerosis (ALS). The revised version incorporates additional assessments of dyspnea, orthopnea, and the need for ventilatory support. The Revised ALSFRS (ALSFRS-R) retains the properties of the original scale and shows strong internal consistency and construct validity.

The ALSFRS-R will be conducted at the Screening Visit, the Baseline Visit, and at the Week 12, Week 24, and Week 36 visits during the Treatment Period and every 12 weeks during the OLE Period. Assessment of ALSFRS-R (e.g., mean change from baseline, change in slope, proportion of patients experiencing a ≥ 6 point decline) are exploratory endpoints.

9.3.2.8 FVC

The extent of respiratory involvement has been reported as a major prognostic factor in ALS patients. Forced vital capacity (FVC) is one index of respiratory function that may be used to indicate potential respiratory compromise in ALS.

FVC will be conducted at the Screening Visit, Baseline Visit, and at the Week 12, Week 24, and Week 36 visits during the Treatment Period and every 12 weeks during the OLE Period. FVC mean change from Baseline is an exploratory endpoint.

9.3.2.9 CGI

The CGI scales (assessing both severity [CGI-S] and improvement [CGI-I]) provides a brief assessment of the clinician's view of the patient's global functioning and severity of current disease state and can be utilized to assess for changes in disease progression over the course of a clinical trial.

The CGI-S will be conducted at the Baseline Visit, and both the CGI-S and CGI-I at the Week 12, Week 24, and Week 36 visits during the Treatment Period and every 12 weeks during the OLE Period. CGI mean change from Baseline is an exploratory endpoint.

9.3.2.10 PGI

The PGI scales (assessing both severity [PGI-S] and improvement [PGI-I]) provides a brief assessment of the patient's view global functioning and severity of current disease state and can be utilized to assess for changes in disease progression over the course of a clinical trial.

The PGI-S will be conducted at the Baseline Visit, and both the PGI-S and PGI-I at the Week 12, Week 24, and Week 36 visits during the Treatment Period and every 12 weeks during the OLE Period. PGI mean change from Baseline is an exploratory endpoint.

9.3.2.11 Healthcare Utilisation

A healthcare utilization questionnaire will document subject utilization of healthcare resources including manual wheelchair, power wheelchair, gastrostomy tube, non-invasive ventilation, and augmentative and alternative communication.

The healthcare utilization form will be implemented at the Baseline Visit, and at the Week 12, Week 24, and Week 36 visits during the Treatment Period and every 12 weeks during the OLE Period. The mean change from Baseline in the proportion of subjects utilising healthcare resources is an exploratory endpoint.

9.3.3 Assessment of Safety

Safety assessments will include the following:

- Treatment emergent AEs and SAEs, both reported and observed
- Physical examination

- Vital sign measurements: Ear temperature, respiration, blood pressure, and heart rate (blood pressure and heart rate will be measured after the patient has been supine for 5 minutes)
- 12-Lead ECG will be conducted in triplicate at the Baseline Visit and averaged. A single ECG collection will occur at each additional scheduled time point
- Venous blood for clinical laboratory tests will include hematology, and blood chemistry. Blood for analysis will be drawn after an overnight fast when possible
- Falls Assessment will be conducted at each study visit following randomisation.
- C-SSRS Columbia Suicide Severity Rating Scale (C-SSRS)

The C-SSRS is a semi-structured interview that captures the occurrence, severity, and frequency of suicide-related thoughts and behaviors during the assessment period (Posner et al., 2011). The interview includes definitions and suggested questions to solicit the type of information needed to determine if a suicide-related thought or behavior occurred.

The C-SSRS contains 2 required items pertaining to suicidal ideation, 4 required items pertaining to suicidal behavior, and 1 required item pertaining to non-suicidal self-injurious behavior. There are 8 additional suicidal ideation items and 2 additional suicidal behavior items which are completed in cases of positive responses for other items, as well as 2 items for completed suicide and suicide behavior present during the interview. Thus, there is a maximum of 19 items to be completed.

The C-SSRS must be performed by an individual who is medically responsible for the subject.

Two versions of the C-SSRS are used in this study:

- The "Baseline" version will be administered at the Screening Visit (Visit -1) and will be completed for all subjects.
- The "Since Last Visit" version will be completed for all subjects at all in-clinic study visits after the Screening Visit (Visit -1).

Outlier criteria for safety results including chemistry, urine, hematology, ECG, vital signs are based upon the National Cancer Institute (NCI) Common Terminology Criteria for Adverse Events (CTCAE) v5.0, November 27, 2017. This outlier criteria document has been authored and approved by the Sponsor safety physician and the CRO physician to enhance review of safety data. The outlier criteria will be used as an additional review tool for TEAE reporting.

9.3.4 PK and PD Assessments

Blood samples for pharmacokinetic (PK) and pharmacodynamics (PD) analyses will be collected during the Treatment Period as outlined in the Time and Events Schedule in Table 1. Blood samples

for pharmacokinetic (PK) and pharmacodynamics (PD) analyses will be collected during the OLE Period as outlined in the Time and Events Schedule in Table 2.

During PK and PD study visits patients should present themselves to the clinical site or hospital without taking the morning dose of study medication. During these visits the PK and PD samples should always be taken at pre-dose trough before administration of the morning dose of the study medication. Accurate time of blood sampling and administration times for PK and PD collection will be documented in the eCRF.

An indwelling venous catheter may be inserted for the collection of blood samples if deemed necessary. Appropriate flushing techniques should be applied to remove dead space blood prior to collecting a sample for PK and PD analyses.

Pharmacokinetic analyses, and pharmacokinetic/pharmacodynamics (PK/PD) modeling as applicable, using population approaches to describe Au whole blood pharmacokinetics including potential influence of relevant patient co-variables (e.g. age, gender, body weight, etc.) or potentially to relate parameters of clinical safety and efficacy response with Au whole blood concentrations will be investigated under separate detailed PK and/or PD evaluation plan(s).

9.3.4.1 Collecting, Processing, and Shipping of PK and PD Samples

For PK analyses, 4 mL of whole blood will be collected for each blood draw for bioanalytical analyses of Au content. Blood samples for PK analyses will be collected in tubes with dipotassium ethylenediaminetetraacetic acid (K_2EDTA), which should not exceed 1.6 mg K_2EDTA per mL of whole blood). PK samples must be placed on wet crushed ice immediately and stored at -70 °C or lower within 15 minutes and processed per the instructions described in the study's Lab Manual.

For PD analyses, additional whole blood and plasma will be collected following the collection of the PK. Further, urine for PD analyses will be collected at the specified visits noted in Table 1 and Table 2. PD sample collection and processing guidelines will be specified in the study's Laboratory Manual.

9.3.4.2 Bioanalytical Methods

Blood concentrations of gold (Au) will be determined by using validated Inductively Coupled Plasma Mass Spectrometer (ICP-MS) analytical methods. The lower limit of quantification, deviation of calibration standards from the theoretical value, and precision have been established using standard methods. Performance of the assay will be assessed by monitoring the analysis of spiked samples with known concentrations of CNM-Au8.

Blood samples will be analyzed under the responsibility of the Sponsor's bioanalytical laboratory at:

Clene Nanomedicine, Inc.
Bioanalytics Laboratory
500 Principio Parkway West, Suite 400
North East, MD 21901-2912
USA

9.3.5 Clinical Laboratory Tests

All routine safety samples will be analyzed by the local sites' clinical laboratory unless otherwise specified in the study laboratory manual. At the end of the study when all planned analyses are completed, all clinical laboratory blood samples will be destroyed per the central clinical laboratory SOPs.

The clinical laboratory tests will include, but are not limited to:

- Hematology: hemoglobin, hematocrit, white blood cell count with differential, red blood cell count, and platelet count.
- Serology (Screening Visit only): human immunodeficiency virus (HIV), hepatitis C (HepC), and hepatitis B (HepB).
- Blood Chemistry: Alanine aminotransferase (ALT; SGPT) and Aspartate aminotransferase (AST; SGOT), total bilirubin, direct bilirubin, Gamma-Glutamyl transferase (GGT), blood urea nitrogen (BUN), creatinine, alkaline phosphatase, lactate dehydrogenase (LDH), sodium, potassium, calcium, chloride, albumin, uric acid, lipid profile/panel, creatine kinase (CK).
- Serum and urine pregnancy test: Beta-human chorionic gonadotropin (βhCG) test for females of childbearing potential.
- Urinalysis: Dipstick analyses will include: Leucocytes, blood, nitrite, protein, urobilinogen, bilirubin, pH, specific gravity, ketones, and glucose. Urinalysis will include macroscopic analysis and microscopic analysis only when indicated by dipstick.
- Urine Drug Screen: Cocaine, cannabinoids, amphetamines, benzodiazepines and opiates. Patients with positive urine drug screen test results will be excluded from the study. Urine Drug Screen kits will be supplied for sites to complete testing onsite. Repeat urine drug screens for validation of initial findings may be conducted at the discretion of the site investigator. If a patient holds an active prescription for a medication that could return a positive result on the above urine drug analytes, the patient may be allowed to continue so long as elicit use is not suspected.

The clinical sites local laboratory or core clinical laboratory will supply appropriate blood collection tubes for each study visit. The Lab Manual will specify the blood collection volumes and specific collection tubes to be utilised.

9.3.6 Physical Examinations

A full physical examination will consist of assessments of the following: skin, ears, nose, throat, head, eyes, lungs/chest, heart, abdomen, musculoskeletal, extremities, neurologic. Following randomisation, a symptom directed physical exam and brief neurological exam will be performed at each visit.

9.3.7 Vital Signs

Vital signs will include respiration rate (breaths per minute), temperature, blood pressure (mmHg) and heart rate (beats per minute [bpm]). Blood pressure and heart rate will be obtained after the patient has been resting in supine position for 5 minutes. If vitals are not able to be captured in a supine position, vitals may be collected in a sitting position. The same arm must always be used for blood pressure and heart rate measurements. Noninvasive measurement should be conducted preferably with a mercury sphygmomanometer or a validated electronic device in accordance with published guidelines (e.g. American Heart Association: Recommendations for Blood Pressure Measurement in Humans and Experimental Animals) (Pickering et al., 2005).

9.3.8 12-Lead ECG

12-lead ECGs will be performed successively in triplicate after the patient has rested quietly for at least 5 minutes in a supine position at the Baseline Visit. If the ECG is not able to be captured in a supine position, vitals may be collected in a sitting position. The average corrected QT interval (QTc) of the triplicate ECG measurements collected prior to Day 1 dosing will serve as that patient's baseline for use in ongoing safety assessment. At all other scheduled timepoints, a single ECG will be collected. When the timing of the measurement coincides with a blood collection, the ECG should be obtained prior to blood collection. In some cases, it may be appropriate to repeat abnormal ECGs to rule out improper lead placement as contributing to the ECG abnormality. It is important that leads are placed in the same positions each time for consistency.

ECG intervals will be summarized and presented descriptively. ECG rhythm will be interpreted by the Investigator as normal (N), abnormal not-clinically significant (aNCS), or abnormal clinically significant (aCS).

9.4 Assessment Windows

Collection of PK samples should occur as close to the nominal timepoint as possible. The actual time of PK sample collection will be recorded. All other assessments should be scheduled around the collection of PK samples at their nominal timepoints.

10 ADVERSE EVENTS

Safety will be assessed up through the frequency of treatment emergent adverse events, serious adverse events, discontinuations due to adverse events, Falls Questionnaire, and the Columbia Suicide Severity Rating Scale (C-SSRS).

10.1 Safety Reporting Guidelines

AE monitoring and reporting is a routine part of every clinical study. All Investigators, clinical site staff, and the Sponsor's employees share in the responsibility for reporting AEs and SAEs.

10.1.1 Sponsor Guidance

The Sponsor is required to notify all participating Investigators and FDA in a written Investigational New Drug (IND) safety report of any AE associated with the use of the investigational drug that is both serious and unexpected, and any finding from tests in laboratory animals that suggests a significant risk for human patients (21 CFR 312.32(c)(1)(i)(A),(B)). The Sponsor is required to keep Investigators informed of new observations discovered by, or reported to the Sponsor regarding the study drug particularly with respect to AEs and safe use (21 CFR 312.55).

10.1.2 Investigator Guidance

The Investigator is required to promptly report to the Sponsor any AE that may reasonably be regarded as caused by or probably caused by the investigational drug. If the AE is alarming, the Investigator shall report the adverse effect immediately to the Sponsor (21 CFR 312.64). Further, Investigators are required to promptly report to the Human Research Ethics Committee (HREC) and/or Institutional Review Board (IRB) as applicable all unanticipated problems involving risks to human patients or others including AEs that should be considered unanticipated problems (21 CFR 56.108(B)(1), 21 CFR 312.53(c)(1)(vii), and 21 CFR 312.66). All Investigators and clinical site staff who learn about or are notified of a SAE must collect and promptly report to the Sponsor (within 24 hours) data according to the study protocol and relevant regulations (21 CFR 312.64, 21 CFR 312.32).

10.2 Adverse Events (AE or Adverse Experience)

Adverse events include any untoward medical occurrence associated with the use of a drug in humans, whether or not considered drug related. Therefore, an AE can be any unfavorable and unintended sign (including an abnormal laboratory finding), symptom, or disease temporally associated with the use of the investigational drug, whether or not considered related to the study drug (attribution of 'unrelated', 'unlikely', 'possible', 'probable', or 'definite') (International Conference on Harmonization [ICH] E2A, E6). Adverse events include:

- Exacerbation of a pre-existing disease.
- Increase in frequency or intensity of a pre-existing episodic disease or medical condition.
- Disease or medical condition detected or diagnosed after study drug administration even though it may have been present prior to the start of the study.
- Continuous persistent disease or symptoms present at Baseline that worsen following the start of the study.
- Lack of efficacy in the acute treatment of a life-threatening disease.
- Events considered by the Investigator to be related to study-mandated procedures.
- Abnormal assessments (e.g., change on physical examination, ECG findings), if they represent a clinically significant finding that were not present at Baseline or worsened during the course of the study. ALS related findings should not be documented as an AE if deemed by an Investigator as related to disease progression. Finding should be noted on exams as related.
- Laboratory test abnormalities if they represent a clinically significant finding, symptomatic or not, which was not present at Baseline or worsened during the course of the study or led to dose reduction, interruption, or permanent discontinuation of the study drug.

Adverse events do not include:

- Medical or surgical procedure not mandated in the protocol (e.g., surgery, endoscopy, tooth extraction). However, the event leading to the procedure is an AE. If this event is serious, the procedure must be described in the SAE narrative on the SAE eCRF.
- Pre-existing disease or medical condition that does not worsen. However, if the condition deteriorates at any time during the study, it should be recorded as an AE.
- Situations in which an adverse change did not occur (e.g., hospitalizations for cosmetic elective surgery or for social and/or convenience reasons).
- Overdose of either study drug without any signs or symptoms. However, overdose of study drug must be reported in the Study Drug Log.

The occurrence of an AE may come to the attention of study personnel during the study visits, during interviews of a study recipient presenting for medical care, or upon review by a study monitor. Information to be collected and recorded include: event description, time of onset, clinician's assessment of severity, relationship to study product (assessed only by those with the training and authority to make a diagnosis, which would include MD, PA, Nurse Practitioner, DO, or Physician Assistant), and time of resolution/stabilization of the event.

All AEs occurring during the study including local and systemic reactions must be documented appropriately regardless of relationship and recorded on the relevant eCRF. If there is any doubt as to whether a clinical observation is an AE, the event should be reported.

10.2.1 Intensity of Adverse Events

The intensity of all AEs will be graded for severity. The investigating clinician will assess all AEs by grading the AE on a three-point scale of 1) mild, 2) moderate, and 3) severe; which are defined as follows:

- **Mild events**: require minimal or no treatment and do not interfere with the patient's daily activities.
- **Moderate events**: result in a low level of inconvenience or concern with the investigational drug. Moderate events may cause some interference with daily functioning.
- **Severe events**: interrupt a patient's usual daily activity and may require systemic drug therapy or other treatment. Severe events are usually incapacitating.

If the intensity of an AE worsens during study drug administration, only the worst intensity should be reported on the relevant AE eCRF. If the AE lessens in intensity, no change in the severity is required. AEs characterized as intermittent require documentation of onset and duration of each episode.

The AE intensity definitions do not apply to clinically significant and asymptomatic laboratory test abnormalities or abnormal assessments (e.g., ECG findings) considered as AEs. The Investigator should tick "non-applicable" on the AE page of the eCRF when identifying the intensity of the AE.

Of note, mild, moderate, or severe AE classifications may or may not be serious (see SAE section). These terms are used to describe the intensity of a specific adverse event (as in mild, moderate, or severe nausea). For example, nausea lasting several hours may be rated as severe, but may not be clinically serious. Alternatively, a fever of 39°C that is not considered severe, may however become serious, if it prolongs hospitalization. Seriousness rather than severity serves as a guide for defining regulatory reporting requirements.

10.2.2 Relationship and Attribution of the AE to Study Drug

10.2.2.1 Relationship to Study Drug

Each adverse event must be assessed by the Investigator as to whether or not there is a reasonable possibility of causal relationship to the study drug, and reported as either related or unrelated. The Investigators assessment of an AE's relationship to the study drug is part of the documentation process, but it is not a factor in determining what is or is not reported in the study.

- Related to Study Drug: This category applies to any AE (whether serious or not) that appears to have a reasonable possibility of causal relationship to the use of the study drug (i.e., a relationship cannot be ruled out). Guidelines to determine whether an event might be considered related include but are not limited to the following:
  - The event occurred in close temporal relationship to study drug administration.
  - The event diminished or disappeared when treatment with the study drug was down titrated, interrupted, or discontinued.
  - The event reoccurred when treatment was reintroduced.
  - Environmental factors such as clinical state and other treatments could not equally have caused the event.
- Unrelated to Study: There is not a reasonable possibility that the administration of the study product caused the event (see above guidelines).

10.2.2.2 Attribution to The Study Drug

After identifying and grading the event, the clinical Investigator must assign an attribution to the AE using the following attribution categories described in the table below. AEs listed as 'possibly, probably' or definitely' related to the investigational drug are considered to have a suspected 'reasonable causal relationship' to the investigational agent (ICH E2A).

Table 23. AE Attribution to the Study Drug

Relationship to Study Drug	Attribution	Description
Unrelated	Unrelated	The AE is clearly NOT related to the study drug
	Unlikely	The AE is doubtfully related to the study drug
Related	Possible	The AE may be related to the study drug
	Probable	The AE is likely related to the study drug
	Definite	The AE is clearly related to the study drug

10.3 Serious Adverse Events

The International Conference on Harmonization (ICH) guidelines define a serious adverse event (SAE) as an AE or suspected adverse reaction that in the view of either the Investigator or Sponsor results in any of the following study patient outcomes:

- Death,
- A life-threatening AE,
- Inpatient hospitalization or prolongation of existing hospitalization,

- A persistent or significant incapacity or substantial disruption of the ability to conduct normal life functions,
- A congenital anomaly/birth defect, or
- An important medical event, requiring medical or surgical intervention (treatment) to prevent at least one of the outcomes listed above.

Life threatening refers to an event in which the patient was at risk of death at the time of the event. It does not refer to an event that hypothetically might have caused death had it been more severe.

Important medical events that do not result in death, are not life threatening, or do not require hospitalizations may be considered serious when, based upon appropriate medical judgment, they may jeopardize the patient and may require medical or surgical intervention to prevent one of the outcomes listed in the SAE definition. Examples of such medical events include allergic bronchospasm requiring intensive treatment in an emergency room or at home, a blood dyscrasia or convulsion that does not result in inpatient hospitalization, or the development of drug dependency or drug abuse.

Complications that occur during hospitalization may be AEs or SAEs depending upon Investigator judgment (i.e., if a complication prolongs hospitalization it would be considered an SAE). However, the following reasons for hospitalizations are not considered AEs, and are therefore also not SAEs:

- Hospitalizations for cosmetic elective surgery, or social and/or convenience reasons.
- Standard monitoring of a pre-existing disease or medical condition that did not worsen (e.g., hospitalization for coronary angiography in a patient with stable angina pectoris).
- Elective treatment of a pre-existing disease or medical condition that did not worsen (e.g., elective hip replacement for arthritis).

10.3.1 Serious Adverse Events Related To Study-Mandated Procedures

A SAE is defined as related to study-mandated procedures if it appears to have a reasonable possibility of a causal relationship (i.e., a relationship cannot be ruled out) to procedures other than administration of the study drug. Examples of study-mandated procedures include blood sampling, car accident on the way to the study unit for a study visit, etc.

10.4 Reporting Requirements for AEs and SAEs

10.4.1 Reporting of Adverse Events

All AEs are collected from the time the informed consent is signed up to 2 weeks after the last study drug dose, or leading to the premature discontinuation of study drug must be recorded on the appropriate AE pages of the eCRF.

Information to be collected includes event description, date of onset, Investigator assessment of severity, Investigator assessment of relationship to study product, date of resolution of the event, seriousness, and outcome.

10.4.2 Reporting Abnormal Laboratory Test Values or Abnormal Clinical Findings

Collection of specific safety laboratory data is outlined in the study visit schedule. Toxicity tables are based on the FDA toxicity tables developed for normal healthy adult volunteers. Laboratory values and abnormal clinical findings that are abnormal based on the toxicity tables must be reported on the appropriate AE eCRF.

10.4.2.1 Reporting of AE Intensity and Causality

Only licensed study clinicians (i.e., Medical Doctor, Doctor of Osteopathy, Nurse Practitioner, Physician's Assistant) should assess the intensity of non-serious AEs. Similarly, only licensed study clinicians as identified on Form FDA 1572 or per ICH E6 standards should assess the causality of AEs.

10.4.2.2 Follow-up of AEs

All AEs occurring during the AE reporting period of the study will be documented appropriately regardless of relationship. All AEs occurring during the treatment period will be followed to adequate resolution or until the patient is considered stable.

Adverse events still ongoing after study drug discontinuation for a given patient will be followed until 28 days after study drug discontinuation or until AE resolution or patient stabilization.

Resolution of an AE is defined as the return to pre-treatment status or stabilization of the condition to the Investigator and/or Sponsor's satisfaction with the expectation that the condition or abnormality may remain chronic. Follow-up procedures, evaluations, and outcomes will be recorded on the patient's eCRF.

10.4.3 Reporting Requirements For SAEs

All SAEs occurring during the course of the study (e.g., signing of informed consent by patient to 28 days following the last dose of the study drug) and any SAEs, considered causally related to study drug that occur following completion of the study, must be reported to the Sponsor.

10.4.3.1 Reporting of SAEs

All serious adverse events that occur during the course of study will be recorded on the appropriate eCRF (e.g., SAE eCRF) and will be reported to Sponsor's drug safety physician.

Any AE that meets a protocol-defined criterion as serious must be submitted immediately (within 24 hours of site or Investigator awareness) on an SAE form to the Sponsor's drug safety physician at the following address:

Chief Medical Officer
Tel: +1 (801) 676-9695
Email: safety@clene.com

Information to be collected includes unique patient ID, event description, date of onset, Investigator assessment of severity, Investigator assessment of relationship to study product, date of resolution of the event, seriousness, and outcome.

Other supporting documentation of the event may be requested by the Sponsor and should be provided as soon as possible. The Sponsor's drug safety physician will review and assess the SAE for regulatory reporting and potential impact on study patient safety and protocol conduct.

10.4.3.2 SAE Reporting Periods

- Screening period: SAEs occurring between signing the Informed Consent Form and study drug initiation should be reported in the eCRF.
- Treatment Period: All SAEs, regardless of causal relationship, must be reported, including those related to study-mandated procedures. Those SAEs occurring during study drug administration, i.e., between study drug initiation and 14 days after study drug discontinuation, are defined as treatment-emergent SAEs. These SAEs are reported on SAE forms and also on AE pages in the eCRF. They are therefore entered into both the drug safety and clinical databases, and must be reconciled before study closure.
- Open-Label Extension Period: All SAEs, regardless of causal relationship, must be reported, including those related to study-mandated procedures. Those SAEs occurring during study drug administration, i.e., between study drug initiation and 14 days after study drug discontinuation, are defined as treatment-emergent SAEs. These SAEs are reported on SAE forms and also on AE pages in the eCRF. They are therefore entered into both the drug safety and clinical databases, and must be reconciled before study closure.
- Follow-Up Period: All SAEs, regardless of causal relationship, occurring 14 days after study drug discontinuation until 28 days after study drug discontinuation must be reported.
- Post Follow-Up Period: If the Investigator becomes aware of a new SAE that is suspected of being causally related to the study drug occurring after 28 days of follow-up or post-study completion, the Investigator will report the event within 24 hours to the Sponsor's drug safety

physician. These SAEs are only entered in the drug safety pharmacovigilance database, and therefore will not affect study closure.

10.4.3.3 SAE Reporting Procedures

- All SAEs must be reported by the Investigator to drug safety physician within 24 hours of the Investigator's first knowledge of the event.
- All SAEs must be recorded on the appropriate SAE forms, irrespective of the study drug received by the patient, and whether this event is considered by the Investigator to be related to study drug. These SAE forms must be faxed and emailed to Clene's drug safety physician.
- The Investigator must complete the SAE form in English (unless otherwise specified), and must assess the relationship of the event to study drug. Such reports will be followed by detailed descriptions that may include copies of hospital case reports, autopsy reports, hospital discharge summaries and other documents when requested and applicable.
- Follow-up information about a previously reported SAE must also be reported within 24 hours of receiving it. The drug safety physician may contact the Investigator to obtain further information.
- The intensity of SAEs may only be assessed by a licensed study physician as described previously. The causality of SAEs may only be assessed by a licensed physician identified on the Form FDA 1572 or per ICH E6 standards.

10.4.3.4 SAE Regulatory Reporting

Following notification from the Investigator, the Sponsor will expedite reporting any suspected adverse reaction that is both serious and unexpected to Health Authorities, HREC, IRBs, and other Investigators, as appropriate.

The Sponsor will report an AE as a suspected adverse reaction only if there is evidence to suggest a causal relationship between the drug and the AE. Sponsor will notify FDA and all participating Investigators (i.e., all Investigators to whom the Sponsor is providing drug under its INDs or under any Investigator's IND) in an IND safety report of potential serious risks from clinical trials or any other source, as soon as possible, but in no case later than 15 calendar days after the Sponsor determines that the information qualifies for reporting as specified in 21 CFR Part 312.32. Further, Sponsor will notify FDA of any unexpected fatal or life-threatening suspected adverse reaction as soon as possible but in no case later than 7 calendar days after the Sponsor's initial receipt of the information. Relevant follow-up information to an IND safety report will be submitted as soon as the

information is available. Upon request from FDA, Sponsor will submit to FDA any additional data or information that the agency deems necessary, as soon as possible, but in no case later than 15 calendar days after receiving the request.

Un-blinding of serious and unexpected AEs will be performed as necessary and appropriate.

All serious adverse events designated as "not related" to study product(s), will be reported to the FDA at least annually in a summary format.

General study reporting periods and safety reporting requirements are summarized in the table below.

Table 24. Summary of Study Safety Reporting Periods

Study Periods:	Screening	Treatment/OLE	Post Follow-up
**Timeframe**	From signature of informed consent to study drug initiation	During study drug administration plus 28 days	After 28 days
**AE/SAE Reporting on AE eCRF**	None	All AEs/SAEs[1]	None
**SAE Reporting on SAE eCRF**	Only if related to study-mandated procedures	All SAEs	If considered causally related to the study medication
**Reconciliation[2]**	Not applicable	Yes	Not applicable
**Clinical Study Report/Final Study Report**	AEs and SAEs may be described	Analyzed	AEs and SAEs may be described

[1]Adverse events still ongoing after study drug discontinuation for a given patient must be followed until 28 days after study drug discontinuation or until resolution or stabilization or until the event is otherwise explained.
[2]Reconciliation between clinical and drug safety databases.

10.5 Pregnancy

10.5.1 Prevention of Pregnancy During the Study

Women should not become pregnant during the study and up to 6 months following study drug discontinuation. Accordingly, women of childbearing potential should take appropriate precautions to prevent pregnancy during the study. If a woman becomes pregnant while on study drug, no further treatment will be administered. In the event of a pregnancy, all study-mandated blood samples will be obtained and the patient will continue in follow-up for safety events.

10.5.2 Reporting of Pregnancy

Irrespective of the treatment received by the patient, any pregnancy which occurs in a study patient or the partner of a male study patient, which occurs during study drug administration or 6 months

following study drug discontinuation must be reported within 24 hours of the Investigator's knowledge of the event.

Pregnancies must be reported to the Sponsor's drug safety physician and on the AE page of the eCRF, as applicable.

10.5.3 Pregnancy Follow-Up

Pregnancies will be followed to pregnancy outcome pending the patient's permission and reported to the Sponsor's drug safety physician. Follow-up information will only be entered in the drug safety database, and hence will not affect study closure.

10.6 Study Halting Rules

Study halting rules will be outlined in the DSMB charter.

10.7 Clinical Monitoring

10.7.1 Site Monitoring Plan

Site monitoring will be conducted to ensure that human patient protection, study procedures, laboratory procedures, study drug administration, and data collection processes are of high quality and meet Sponsor, International Conference on Harmonization (ICH)/Good Clinical Practice (GCP) guidelines and applicable regulatory requirements, and that the study is conducted in accordance with the protocol and Sponsor's SOPs. The Sponsor or its designee will conduct site-monitoring visits as specified in the monitoring plan.

Monitoring visits will include, but are not limited to, review of regulatory files, accountability records, eCRFs, patient source records (i.e., electronic medical records), informed consent forms (ICFs), medical and laboratory reports, and protocol compliance. Study monitors will meet with Investigators to discuss any problems and actions to be taken and document visit findings and discussions.

11 STATISTICS AND DATA MANAGEMENT

A statistical analysis plan (SAP) will provide details of planned analyses and summary documents, such as tables, listings and figures. All variables will be analyzed descriptively with appropriate statistical methods: categorical variables by frequency tables and continuous variables by sample statistics (i.e., mean, SD, minimum, median, quartiles and maximum). An overview of the planned analyses is provided here. However, final analyses are not limited to the summaries described herein but will be defined in the SAP prior to final database lock.

11.1 Determination of Sample Size and Analyses

Based on previously reported longitudinal studies of MUNIXscore(4) in ALS patients (Neuwirth et al., 2015); it is assumed the common standard deviation for the MUNIXscore(4) is 19.8% with a placebo deterioration of 38.4% (e.g., 61.6% of the baseline index value) at Week 36. The active treatment group deterioration is estimated at 19.2% (e.g., 80.8% of the baseline index value) at Week 36. Accordingly, sample size calculations were based on the following criteria:

a) Mean difference between the MUNIX(4)score indexed value in the change from baseline between active and placebo of 19.2% at Week 36;

b) common standard deviation (SD) of 19.8%;

c) α value of 0.05;

d) β power of 0.8; and

e) 12.5% dropout rate.

At a 1:1 (CNM-Au8 30 mg: Placebo) allocation, 80% power and 5% statistical significance rate, an estimated 36 evaluable patients will be required for this study. With a 12.5% estimated non-evaluable rate, it is planned to assign 42 patients (21 active:21 placebo) to randomised treatment.

11.2 Analysis Populations

The following patient sets are applicable to this study:

- **Intent to Treat Analysis Set** – The Intent To Treat Set (ITT) will consist of Screened patients for whom a randomisation number has been assigned at Visit 0.
- **Safety Analysis Set** – The Safety Analysis Set (SAS) will consist of patients in the Randomised Set who receive at least 1 dose of investigational product.
- **Partial Analysis Set** – The Partial Analysis Set (PAS) will consist of all patients in the Safety Set with at least 1 post-baseline EMG measurement.

- **Per Protocol Set –** The Per Protocol Set (PPS) will consist of all patients in the Safety Set who have completed 36-weeks of treatment and all of the Primary and Secondary endpoints and who are treatment adherent per Section 8.5.

11.3 Baseline Characteristics and Patient Disposition

Overall Baseline and demographic data will be summarized using descriptive statistics. Patient disposition (e.g., the number of patients enrolled, completed, and discontinued) will be summarized and medical history and physical examination findings will be listed.

11.4 Prior and Concomitant Medications

Medications taken after the patient receives study drug through Follow-Up will be listed by treatment, dose level and patient.

11.5 Pharmacokinetic Analyses

Pharmacokinetic parameters will be derived using noncompartmental analyses employing appropriate software and graphing tools (e.g. WinNonlin® Professional version 6.3 [Pharsight Corp], SAS® for Windows® Version 9.4, SigmaPlot for Windows Version 12.5; or higher versions).

Au concentrations will be summarized using descriptive statistics (including N, mean, SD, coefficient of variation (CV%), median, minimum, and maximum) for each treatment. Concentrations below the limit of quantification (BLQ) will be treated as zero for the computation of descriptive statistics and for construction of mean concentration-time profiles. Concentrations assigned a value of missing will be omitted from the calculation of descriptive statistics.

The following PK parameters will be estimated by noncompartmental methods from whole blood samples. Actual elapsed time from dosing will be used to estimate all individual PK parameters.

Table 25. Summary of PK Parameters

Parameter	Description
C_{max}	Maximum observed plasma concentration
T_{max}	Time of maximum concentration (h), obtained directly from the observed concentration versus time data.
CL/F	Apparent systemic clearance
$AUC_{(0-24)}$	Area under the plasma concentration-time curve from time 0 to the end of the 24-hour dosing interval

Derived PK descriptive statistics will be tabulated by dosing group and summary statistics. Descriptive statistics for PK parameters will include the arithmetic and geometric mean (for C_{max}, T_{max}, $AUC_{(0-24)}$, and CL/F), CV%, SD of the arithmetic mean, median, minimum, maximum, and N.

Population PK and PK/PD models may be developed to address objectives that require an integrative interpretation of the study results. These include assessment of the dose proportionality, investigation of the nature of the PK/PD relationship, and the use of study results as part of a larger model-based data analysis. If population PK/PD models are developed, a separate Pharmacometric Analysis Plan will be written.

11.6 Safety Analyses

All safety summaries will be descriptive; no statistical significance tests will be performed on safety data and will be based on the safety population.

Safety assessments include extent of exposure, incidence of treatment-emergent AEs, drug-related AEs, deaths, SAEs, AEs leading to discontinuation from the study, Falls Questionnaire, and the Columbia Suicidality Severity Rating Scale (C-SSRS). Changes from baseline in clinical laboratory results, ECGs and vital signs will also be summarized by treatment group and time point.

At each time point, absolute values and change from baseline of each safety endpoint will be summarized with n, mean, SD, standard error of the mean (SEM), median, Min, and Max values. The number of available observations and out-of-range values (absolute and in percentage) will be presented. Values outside the reference range will be flagged in the listing. ‘H’ and ‘L’, denoting values above or below the Investigator reference range (when present), will flag out-of-range results.

The AE coding dictionary for this study will be Medical Dictionary for Regulatory Activities (MedDRA). It will be used to summarize AEs by primary system organ class (SOC) and preferred term (PT).

11.7 Efficacy Analyses

The primary endpoint, the mean change in the MUNIXscore(4) value will be analysed using Mixed Model for Repeated Measures (MMRM) with treatment (Week 12, Week 24, and Week 36) as fixed effects and Baseline ΔFS, Baseline ALSFRS-R scores, and bulbar vs. limb onset as covariates. Estimates of least-square means, standard errors and 95% confidence intervals will be presented by treatment group. For the active treatment versus placebo comparisons, the least square mean difference, the standard error of the difference, and the 95% confidence interval of the difference will be presented.

Descriptive summaries of the absolute and change from baseline mean scores by week will be presented for all efficacy measures. Specific analysis plans for the secondary and exploratory efficacy plans will be described in the SAP. Primary and secondary efficacy analyses will be based on the intent to treat (ITT) population. Additional exploratory analyses may examine different study population sets as described in Section 11.2.

11.8 Interim analyses

No pre-specified interim analyses are planned per protocol.

The Independent Data Safety Monitoring Board (DSMB) will meet at regularly scheduled intervals as detailed in the DSMB Charter. The following un-blinded data may be reviewed by the DSMB based on the standards detailed in the DSMB charter (e.g., safety signals, trends warranting further un-blinded review) and used to determine if study continuation may occur:

- AEs and SAEs
- Physical examination results
- Clinical laboratory tests

If un-blinded data are required, this data shall only be reviewed in a closed session of DSMB and not available to the Sponsor for review.

11.9 Data Handling and Quality Assurance

11.9.1 Data Recording

Limited data may be entered solely into the eCRF (e.g., ethnic group). All other data must have source documentation available, which will be provided by the Sponsor. A source document checklist will be used at the site to identify the source data for all data points collected, and the study monitor will work with the site to complete the source documentation verification.

11.9.1.1 Data Recorded From Screening Failures

For patients who do not meet selection criteria (Screen Failures), source data will be recorded and entered into the eCRF, including the reason for the screen failure, and demographic information (e.g., patient number, date of birth/age, sex, height, weight, race and ethnicity), and the date of the study visit.

For patients classified as Screen Failures with an SAE during the Screening visit (Visit -1), the following additional data should be collected in the eCRF:

- All information about the SAE
- Other related including:
    - Concomitant medication
    - Medical history
    - Any other information needed to complete the SAE eCRF page

11.9.2 Data Monitoring

In accordance with applicable regulations, Good Clinical Practice (GCP), and the Sponsor's and/or the Clinical Research Organization (CRO) procedures, study monitors will contact the site prior to the start of the study to review with the site staff the protocol, study requirements, and their responsibilities to satisfy regulatory, ethical, and Sponsor's requirements. When reviewing data collection procedures, the discussion will also include identification and documentation of source data items.

The Sponsor and/or CRO monitor will visit clinical sites periodically to monitor study activity to verify that the:

- Data are authentic, accurate, and complete
- Safety and rights of patients are being protected
- Study is conducted in accordance with the currently approved protocol (including study treatment being used in accordance with the protocol)
- All other study agreements, GCP, and all applicable regulatory requirements are being met.

The site Investigator and the head of the medical institution, as applicable, agree to allow the Sponsor and/or CRO study monitor direct access to all relevant documents at the clinical site.

11.9.3 Data Collection and Management

Data collection will be conducted via a validated electronic data collection (EDC) system, which will be managed by the Sponsor's biostatistics and data management CRO.

Patient data necessary for analysis and reporting will be entered and/or transmitted into a validated database or data system. Clinical data management will be performed in accordance with applicable Sponsor and CRO standards for data cleaning procedures. This is applicable for data recorded on the eCRF as well as for data from other sources (e.g., IRT, laboratory, ECG). For data coding (e.g., AEs, medication), internationally recognized and accepted dictionaries will be used. Data will be electronically verified through use of programmed edit checks specified by the clinical team. Discrepancies in the data will be brought to the attention of the clinical team, and investigational site

personnel, as applicable. Resolutions to these issues will be reflected in the database. An audit trail within the system will track all changes made to the data.

11.9.4 Data Audit and Inspection

To ensure compliance with GCP and regulatory requirements, a member of the Sponsor's quality assurance unit, or designated CRO representative, may conduct an audit to assess the performance of the study at the study site and of the study documents originating there. The site Investigator and institution will be informed of the audit outcome. In addition, inspections by regulatory health authority representatives and/or HREC(s)/IRB(s) are possible. The Investigator should notify the Sponsor immediately of any such inspection. The Investigator/institution agrees to allow the auditor or inspector direct access to all relevant documents and to provide sufficient time and staff support to review any findings and any discuss any issues. Audits and inspections may occur at any time during or after completion of the study.

11.9.5 Data Archiving

Essential site documents shall be archived safely and securely in such a way that ensures that they are readily available upon regulatory authorities' request. Patient files will be archived according to local regulations and in accordance with the maximum period of time permitted by the hospital, institution or private practice associated with the study. Where the archiving procedures do not meet the minimum timelines required by the Sponsor, alternative arrangements must be made to ensure the availability of the source documents for the required period. The Investigator/institution shall notify the Sponsor if the archival arrangements change (e.g., relocation or transfer of ownership).

The Investigator site file is not to be destroyed without the Sponsor's written approval.

12 RESPONSIBILITIES, ETHICS, AND LEGAL ASPECTS

12.1 Investigator Responsibilities

12.1.1 Good Clinical Practice

The Investigator will ensure that this study is conducted in full compliance with the principles of the "Declaration of Helsinki" (as amended in Edinburgh, Tokyo, Venice, Hong Kong, and South Africa), ICH guidelines, or with the laws and regulations of the country in which the research is conducted, whichever affords the greater protection to the study Patient. For studies conducted under a United States IND, the Investigator will ensure that the basic principles of "Good Clinical Practice," as outlined in 21 CFR 312, subpart D, "Responsibilities of Sponsors and Investigators," 21 CFR, Part 50, 1998, and 21 CFR, Part 56, 1998, are adhered to. This study will be conducted in compliance with 21 CFR, Part 320, 1993, "Retention of Bioavailability and Bioequivalence Testing Samples."

12.1.2 Institutional Review Board (IRB)/Independent Ethics Committee (IEC) Approval

The Investigator will submit this protocol and any related documents to an IEC, Human Research Ethics Committee (HREC), and the Competent Authority (CA) as applicable. Approval from the EC and the statement of no objection from the CA must be obtained before starting the study, and should be documented in a dated letter/email to the Investigator, clearly identifying the study, the documents reviewed and the date of approval. A list of EC members must be provided, including the functions of these members. If study staff were present, it must be clear that none of these persons voted. Modifications made to the protocol after receipt of the EC approval must also be submitted as amendments by the Investigator to the EC in accordance with local procedures and regulations.

12.1.3 Informed Consent

It is the responsibility of the Investigator or designee to obtain written informed consent from each individual participating in this study after adequate explanation of the aims, methods, objectives, and potential hazards of the study and prior to undertaking any study-related procedures. The Investigator or designee must utilize an HREC/IRB-approved informed consent form (ICF) for documenting written informed consent. Each informed consent will be appropriately signed and dated by the patient and the person obtaining consent. A patient may enter the study only if the patient or legal representative voluntarily agrees to sign the informed consent form. A copy of the signed consent form will be provided to the patient.

If informed consent is obtained on the date that Screening study procedures (Visit -1) are performed, the study record or patient's clinical record must clearly show that informed consent was obtained prior to these procedures.

If the patient is not capable of providing a signature, a verbal statement of consent can also be given in the presence of an impartial witness (e.g., independent of the Sponsor and the Investigator). This is to be documented by a signature from the informing physician as well as by a signature from the witness.

The ICF and any other written information provided to patients or the patient's legal representative will be revised whenever important new information becomes available that may be relevant to the patient's consent, or there is an amendment to the protocol that necessitates a change to the content of the patient information and/or the written ICF. The Investigator will inform the patient or legal representative in a timely manner and will ask patients to confirm their participation in the study by signing the revised informed consent form. Any revised written informed consent form and written information must receive the HREC/IEC/IRB's approval prior to use.

12.1.4 Adherence to Protocol

The Investigator may not modify or alter the procedures described in this protocol and is required to strictly adherence to all specifications laid down in this protocol for all aspects of study conduct.

Neither the Sponsor nor the Investigator will implement protocol amendments or modifications to the study protocol without agreement by both parties. However, the Investigator or the Sponsor may implement a deviation from, or a change to the protocol to eliminate an immediate hazard to patients without prior HREC/IEC/IRB/Sponsor approval. In this case, the implemented deviation or change, the reasons for it, and if appropriate, the proposed protocol amendment should be submitted to the IEC/IRB/head of medical institution and Sponsor as soon as possible.

Any deviations from the protocol must be explained and documented by the Investigator.

12.1.5 Confidentiality

The Investigator must assure that patients' anonymity will be strictly maintained and that their identities are protected from unauthorized parties. Only patient initials and an identification code (i.e., not names) should be recorded on any form submitted to the Sponsor and IRB/HREC. The Investigator must keep a patient log showing codes, names, and addresses for all patients screened and for all patients enrolled in the study.

12.1.6 Study Files and Retention of Records

The Investigator must maintain adequate and accurate records to enable the conduct of the study to be fully documented and the study data to be subsequently verified. These documents should be

classified into 2 separate categories (although not limited to) the following: (1) Investigator's study file, and (2) Patient clinical source documents.

The Investigator's study file will contain the protocol/amendments, CRF and query forms, IRB/HREC approval with correspondence, informed consent, drug records, staff curriculum vitae and authorization forms, and other appropriate documents and correspondence.

Patient clinical source documents would include (although is not limited to) the following: patient hospital/clinic records, physician's and nurse's notes, appointment book, original laboratory reports, ECG, electroencephalogram (EEG), EMG, X-ray, MRI, pathology and special assessment reports, consultant letters, screening and enrollment log, etc.

All clinical study documents must be retained by the Investigator until at least two years after the last approval of a marketing application in an International Conference on Harmonization (ICH) region (i.e., United States, Europe, or Japan) and until there are no pending or contemplated marketing applications in an ICH region; or, until two years after the IND is discontinued and regulatory authorities have been notified. The Investigator must notify Sponsor prior to destroying any clinical study records.

Should the Investigator wish to move study records to another location, arrangements must be made to store these in sealed containers so that they can be returned sealed to the Investigator in case of a regulatory audit. Where source documents are required for the continued care of the Patient, appropriate copies should be made for storage outside of the site.

12.1.7 Case Report Forms

For each Patient who receives study drug, a CRF must be completed and signed (electronically signed if eCRF) by the Principal Investigator or sub-Investigator within a reasonable period after data collection. This also applies to records for those patients who fail to complete the study. If a patient withdraws from the study, the reason must be noted on the eCRF. If a patient is withdrawn from the study because of a treatment-limiting adverse event, thorough efforts should be made to clearly document the outcome.

12.1.8 Drug Accountability

The Investigator or designee (i.e., pharmacist) is responsible for ensuring adequate accountability of all used and unused study drug. This includes acknowledgment of receipt of each shipment of study product (quantity and condition) and Patient dispensing records and returned or destroyed study product. Dispensing records will document quantities received and quantities dispensed to Patients,

including lot/batch number, date dispensed, Patient identifier number, Patient initials, and the initials of the person dispensing or witnessing dispensing of the study medication.

At study initiation, the monitor will evaluate the site's SOP for study drug disposal/destruction in order to ensure that it complies with study requirements. Following unblinded study drug reconciliation by the unblinded monitor, the study site will be instructed by the Sponsor to destroy and unused or returned study drug.

12.1.9 Inspections

The Investigator will provide access to source documents and all study records for this study to appropriately qualified personnel from the Sponsor or its representatives, and to regulatory authority inspectors.

12.2 Sponsor Responsibilities

12.2.1 Study Materials and Instructions

It is the Sponsor's responsibility to ensure that the Investigator is provided with the documents and other study materials necessary to conduct the study. Examples of those materials include, but are not limited to: protocol, Investigator's Brochure, study drug, eCRF, SAE collection forms, logs, etc. The Sponsor will also provide training and oversight through site and medical monitoring.

12.2.2 Protocol Modifications

Protocol modifications, except those intended to reduce immediate risk to study patients, will be made by Sponsor-initiated amendment. IRB/HREC approval must be obtained before changes can be implemented except for non-substantial amendments (e.g., changes in study staff or contact details or minor changes in the packaging or labeling of the investigational product) as described below.

Administrative or logistical minor changes will require a non-substantial amendment. Non-substantial amendments will be approved (signed) by the Investigator(s) and will be recorded and filed by the Investigator/Sponsor and the Ethics Committee (EC) and the Competent Authority (CA) may or may not be notified per local regulatory guidelines. The implementation of a non-substantial amendment can be done without notification or approval to the appropriate EC or CA.

The following amendments will be regarded as non-substantial:

- Change in timing of the samples;
- Changes in assay-type and/or institution where an assay will be performed, provided that validated assays will be used;

- Editorial changes to the patient information sheets;
- Determination of additional parameters in already collected materials, which agree with the study objectives and do not provide prognostic or genetic information;
- Other statistical analyses than described in the protocol.

12.2.3 Insurance

The Sponsor maintains clinical trial insurance coverage for this study in accordance with the laws and regulations of the country in which the study is performed.

12.2.4 Premature Study Termination

The Sponsor has the right to prematurely close this study or, if applicable, individual segments of the study, including but not limited to: treatment arms, study sites, titration steps, and study procedures; if at any time the risk-benefit profile of the investigational product, center conduct, or specific study treatment segment becomes unacceptable due to:

- Safety findings from this study (e.g., SAEs)
- Procedural issues or protocol violations from a study site that potentially affects patient safety
- Safety findings from other parallel studies in different patient populations
- Results from animal toxicology studies (e.g., toxicity, teratogenicity, carcinogenicity, or reproduction toxicity).

Should premature termination be necessary, the Sponsor and Investigator will arrange discontinuation procedures and notify the appropriate regulatory authorities, IRBs and HRECs. In terminating the study, the Sponsor and the Investigator will assure that adequate consideration is given to the protection of the Patients' interests.

12.3 Joint Investigator/Sponsor Responsibilities

12.3.1 Access to Information for Monitoring

In accordance with International Conference on Harmonization Good Clinical Practice (ICH-GCP) guidelines, the study monitor must have direct access to the Investigator's source documentation in order to verify the data recorded in the CRFs for consistency.

The monitor is responsible for routine review of the CRFs at regular intervals throughout the study, to verify adherence to the protocol, and the completeness, consistency and accuracy of the data being entered on them. The monitor should have access to any patient records needed to verify the entries

on the CRFs. The Investigator agrees to cooperate with the monitor to ensure that any problems detected in the course of these monitoring visits are resolved.

12.3.2 Study Report and Publications

After conclusion of the study, Investigators in this study may communicate, orally present, or publish in scientific journals or other scholarly media only after the following conditions have been met:

- The results of the study in their entirety have been publicly disclosed by or with the consent of Sponsor in an abstract, manuscript, or presentation form; or
- The study has been completed for at least 2 years.

No such communication, presentation, or publication will include Sponsor's confidential information.

Any information, inventions, or discoveries (whether patentable or not), innovations, suggestions, ideas, and reports, made or developed by the Investigator(s) as a result of conducting this study shall be promptly disclosed to the Sponsor and shall be the sole property of the Sponsor. The Investigator agrees, upon the Sponsor's request and at the Sponsor's expense, to execute such documents and to take such other actions, as the Sponsor deems necessary or appropriate to obtain patents in the Sponsor's name covering any of the foregoing.

The Investigator will submit any proposed publication or presentation along with the respective scientific journal or presentation forum to the Sponsor at least 30 days prior to submission of the publication or presentation. The Investigator will comply with Sponsor's request to delete references to its confidential information (other than the study results) in any paper or presentation and agrees to withhold publication or presentation for an additional 60 days in order to obtain patent protection if deemed necessary.

13 PROTOCOL AMENDMENTS

A protocol amendment to version 1.0 of clinical protocol CNMAu8.205 was performed on 06September2019 to included added safety follow-up telephone calls as requested by the reviewing Human Research Ethics Committee (Sydney Local Health District). These changes will be noted in a supplementation summary of changes submitted alongside the revised protocol.

A protocol amendment to version 2.0 of clinical protocol CNMAu8.205 was performed on 27November2019 to remove the ENCALS inclusion criteria, stratification language, and clarification on inconsistencies between sections of the protocol and the PD analysis plan. These changes will be noted in a supplementation summary of changes submitted alongside the revised protocol.

A protocol amendment to Version 3.0 of clinical protocol CNMAu8.205 was finalized on 03March2020 to increase the age range of participation and provide additional clarity surrounding study parameters. These changes will be noted in a supplementation summary of changes submitted alongside the revised protocol.

A protocol amendment to Version 4.0 of clinical protocol CNMAu8.205 was finalized on 08September2020 to include the optional Open-Label Extension Period of the trial. These changes will be noted in a supplemententation summary of changes submitted alongside the revised protocol.

A protocol amendment to Version 5.0 of clinical protocol CNMAu8.205 was finalized on 14May2021 to clarify presentation of the primary endpoint as the summed MUNIX(4) change instead of the average MUNIX(4) change. Forced vital capacity was moved from an exploratory endpoint to secondary endpoint in order to include a clinical endpoint in the analysis hierarchy. Additionally, on 27July2021 an administrative amendment was was finalized to correct the contact details for one of the participating Investigators. These changes will be noted in a supplemententation summary of changes submitted alongside the revised protocol.

A protocol amendment to Version 6.1 of the clinical protocol CNMAu8.205 was finalized on 04October2021 to extend the OLE phase beyond 48-weeks until discontinued by the Sponsor. These changes will be noted in a supplementation summary of changes submitted alongside the revised protocol.

14 APPENDICES

Appendices to this version include:

- American Heart Association: Recommendations for Blood Pressure Measurement in Humans and Experimental Animals (Pickering et al., 2005).
- Sponsor Outlier Criteria based upon the CTCAE (v5.0; November 27, 2017).

15 REFERENCES

Alix, J.J.P., Neuwirth, C., Gelder, L., Burkhardt, C., Castro, J., de Carvalho, M., Gawel, M., Goedee, S., Grosskreutz, J., Lenglet, T., et al. (2019). Assessment of the reliability of the motor unit size index (MUSIX) in single subject “round-robin” and multi-centre settings. Clin. Neurophysiol. Off. J. Int. Fed. Clin. Neurophysiol. *130*, 666–674.

D’Amico, E., Factor-Litvak, P., Santella, R.M., and Mitsumoto, H. (2013). Clinical perspective on oxidative stress in sporadic amyotrophic lateral sclerosis. Free Radic. Biol. Med. *65*, 509–527.

Angelova, P.R., and Abramov, A.Y. (2018). Role of mitochondrial ROS in the brain: from physiology to neurodegeneration. FEBS Lett. *592*, 692–702.

Arthur, K.C., Calvo, A., Price, T.R., Geiger, J.T., Chiò, A., and Traynor, B.J. (2016). Projected increase in amyotrophic lateral sclerosis from 2015 to 2040. Nat. Commun. *7*, 12408.

Berry, J.D., Miller, R., Moore, D.H., Cudkowicz, M.E., van den Berg, L.H., Kerr, D.A., Dong, Y., Ingersoll, E.W., and Archibald, D. (2013). The Combined Assessment of Function and Survival (CAFS): a new endpoint for ALS clinical trials. Amyotroph. Lateral Scler. Front. Degener. *14*, 162–168.

Blokhuis, A.M., Groen, E.J.N., Koppers, M., van den Berg, L.H., and Pasterkamp, R.J. (2013). Protein aggregation in amyotrophic lateral sclerosis. Acta Neuropathol. (Berl.) *125*, 777–794.

Bostock, H. (2016). Estimating motor unit numbers from a CMAP scan. Muscle Nerve *53*, 889–896.

Bozzo, F., Mirra, A., and Carrì, M.T. (2017). Oxidative stress and mitochondrial damage in the pathogenesis of ALS: New perspectives. Neurosci. Lett. *636*, 3–8.

Canosa, A., Pagani, M., Cistaro, A., Montuschi, A., Iazzolino, B., Fania, P., Cammarosano, S., Ilardi, A., Moglia, C., Calvo, A., et al. (2016). 18F-FDG-PET correlates of cognitive impairment in ALS. Neurology *86*, 44–49.

Cantó, C., Menzies, K.J., and Auwerx, J. (2015). NAD(+) Metabolism and the Control of Energy Homeostasis: A Balancing Act between Mitochondria and the Nucleus. Cell Metab. *22*, 31–53.

de Carvalho, M., Dengler, R., Eisen, A., England, J.D., Kaji, R., Kimura, J., Mills, K., Mitsumoto, H., Nodera, H., Shefner, J., et al. (2008). Electrodiagnostic criteria for diagnosis of ALS. Clin. Neurophysiol. Off. J. Int. Fed. Clin. Neurophysiol. *119*, 497–503.

Cheah, B.C., Vucic, S., Krishnan, A.V., Boland, R.A., and Kiernan, M.C. (2011). Neurophysiological index as a biomarker for ALS progression: validity of mixed effects models. Amyotroph. Lateral Scler. Off. Publ. World Fed. Neurol. Res. Group Mot. Neuron Dis. *12*, 33–38.

Chouinard, P.A., Meena, D.K., Whitwell, R.L., Hilchey, M.D., and Goodale, M.A. (2017). A TMS Investigation on the Role of Lateral Occipital Complex and Caudal Intraparietal Sulcus in the Perception of Object Form and Orientation. J Cogn Neurosci *29*, 881–895.

Chua, C.E.L., and Tang, B.L. (2013). Linking membrane dynamics and trafficking to autophagy and the unfolded protein response. J. Cell. Physiol. *228*, 1638–1640.

Conforti, L., Janeckova, L., Wagner, D., Mazzola, F., Cialabrini, L., Di Stefano, M., Orsomando, G., Magni, G., Bendotti, C., Smyth, N., et al. (2011). Reducing expression of NAD+ synthesizing enzyme NMNAT1 does not affect the rate of Wallerian degeneration. FEBS J *278*, 2666–2679.

Dalakas, M.C., Hatazawa, J., Brooks, R.A., and Di Chiro, G. (1987). Lowered cerebral glucose utilization in amyotrophic lateral sclerosis. Ann. Neurol. *22*, 580–586.

Deacon, R.M.J. (2013a). Measuring motor coordination in mice. J. Vis. Exp. JoVE e2609.

Deacon, R.M.J. (2013b). Measuring the strength of mice. J. Vis. Exp. JoVE.

Du, Y., Wen, Y., Guo, X., Hao, J., Wang, W., He, A., Fan, Q., Li, P., Liu, L., Liang, X., et al. (2018). A Genome-wide Expression Association Analysis Identifies Genes and Pathways Associated with Amyotrophic Lateral Sclerosis. Cell. Mol. Neurobiol. *38*, 635–639.

Dupuis, L., Pradat, P.-F., Ludolph, A.C., and Loeffler, J.-P. (2011). Energy metabolism in amyotrophic lateral sclerosis. Lancet Neurol. *10*, 75–82.

Ferrante, R.J., Browne, S.E., Shinobu, L.A., Bowling, A.C., Baik, M.J., MacGarvey, U., Kowall, N.W., Brown, R.H., and Beal, M.F. (1997). Evidence of increased oxidative damage in both sporadic and familial amyotrophic lateral sclerosis. J. Neurochem. *69*, 2064–2074.

Gladman, M., and Zinman, L. (2015). The economic impact of amyotrophic lateral sclerosis: a systematic review. Expert Rev. Pharmacoecon. Outcomes Res. *15*, 439–450.

Grant, C.M. (2008). Metabolic reconfiguration is a regulated response to oxidative stress. J. Biol. *7*, 1.

Haidet-Phillips, A.M., Hester, M.E., Miranda, C.J., Meyer, K., Braun, L., Frakes, A., Song, S., Likhite, S., Murtha, M.J., Foust, K.D., et al. (2011). Astrocytes from familial and sporadic ALS patients are toxic to motor neurons. Nat. Biotechnol. *29*, 824–828.

Hatzipetros, T., Kidd, J.D., Moreno, A.J., Thompson, K., Gill, A., and Vieira, F.G. (2015). A Quick Phenotypic Neurological Scoring System for Evaluating Disease Progression in the SOD1-G93A Mouse Model of ALS. J. Vis. Exp. JoVE.

Heiman-Patterson, T.D., Deitch, J.S., Blankenhorn, E.P., Erwin, K.L., Perreault, M.J., Alexander, B.K., Byers, N., Toman, I., and Alexander, G.M. (2005). Background and gender effects on survival in the TgN(SOD1-G93A)1Gur mouse model of ALS. J. Neurol. Sci. *236*, 1–7.

Hou, Y., Lautrup, S., Cordonnier, S., Wang, Y., Croteau, D.L., Zavala, E., Zhang, Y., Moritoh, K., O'Connell, J.F., Baptiste, B.A., et al. (2018). NAD(+) supplementation

normalizes key Alzheimer's features and DNA damage responses in a new AD mouse model with introduced DNA repair deficiency. Proc Natl Acad Sci U A *115*, E1876–E1885.

Huang, X., El-Sayed, I.H., Yi, X., and El-Sayed, M.A. (2005). Gold nanoparticles: catalyst for the oxidation of NADH to NAD(+). J Photochem Photobiol B *81*, 76–83.

Jacobsen, A.B., Bostock, H., Fuglsang-Frederiksen, A., Duez, L., Beniczky, S., Møller, A.T., Blicher, J.U., and Tankisi, H. (2017). Reproducibility, and sensitivity to motor unit loss in amyotrophic lateral sclerosis, of a novel MUNE method: MScanFit MUNE. Clin. Neurophysiol. Off. J. Int. Fed. Clin. Neurophysiol. *128*, 1380–1388.

Jacobsen, A.B., Kristensen, R.S., Witt, A., Kristensen, A.G., Duez, L., Beniczky, S., Fuglsang-Frederiksen, A., and Tankisi, H. (2018). The utility of motor unit number estimation methods versus quantitative motor unit potential analysis in diagnosis of ALS. Clin. Neurophysiol. Off. J. Int. Fed. Clin. Neurophysiol. *129*, 646–653.

Jacobsen, A.B., Bostock, H., and Tankisi, H. (2019). Following disease progression in motor neuron disorders with 3 motor unit number estimation methods. Muscle Nerve *59*, 82–87.

Jaiswal, M.K. (2018). Riluzole and edaravone: A tale of two amyotrophic lateral sclerosis drugs. Med. Res. Rev.

Jung, C., Higgins, C.M.J., and Xu, Z. (2002). Mitochondrial electron transport chain complex dysfunction in a transgenic mouse model for amyotrophic lateral sclerosis. J. Neurochem. *83*, 535–545.

Van Laere, K., Vanhee, A., Verschueren, J., De Coster, L., Driesen, A., Dupont, P., Robberecht, W., and Van Damme, P. (2014). Value of 18fluorodeoxyglucose-positron-emission tomography in amyotrophic lateral sclerosis: a prospective study. JAMA Neurol. *71*, 553–561.

Lederer, C.W., Torrisi, A., Pantelidou, M., Santama, N., and Cavallaro, S. (2007). Pathways and genes differentially expressed in the motor cortex of patients with sporadic amyotrophic lateral sclerosis. BMC Genomics *8*, 26.

Lu, M., Zhu, X.-H., and Chen, W. (2016). In vivo (31) P MRS assessment of intracellular NAD metabolites and NAD(+) /NADH redox state in human brain at 4 T. NMR Biomed. *29*, 1010–1017.

Lutz, C. (2018). Mouse models of ALS: Past, present and future. Brain Res. *1693*, 1–10.

Manfredi, G., and Xu, Z. (2005). Mitochondrial dysfunction and its role in motor neuron degeneration in ALS. Mitochondrion *5*, 77–87.

Maracchioni, A., Totaro, A., Angelini, D.F., Di Penta, A., Bernardi, G., Carrì, M.T., and Achsel, T. (2007). Mitochondrial damage modulates alternative splicing in neuronal cells: implications for neurodegeneration. J. Neurochem. *100*, 142–153.

Martinou, J.C., Martinou, I., and Kato, A.C. (1992). Cholinergic differentiation factor (CDF/LIF) promotes survival of isolated rat embryonic motoneurons in vitro. Neuron *8*, 737–744.

Mattiazzi, M., D'Aurelio, M., Gajewski, C.D., Martushova, K., Kiaei, M., Beal, M.F., and Manfredi, G. (2002). Mutated human SOD1 causes dysfunction of oxidative phosphorylation in mitochondria of transgenic mice. J. Biol. Chem. *277*, 29626–29633.

Menon, P., Kiernan, M.C., Yiannikas, C., Stroud, J., and Vucic, S. (2013). Split-hand index for the diagnosis of amyotrophic lateral sclerosis. Clin. Neurophysiol. Off. J. Int. Fed. Clin. Neurophysiol. *124*, 410–416.

Meyer, K., Ferraiuolo, L., Miranda, C.J., Likhite, S., McElroy, S., Renusch, S., Ditsworth, D., Lagier-Tourenne, C., Smith, R.A., Ravits, J., et al. (2014a). Direct conversion of patient fibroblasts demonstrates non-cell autonomous toxicity of astrocytes to motor neurons in familial and sporadic ALS. Proc. Natl. Acad. Sci. U. S. A. *111*, 829–832.

Meyer, K., Ferraiuolo, L., Miranda, C.J., Likhite, S., McElroy, S., Renusch, S., Ditsworth, D., Lagier-Tourenne, C., Smith, R.A., Ravits, J., et al. (2014b). Direct conversion of patient fibroblasts demonstrates non-cell autonomous toxicity of astrocytes to motor neurons in familial and sporadic ALS. Proc. Natl. Acad. Sci. U. S. A. *111*, 829–832.

Mitsumoto, H., Brooks, B.R., and Silani, V. (2014). Clinical trials in amyotrophic lateral sclerosis: why so many negative trials and how can trials be improved? Lancet Neurol. *13*, 1127–1138.

Nandedkar, S.D., Nandedkar, D.S., Barkhaus, P.E., and Stalberg, E.V. (2004). Motor unit number index (MUNIX). IEEE Trans. Biomed. Eng. *51*, 2209–2211.

Nandedkar, S.D., Barkhaus, P.E., and Stålberg, E.V. (2010). Motor unit number index (MUNIX): principle, method, and findings in healthy subjects and in patients with motor neuron disease. Muscle Nerve *42*, 798–807.

Neumann, M., Sampathu, D.M., Kwong, L.K., Truax, A.C., Micsenyi, M.C., Chou, T.T., Bruce, J., Schuck, T., Grossman, M., Clark, C.M., et al. (2006). Ubiquitinated TDP-43 in frontotemporal lobar degeneration and amyotrophic lateral sclerosis. Science *314*, 130–133.

Neuwirth, C., Nandedkar, S., Stålberg, E., and Weber, M. (2010). Motor unit number index (MUNIX): a novel neurophysiological technique to follow disease progression in amyotrophic lateral sclerosis. Muscle Nerve *42*, 379–384.

Neuwirth, C., Barkhaus, P.E., Burkhardt, C., Castro, J., Czell, D., de Carvalho, M., Nandedkar, S., Stålberg, E., and Weber, M. (2015). Tracking motor neuron loss in a set of six muscles in amyotrophic lateral sclerosis using the Motor Unit Number Index (MUNIX): a 15-month longitudinal multicentre trial. J. Neurol. Neurosurg. Psychiatry *86*, 1172–1179.

Neuwirth, C., Braun, N., Claeys, K.G., Bucelli, R., Fournier, C., Bromberg, M., Petri, S., Goedee, S., Lenglet, T., Leppanen, R., et al. (2018). Implementing Motor Unit Number Index (MUNIX) in a large clinical trial: Real world experience from 27 centres. Clin. Neurophysiol. Off. J. Int. Fed. Clin. Neurophysiol. *129*, 1756–1762.

Pickering, T.G., Hall, J.E., Appel, L.J., Falkner, B.E., Graves, J., Hill, M.N., Jones, D.W., Kurtz, T., Sheps, S.G., and Roccella, E.J. (2005). Recommendations for blood pressure measurement in humans and experimental animals: part 1: blood pressure measurement in humans: a statement for professionals from the Subcommittee of Professional and Public

Education of the American Heart Association Council on High Blood Pressure Research. Circulation *111*, 697–716.

Poesen, K., and Van Damme, P. (2018). Diagnostic and Prognostic Performance of Neurofilaments in ALS. Front. Neurol. *9*, 1167.

Posner, K., Brown, G.K., Stanley, B., Brent, D.A., Yershova, K.V., Oquendo, M.A., Currier, G.W., Melvin, G.A., Greenhill, L., Shen, S., et al. (2011). The Columbia-Suicide Severity Rating Scale: initial validity and internal consistency findings from three multisite studies with adolescents and adults. Am. J. Psychiatry *168*, 1266–1277.

Qian, K., Huang, H., Peterson, A., Hu, B., Maragakis, N.J., Ming, G.-L., Chen, H., and Zhang, S.-C. (2017). Sporadic ALS Astrocytes Induce Neuronal Degeneration In Vivo. Stem Cell Rep. *8*, 843–855.

Reddi, A.R., and Culotta, V.C. (2013). SOD1 Integrates Signals from Oxygen and Glucose to Repress Respiration. Cell *152*, 224–235.

Rone, M.B., Cui, Q.L., Fang, J., Wang, L.C., Zhang, J., Khan, D., Bedard, M., Almazan, G., Ludwin, S.K., Jones, R., et al. (2016). Oligodendrogliopathy in Multiple Sclerosis: Low Glycolytic Metabolic Rate Promotes Oligodendrocyte Survival. J Neurosci *36*, 4698–4707.

Sanchez-Ramos, C., Tierrez, A., Fabregat-Andrés, O., Wild, B., Sánchez-Cabo, F., Arduini, A., Dopazo, A., and Monsalve, M. (2011). PGC-1α regulates translocated in liposarcoma activity: role in oxidative stress gene expression. Antioxid. Redox Signal. *15*, 325–337.

Sasaki, Y., Vohra, B.P.S., Lund, F.E., and Milbrandt, J. (2009). Nicotinamide Mononucleotide Adenylyl Transferase-Mediated Axonal Protection Requires Enzymatic Activity But Not Increased Levels of Neuronal Nicotinamide Adenine Dinucleotide. J. Neurosci. *29*, 5525–5535.

Shepheard, S.R., Wuu, J., Cardoso, M., Wiklendt, L., Dinning, P.G., Chataway, T., Schultz, D., Benatar, M., and Rogers, M.-L. (2017). Urinary p75ECD: A prognostic, disease progression, and pharmacodynamic biomarker in ALS. Neurology *88*, 1137–1143.

Sholl, D.A. (1953). Dendritic organization in the neurons of the visual and motor cortices of the cat. J. Anat. *87*, 387–406.

Siklos, L., Engelhardt, J., Harati, Y., Smith, R.G., Joó, F., and Appel, S.H. (1996). Ultrastructural evidence for altered calcium in motor nerve terminals in amyotropic lateral sclerosis. Ann. Neurol. *39*, 203–216.

Stincone, A., Prigione, A., Cramer, T., Wamelink, M.M., Campbell, K., Cheung, E., Olin-Sandoval, V., Gruning, N.M., Kruger, A., Tauqeer Alam, M., et al. (2015). The return of metabolism: biochemistry and physiology of the pentose phosphate pathway. Biol Rev Camb Philos Soc *90*, 927–963.

Sydenstricker, V.P. (1958). The History of Pellagra, Its Recognition as a Disorder of Nutrition and Its Conquest. Am. J. Clin. Nutr. *6*, 409–414.

Szelechowski, M., Amoedo, N., Obre, E., Léger, C., Allard, L., Bonneu, M., Claverol, S., Lacombe, D., Oliet, S., Chevallier, S., et al. (2018). Metabolic Reprogramming in Amyotrophic Lateral Sclerosis. Sci. Rep. *8*, 3953.

Tefera, T.W., and Borges, K. (2016). Metabolic Dysfunctions in Amyotrophic Lateral Sclerosis Pathogenesis and Potential Metabolic Treatments. Front. Neurosci. *10*, 611.

Vandoorne, T., De Bock, K., and Van Den Bosch, L. (2018). Energy metabolism in ALS: an underappreciated opportunity? Acta Neuropathol. (Berl.) *135*, 489–509.

Villanueva-Paz, M., Cotán, D., Garrido-Maraver, J., Oropesa-Ávila, M., de la Mata, M., Delgado-Pavón, A., de Lavera, I., Alcocer-Gómez, E., Álvarez-Córdoba, M., and Sánchez-Alcázar, J.A. (2016). AMPK Regulation of Cell Growth, Apoptosis, Autophagy, and Bioenergetics. EXS *107*, 45–71.

Van Weehaeghe, D., Ceccarini, J., Delva, A., Robberecht, W., Van Damme, P., and Van Laere, K. (2016). Prospective Validation of 18F-FDG Brain PET Discriminant Analysis Methods in the Diagnosis of Amyotrophic Lateral Sclerosis. J. Nucl. Med. Off. Publ. Soc. Nucl. Med. *57*, 1238–1243.

Wiedemann, F.R., Manfredi, G., Mawrin, C., Beal, M.F., and Schon, E.A. (2002). Mitochondrial DNA and respiratory chain function in spinal cords of ALS patients. J. Neurochem. *80*, 616–625.

Zhang, T., Baldie, G., Periz, G., and Wang, J. (2014). RNA-processing protein TDP-43 regulates FOXO-dependent protein quality control in stress response. PLoS Genet. *10*, e1004693.

Zhao, W., Varghese, M., Yemul, S., Pan, Y., Cheng, A., Marano, P., Hassan, S., Vempati, P., Chen, F., Qian, X., et al. (2011). Peroxisome proliferator activator receptor gamma coactivator-1alpha (PGC-1α) improves motor performance and survival in a mouse model of amyotrophic lateral sclerosis. Mol. Neurodegener. *6*, 51.

Zhu, X.-H., Lu, M., Lee, B.-Y., Ugurbil, K., and Chen, W. (2015). In vivo NAD assay reveals the intracellular NAD contents and redox state in healthy human brain and their age dependences. Proc. Natl. Acad. Sci. *112*, 2876.
